# Supplementary material for: A methylation-phosphorylation switch controls EZH2 stability and hematopoiesis
Source: eLife. 2024 Feb 12;13:e86168. doi: 10.7554/eLife.86168 (PMC10901513; doi:10.7554/eLife.86168)

Figure 1B-EZH2

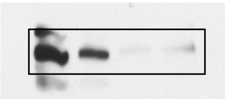

Figure 1B-SUZ12

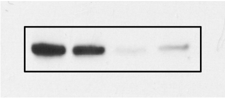

Figure 1B-EED

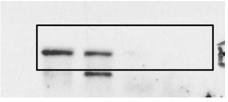

Figure 1B-LSD1

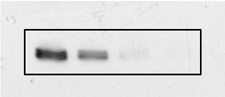

Figure 1B-Actin

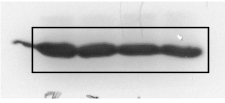

Figure 1D-EZH2

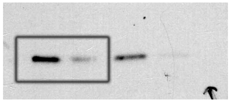

Figure 1D-SUZ12

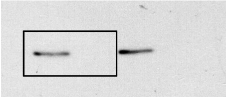

Figure 1D-EED

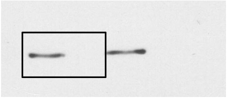

Figure 1D-LSD1

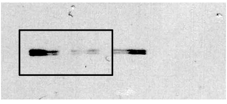

Figure 1D-Actin

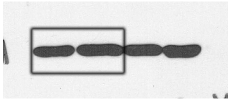

Figure 1D-H3K27me3

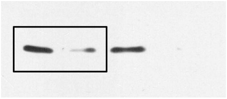

Figure 1D-H3

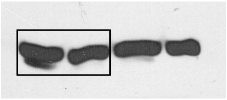

Figure 1E-EZH2

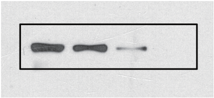

Figure 1E-LSD1

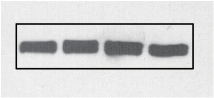

Figure 1E-Actin

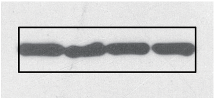

Figure 1F-EZH2

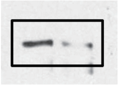

Figure 1F-LSD1

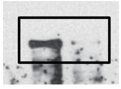

Figure 1F-H3K27me3

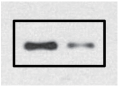

Figure 1F-H3

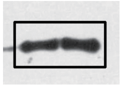

Figure 1G-EZH2

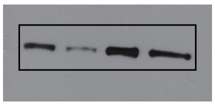

Figure 1G-LSD1

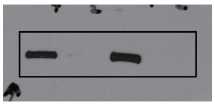

Figure 1G-Actin

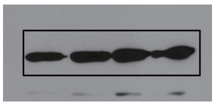

Supplement: Figure 1—source data 1. [file elife-86168-fig1-data1.zip › Figure 1 source data 1/Figure 1-annotated source data.pdf]
