# Supplementary figures and images for: A methylation-phosphorylation switch controls EZH2 stability and hematopoiesis (part 1 of 7)
Source: eLife. 2024 Feb 12;13:e86168. doi: 10.7554/eLife.86168 (PMC10901513; doi:10.7554/eLife.86168)

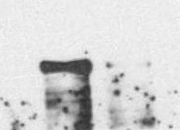

Supplement: Figure 1—source data 1. [file elife-86168-fig1-data1.zip › Figure 1 source data 1/Fig.1F pa-1 siLSD1 anti-LSD1 uncropped.tif]

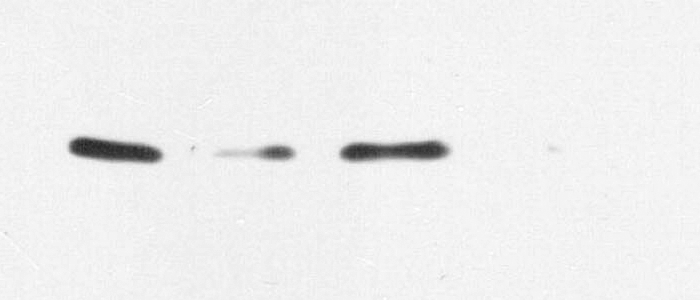

Supplement: Figure 1—source data 1. [file elife-86168-fig1-data1.zip › Figure 1 source data 1/Fig.1D actin-cretm-lsd1 MEF treated with 4-OH tam Anti-H3K27me3 uncropped.tif]

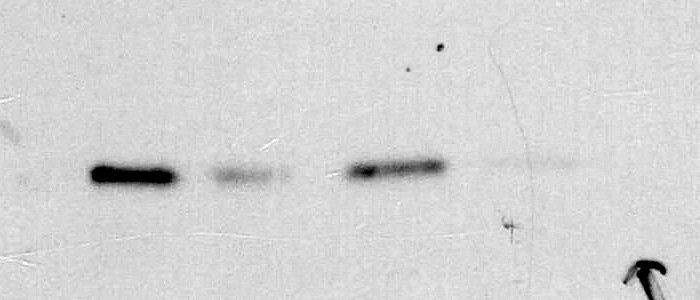

Supplement: Figure 1—source data 1. [file elife-86168-fig1-data1.zip › Figure 1 source data 1/Fig.1D actin-cretm-lsd1 MEF treated with 4-OH tam Anti-EZH2 uncropped.tif]

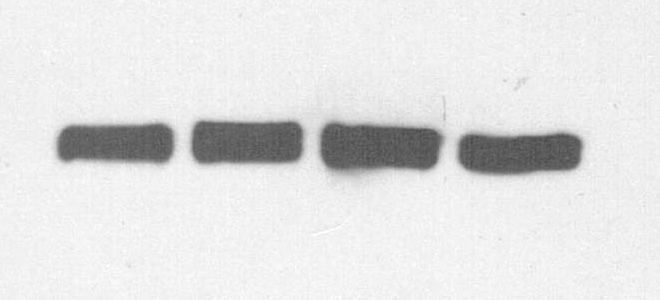

Supplement: Figure 1—source data 1. [file elife-86168-fig1-data1.zip › Figure 1 source data 1/Fig.1E 20220802 MEF WT Treated with cbb3001 for 15h check ezh2 anti-lsd1 uncropped.tif]

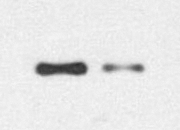

Supplement: Figure 1—source data 1. [file elife-86168-fig1-data1.zip › Figure 1 source data 1/Fig.1F pa-1 siLSD1 anti-H3K27me3 uncropped.tif]

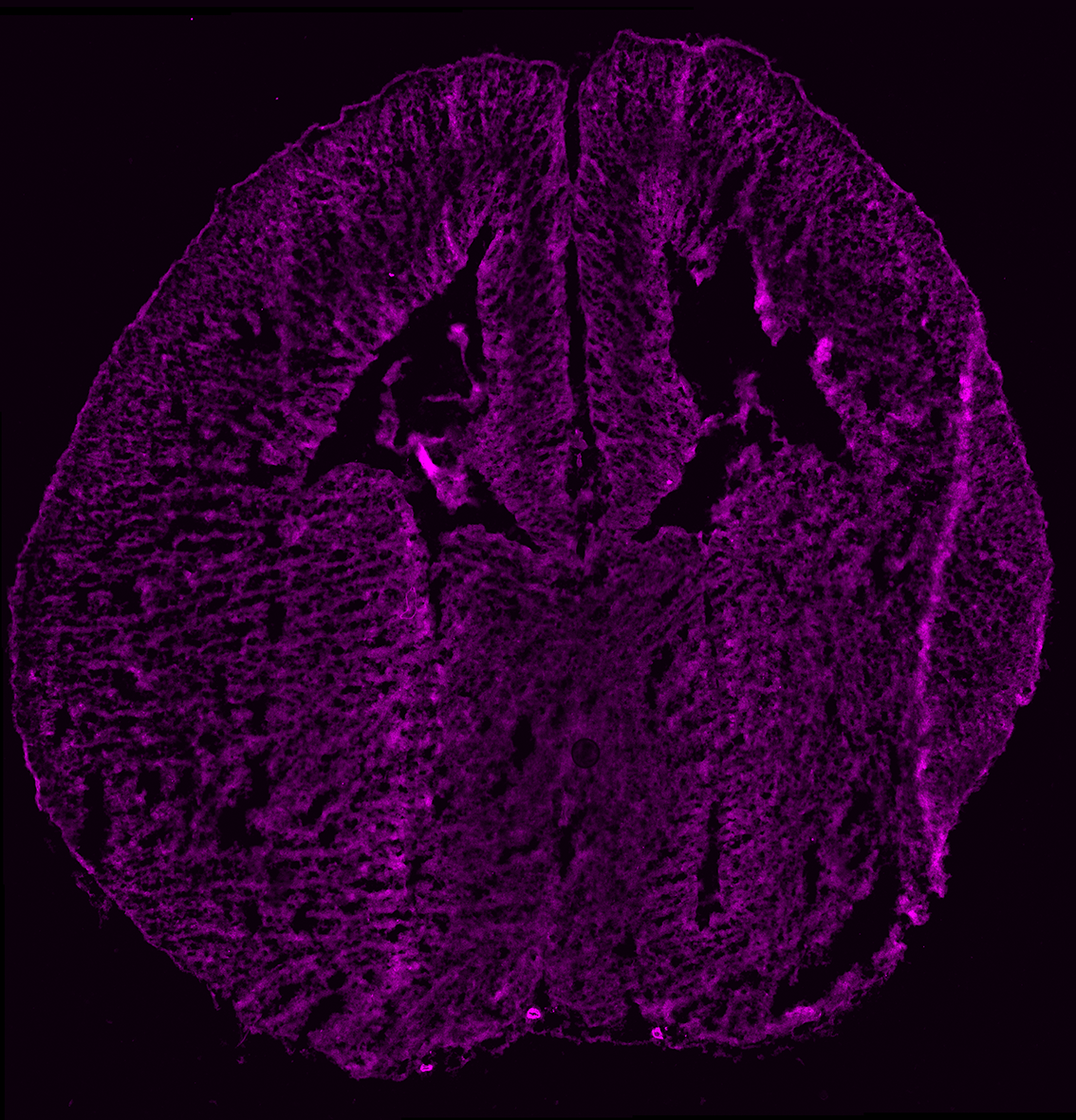

Supplement: Figure 1—source data 1. [file elife-86168-fig1-data1.zip › Figure 1 source data 1/Fig. 1A Nestin-lsd1 EZH2.tif]

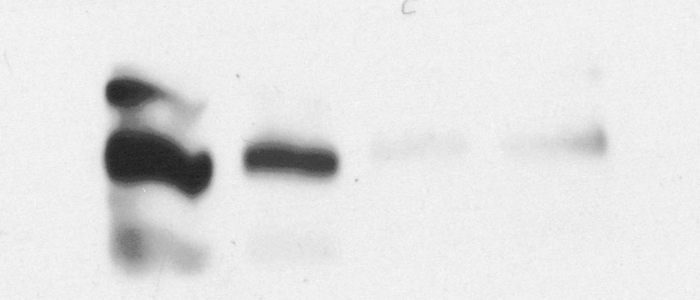

Supplement: Figure 1—source data 1. [file elife-86168-fig1-data1.zip › Figure 1 source data 1/Fig.1B P0 nestin-lsd1flox anti-EZH2 uncropped.tif]

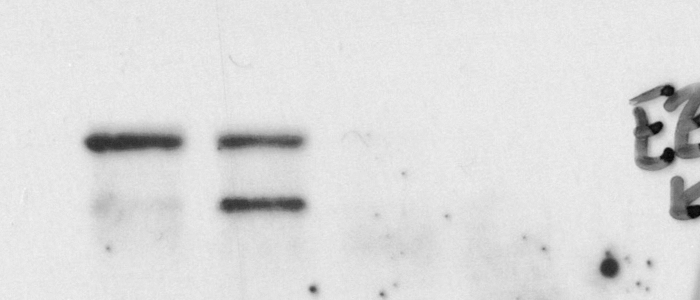

Supplement: Figure 1—source data 1. [file elife-86168-fig1-data1.zip › Figure 1 source data 1/Fig.1B P0 nestin-lsd1flox anti-eed uncropped .tif]

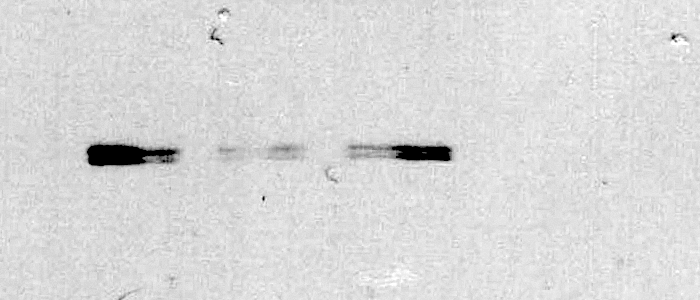

Supplement: Figure 1—source data 1. [file elife-86168-fig1-data1.zip › Figure 1 source data 1/Fig.1D actin-cretm-lsd1 MEF treated with 4-OH tam Anti-lsd1 uncropped.tif]

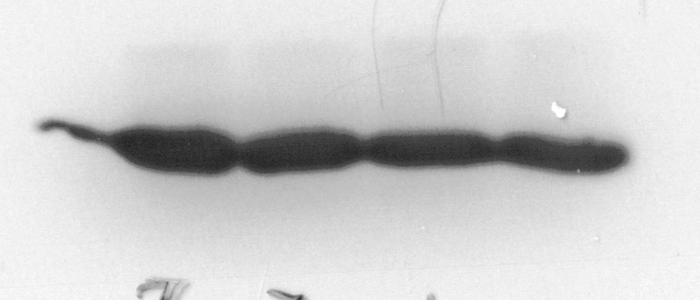

Supplement: Figure 1—source data 1. [file elife-86168-fig1-data1.zip › Figure 1 source data 1/Fig.1B P0 nestin-lsd1flox anti-Actin 1 uncropped.tif]

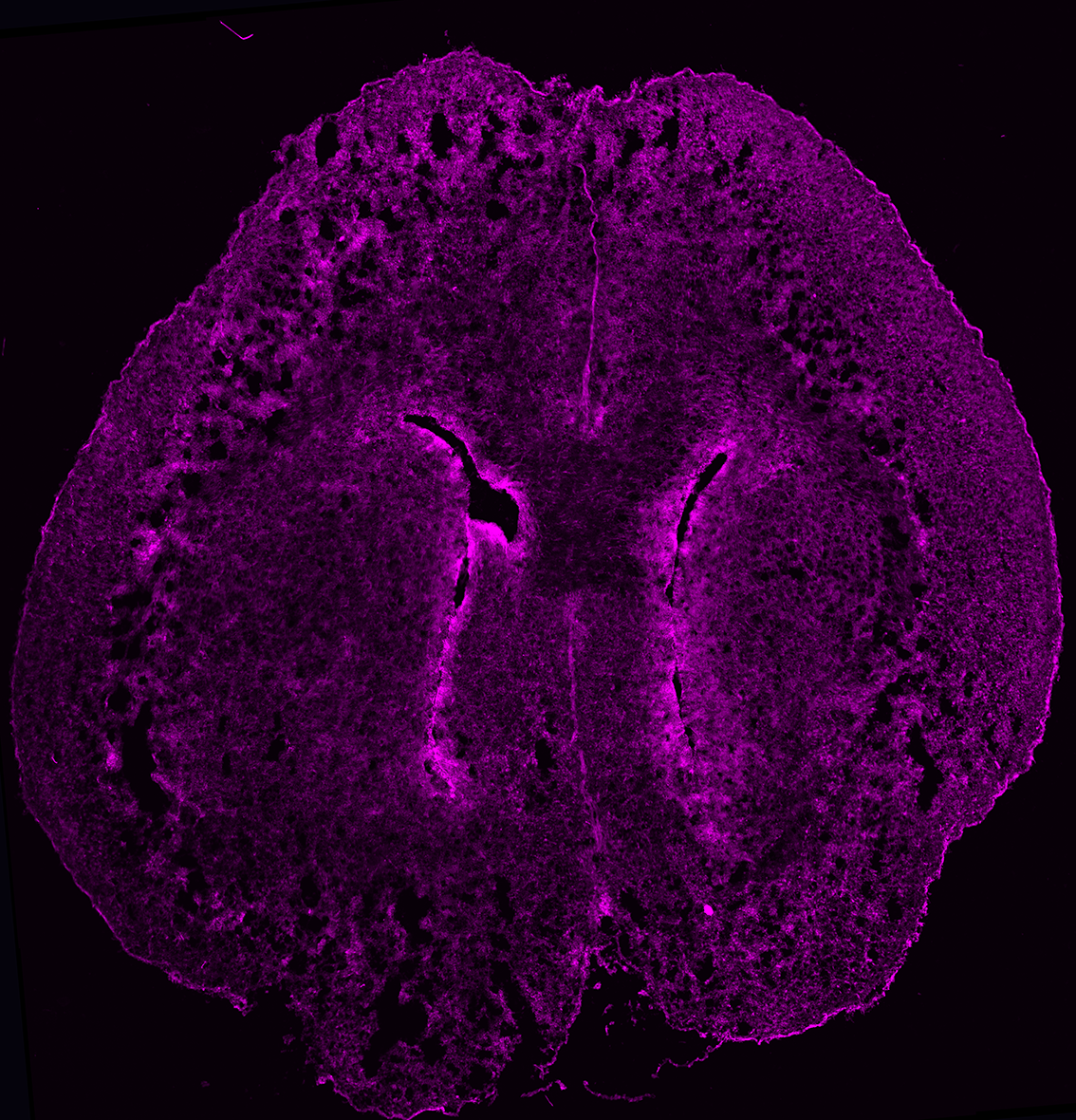

Supplement: Figure 1—source data 1. [file elife-86168-fig1-data1.zip › Figure 1 source data 1/Fig. 1A ctrl EZH2.tif]

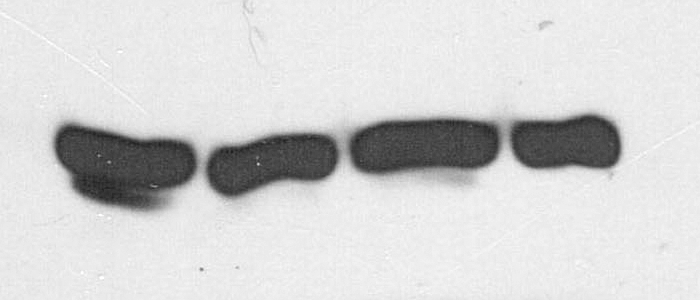

Supplement: Figure 1—source data 1. [file elife-86168-fig1-data1.zip › Figure 1 source data 1/Fig.1D actin-cretm-lsd1 MEF treated with 4-OH tam Anti-H3 uncropped.tif]

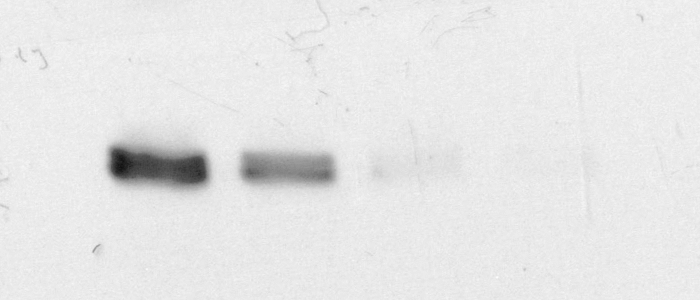

Supplement: Figure 1—source data 1. [file elife-86168-fig1-data1.zip › Figure 1 source data 1/Fig.1B P0 nestin-lsd1flox anti-LSD1 uncropped.tif]

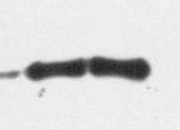

Supplement: Figure 1—source data 1. [file elife-86168-fig1-data1.zip › Figure 1 source data 1/Fig.1F pa-1 siLSD1 anti-H3 uncropped.tif]

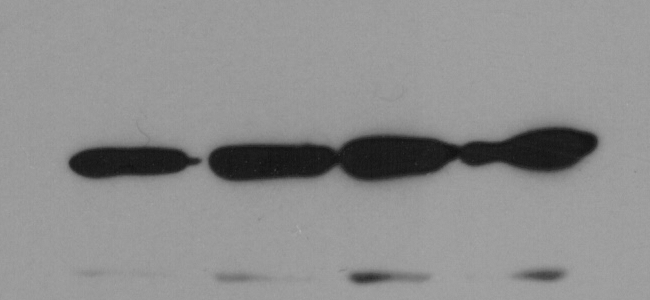

Supplement: Figure 1—source data 1. [file elife-86168-fig1-data1.zip › Figure 1 source data 1/Fig.1G Western Anti-actin.tif]

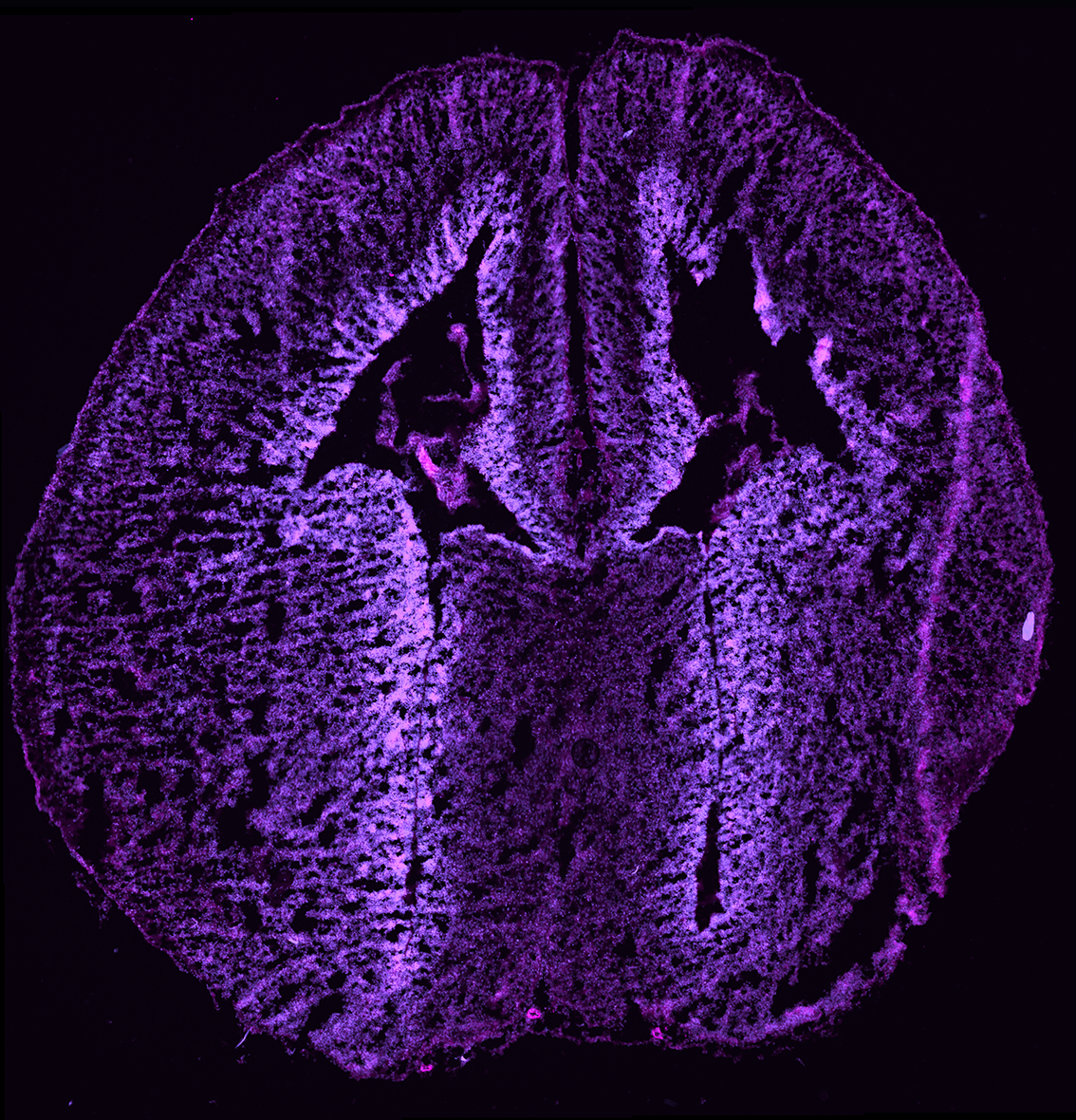

Supplement: Figure 1—source data 1. [file elife-86168-fig1-data1.zip › Figure 1 source data 1/Fig. 1A Nestin-lsd1 EZH2-DAPI.tif]

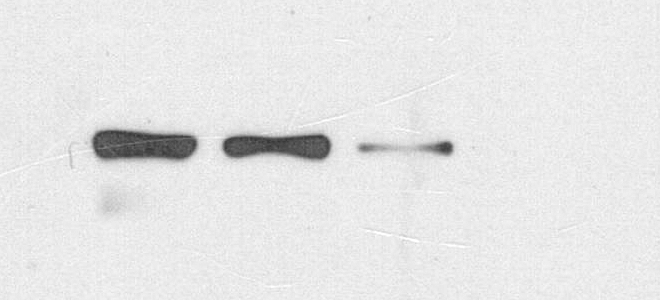

Supplement: Figure 1—source data 1. [file elife-86168-fig1-data1.zip › Figure 1 source data 1/Fig.1E 20220802 MEF WT Treated with cbb3001 for 15h check ezh2 anti-ezh2 uncropped.tif]

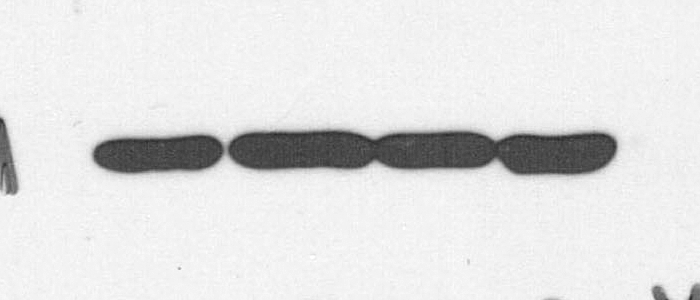

Supplement: Figure 1—source data 1. [file elife-86168-fig1-data1.zip › Figure 1 source data 1/Fig.1D actin-cretm-lsd1 MEF treated with 4-OH tam Anti-actin uncropped .tif]

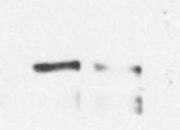

Supplement: Figure 1—source data 1. [file elife-86168-fig1-data1.zip › Figure 1 source data 1/Fig.1F pa-1 siLSD1 anti-EZH2 uncropped.tif]

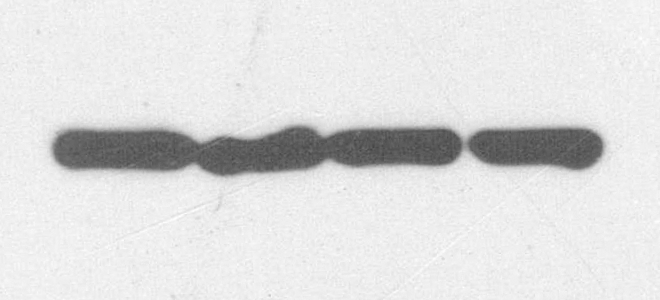

Supplement: Figure 1—source data 1. [file elife-86168-fig1-data1.zip › Figure 1 source data 1/Fig.1E 20220802 MEF WT Treated with cbb3001 for 15h check ezh2 anti-actin uncropped.tif]

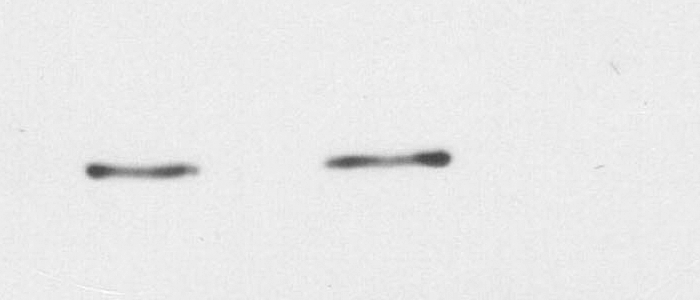

Supplement: Figure 1—source data 1. [file elife-86168-fig1-data1.zip › Figure 1 source data 1/Fig.1D actin-cretm-lsd1 MEF treated with 4-OH tam Anti-eed uncropped.tif]

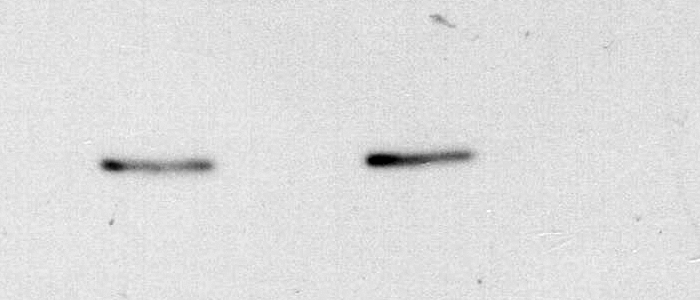

Supplement: Figure 1—source data 1. [file elife-86168-fig1-data1.zip › Figure 1 source data 1/Fig.1D actin-cretm-lsd1 MEF treated with 4-OH tam Anti-suz12 uncropped.tif]

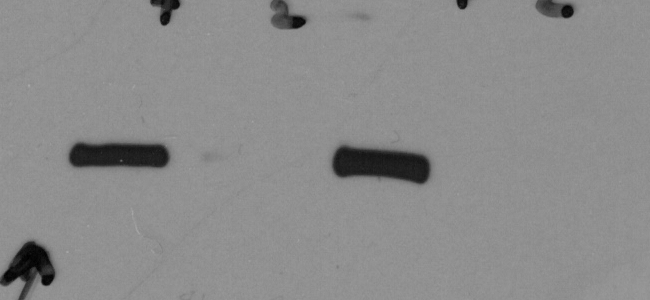

Supplement: Figure 1—source data 1. [file elife-86168-fig1-data1.zip › Figure 1 source data 1/Fig.1G Western Anti-LSD1.tif]

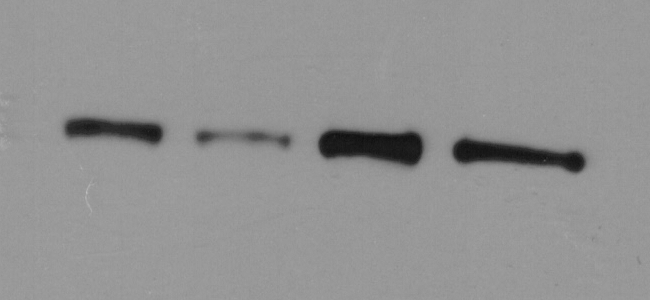

Supplement: Figure 1—source data 1. [file elife-86168-fig1-data1.zip › Figure 1 source data 1/Fig.1G Western Anti-EZH2.tif]

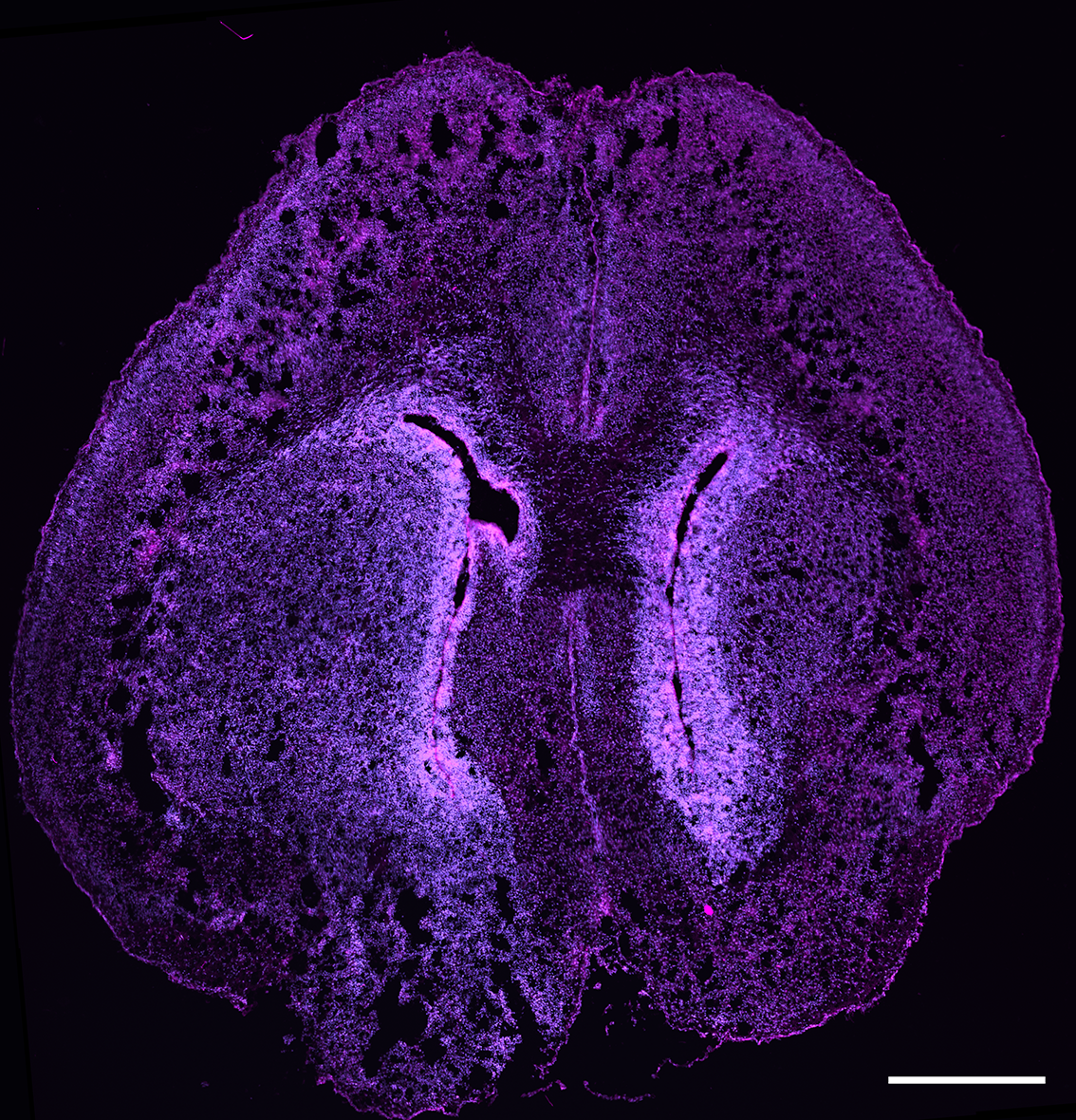

Supplement: Figure 1—source data 1. [file elife-86168-fig1-data1.zip › Figure 1 source data 1/Fig. 1A ctrl EZH2-DAPI.tif]

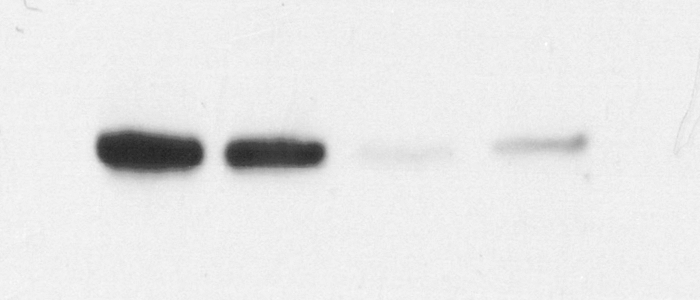

Supplement: Figure 1—source data 1. [file elife-86168-fig1-data1.zip › Figure 1 source data 1/Fig.1B P0 nestin-lsd1flox anti-SUZ12 uncropped.tif]

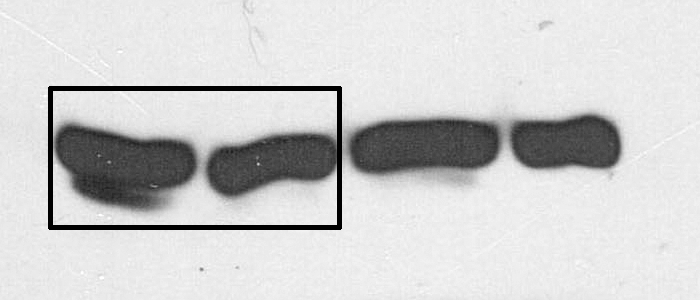

Supplement: Figure 1—source data 1. [file elife-86168-fig1-data1.zip › Figure 1 source data 1/annotated/Fig.1D actin-cretm-lsd1 MEF treated with 4-OH tam Anti-H3 uncropped.tif]

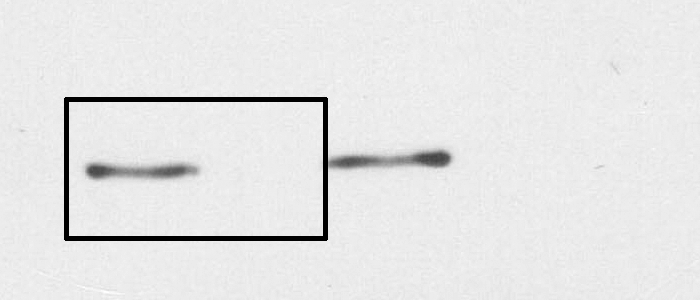

Supplement: Figure 1—source data 1. [file elife-86168-fig1-data1.zip › Figure 1 source data 1/annotated/Fig.1D actin-cretm-lsd1 MEF treated with 4-OH tam Anti-eed uncropped.tif]

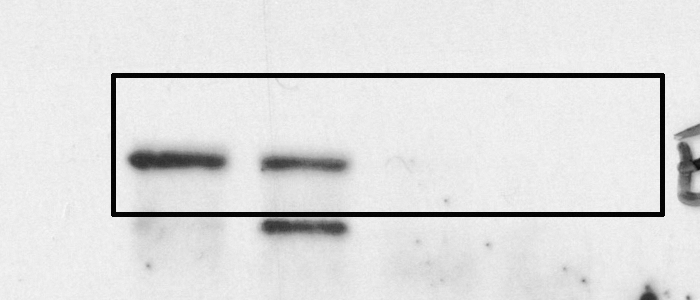

Supplement: Figure 1—source data 1. [file elife-86168-fig1-data1.zip › Figure 1 source data 1/annotated/Fig.1B P0 nestin-lsd1flox anti-eed uncropped.tif]

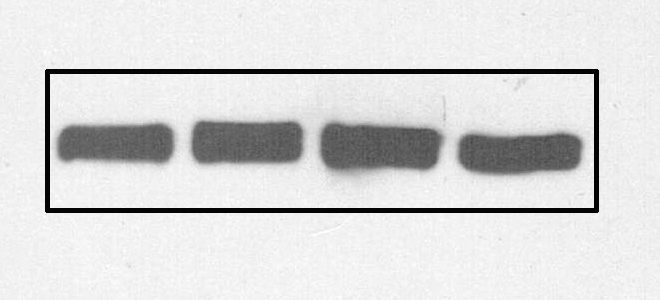

Supplement: Figure 1—source data 1. [file elife-86168-fig1-data1.zip › Figure 1 source data 1/annotated/Fig.1E 20220802 MEF WT Treated with cbb3001 for 15h check ezh2 anti-lsd1 uncropped.tif]

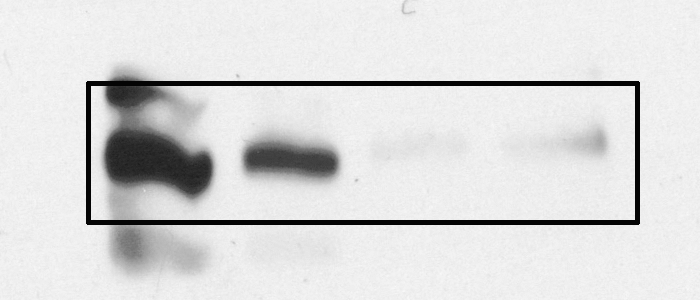

Supplement: Figure 1—source data 1. [file elife-86168-fig1-data1.zip › Figure 1 source data 1/annotated/Fig.1B P0 nestin-lsd1flox anti-EZH2 uncropped.tif]

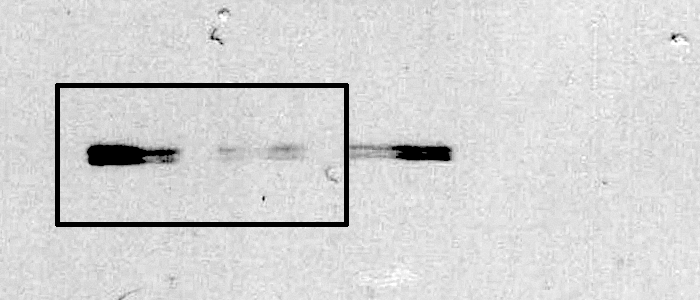

Supplement: Figure 1—source data 1. [file elife-86168-fig1-data1.zip › Figure 1 source data 1/annotated/Fig.1D actin-cretm-lsd1 MEF treated with 4-OH tam Anti-lsd1 uncropped.tif]

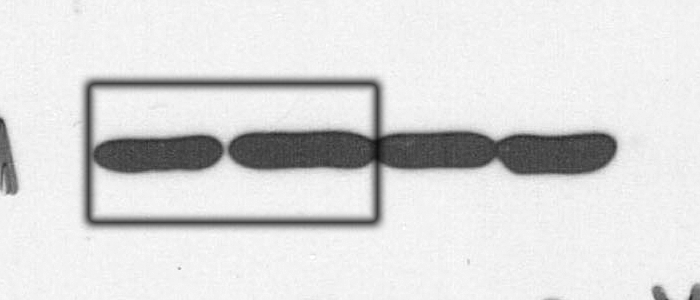

Supplement: Figure 1—source data 1. [file elife-86168-fig1-data1.zip › Figure 1 source data 1/annotated/Fig.1D actin-cretm-lsd1 MEF treated with 4-OH tam Anti-actin uncropped.tif]

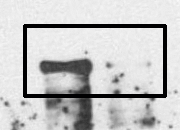

Supplement: Figure 1—source data 1. [file elife-86168-fig1-data1.zip › Figure 1 source data 1/annotated/Fig.1F pa-1 siLSD1 anti-LSD1 uncropped.tif]

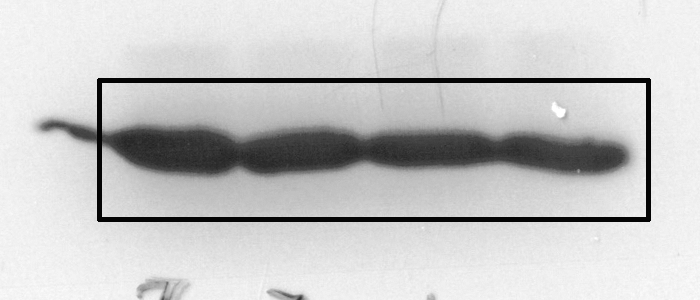

Supplement: Figure 1—source data 1. [file elife-86168-fig1-data1.zip › Figure 1 source data 1/annotated/Fig.1B P0 nestin-lsd1flox anti-actin uncropped.tif]

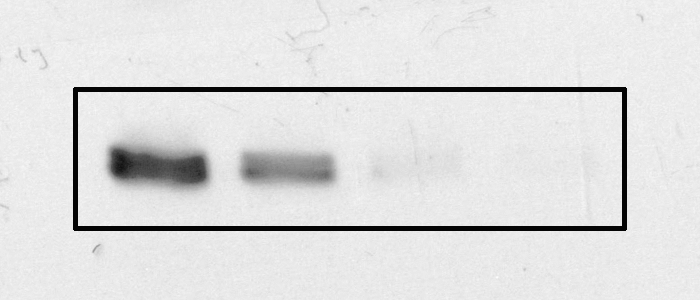

Supplement: Figure 1—source data 1. [file elife-86168-fig1-data1.zip › Figure 1 source data 1/annotated/Fig.1B P0 nestin-lsd1flox anti-LSD1 uncropped.tif]

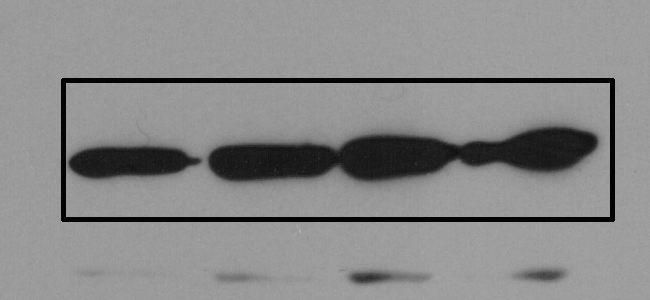

Supplement: Figure 1—source data 1. [file elife-86168-fig1-data1.zip › Figure 1 source data 1/annotated/Fig.1G Western Anti-actin.tif]

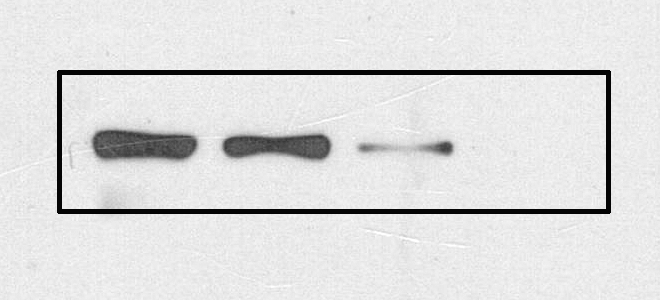

Supplement: Figure 1—source data 1. [file elife-86168-fig1-data1.zip › Figure 1 source data 1/annotated/Fig.1E 20220802 MEF WT Treated with cbb3001 for 15h check ezh2 anti-ezh2 uncropped.tif]

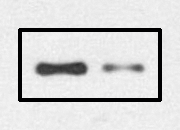

Supplement: Figure 1—source data 1. [file elife-86168-fig1-data1.zip › Figure 1 source data 1/annotated/Fig.1F pa-1 siLSD1 anti-H3K27me3 uncropped.tif]

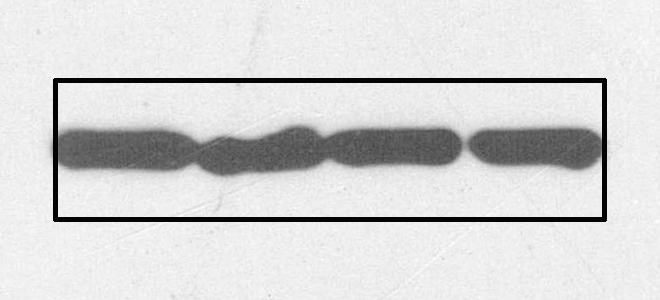

Supplement: Figure 1—source data 1. [file elife-86168-fig1-data1.zip › Figure 1 source data 1/annotated/Fig.1E 20220802 MEF WT Treated with cbb3001 for 15h check ezh2 anti-actin uncropped.tif]

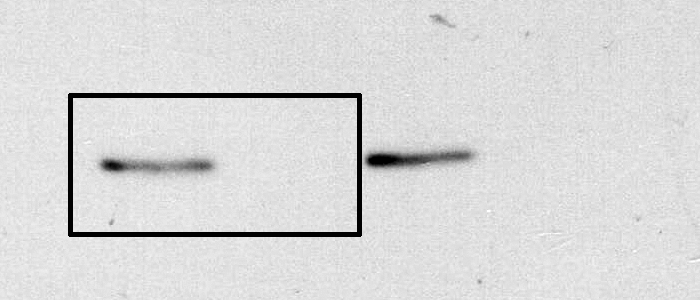

Supplement: Figure 1—source data 1. [file elife-86168-fig1-data1.zip › Figure 1 source data 1/annotated/Fig.1D actin-cretm-lsd1 MEF treated with 4-OH tam Anti-suz12 uncropped.tif]

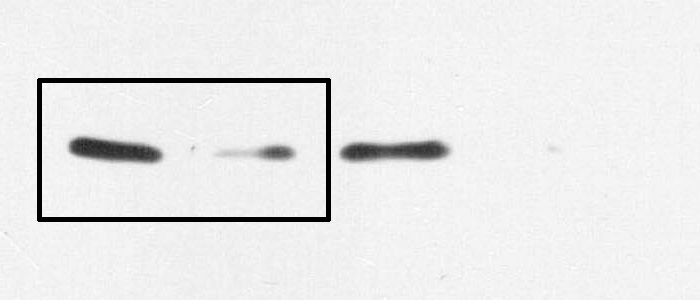

Supplement: Figure 1—source data 1. [file elife-86168-fig1-data1.zip › Figure 1 source data 1/annotated/Fig.1D actin-cretm-lsd1 MEF treated with 4-OH tam Anti-H3K27me3 uncropped.tif]

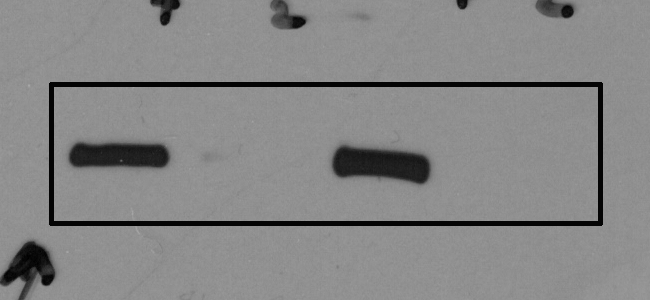

Supplement: Figure 1—source data 1. [file elife-86168-fig1-data1.zip › Figure 1 source data 1/annotated/Fig.1G Western Anti-LSD1.tif]

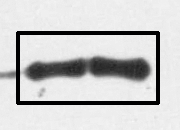

Supplement: Figure 1—source data 1. [file elife-86168-fig1-data1.zip › Figure 1 source data 1/annotated/Fig.1F pa-1 siLSD1 anti-H3 uncropped.tif]

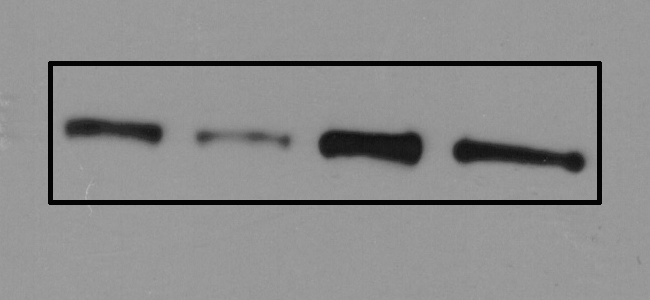

Supplement: Figure 1—source data 1. [file elife-86168-fig1-data1.zip › Figure 1 source data 1/annotated/Fig.1G Western Anti-EZH2.tif]

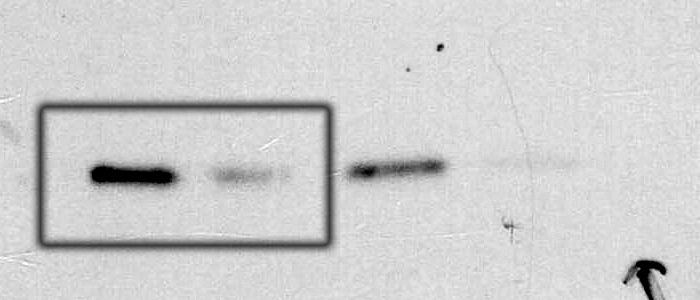

Supplement: Figure 1—source data 1. [file elife-86168-fig1-data1.zip › Figure 1 source data 1/annotated/Fig.1D actin-cretm-lsd1 MEF treated with 4-OH tam Anti-EZH2 uncropped.tif]

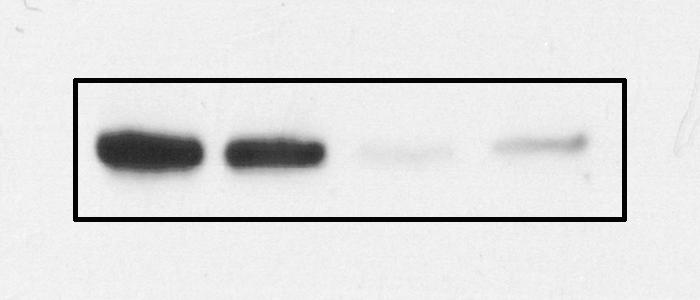

Supplement: Figure 1—source data 1. [file elife-86168-fig1-data1.zip › Figure 1 source data 1/annotated/Fig.1B P0 nestin-lsd1flox anti-SUZ12 uncropped.tif]

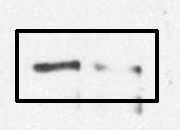

Supplement: Figure 1—source data 1. [file elife-86168-fig1-data1.zip › Figure 1 source data 1/annotated/Fig.1F pa-1 siLSD1 anti-EZH2 uncropped.tif]

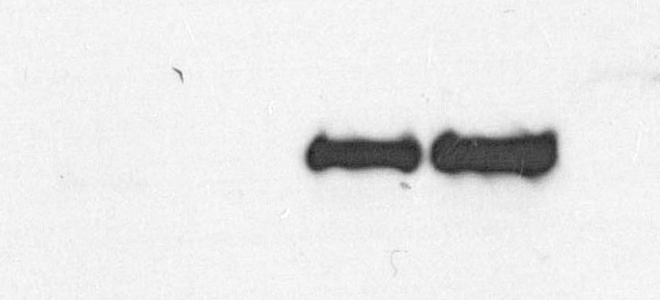

Supplement: Figure 1—figure supplement 1—source data 1. [file elife-86168-fig1-figsupp1-data1.zip › Figure 1-figure supplement 1 source data 1/figure supplement 1B H1299 wt -lsd1 si-LSD1-3-utr Check EZH2 rep.2 anti-flag-lsd1 uncropped.tif]

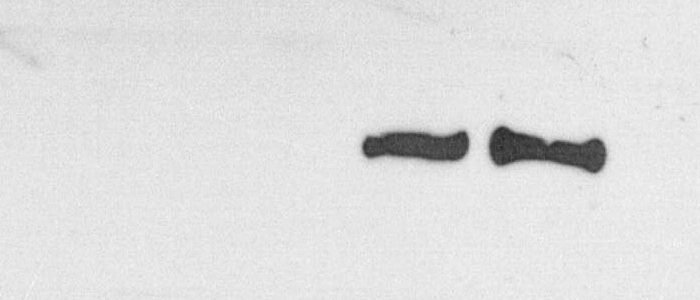

Supplement: Figure 1—figure supplement 1—source data 1. [file elife-86168-fig1-figsupp1-data1.zip › Figure 1-figure supplement 1 source data 1/figure supplement 1C cell expression LSD1-531AA mutant si LSD1 anti-Flag-LSD1-531aa uncropped.tif]

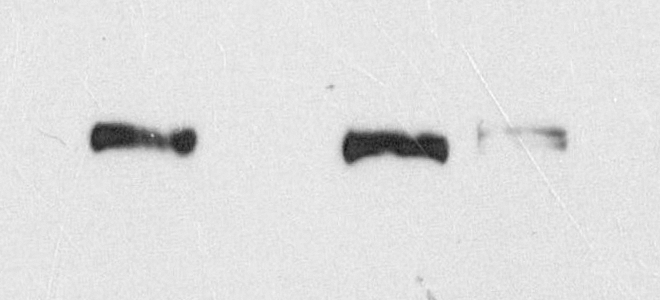

Supplement: Figure 1—figure supplement 1—source data 1. [file elife-86168-fig1-figsupp1-data1.zip › Figure 1-figure supplement 1 source data 1/figure supplement 1B H1299 wt -lsd1 si-LSD1-3-utr Check EZH2 rep.2 anti-lsd1 uncropped.tif]

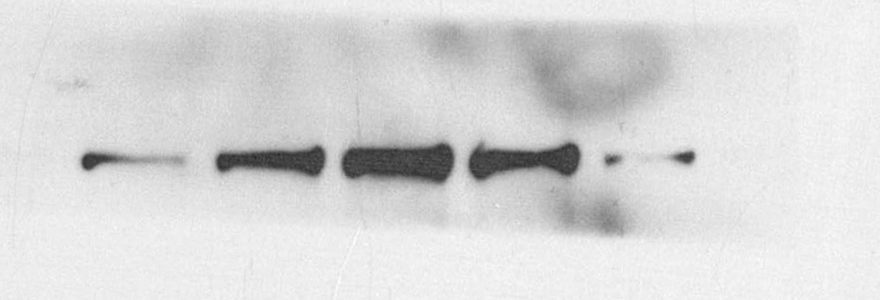

Supplement: Figure 1—figure supplement 1—source data 1. [file elife-86168-fig1-figsupp1-data1.zip › Figure 1-figure supplement 1 source data 1/figure supplement 1A H520 si LSD1 -1 -3utr anti-ezh2 uncropped.tif]

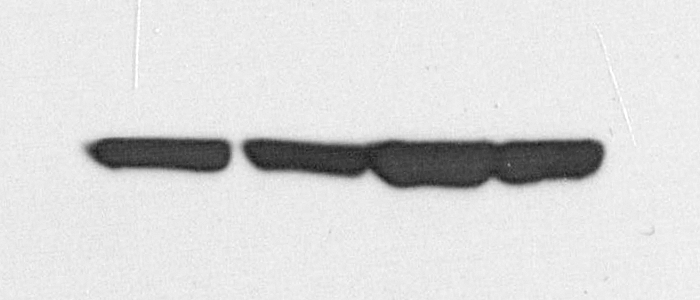

Supplement: Figure 1—figure supplement 1—source data 1. [file elife-86168-fig1-figsupp1-data1.zip › Figure 1-figure supplement 1 source data 1/figure supplement 1C cell expression LSD1-531AA mutant si LSD1 anti-gapdh uncropped.tif]

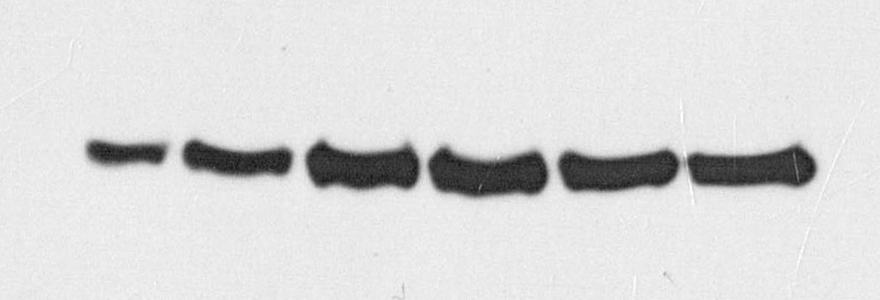

Supplement: Figure 1—figure supplement 1—source data 1. [file elife-86168-fig1-figsupp1-data1.zip › Figure 1-figure supplement 1 source data 1/figure supplement 1A H520 si LSD1 -1 -3utr anti-H3 uncropped.tif]

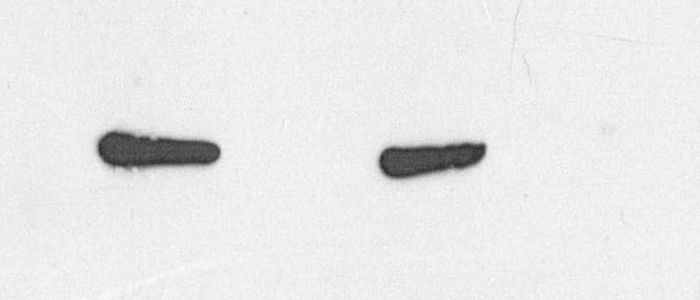

Supplement: Figure 1—figure supplement 1—source data 1. [file elife-86168-fig1-figsupp1-data1.zip › Figure 1-figure supplement 1 source data 1/figure supplement 1C cell expression LSD1-531AA mutant si LSD1 anti-EZH2 uncropped.tif]

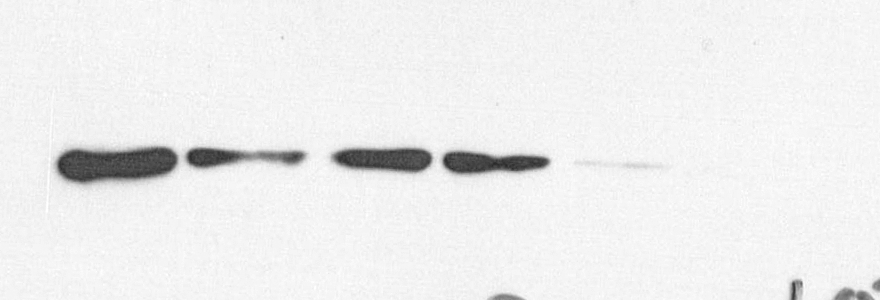

Supplement: Figure 1—figure supplement 1—source data 1. [file elife-86168-fig1-figsupp1-data1.zip › Figure 1-figure supplement 1 source data 1/figure supplement 1A H520 si LSD1 -1 -3utr anti-LSD1 uncropped.tif]

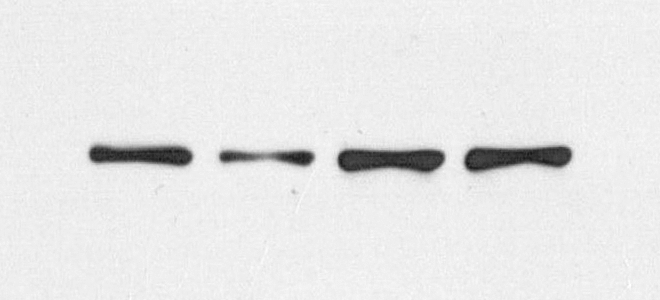

Supplement: Figure 1—figure supplement 1—source data 1. [file elife-86168-fig1-figsupp1-data1.zip › Figure 1-figure supplement 1 source data 1/figure supplement 1B H1299 wt -lsd1 si-LSD1-3-utr Check EZH2 rep.2 anti-EZH2 uncropped.tif]

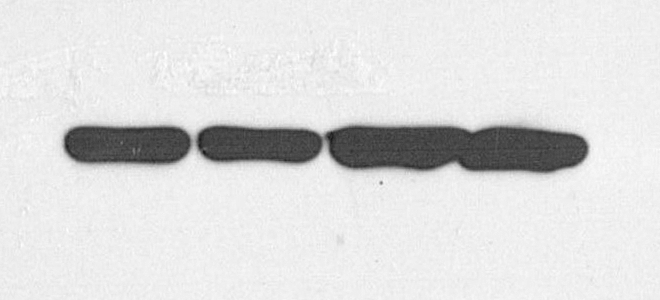

Supplement: Figure 1—figure supplement 1—source data 1. [file elife-86168-fig1-figsupp1-data1.zip › Figure 1-figure supplement 1 source data 1/figure supplement 1B H1299 wt -lsd1 si-LSD1-3-utr Check EZH2 rep.2 anti-GAPDH uncropped.tif]

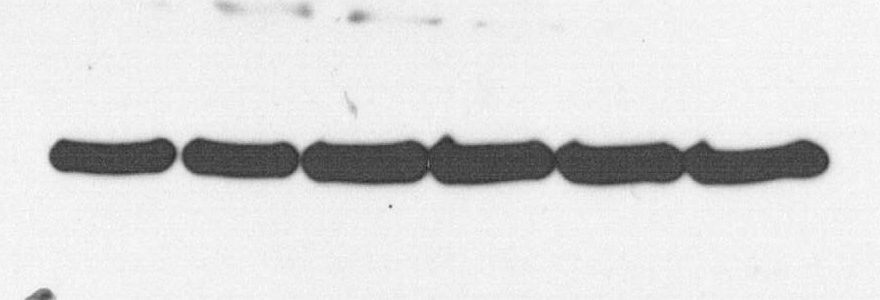

Supplement: Figure 1—figure supplement 1—source data 1. [file elife-86168-fig1-figsupp1-data1.zip › Figure 1-figure supplement 1 source data 1/figure supplement 1A H520 si LSD1 -1 -3utr anti-gapdh uncropped.tif]

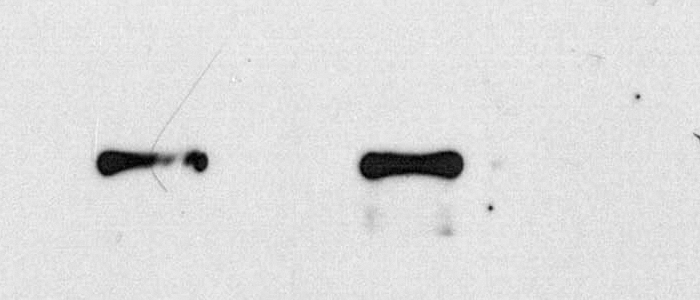

Supplement: Figure 1—figure supplement 1—source data 1. [file elife-86168-fig1-figsupp1-data1.zip › Figure 1-figure supplement 1 source data 1/figure supplement 1C cell expression LSD1-531AA mutant si LSD1 anti-LSD1 uncropped.tif]

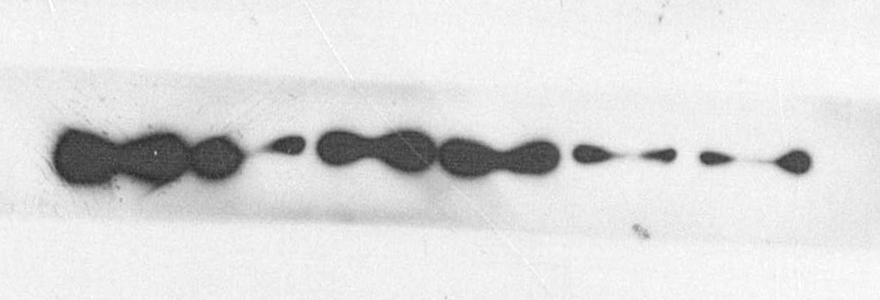

Supplement: Figure 1—figure supplement 1—source data 1. [file elife-86168-fig1-figsupp1-data1.zip › Figure 1-figure supplement 1 source data 1/figure supplement 1A H520 si LSD1 -1 -3utr anti-H3K27me3 uncropped.tif]

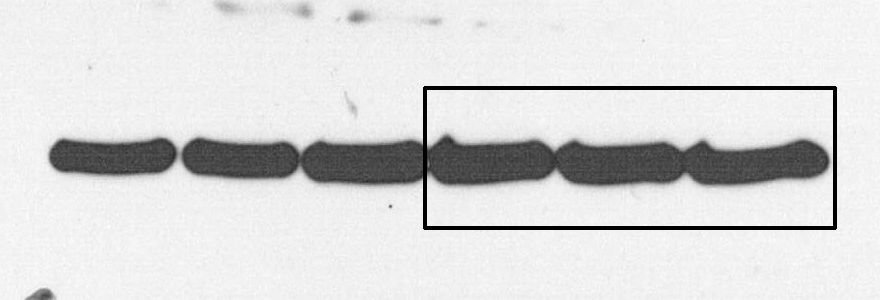

Supplement: Figure 1—figure supplement 1—source data 1. [file elife-86168-fig1-figsupp1-data1.zip › Figure 1-figure supplement 1 source data 1/annotated/figure supplement 1A H520 si LSD1 -1 -3utr anti-gapdh uncropped.tif]

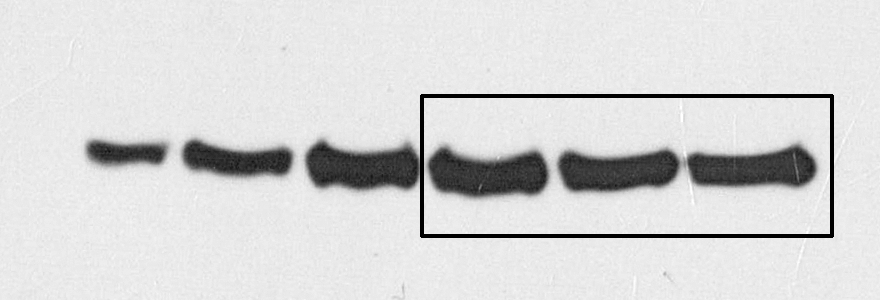

Supplement: Figure 1—figure supplement 1—source data 1. [file elife-86168-fig1-figsupp1-data1.zip › Figure 1-figure supplement 1 source data 1/annotated/figure supplement 1A H520 si LSD1 -1 -3utr anti-H3 uncropped.tif]

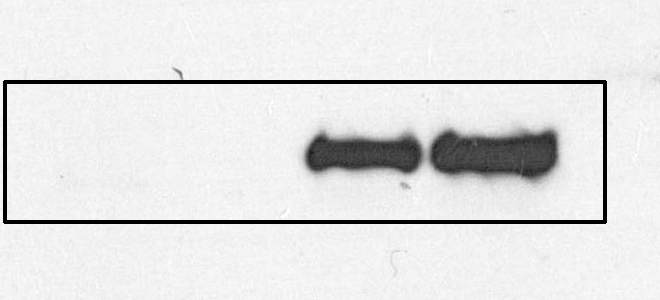

Supplement: Figure 1—figure supplement 1—source data 1. [file elife-86168-fig1-figsupp1-data1.zip › Figure 1-figure supplement 1 source data 1/annotated/figure supplement 1B H1299 wt -lsd1 si-LSD1-3-utr Check EZH2 rep.2 anti-flag-lsd1 uncropped.tif]

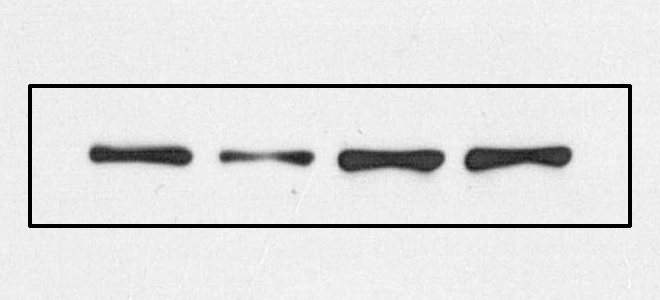

Supplement: Figure 1—figure supplement 1—source data 1. [file elife-86168-fig1-figsupp1-data1.zip › Figure 1-figure supplement 1 source data 1/annotated/figure supplement 1B H1299 wt -lsd1 si-LSD1-3-utr Check EZH2 rep.2 anti-EZH2 uncropped.tif]

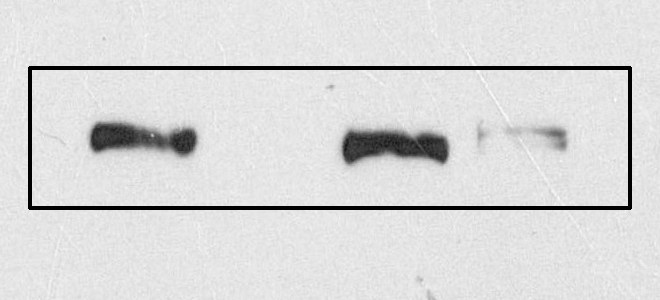

Supplement: Figure 1—figure supplement 1—source data 1. [file elife-86168-fig1-figsupp1-data1.zip › Figure 1-figure supplement 1 source data 1/annotated/figure supplement 1B H1299 wt -lsd1 si-LSD1-3-utr Check EZH2 rep.2 anti-lsd1 uncropped.tif]

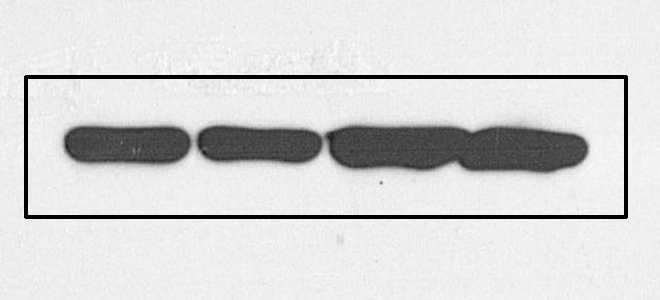

Supplement: Figure 1—figure supplement 1—source data 1. [file elife-86168-fig1-figsupp1-data1.zip › Figure 1-figure supplement 1 source data 1/annotated/figure supplement 1B H1299 wt -lsd1 si-LSD1-3-utr Check EZH2 rep.2 anti-GAPDH uncropped.tif]

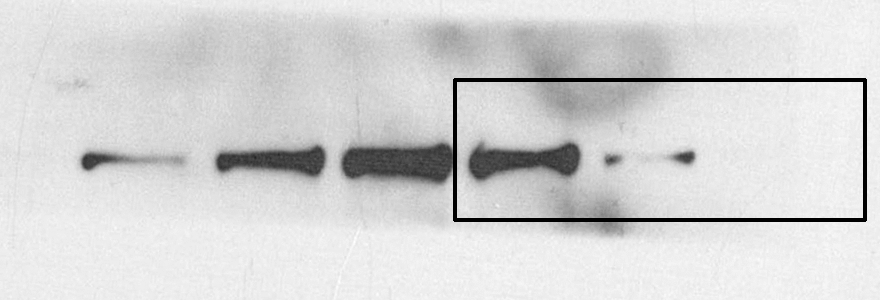

Supplement: Figure 1—figure supplement 1—source data 1. [file elife-86168-fig1-figsupp1-data1.zip › Figure 1-figure supplement 1 source data 1/annotated/figure supplement 1A H520 si LSD1 -1 -3utr anti-ezh2 uncropped.tif]

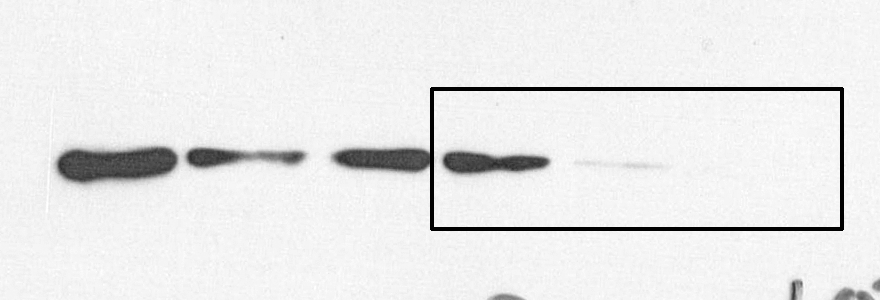

Supplement: Figure 1—figure supplement 1—source data 1. [file elife-86168-fig1-figsupp1-data1.zip › Figure 1-figure supplement 1 source data 1/annotated/figure supplement 1A H520 si LSD1 -1 -3utr anti-LSD1 uncropped.tif]

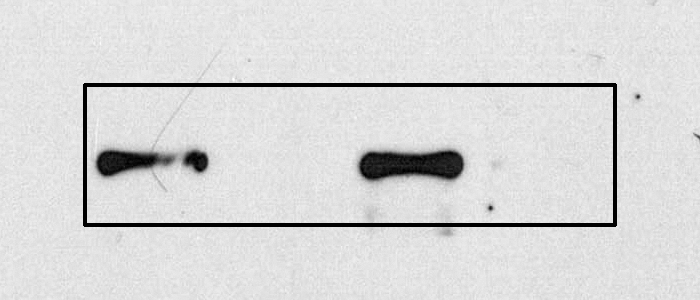

Supplement: Figure 1—figure supplement 1—source data 1. [file elife-86168-fig1-figsupp1-data1.zip › Figure 1-figure supplement 1 source data 1/annotated/figure supplement 1C cell expression LSD1-531AA mutant si LSD1 anti-LSD1 uncropped.tif]

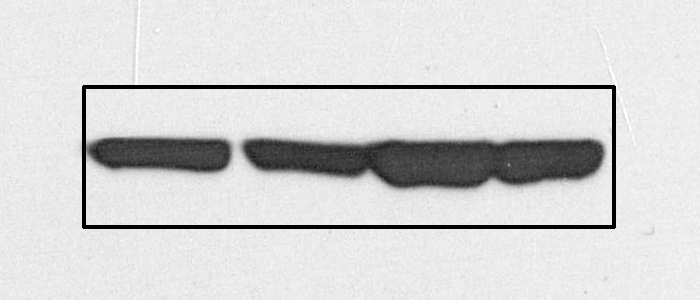

Supplement: Figure 1—figure supplement 1—source data 1. [file elife-86168-fig1-figsupp1-data1.zip › Figure 1-figure supplement 1 source data 1/annotated/figure supplement 1C cell expression LSD1-531AA mutant si LSD1 anti-gapdh uncropped.tif]

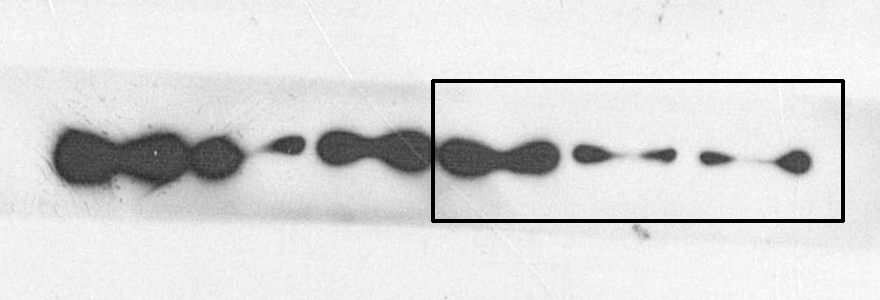

Supplement: Figure 1—figure supplement 1—source data 1. [file elife-86168-fig1-figsupp1-data1.zip › Figure 1-figure supplement 1 source data 1/annotated/figure supplement 1A H520 si LSD1 -1 -3utr anti-H3K27me3 uncropped.tif]

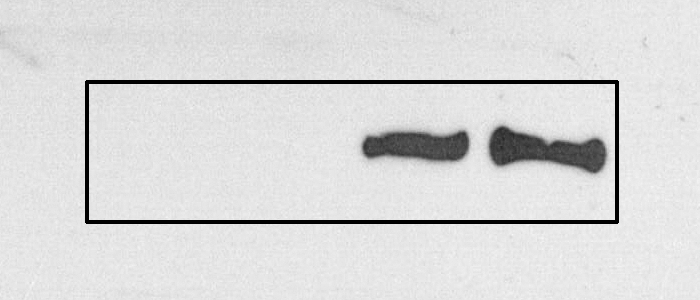

Supplement: Figure 1—figure supplement 1—source data 1. [file elife-86168-fig1-figsupp1-data1.zip › Figure 1-figure supplement 1 source data 1/annotated/figure supplement 1C cell expression LSD1-531AA mutant si LSD1 anti-Flag-LSD1-531aa uncropped.tif]

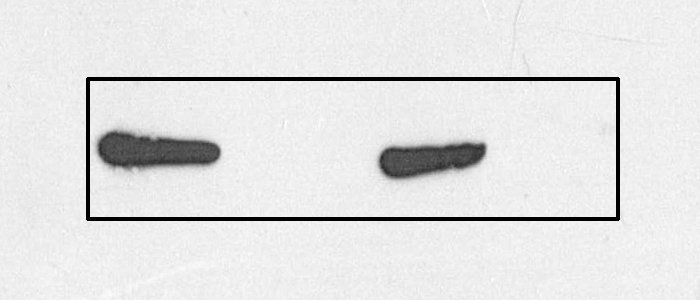

Supplement: Figure 1—figure supplement 1—source data 1. [file elife-86168-fig1-figsupp1-data1.zip › Figure 1-figure supplement 1 source data 1/annotated/figure supplement 1C cell expression LSD1-531AA mutant si LSD1 anti-EZH2 uncropped.tif]

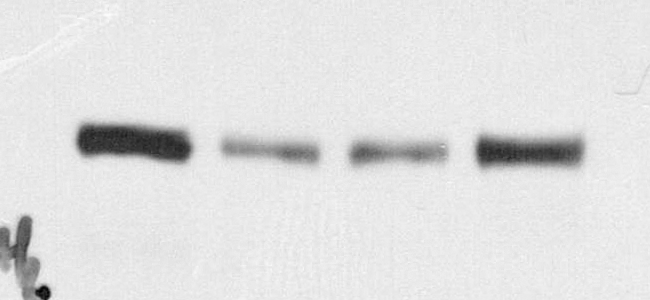

Supplement: Figure 2—source data 1. [file elife-86168-fig2-data1.zip › Figure 2 source data 1/Fig.2F Western anti-lsd1.tif]

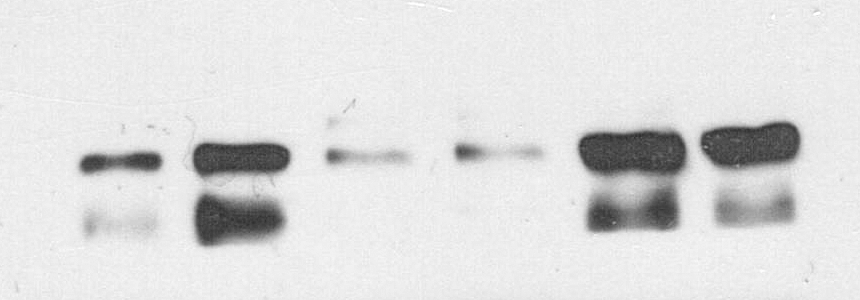

Supplement: Figure 2—source data 1. [file elife-86168-fig2-data1.zip › Figure 2 source data 1/Fig.2B 20220805 MEF WT L3-KO CHECK ezh2 anti-ezh2 uncropped.tif]

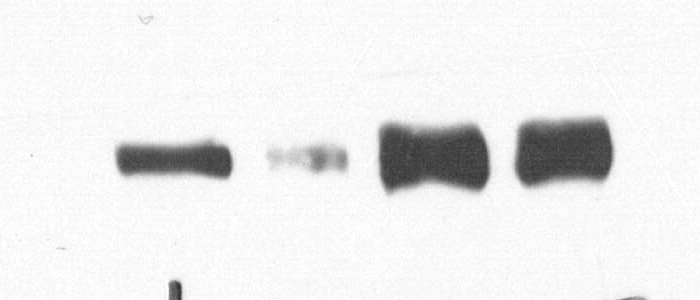

Supplement: Figure 2—source data 1. [file elife-86168-fig2-data1.zip › Figure 2 source data 1/Fig.2G 20220801 si lsd1 l3 #2 anti-flag-EZH2 uncropped.tif]

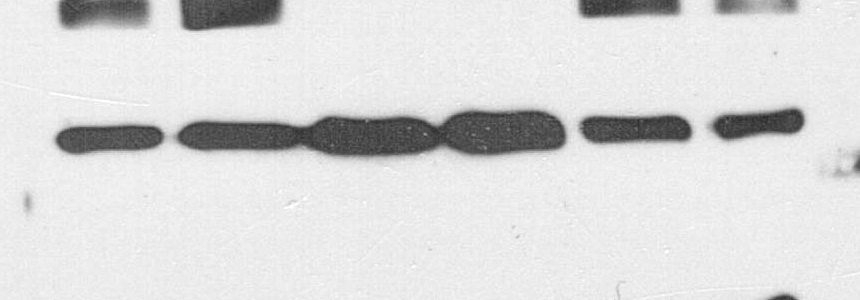

Supplement: Figure 2—source data 1. [file elife-86168-fig2-data1.zip › Figure 2 source data 1/Fig.2B 20220805 MEF WT L3-KO CHECK ezh2 anti-actin uncropped.tif]

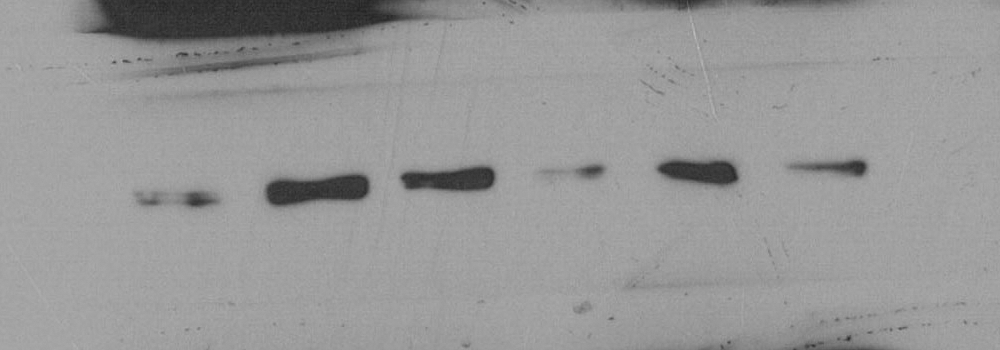

Supplement: Figure 2—source data 1. [file elife-86168-fig2-data1.zip › Figure 2 source data 1/Fig.2E 20220202 mef actin lsd1 l3flox anti-LSD1 uncropped.tif]

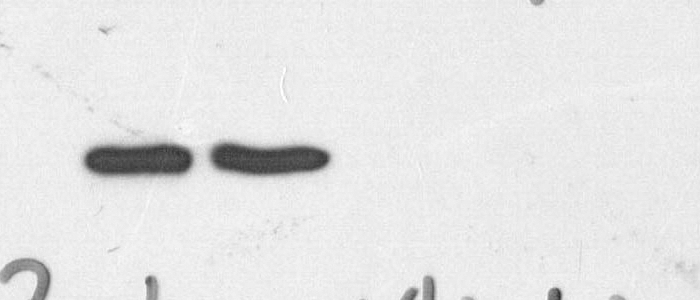

Supplement: Figure 2—source data 1. [file elife-86168-fig2-data1.zip › Figure 2 source data 1/Fig.2G 20220801 si lsd1 l3 #2 anti-L3MBTL3 uncropped.TIF]

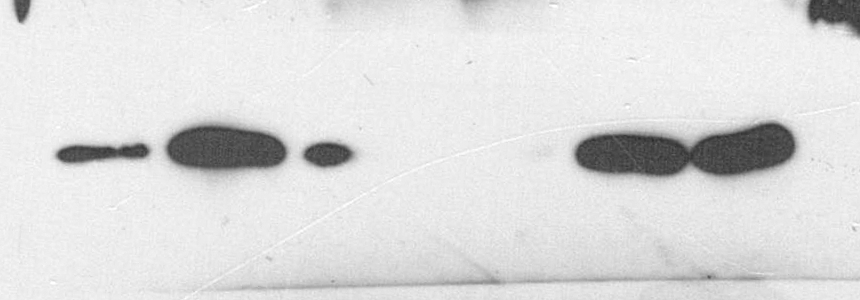

Supplement: Figure 2—source data 1. [file elife-86168-fig2-data1.zip › Figure 2 source data 1/Fig.2B 20220805 MEF WT L3-KO CHECK ezh2 anti-H3k27me3 uncropped.tif]

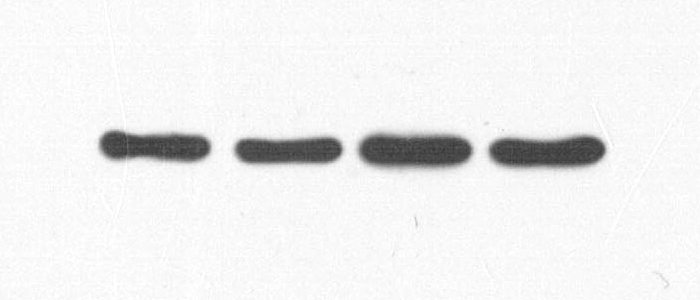

Supplement: Figure 2—source data 1. [file elife-86168-fig2-data1.zip › Figure 2 source data 1/Fig.2G 20220801 si lsd1 l3 #2 anti-flag-actin uncropped.tif]

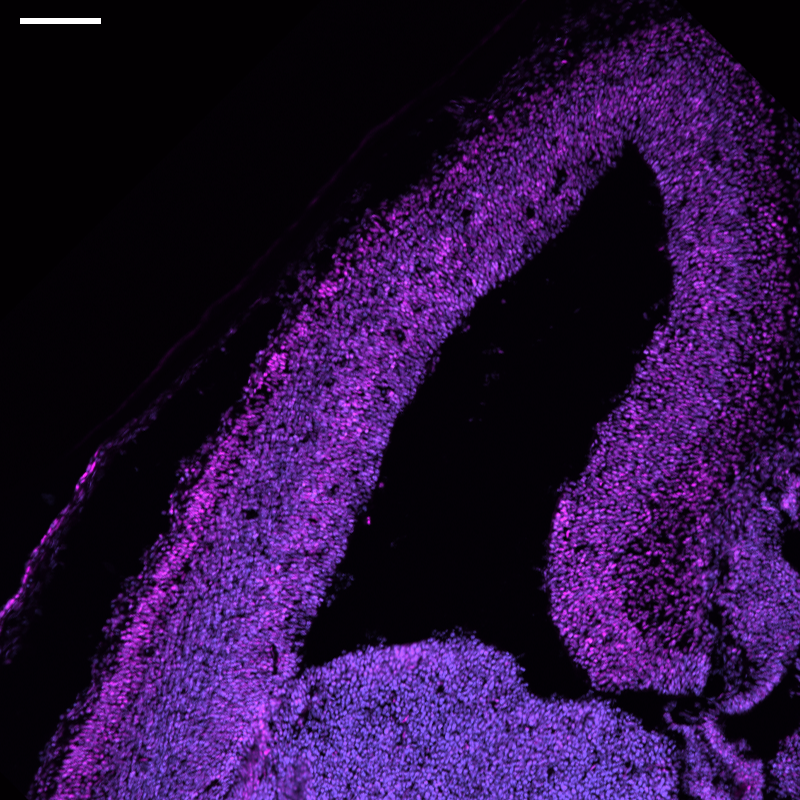

Supplement: Figure 2—source data 1. [file elife-86168-fig2-data1.zip › Figure 2 source data 1/Fig.2D 20211129 nestin-l3fl stain h3k27me3 ctrl -6.tif]

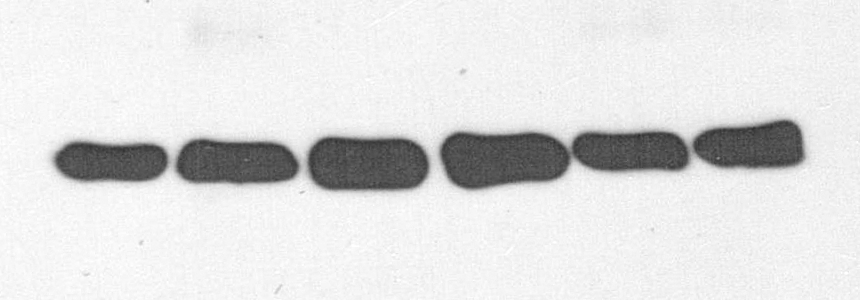

Supplement: Figure 2—source data 1. [file elife-86168-fig2-data1.zip › Figure 2 source data 1/Fig.2B 20220805 MEF WT L3-KO CHECK ezh2 anti-H3 uncropped.tif]

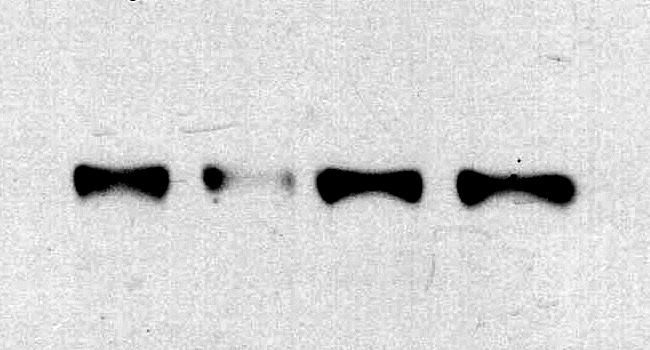

Supplement: Figure 2—source data 1. [file elife-86168-fig2-data1.zip › Figure 2 source data 1/Fig.2F Western anti-ezh2.tif]

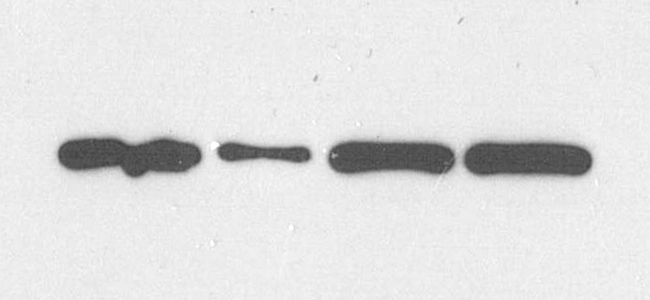

Supplement: Figure 2—source data 1. [file elife-86168-fig2-data1.zip › Figure 2 source data 1/Fig.2F Western anti-flag-EZH2.tif]

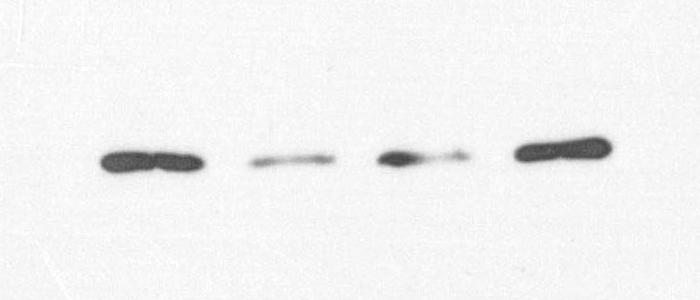

Supplement: Figure 2—source data 1. [file elife-86168-fig2-data1.zip › Figure 2 source data 1/Fig.2G 20220801 si lsd1 l3 #2 anti-LSD1 uncropped.TIF]

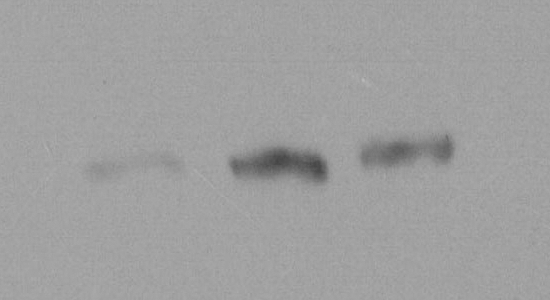

Supplement: Figure 2—source data 1. [file elife-86168-fig2-data1.zip › Figure 2 source data 1/Fig.2A Western anti-EZH2.tif]

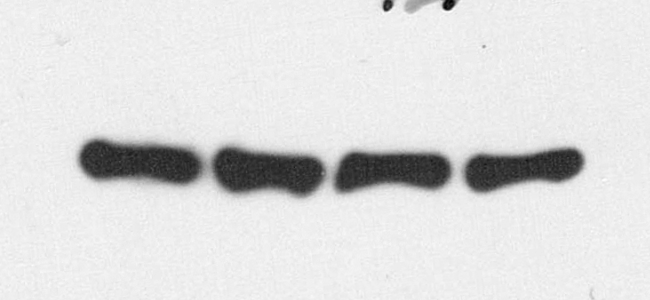

Supplement: Figure 2—source data 1. [file elife-86168-fig2-data1.zip › Figure 2 source data 1/Fig.2F Western anti-actin.tif]

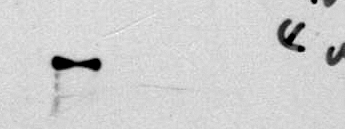

Supplement: Figure 2—source data 1. [file elife-86168-fig2-data1.zip › Figure 2 source data 1/Fig.2B MEF WT L3-KO CHECK ezh2 anti-L3MBTL3 uncropped.tif]

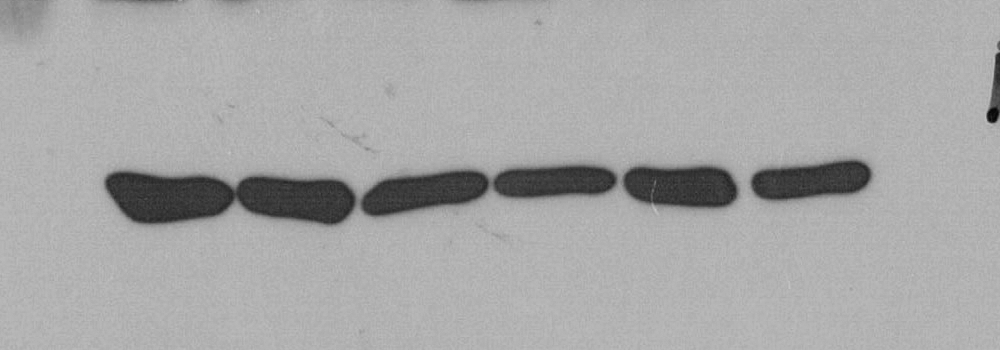

Supplement: Figure 2—source data 1. [file elife-86168-fig2-data1.zip › Figure 2 source data 1/Fig.2E 20220202 mef actin lsd1 l3flox anti-Actin uncropped.tif]

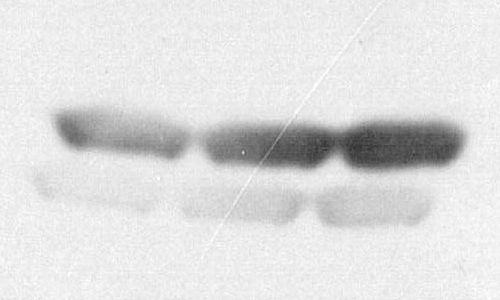

Supplement: Figure 2—source data 1. [file elife-86168-fig2-data1.zip › Figure 2 source data 1/Fig.2A Western anti-ACTIN.tif]

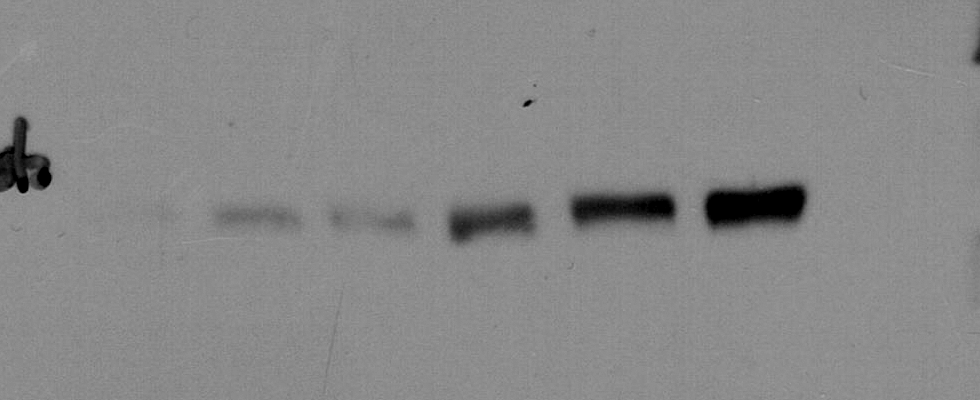

Supplement: Figure 2—source data 1. [file elife-86168-fig2-data1.zip › Figure 2 source data 1/Fig.2C 20211102 nestin-l3flox anti-ezh2.tif]

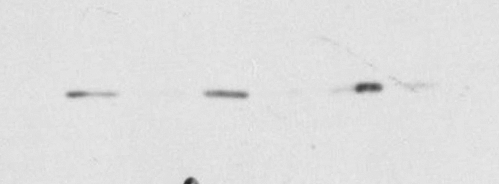

Supplement: Figure 2—source data 1. [file elife-86168-fig2-data1.zip › Figure 2 source data 1/Fig.2C 20211102 nestin-l3flox anti-L3MBTL3.tif]

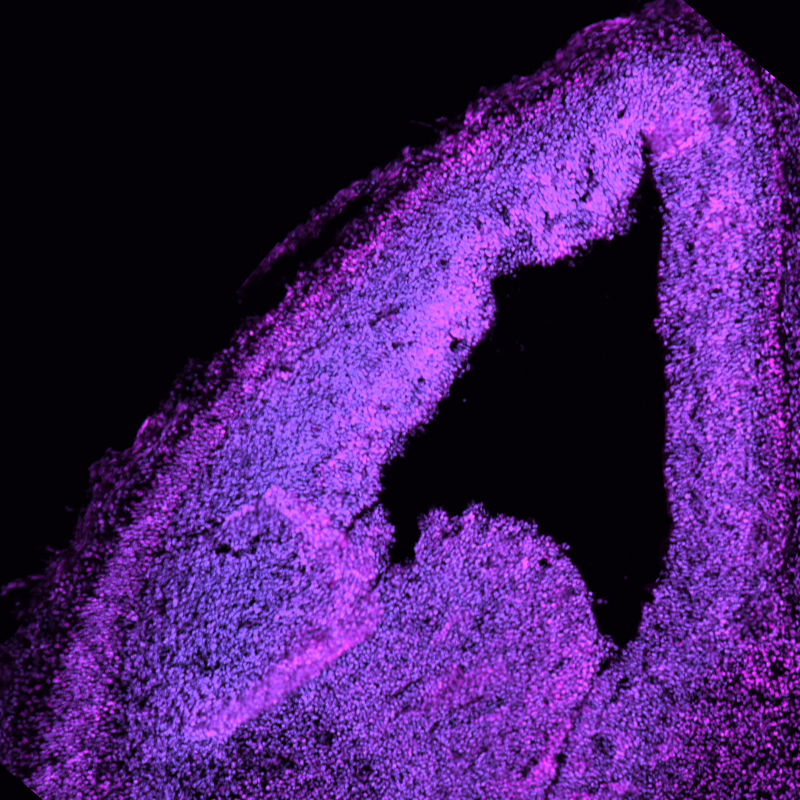

Supplement: Figure 2—source data 1. [file elife-86168-fig2-data1.zip › Figure 2 source data 1/Fig.2D 20211125 nestin-l3fl stain ezh2 flfl 1.tif]

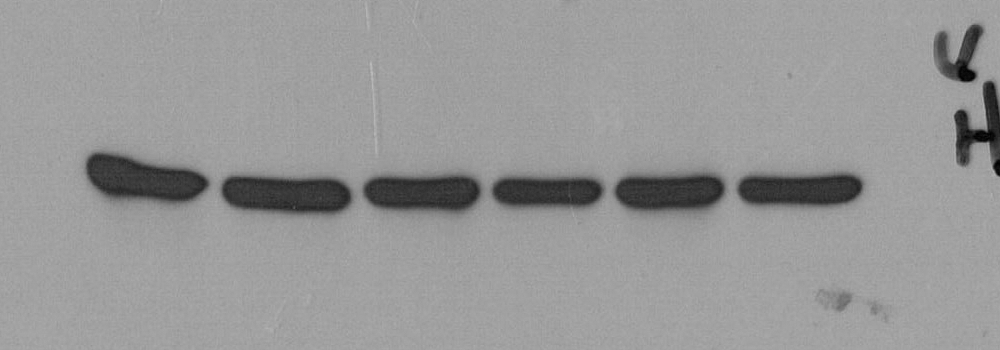

Supplement: Figure 2—source data 1. [file elife-86168-fig2-data1.zip › Figure 2 source data 1/Fig.2E 20220202 mef actin lsd1 l3flox anti-H3 upcropped.tif]

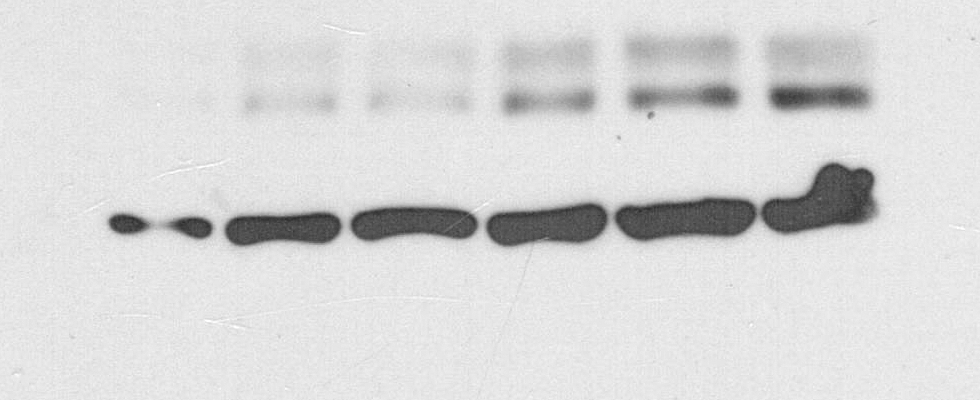

Supplement: Figure 2—source data 1. [file elife-86168-fig2-data1.zip › Figure 2 source data 1/Fig.2C 20211102 nestin-l3flox anti-actin.tif]

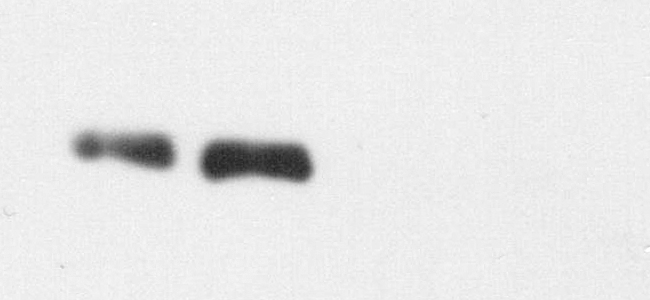

Supplement: Figure 2—source data 1. [file elife-86168-fig2-data1.zip › Figure 2 source data 1/Fig.2F Western anti-l3.tif]

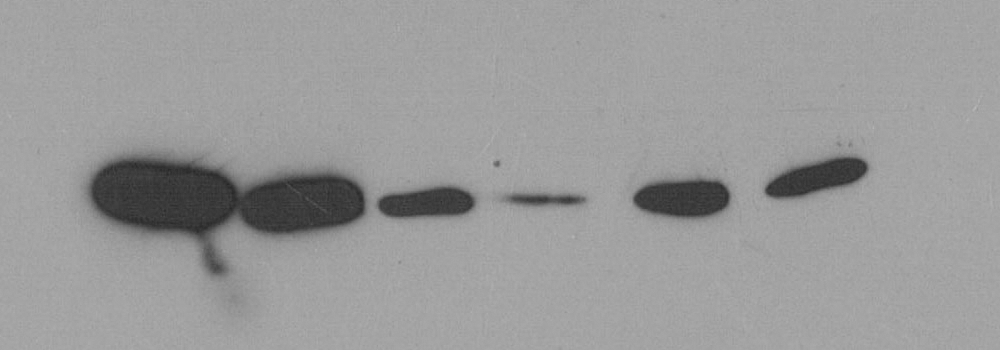

Supplement: Figure 2—source data 1. [file elife-86168-fig2-data1.zip › Figure 2 source data 1/Fig.2E 20220202 mef actin lsd1 l3flox anti-H3K27me3 uncropped.tif]

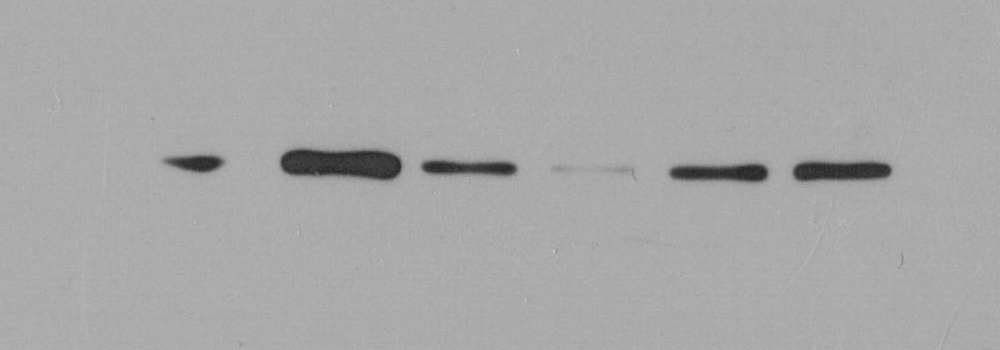

Supplement: Figure 2—source data 1. [file elife-86168-fig2-data1.zip › Figure 2 source data 1/Fig.2E 20220202 mef actin lsd1 l3flox anti-EZH2 uncropped.tif]
