# Supplementary material for: A methylation-phosphorylation switch controls EZH2 stability and hematopoiesis
Source: eLife. 2024 Feb 12;13:e86168. doi: 10.7554/eLife.86168 (PMC10901513; doi:10.7554/eLife.86168)

Figure1-figure supplement 1A-EZH2

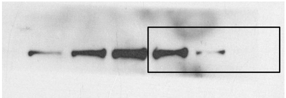

Figure1-figure supplement 1A-LSD1

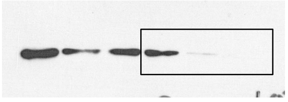

Figure1-figure supplement 1A-GAPDH

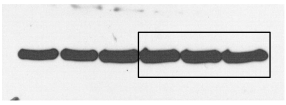

Figure1-figure supplement 1A-H3K27me3

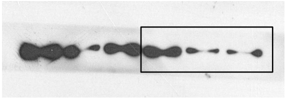

Figure1-figure supplement 1A-H3

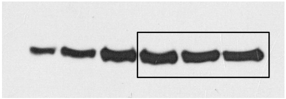

Figure 1-figure supplement 1B-EZH2

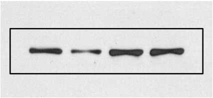

Figure 1-figure supplement 1B-Flag-LSD1

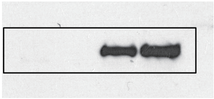

Figure 1-figure supplement 1B-Endogenous LSD1

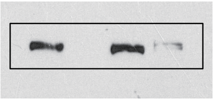

Figure 1-figure supplement 1B-GAPDH

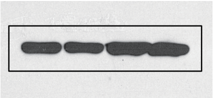

Figure 1-figure supplement 1C-EZH2

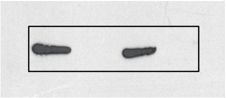

Figure 1-figure supplement 1C-Flag-LSD1-mutant

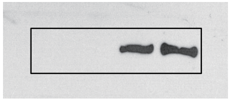

Figure 1-figure supplement 1C-Endogenous LSD1

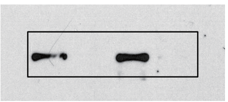

Figure 1-figure supplement 1C-GAPDH

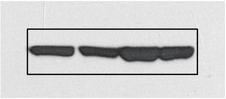

Supplement: Figure 1—figure supplement 1—source data 1. [file elife-86168-fig1-figsupp1-data1.zip › Figure 1-figure supplement 1 source data 1/Figure 1-figure supplement1 .pdf]
