# Supplementary material for: A methylation-phosphorylation switch controls EZH2 stability and hematopoiesis
Source: eLife. 2024 Feb 12;13:e86168. doi: 10.7554/eLife.86168 (PMC10901513; doi:10.7554/eLife.86168)

Figure 2A-EZH2

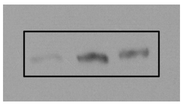

Figure 2A-SUZ12

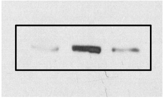

Figure 2A-L3MBTL3

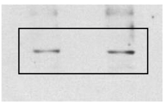

Figure 2A-Actin

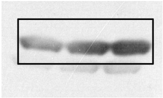

Figure 2B-EZH2

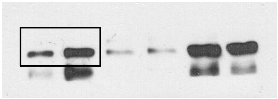

Figure 2B-H3

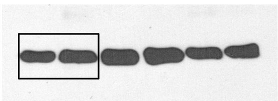

Figure 2B-Actin

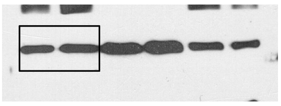

Figure 2B-H3K27me3

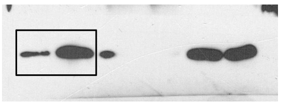

Figure 2B-L3MBTL3

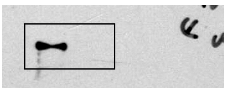

Figure 2C-EZH2

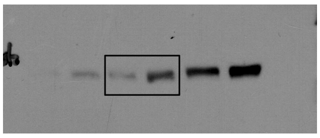

Figure 2C-SUZ12

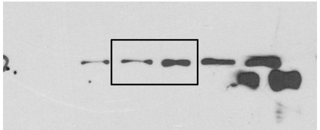

Figure 2C-L3MBTL3

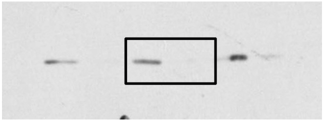

Figure 2C-Actin

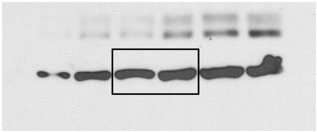

Figure 2E-EZH2

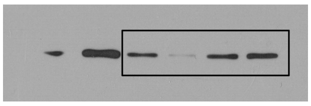

Figure 2E-H3K27me3

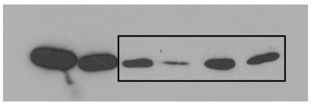

Figure 2E-Actin

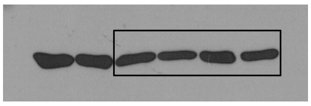

Figure 2E-LSD1

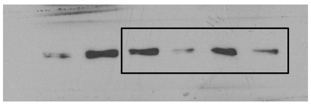

Figure 2E-H3

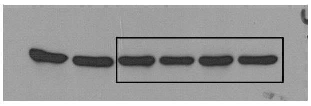

Figure 2F-EZH2

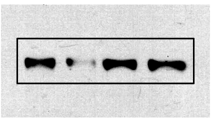

Figure 2F-L3MBTL3

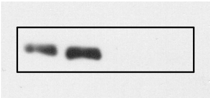

Figure 2F-Actin

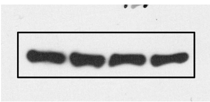

Figure 2F-LSD1

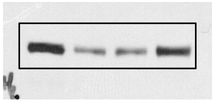

Figure 2F-Flag-EZH2

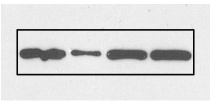

Figure 2G-L3MBTL3

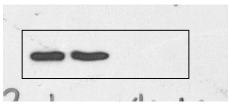

Figure 2G-Actin

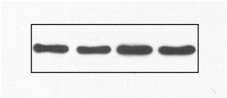

Figure 2G-LSD1

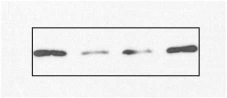

Figure 2G-Flag-EZH2

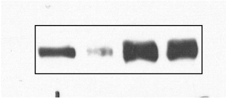

Supplement: Figure 2—source data 1. [file elife-86168-fig2-data1.zip › Figure 2 source data 1/Figure 2-annotated source data.pdf]
