# Supplementary figures and images for: A methylation-phosphorylation switch controls EZH2 stability and hematopoiesis (part 2 of 7)
Source: eLife. 2024 Feb 12;13:e86168. doi: 10.7554/eLife.86168 (PMC10901513; doi:10.7554/eLife.86168)

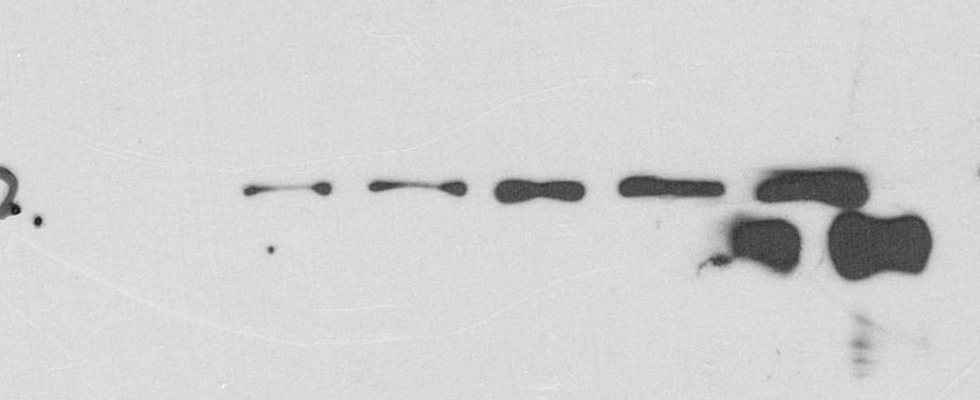

Supplement: Figure 2—source data 1. [file elife-86168-fig2-data1.zip › Figure 2 source data 1/Fig.2C 20211102 nestin-l3flox anti-suz12.tif]

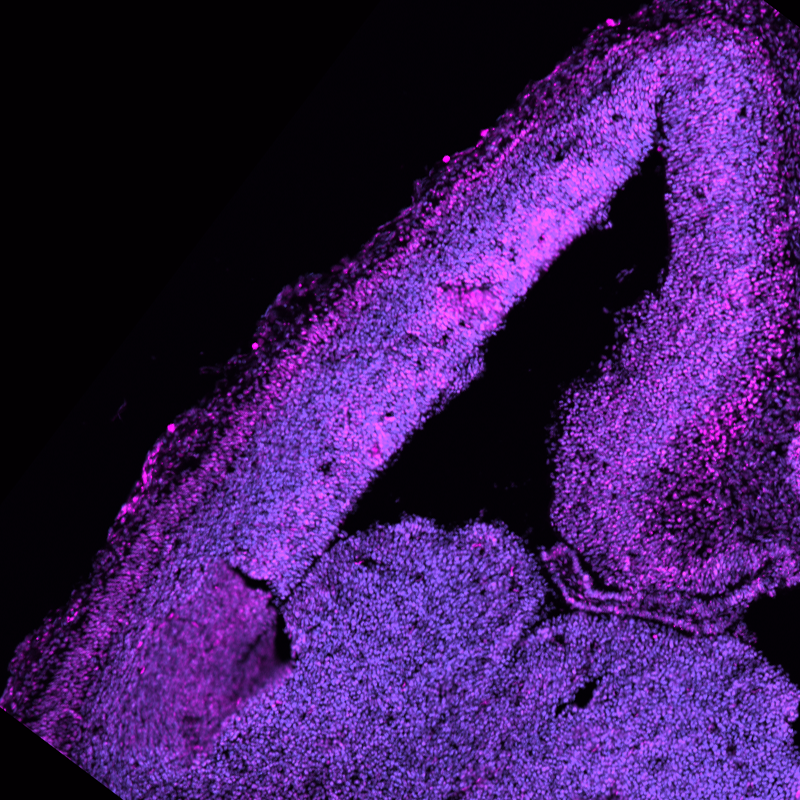

Supplement: Figure 2—source data 1. [file elife-86168-fig2-data1.zip › Figure 2 source data 1/Fig.2D 20211129 nestin-l3fl stain h3k27me3 flfl 6.tif]

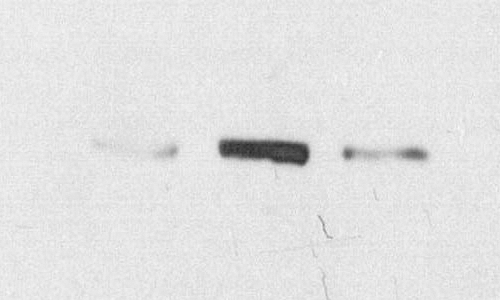

Supplement: Figure 2—source data 1. [file elife-86168-fig2-data1.zip › Figure 2 source data 1/Fig.2A Western anti-SUZ12.tif]

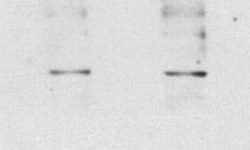

Supplement: Figure 2—source data 1. [file elife-86168-fig2-data1.zip › Figure 2 source data 1/Fig.2A Western anti-L3MBTL3.tif]

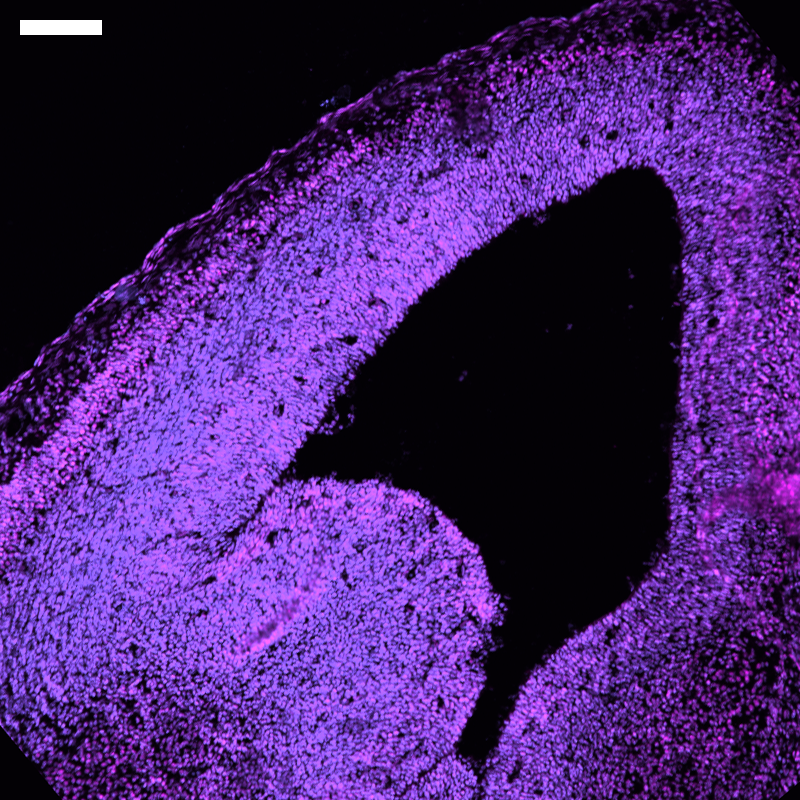

Supplement: Figure 2—source data 1. [file elife-86168-fig2-data1.zip › Figure 2 source data 1/Fig.2D 20211125 nestin-l3fl stain ezh2 ctrl 3.tif]

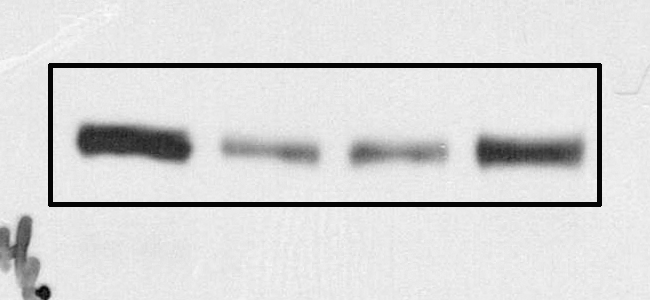

Supplement: Figure 2—source data 1. [file elife-86168-fig2-data1.zip › Figure 2 source data 1/annotated/Fig.2F Western anti-lsd1.tif]

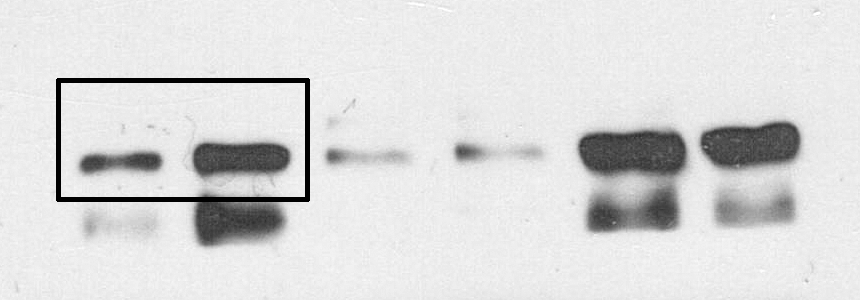

Supplement: Figure 2—source data 1. [file elife-86168-fig2-data1.zip › Figure 2 source data 1/annotated/Fig.2B 20220805 MEF WT L3-KO CHECK ezh2 anti-ezh2 uncropped.tif]

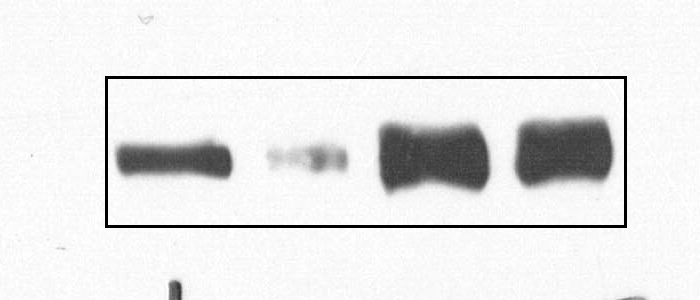

Supplement: Figure 2—source data 1. [file elife-86168-fig2-data1.zip › Figure 2 source data 1/annotated/Fig.2G 20220801 si lsd1 l3 #2 anti-flag-EZH2 uncropped.tif]

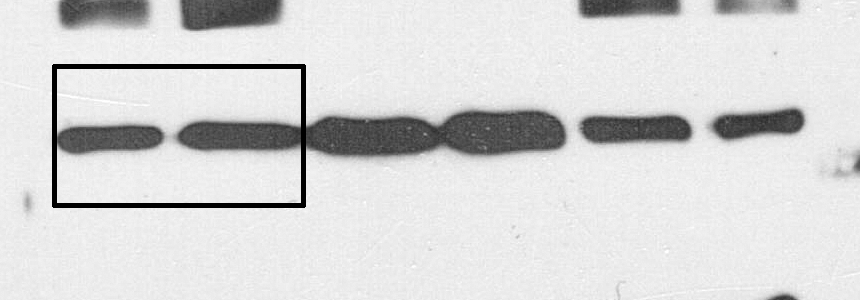

Supplement: Figure 2—source data 1. [file elife-86168-fig2-data1.zip › Figure 2 source data 1/annotated/Fig.2B 20220805 MEF WT L3-KO CHECK ezh2 anti-actin uncropped.tif]

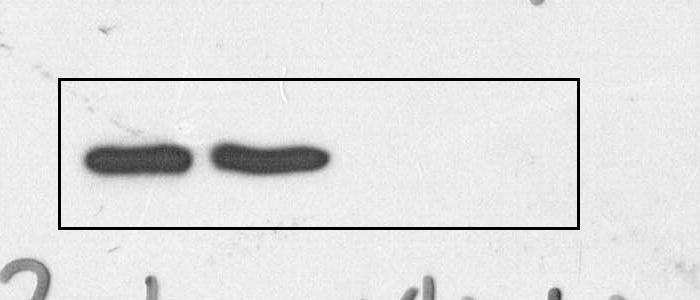

Supplement: Figure 2—source data 1. [file elife-86168-fig2-data1.zip › Figure 2 source data 1/annotated/Fig.2G 20220801 si lsd1 l3 #2 anti-L3MBTL3 uncropped.TIF]

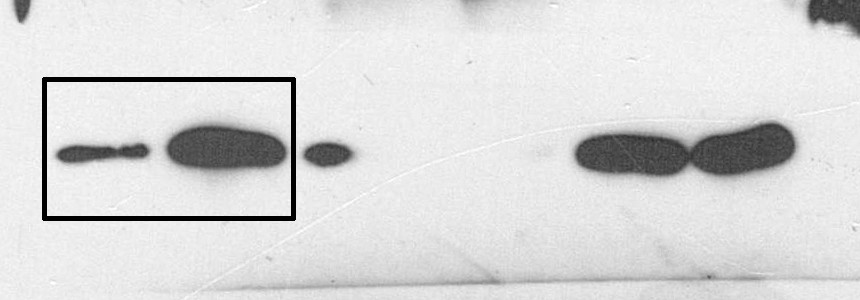

Supplement: Figure 2—source data 1. [file elife-86168-fig2-data1.zip › Figure 2 source data 1/annotated/Fig.2B 20220805 MEF WT L3-KO CHECK ezh2 anti-H3k27me3 uncropped.tif]

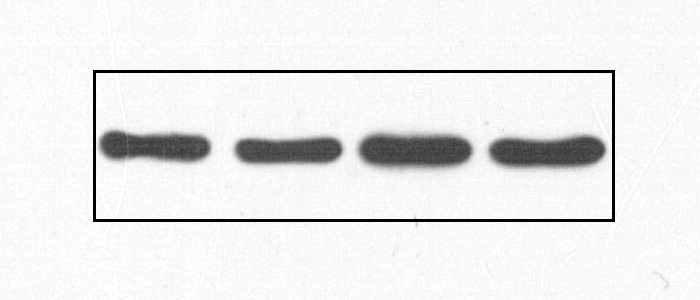

Supplement: Figure 2—source data 1. [file elife-86168-fig2-data1.zip › Figure 2 source data 1/annotated/Fig.2G 20220801 si lsd1 l3 #2 anti-flag-actin uncropped.tif]

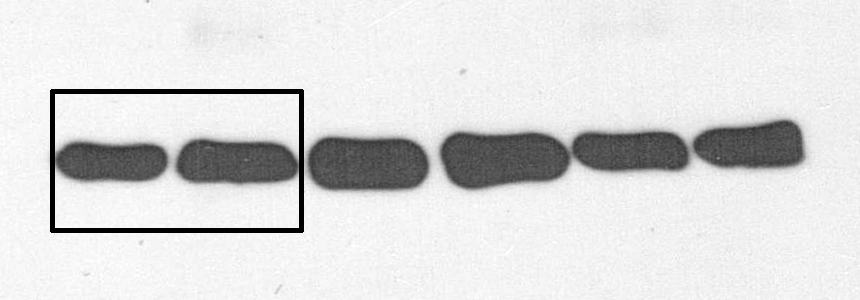

Supplement: Figure 2—source data 1. [file elife-86168-fig2-data1.zip › Figure 2 source data 1/annotated/Fig.2B 20220805 MEF WT L3-KO CHECK ezh2 anti-H3 uncropped.tif]

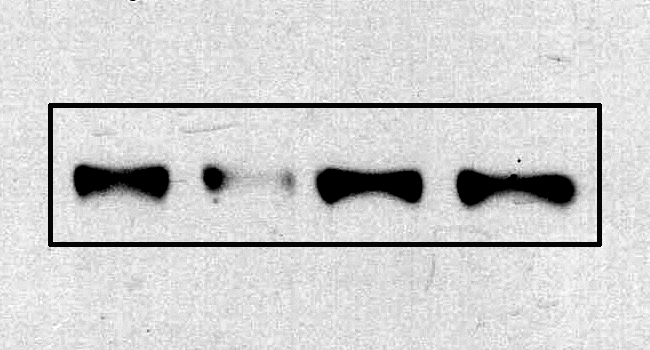

Supplement: Figure 2—source data 1. [file elife-86168-fig2-data1.zip › Figure 2 source data 1/annotated/Fig.2F Western anti-ezh2.tif]

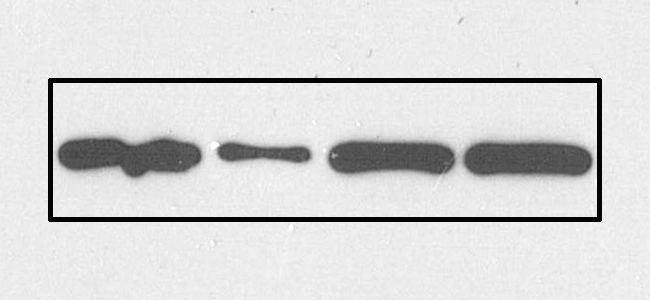

Supplement: Figure 2—source data 1. [file elife-86168-fig2-data1.zip › Figure 2 source data 1/annotated/Fig.2F Western anti-flag-EZH2.tif]

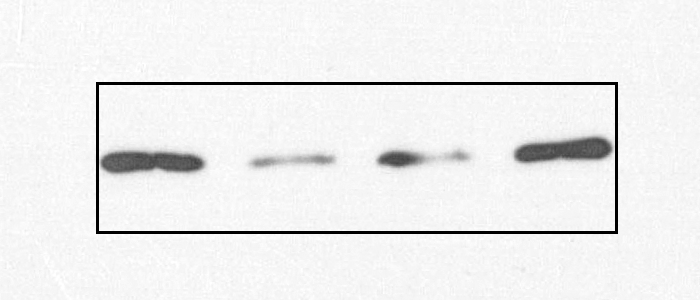

Supplement: Figure 2—source data 1. [file elife-86168-fig2-data1.zip › Figure 2 source data 1/annotated/Fig.2G 20220801 si lsd1 l3 #2 anti-LSD1 uncropped.TIF]

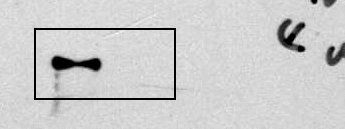

Supplement: Figure 2—source data 1. [file elife-86168-fig2-data1.zip › Figure 2 source data 1/annotated/Fig.2B MEF WT L3-KO CHECK ezh2 anti-L3MBTL3 uncropped.tif]

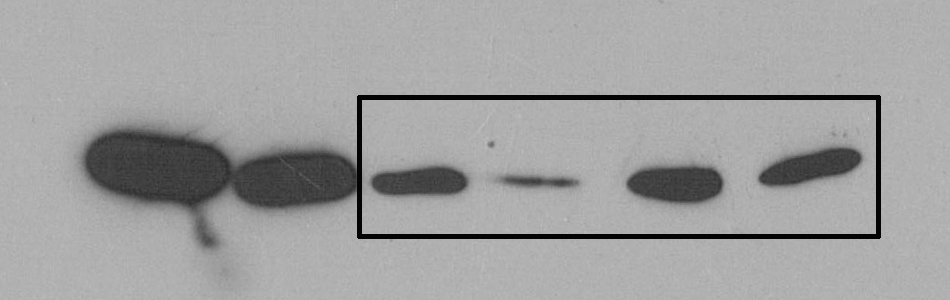

Supplement: Figure 2—source data 1. [file elife-86168-fig2-data1.zip › Figure 2 source data 1/annotated/Fig.2E 20220202 mef actin lsd1 l3flox anti-H3K27me3 uncropped.tif]

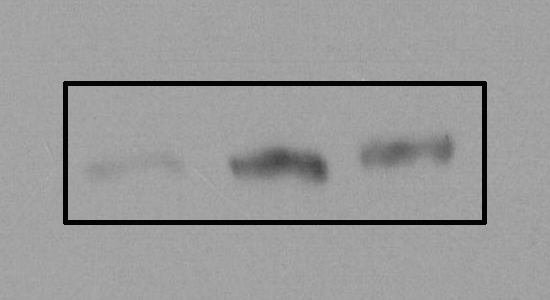

Supplement: Figure 2—source data 1. [file elife-86168-fig2-data1.zip › Figure 2 source data 1/annotated/Fig.2A Western anti-EZH2.tif]

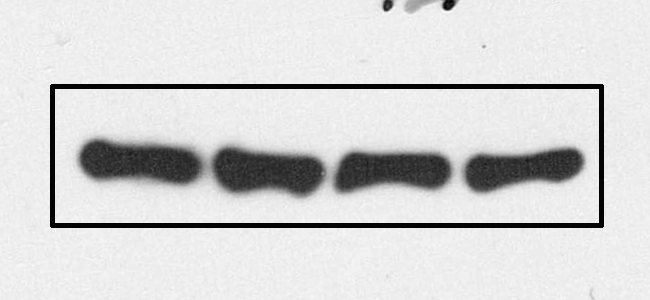

Supplement: Figure 2—source data 1. [file elife-86168-fig2-data1.zip › Figure 2 source data 1/annotated/Fig.2F Western anti-actin.tif]

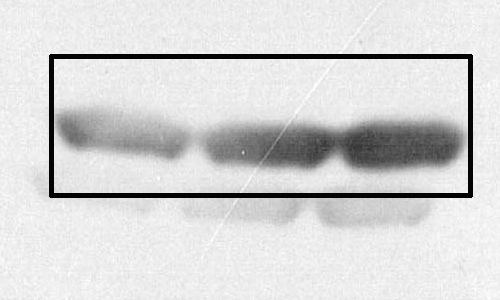

Supplement: Figure 2—source data 1. [file elife-86168-fig2-data1.zip › Figure 2 source data 1/annotated/Fig.2A Western anti-ACTIN.tif]

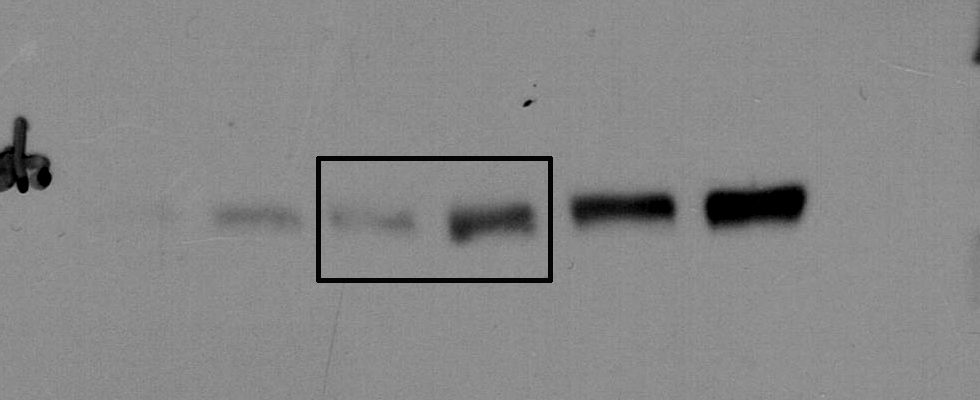

Supplement: Figure 2—source data 1. [file elife-86168-fig2-data1.zip › Figure 2 source data 1/annotated/Fig.2C 20211102 nestin-l3flox anti-ezh2.tif]

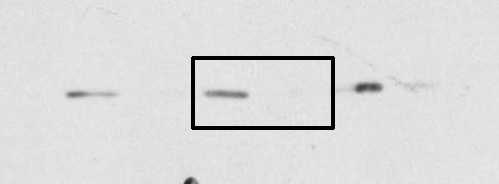

Supplement: Figure 2—source data 1. [file elife-86168-fig2-data1.zip › Figure 2 source data 1/annotated/Fig.2C 20211102 nestin-l3flox anti-L3MBTL3.tif]

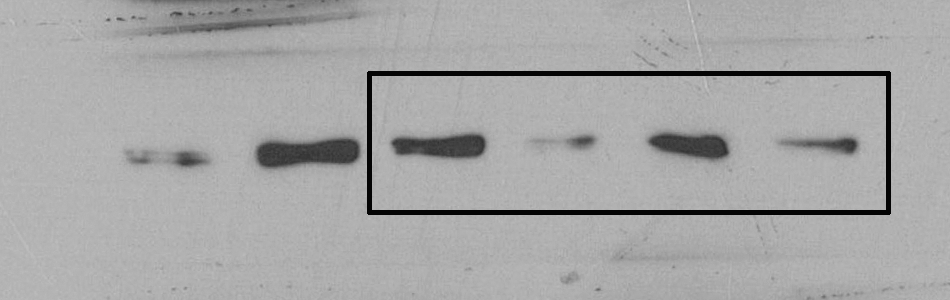

Supplement: Figure 2—source data 1. [file elife-86168-fig2-data1.zip › Figure 2 source data 1/annotated/Fig.2E 20220202 mef actin lsd1 l3flox anti-LSD1 uncropped.tif]

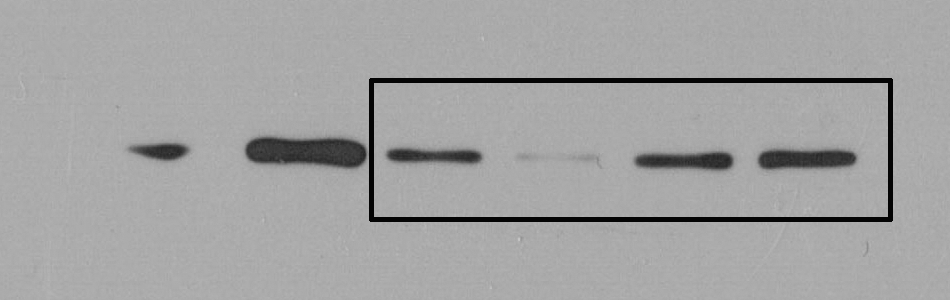

Supplement: Figure 2—source data 1. [file elife-86168-fig2-data1.zip › Figure 2 source data 1/annotated/Fig.2E 20220202 mef actin lsd1 l3flox anti-EZH2 uncropped.tif]

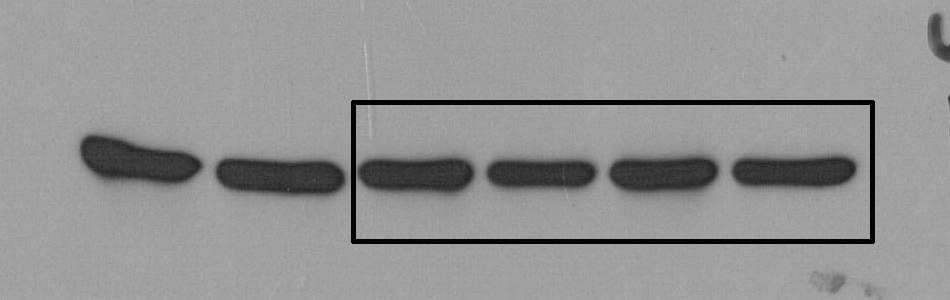

Supplement: Figure 2—source data 1. [file elife-86168-fig2-data1.zip › Figure 2 source data 1/annotated/Fig.2E 20220202 mef actin lsd1 l3flox anti-H3 upcropped.tif]

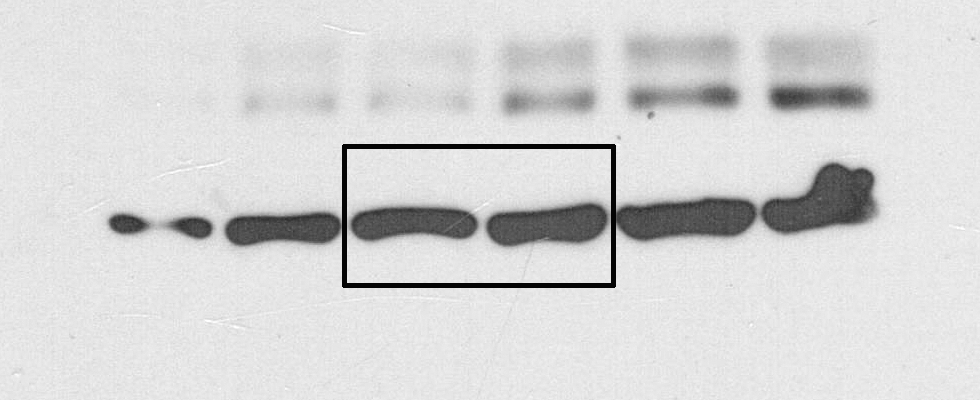

Supplement: Figure 2—source data 1. [file elife-86168-fig2-data1.zip › Figure 2 source data 1/annotated/Fig.2C 20211102 nestin-l3flox anti-actin.tif]

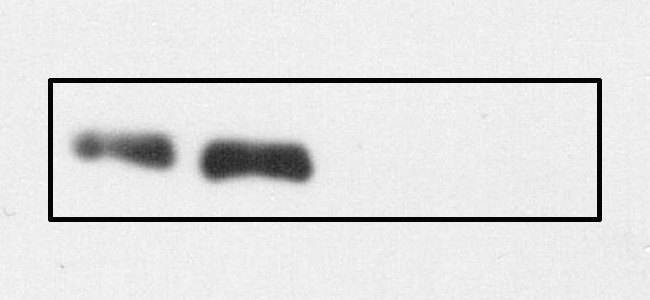

Supplement: Figure 2—source data 1. [file elife-86168-fig2-data1.zip › Figure 2 source data 1/annotated/Fig.2F Western anti-l3.tif]

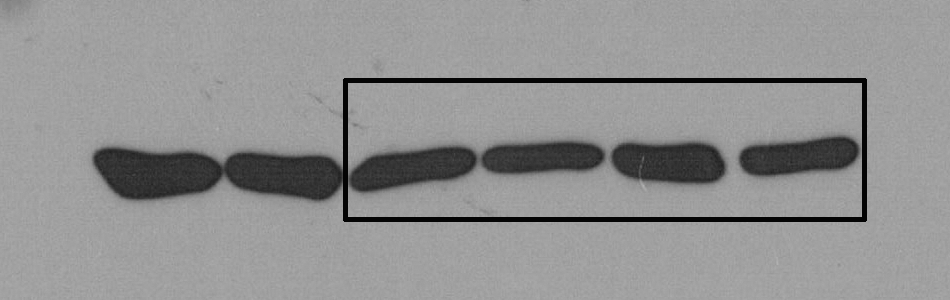

Supplement: Figure 2—source data 1. [file elife-86168-fig2-data1.zip › Figure 2 source data 1/annotated/Fig.2E 20220202 mef actin lsd1 l3flox anti-Actin uncropped.tif]

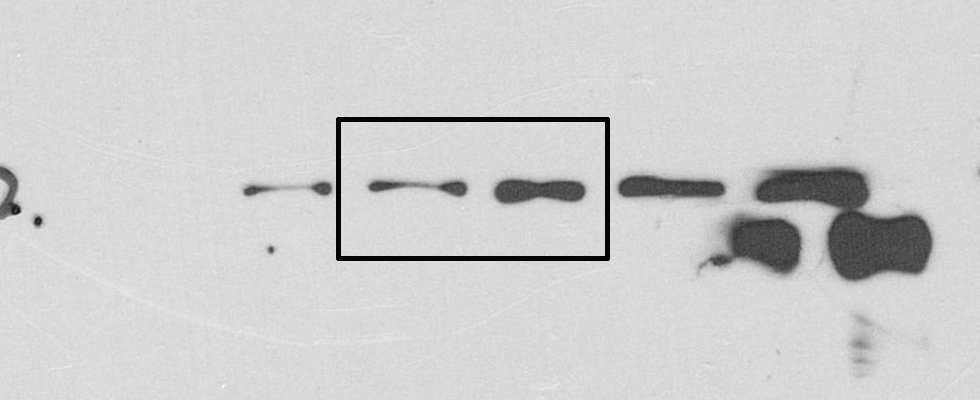

Supplement: Figure 2—source data 1. [file elife-86168-fig2-data1.zip › Figure 2 source data 1/annotated/Fig.2C 20211102 nestin-l3flox anti-suz12.tif]

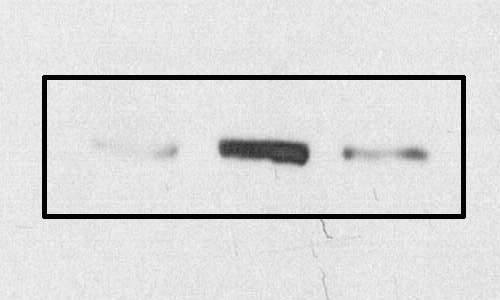

Supplement: Figure 2—source data 1. [file elife-86168-fig2-data1.zip › Figure 2 source data 1/annotated/Fig.2A Western anti-SUZ12.tif]

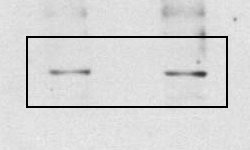

Supplement: Figure 2—source data 1. [file elife-86168-fig2-data1.zip › Figure 2 source data 1/annotated/Fig.2A Western anti-L3MBTL3.tif]

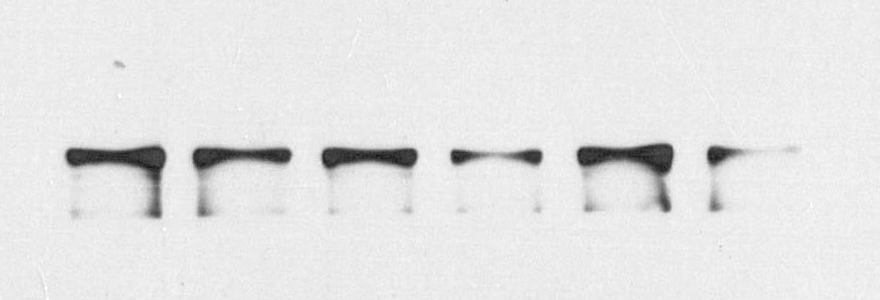

Supplement: Figure 2—figure supplement 2—source data 1. [file elife-86168-fig2-figsupp2-data1.zip › Figure 2-figure supplement2 source data 1/Figure2-figure supplement 2B HCT116 WT L3-stable cell lines anti-EZH2 uncropped.tif]

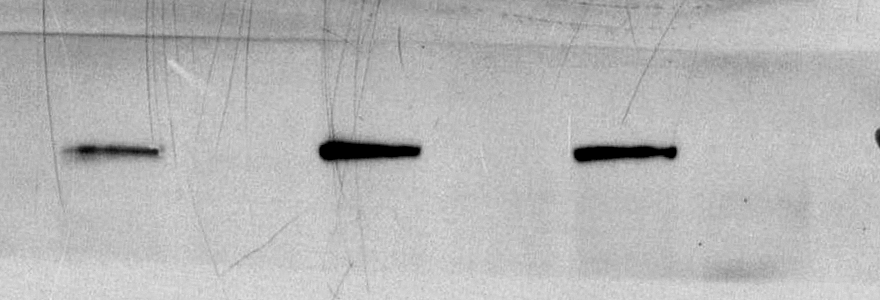

Supplement: Figure 2—figure supplement 2—source data 1. [file elife-86168-fig2-figsupp2-data1.zip › Figure 2-figure supplement2 source data 1/Figure2-figure supplement 2A hct116 wt l3-ko check EZH2 Anti-l3mbtl3 rep.1 uncropped.tif]

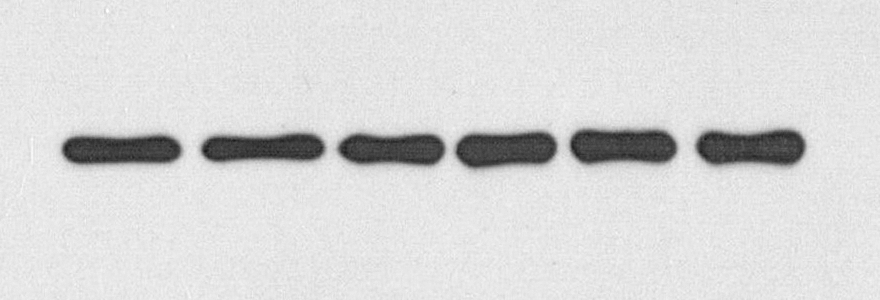

Supplement: Figure 2—figure supplement 2—source data 1. [file elife-86168-fig2-figsupp2-data1.zip › Figure 2-figure supplement2 source data 1/Figure2-figure supplement 2B HCT116 WT L3-stable cell lines anti-GAPDH uncropped.tif]

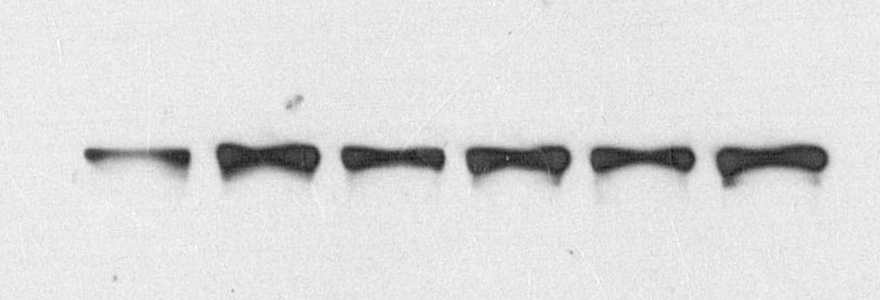

Supplement: Figure 2—figure supplement 2—source data 1. [file elife-86168-fig2-figsupp2-data1.zip › Figure 2-figure supplement2 source data 1/Figure2-figure supplement 2A hct116 wt l3-ko check EZH2 Anti-EZH2 rep.1 uncropped.tif]

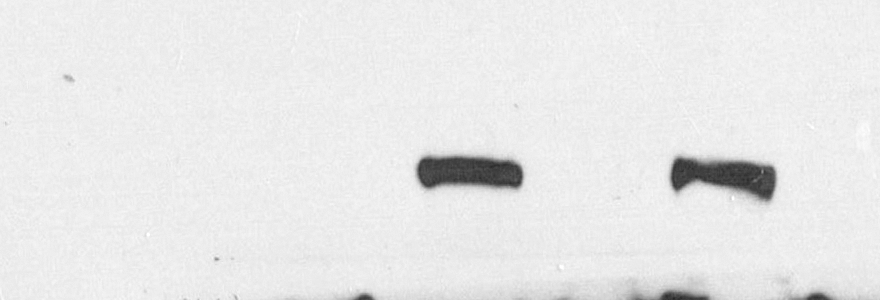

Supplement: Figure 2—figure supplement 2—source data 1. [file elife-86168-fig2-figsupp2-data1.zip › Figure 2-figure supplement2 source data 1/Figure2-figure supplement 2B HCT116 WT L3-stable cell lines anti-flag-l3mbtl3 uncropped.tif]

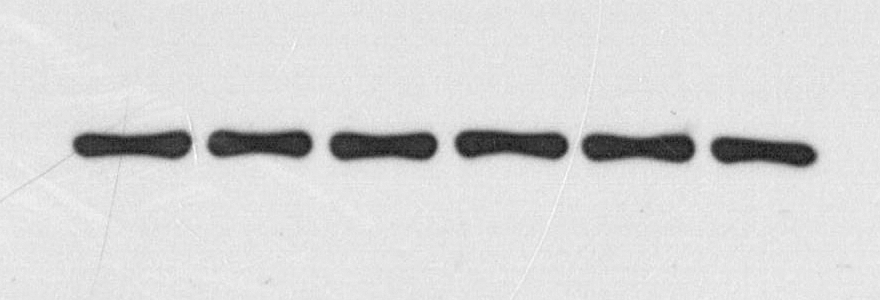

Supplement: Figure 2—figure supplement 2—source data 1. [file elife-86168-fig2-figsupp2-data1.zip › Figure 2-figure supplement2 source data 1/Figure2-figure supplement 2A hct116 wt l3-ko check EZH2 Anti-actin rep.1 uncropped.tif]

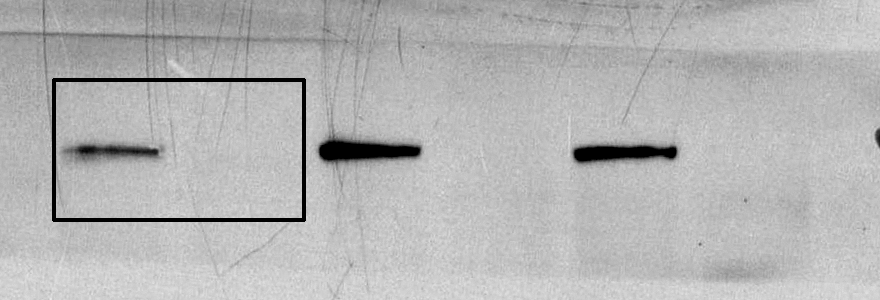

Supplement: Figure 2—figure supplement 2—source data 1. [file elife-86168-fig2-figsupp2-data1.zip › Figure 2-figure supplement2 source data 1/annotated/Figure2-figure supplement 2A hct116 wt l3-ko check EZH2 Anti-l3mbtl3 rep.1 uncropped.tif]

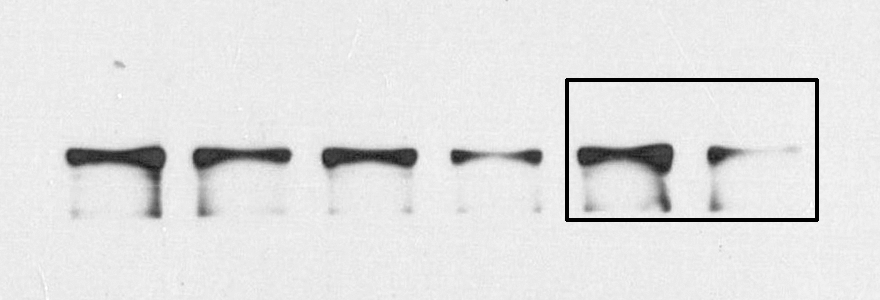

Supplement: Figure 2—figure supplement 2—source data 1. [file elife-86168-fig2-figsupp2-data1.zip › Figure 2-figure supplement2 source data 1/annotated/Figure2-figure supplement 2B HCT116 WT L3-stable cell lines anti-EZH2 uncropped.tif]

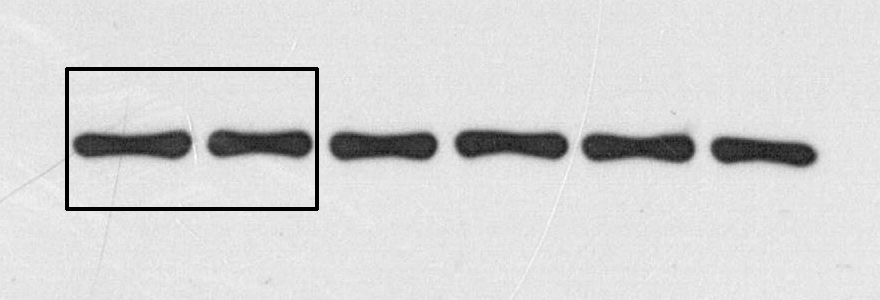

Supplement: Figure 2—figure supplement 2—source data 1. [file elife-86168-fig2-figsupp2-data1.zip › Figure 2-figure supplement2 source data 1/annotated/Figure2-figure supplement 2A hct116 wt l3-ko check EZH2 Anti-actin rep.1 uncropped.tif]

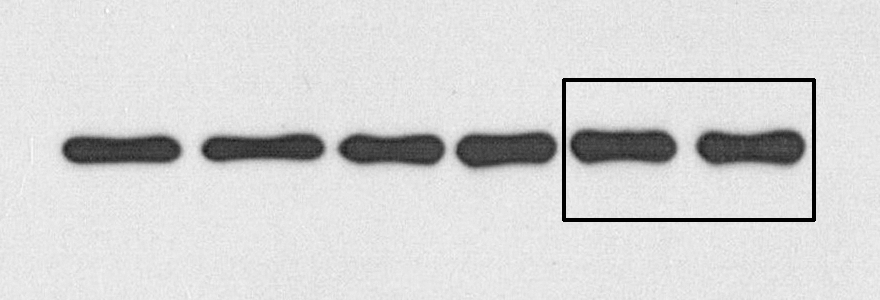

Supplement: Figure 2—figure supplement 2—source data 1. [file elife-86168-fig2-figsupp2-data1.zip › Figure 2-figure supplement2 source data 1/annotated/Figure2-figure supplement 2B HCT116 WT L3-stable cell lines anti-GAPDH uncropped.tif]

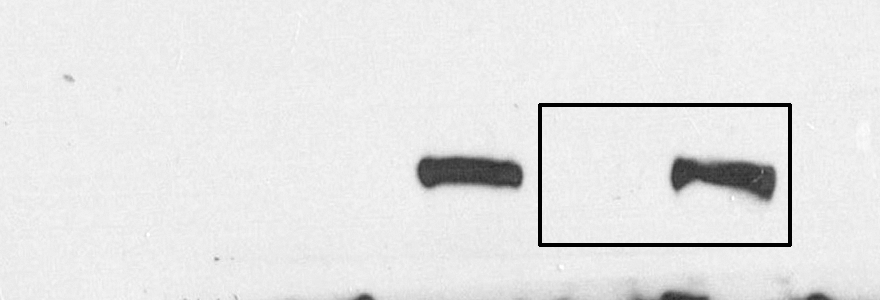

Supplement: Figure 2—figure supplement 2—source data 1. [file elife-86168-fig2-figsupp2-data1.zip › Figure 2-figure supplement2 source data 1/annotated/Figure2-figure supplement 2B HCT116 WT L3-stable cell lines anti-flag-l3mbtl3 uncropped.tif]

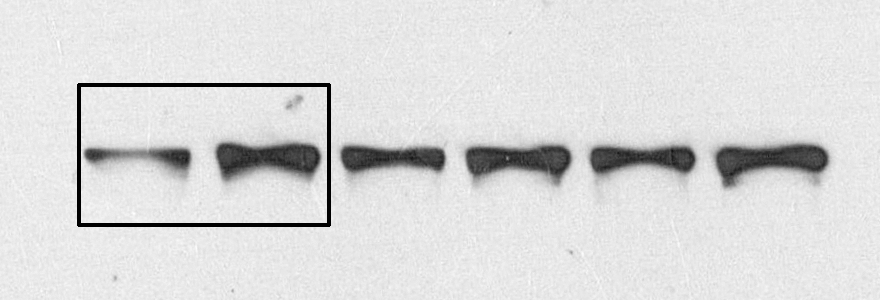

Supplement: Figure 2—figure supplement 2—source data 1. [file elife-86168-fig2-figsupp2-data1.zip › Figure 2-figure supplement2 source data 1/annotated/Figure2-figure supplement 2A hct116 wt l3-ko check EZH2 Anti-EZH2 rep.1 uncropped.tif]

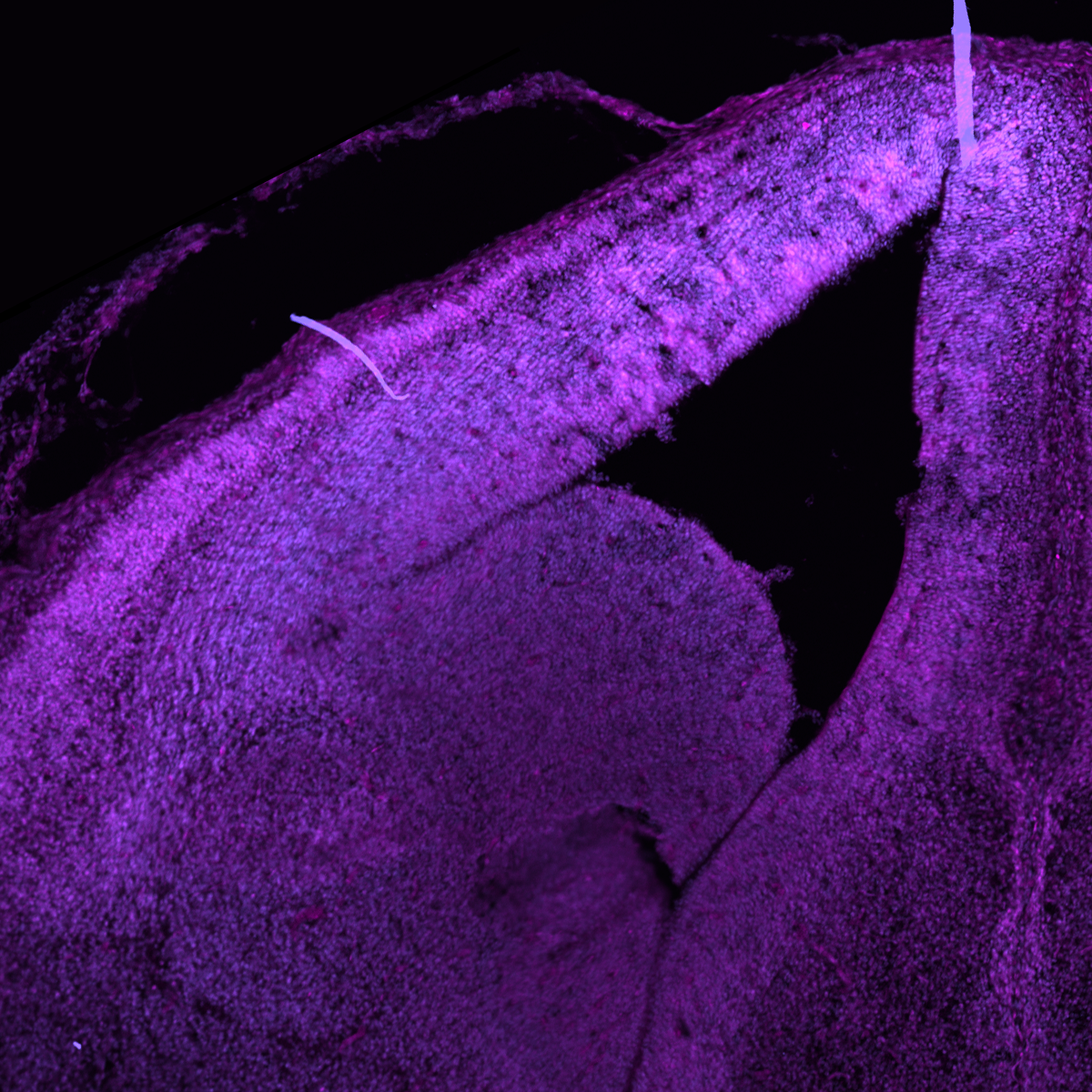

Supplement: Figure 3—source data 1. [file elife-86168-fig3-data1.zip › Figure 3 source data 1/Fig.3D 20220429 dcaf5 e14.4 wt anti h3k27me3 merge cut.tif]

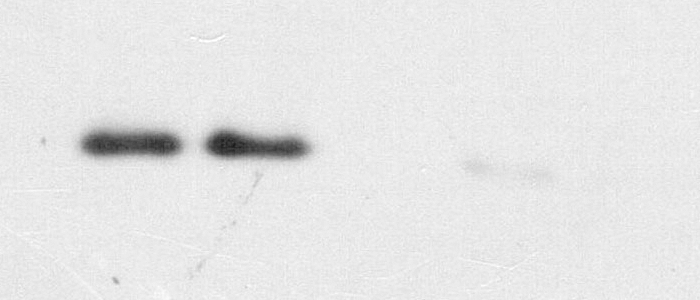

Supplement: Figure 3—source data 1. [file elife-86168-fig3-data1.zip › Figure 3 source data 1/Fig.3F 20220801 si lsd1 d5 #2 anti-d5 rep3 uncropped.tif]

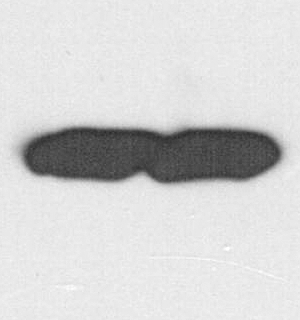

Supplement: Figure 3—source data 1. [file elife-86168-fig3-data1.zip › Figure 3 source data 1/Fig.3C 20220505 dcaf5 e14.5 head check ezh2 anti-H3 uncropped.tif]

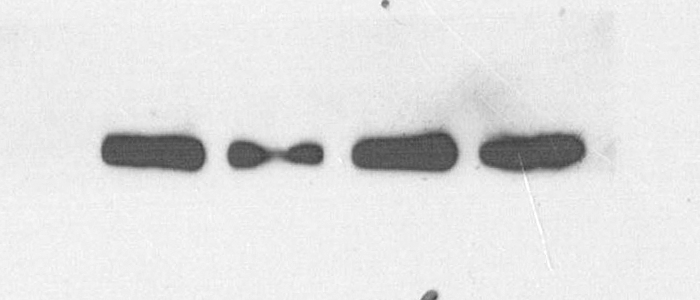

Supplement: Figure 3—source data 1. [file elife-86168-fig3-data1.zip › Figure 3 source data 1/Fig.3F 20220801 si lsd1 d5 #2 anti-flag-EZH2 uncropped.tif]

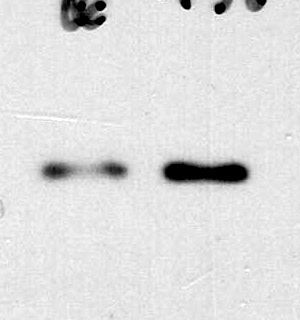

Supplement: Figure 3—source data 1. [file elife-86168-fig3-data1.zip › Figure 3 source data 1/Fig.3C 20220505 dcaf5 e14.5 head check ezh2 anti-h3k27me3 uncropped.tif]

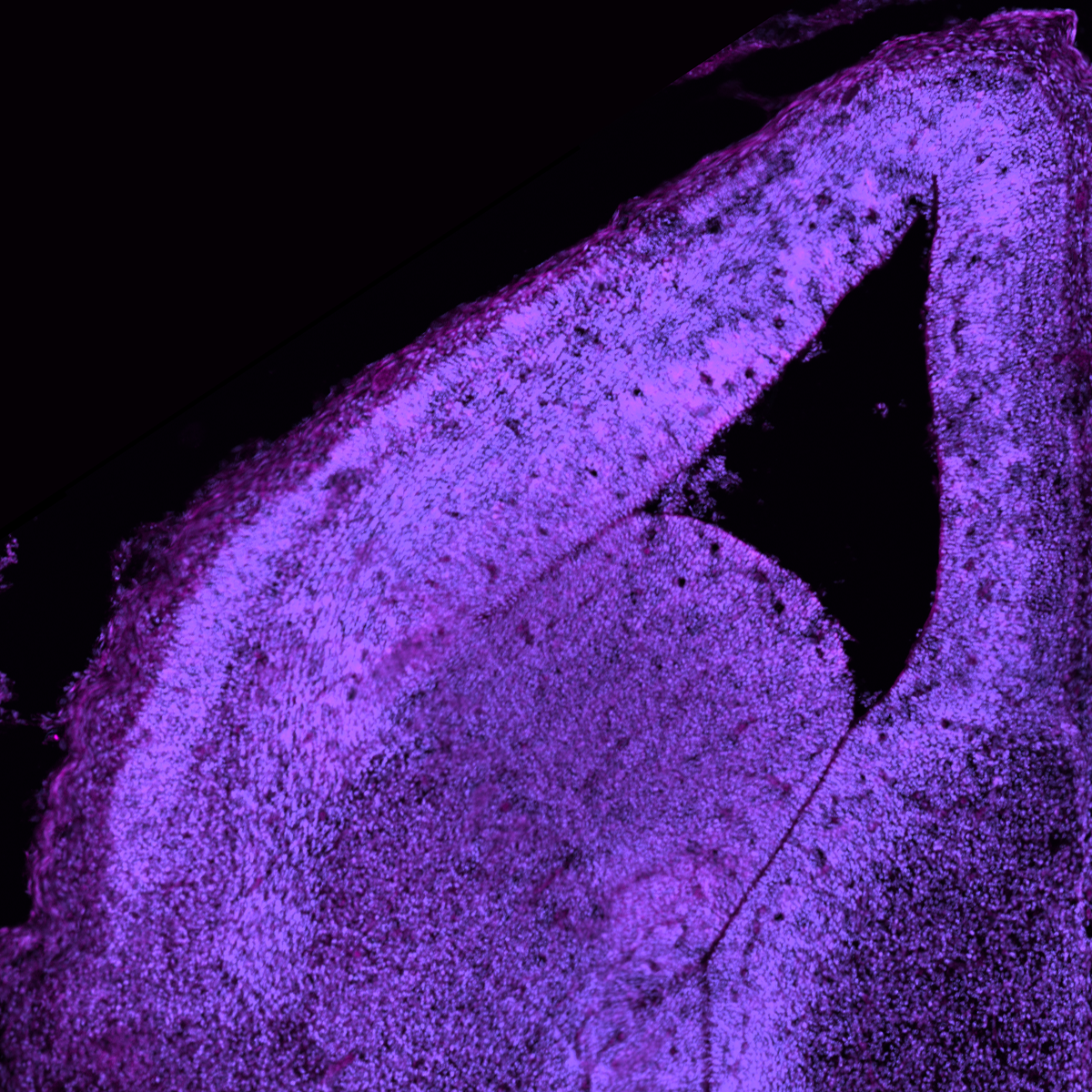

Supplement: Figure 3—source data 1. [file elife-86168-fig3-data1.zip › Figure 3 source data 1/Fig.3D 20220429 dcaf5 e14.5 wt anti-ezh2 merge cut.tif]

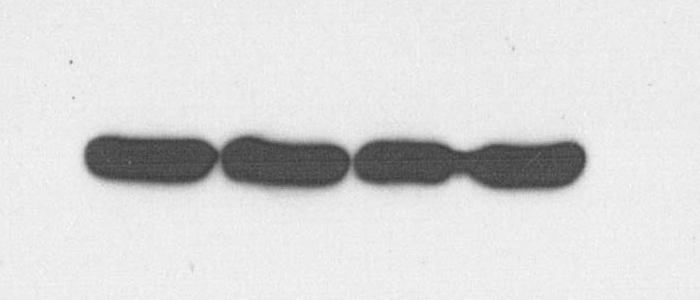

Supplement: Figure 3—source data 1. [file elife-86168-fig3-data1.zip › Figure 3 source data 1/Fig.3F 20220801 si lsd1 d5 #2 anti-actin rep3 uncropped.tif]

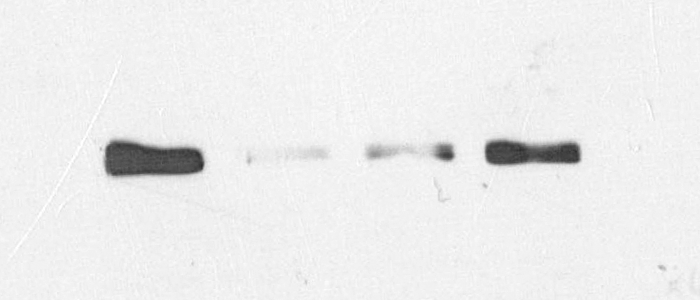

Supplement: Figure 3—source data 1. [file elife-86168-fig3-data1.zip › Figure 3 source data 1/Fig.3F 20220801 si lsd1 d5 #2 anti-lsd1 rep.3 uncropped.tif]

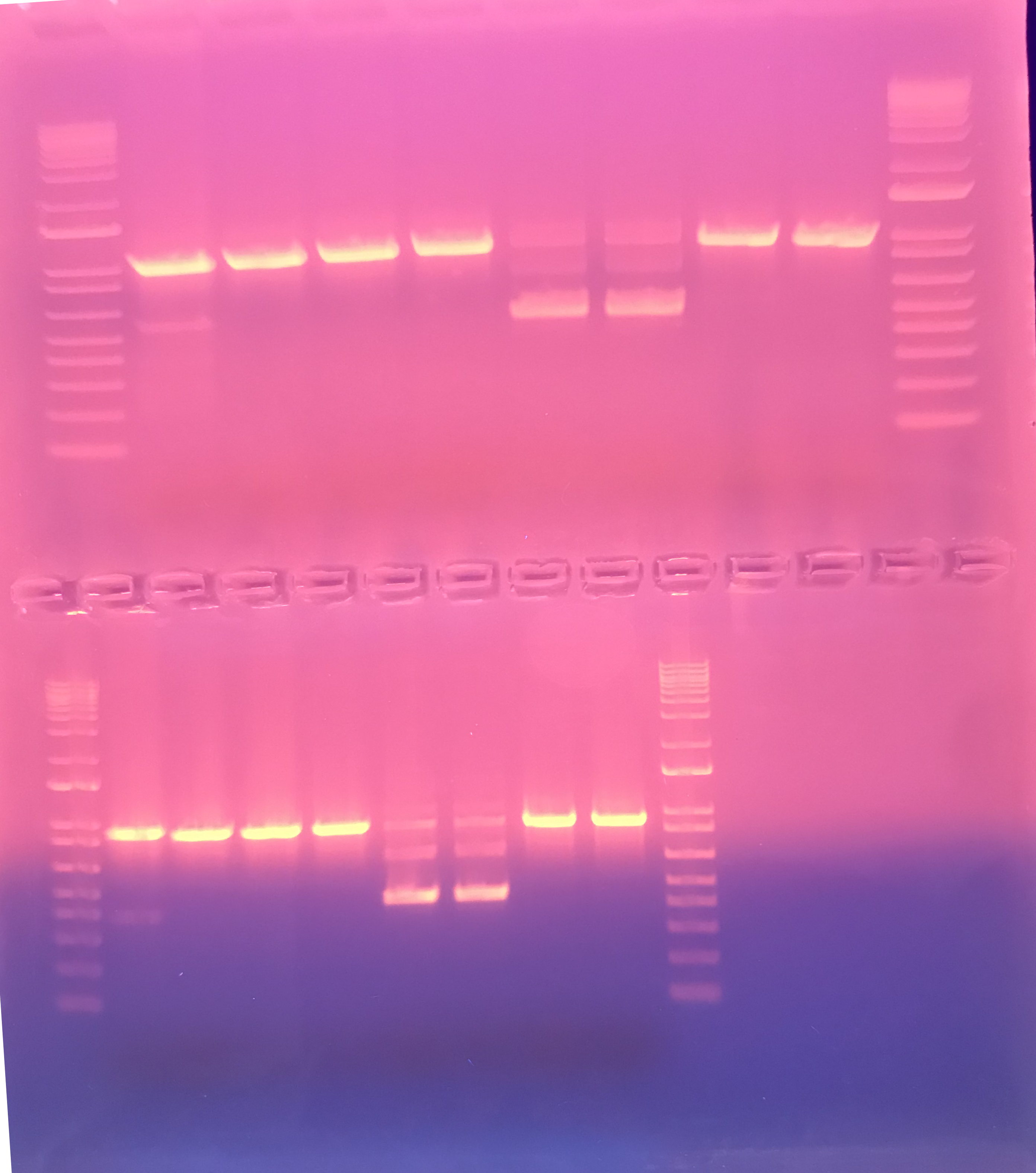

Supplement: Figure 3—source data 1. [file elife-86168-fig3-data1.zip › Figure 3 source data 1/Fig.3B Uncropped.tif]

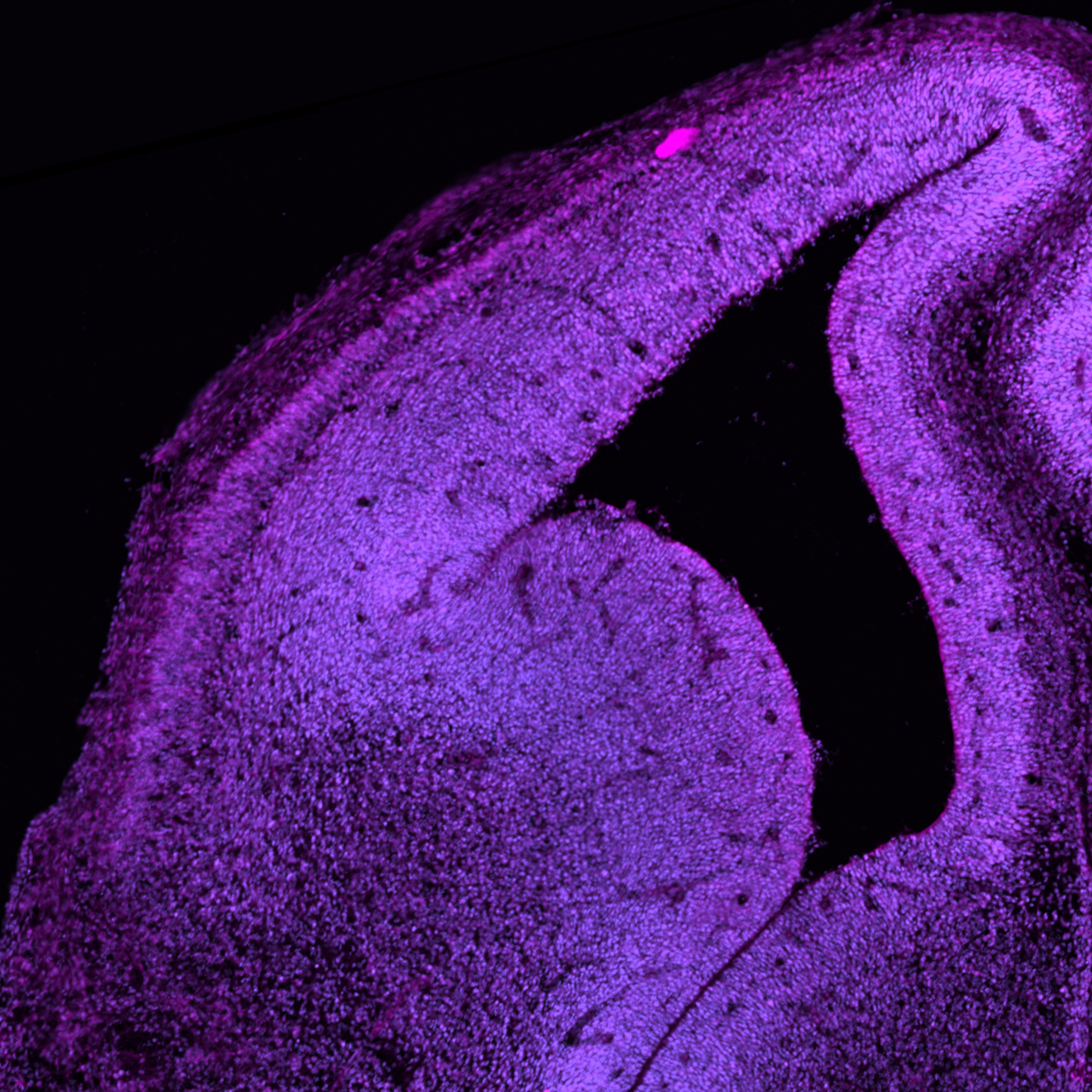

Supplement: Figure 3—source data 1. [file elife-86168-fig3-data1.zip › Figure 3 source data 1/Fig.3D 20220429 dcaf5 e14.5 ko anti-ezh2 merge cut.tif]

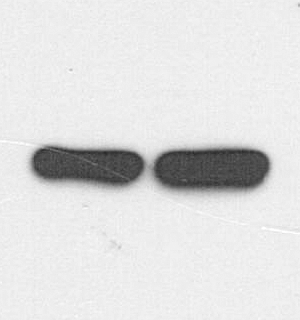

Supplement: Figure 3—source data 1. [file elife-86168-fig3-data1.zip › Figure 3 source data 1/Fig.3C 20220505 dcaf5 e14.5 head check ezh2 anti-actin uncropped.tif]

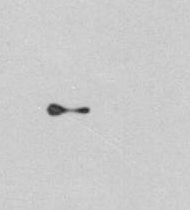

Supplement: Figure 3—source data 1. [file elife-86168-fig3-data1.zip › Figure 3 source data 1/Fig.3C 20220505 dcaf5 e14.5 head check ezh2 anti-DCAF5 uncropped.tif]

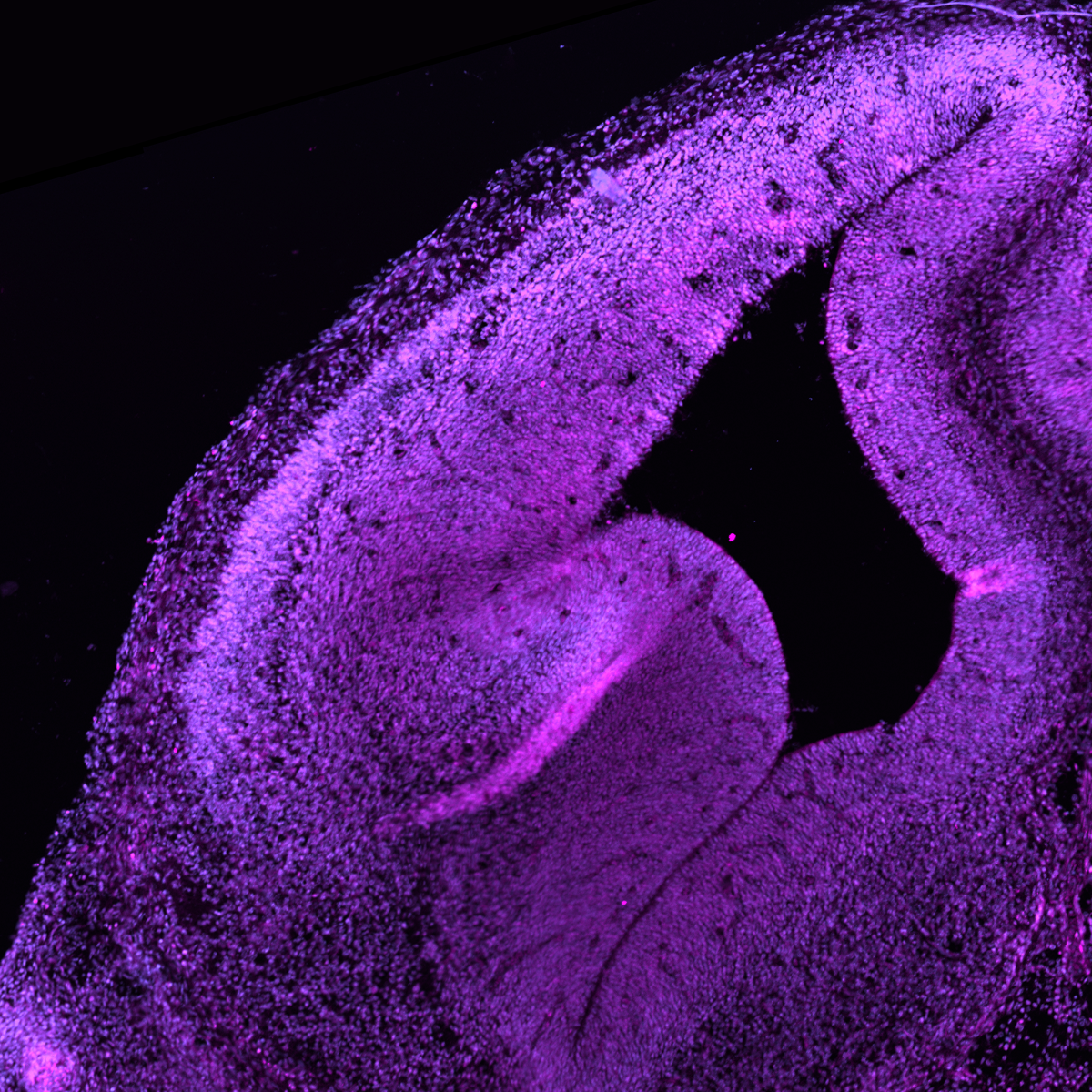

Supplement: Figure 3—source data 1. [file elife-86168-fig3-data1.zip › Figure 3 source data 1/Fig.3D 20220429 dcaf5-ko e14.5 anti-h3k27me3 merge cut.tif]

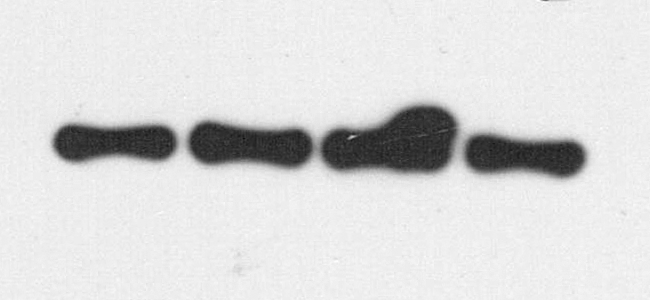

Supplement: Figure 3—source data 1. [file elife-86168-fig3-data1.zip › Figure 3 source data 1/Fig.3E 20220429 h1299-EZH2 si lsd1 D5 rescue 1 anti-Actin uncropped.tif]

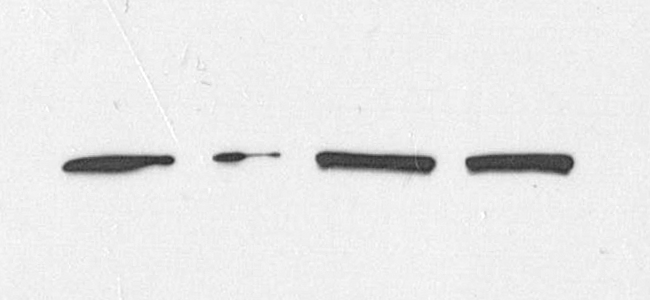

Supplement: Figure 3—source data 1. [file elife-86168-fig3-data1.zip › Figure 3 source data 1/Fig.3E 20220429 h1299-EZH2 si lsd1 D5 rescue 1 anti-flag-EZH2 uncropped.tif]

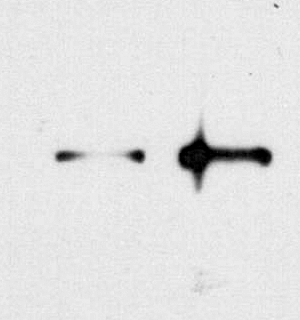

Supplement: Figure 3—source data 1. [file elife-86168-fig3-data1.zip › Figure 3 source data 1/Fig.3C 20220505 dcaf5 e14.5 head check ezh2 anti-ezh2 uncropped.tif]

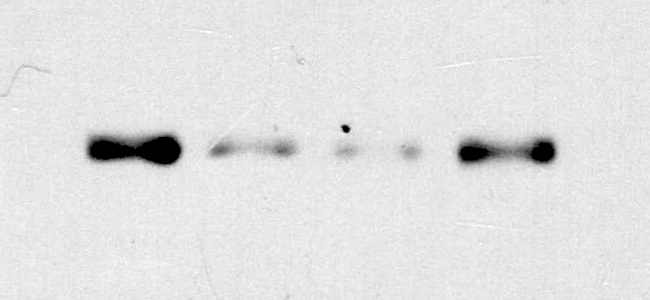

Supplement: Figure 3—source data 1. [file elife-86168-fig3-data1.zip › Figure 3 source data 1/Fig.3E 20220429 h1299-EZH2 si lsd1 D5 rescue 1 anti-LSD1 uncropped.tif]

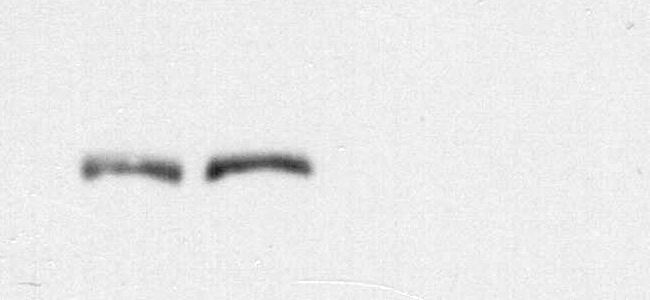

Supplement: Figure 3—source data 1. [file elife-86168-fig3-data1.zip › Figure 3 source data 1/Fig.3E 20220429 h1299-EZH2 si lsd1 D5 rescue 1 anti-D5 uncropped.tif]

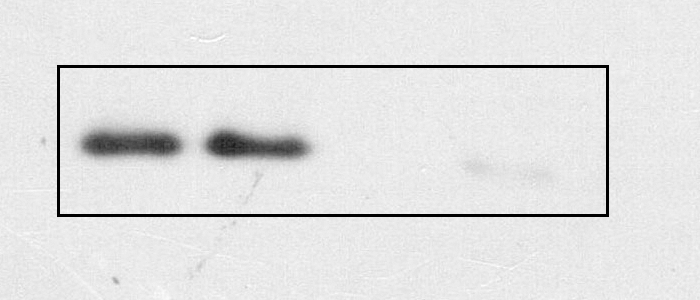

Supplement: Figure 3—source data 1. [file elife-86168-fig3-data1.zip › Figure 3 source data 1/annotated/Fig.3F 20220801 si lsd1 d5 #2 anti-d5 rep3 uncropped.tif]

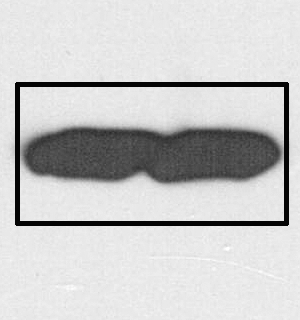

Supplement: Figure 3—source data 1. [file elife-86168-fig3-data1.zip › Figure 3 source data 1/annotated/Fig.3C 20220505 dcaf5 e14.5 head check ezh2 anti-H3 uncropped.tif]

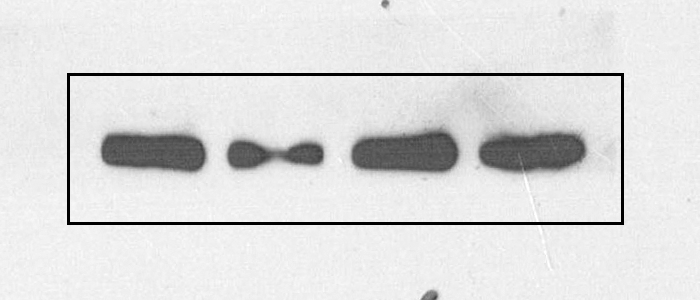

Supplement: Figure 3—source data 1. [file elife-86168-fig3-data1.zip › Figure 3 source data 1/annotated/Fig.3F 20220801 si lsd1 d5 #2 anti-flag-EZH2 uncropped.tif]

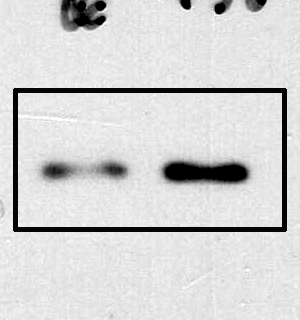

Supplement: Figure 3—source data 1. [file elife-86168-fig3-data1.zip › Figure 3 source data 1/annotated/Fig.3C 20220505 dcaf5 e14.5 head check ezh2 anti-h3k27me3 uncropped.tif]

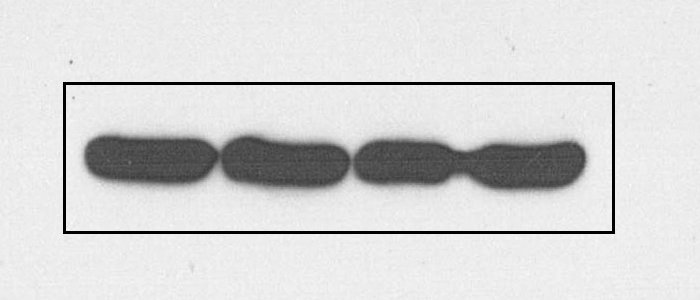

Supplement: Figure 3—source data 1. [file elife-86168-fig3-data1.zip › Figure 3 source data 1/annotated/Fig.3F 20220801 si lsd1 d5 #2 anti-actin rep3 uncropped.tif]

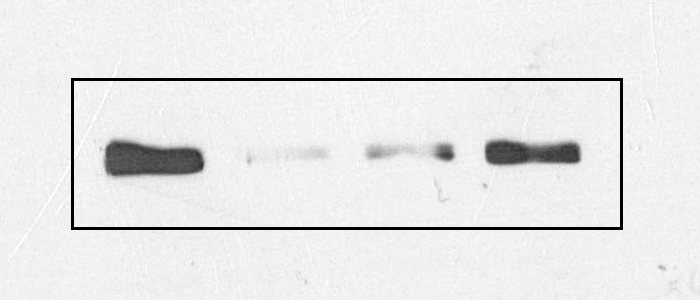

Supplement: Figure 3—source data 1. [file elife-86168-fig3-data1.zip › Figure 3 source data 1/annotated/Fig.3F 20220801 si lsd1 d5 #2 anti-lsd1 rep.3 uncropped.tif]

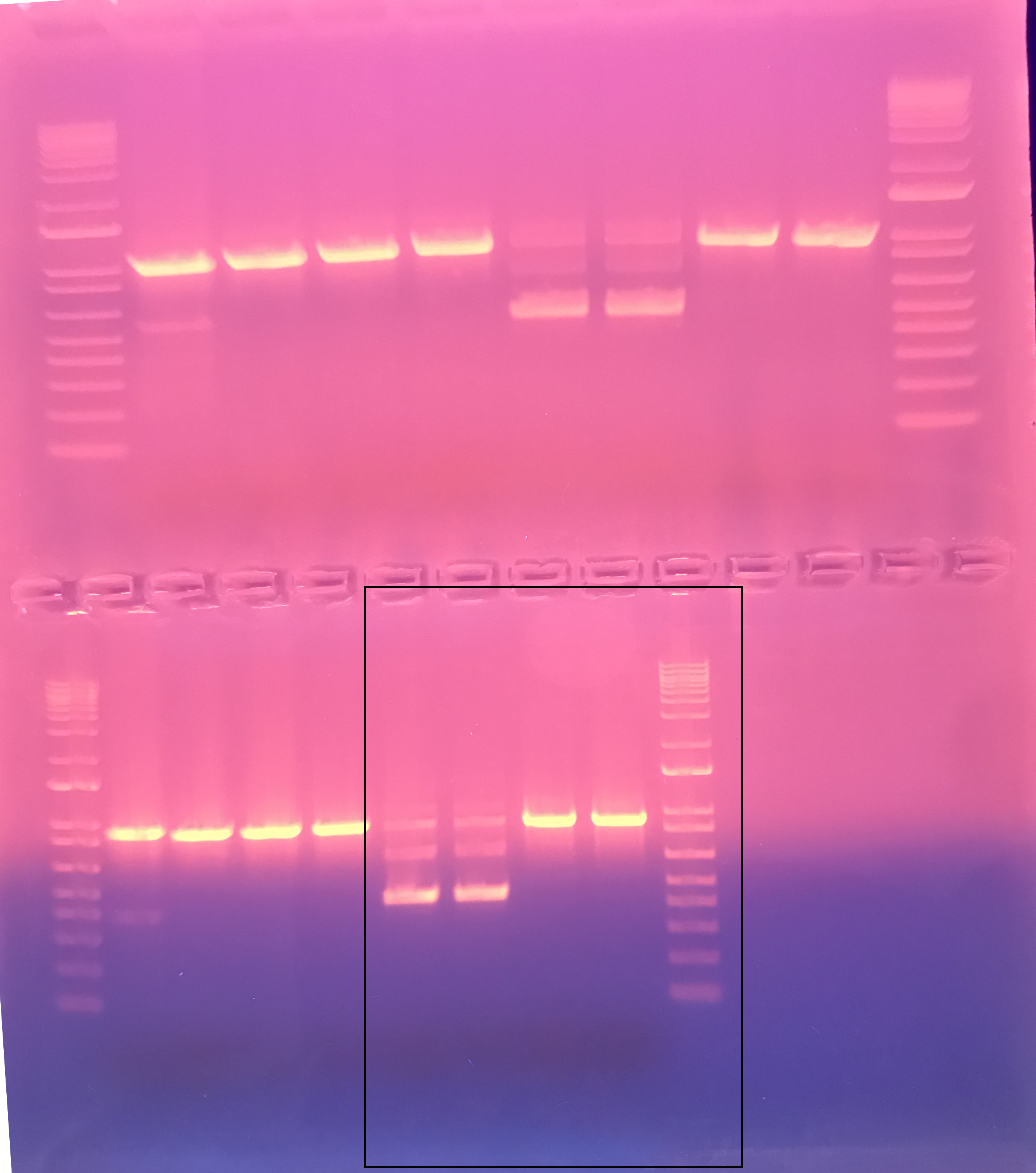

Supplement: Figure 3—source data 1. [file elife-86168-fig3-data1.zip › Figure 3 source data 1/annotated/Fig.3B Uncropped.tif]

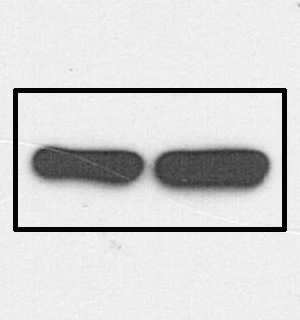

Supplement: Figure 3—source data 1. [file elife-86168-fig3-data1.zip › Figure 3 source data 1/annotated/Fig.3C 20220505 dcaf5 e14.5 head check ezh2 anti-actin uncropped.tif]

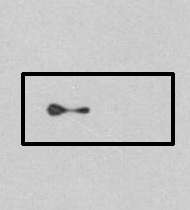

Supplement: Figure 3—source data 1. [file elife-86168-fig3-data1.zip › Figure 3 source data 1/annotated/Fig.3C 20220505 dcaf5 e14.5 head check ezh2 anti-DCAF5 uncropped.tif]

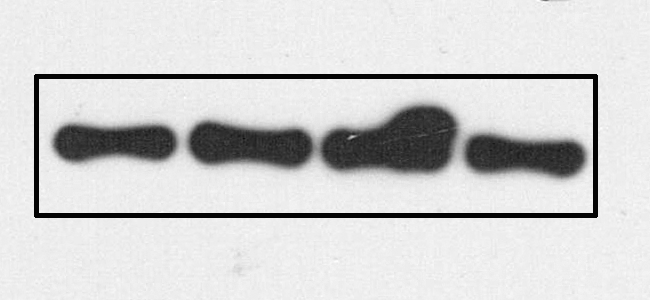

Supplement: Figure 3—source data 1. [file elife-86168-fig3-data1.zip › Figure 3 source data 1/annotated/Fig.3E 20220429 h1299-EZH2 si lsd1 D5 rescue 1 anti-Actin uncropped.tif]

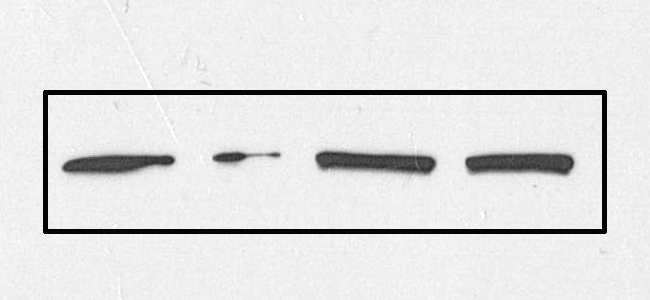

Supplement: Figure 3—source data 1. [file elife-86168-fig3-data1.zip › Figure 3 source data 1/annotated/Fig.3E 20220429 h1299-EZH2 si lsd1 D5 rescue 1 anti-flag-EZH2 uncropped.tif]

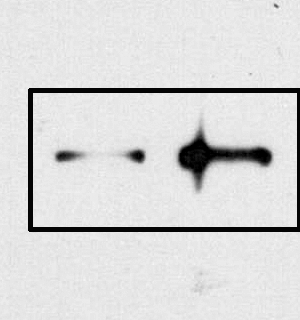

Supplement: Figure 3—source data 1. [file elife-86168-fig3-data1.zip › Figure 3 source data 1/annotated/Fig.3C 20220505 dcaf5 e14.5 head check ezh2 anti-ezh2 uncropped.tif]

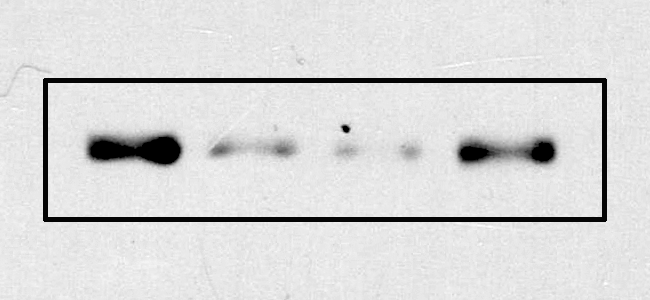

Supplement: Figure 3—source data 1. [file elife-86168-fig3-data1.zip › Figure 3 source data 1/annotated/Fig.3E 20220429 h1299-EZH2 si lsd1 D5 rescue 1 anti-LSD1 uncropped.tif]

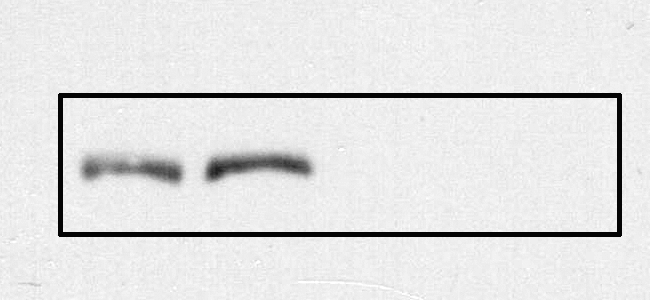

Supplement: Figure 3—source data 1. [file elife-86168-fig3-data1.zip › Figure 3 source data 1/annotated/Fig.3E 20220429 h1299-EZH2 si lsd1 D5 rescue 1 anti-D5 uncropped.tif]

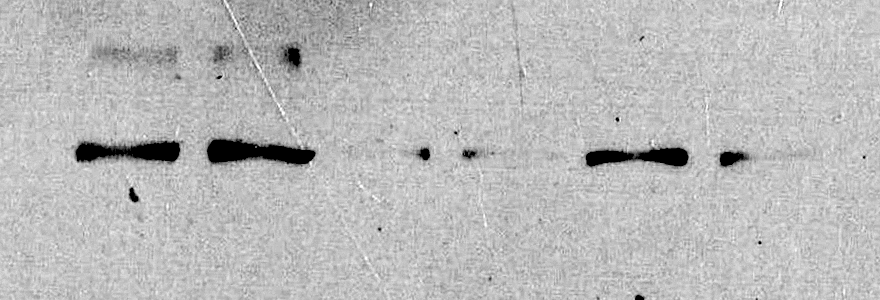

Supplement: Figure 4—source data 1. [file elife-86168-fig4-data1.zip › Figure 4 source data 1/Fig.4E 202303 Nestin-cre lsd1flox Check EZH2K20me anti-LSD1 uncropped.tif]

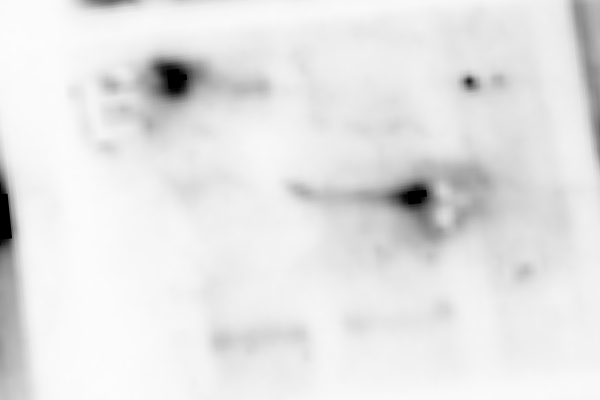

Supplement: Figure 4—source data 1. [file elife-86168-fig4-data1.zip › Figure 4 source data 1/Fig.4F mouse e15 embryo wt l3-ko ip EZH2 input anti-ezh2-k20me uncropped.tif]

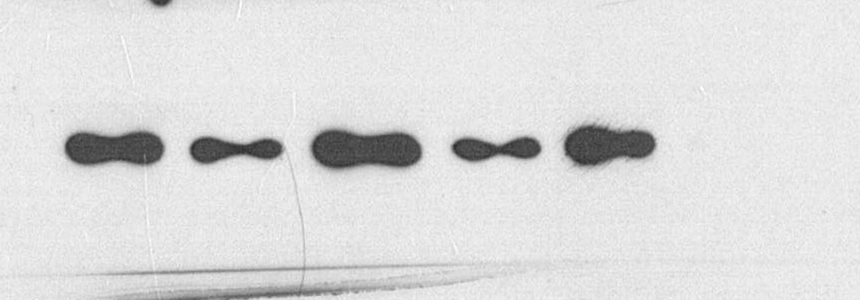

Supplement: Figure 4—source data 1. [file elife-86168-fig4-data1.zip › Figure 4 source data 1/Fig.4G 20230326 HCT116 transfect with set7 check EZH2 ANti-H3K27me3 uncropped.tif]

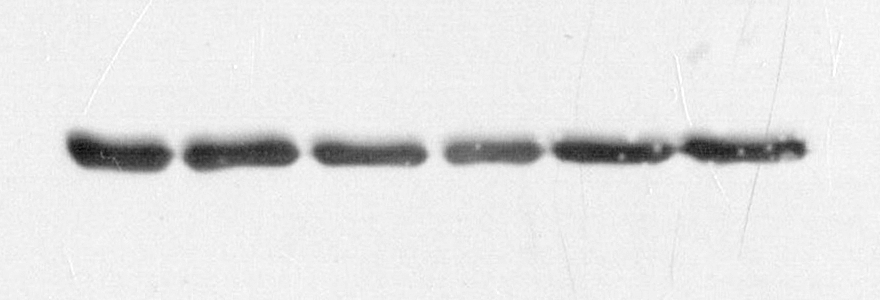

Supplement: Figure 4—source data 1. [file elife-86168-fig4-data1.zip › Figure 4 source data 1/Fig.4D hct116 si LSD1 -MG132 anti-H3 Uncropped.tif]

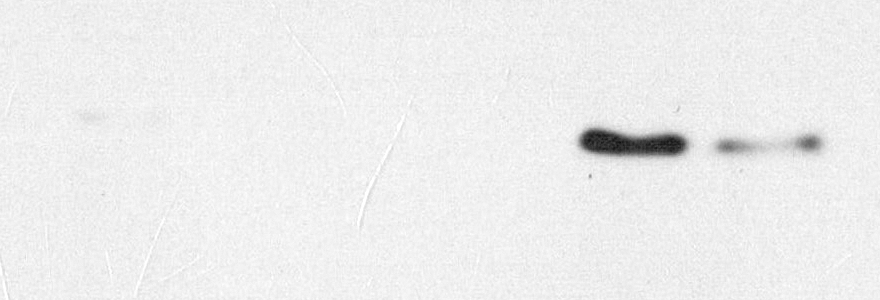

Supplement: Figure 4—source data 1. [file elife-86168-fig4-data1.zip › Figure 4 source data 1/Fig.4E 202303 Nestin-cre lsd1flox Check EZH2K20me anti-H3K27me3 uncropped.tif]

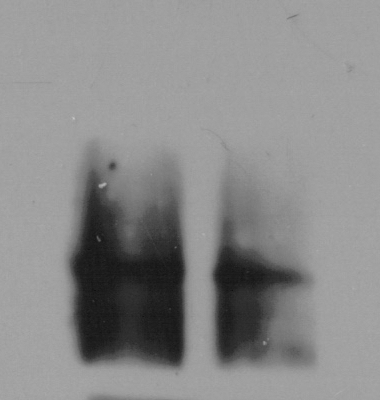

Supplement: Figure 4—source data 1. [file elife-86168-fig4-data1.zip › Figure 4 source data 1/Fig.4H 20200614 293-set7 l3-ip ip anti-l3.tif]

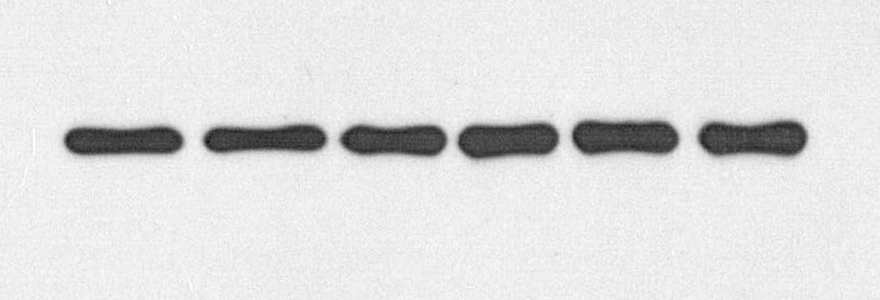

Supplement: Figure 4—source data 1. [file elife-86168-fig4-data1.zip › Figure 4 source data 1/Fig.4E 202303 Nestin-cre lsd1flox Check EZH2K20me anti-H3 uncropped.tif]

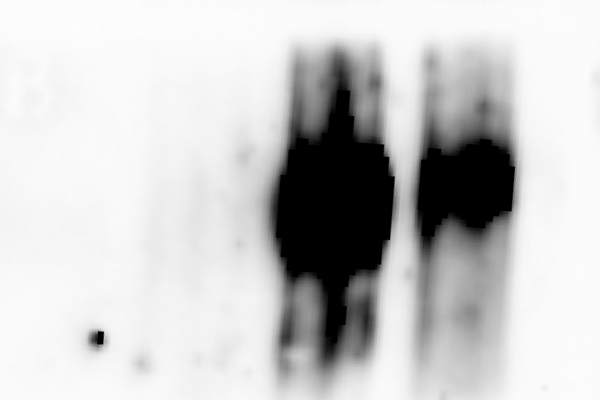

Supplement: Figure 4—source data 1. [file elife-86168-fig4-data1.zip › Figure 4 source data 1/Fig.4F mouse e15 embryo wt l3-ko ip EZH2 ip anti-ezh2 uncropped.tif]

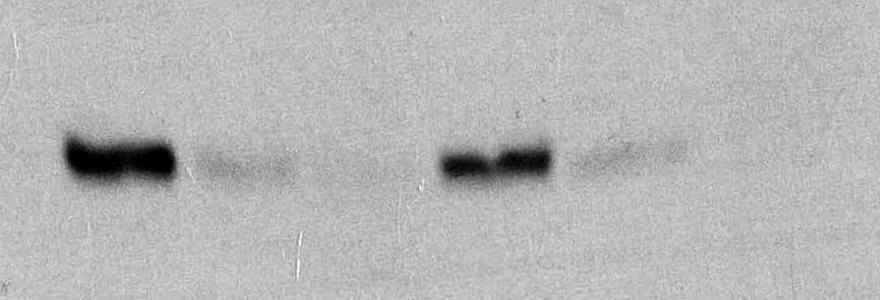

Supplement: Figure 4—source data 1. [file elife-86168-fig4-data1.zip › Figure 4 source data 1/Fig.4D hct116 si LSD1 -MG132 anti-LSD1 Uncropped.tif]

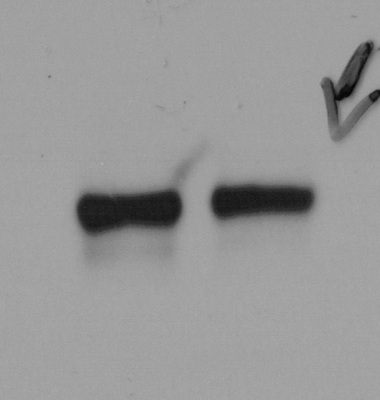

Supplement: Figure 4—source data 1. [file elife-86168-fig4-data1.zip › Figure 4 source data 1/Fig.4H 20200614 293-set7 l3-ip input anti-ezh2.tif]

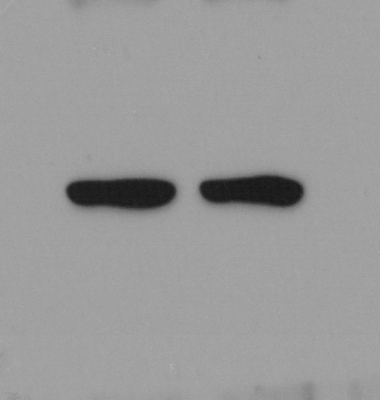

Supplement: Figure 4—source data 1. [file elife-86168-fig4-data1.zip › Figure 4 source data 1/Fig.4H 20200614 293-set7 l3-ip anti-actin.tif]

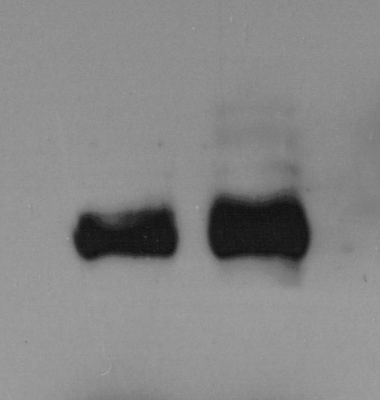

Supplement: Figure 4—source data 1. [file elife-86168-fig4-data1.zip › Figure 4 source data 1/Fig.4H 20200614 293-set7 l3-ip input anti-l3.tif]

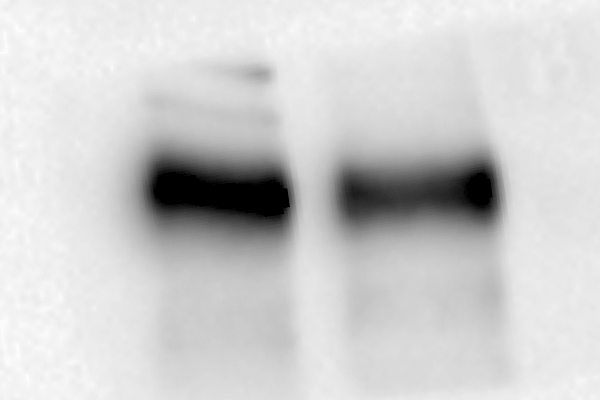

Supplement: Figure 4—source data 1. [file elife-86168-fig4-data1.zip › Figure 4 source data 1/Fig.4F mouse e15 embryo wt l3-ko ip EZH2 input anti-ezh2 uncropped.tif]

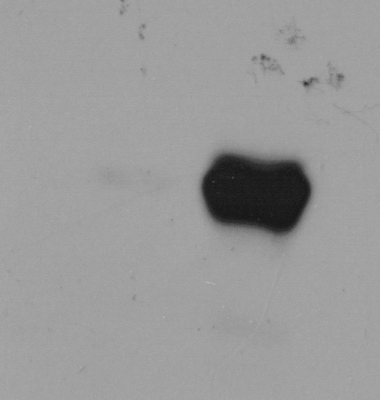

Supplement: Figure 4—source data 1. [file elife-86168-fig4-data1.zip › Figure 4 source data 1/Fig.4H 20200614 293-set7 l3-ip anti-set7.tif]

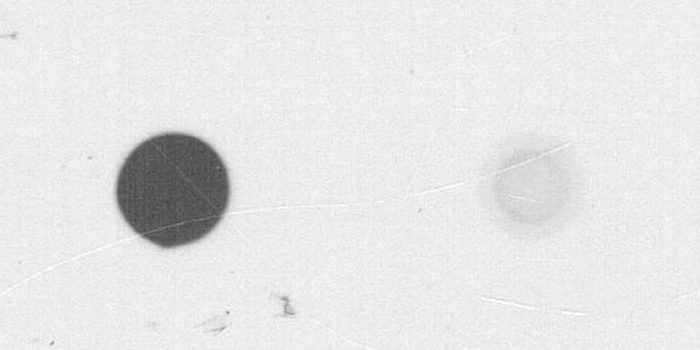

Supplement: Figure 4—source data 1. [file elife-86168-fig4-data1.zip › Figure 4 source data 1/Fig.4C 20220719 ezh2 gst- lsd1 demethylation anti-K20me uncropped.tif]

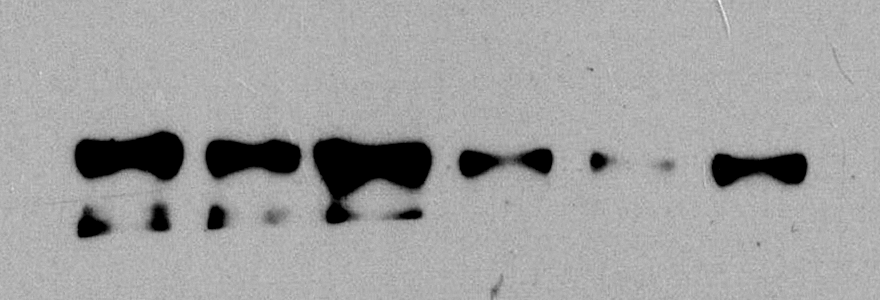

Supplement: Figure 4—source data 1. [file elife-86168-fig4-data1.zip › Figure 4 source data 1/Fig.4D hct116 si LSD1 -MG132 anti-ezh2K20me Uncropped.tif]

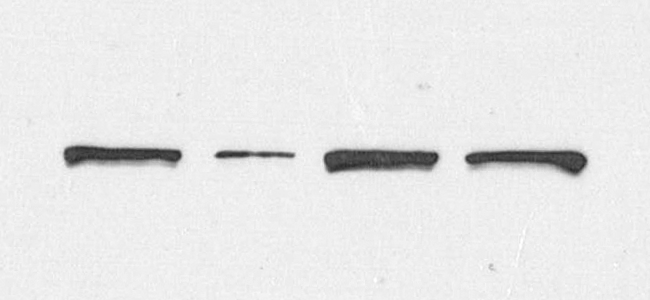

Supplement: Figure 4—source data 1. [file elife-86168-fig4-data1.zip › Figure 4 source data 1/Fig.4I Western si lsd1 set7 rescue 1 anti-flag-EZH2 uncropped.tif]

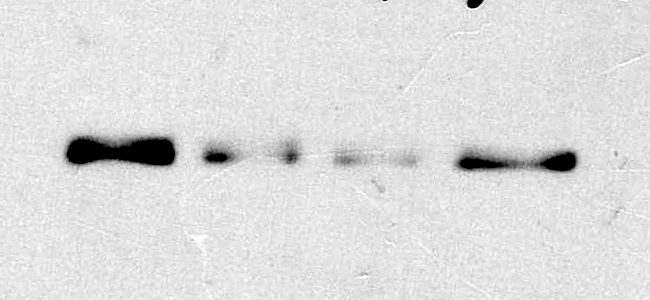

Supplement: Figure 4—source data 1. [file elife-86168-fig4-data1.zip › Figure 4 source data 1/Fig.4I Western si lsd1 set7 rescue 1 anti-lsd1 uncropped.tif]

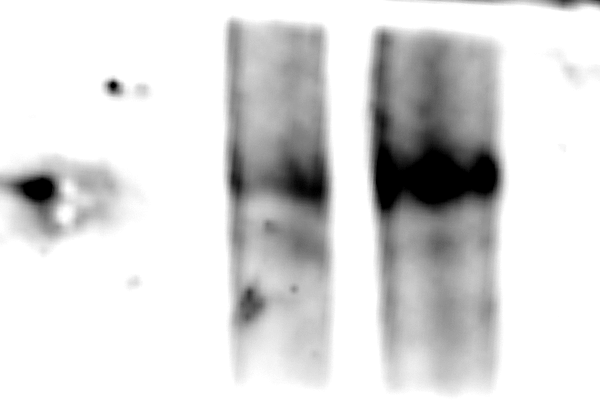

Supplement: Figure 4—source data 1. [file elife-86168-fig4-data1.zip › Figure 4 source data 1/Fig.4F mouse e15 embryo wt l3-ko ip EZH2 ip anti-ezh2-k20me uncropped.tif]

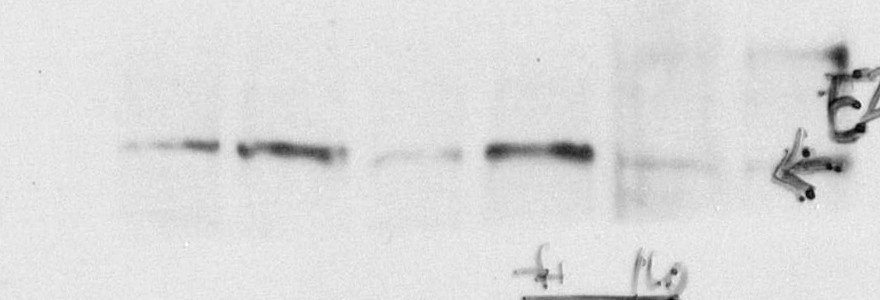

Supplement: Figure 4—source data 1. [file elife-86168-fig4-data1.zip › Figure 4 source data 1/Fig.4E 202303 Nestin-cre lsd1flox Check EZH2K20me anti-EZH2 uncropped.tif]

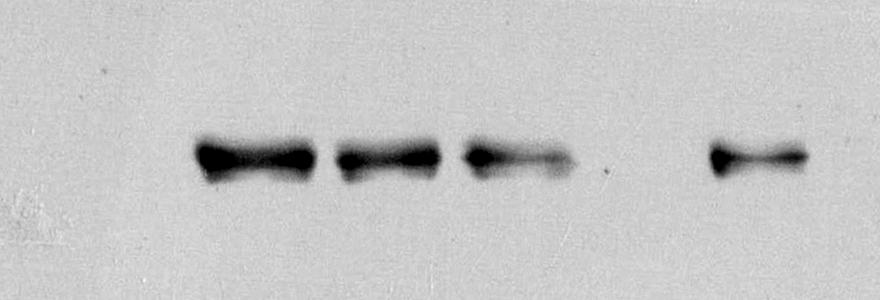

Supplement: Figure 4—source data 1. [file elife-86168-fig4-data1.zip › Figure 4 source data 1/Fig.4D hct116 si LSD1 -MG132 anti-H3K27me3 Uncropped.tif]

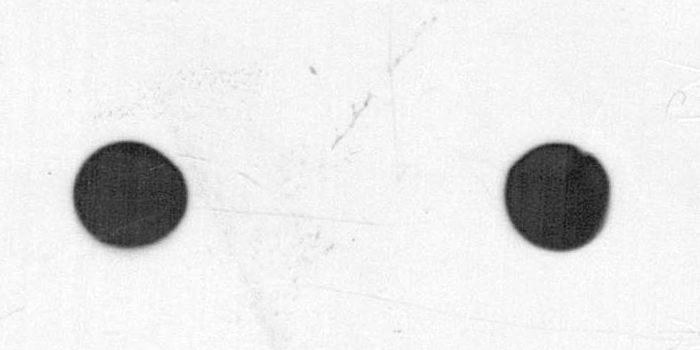

Supplement: Figure 4—source data 1. [file elife-86168-fig4-data1.zip › Figure 4 source data 1/Fig.4C 20220719 ezh2 gst- lsd1 demethylation anti-EZH2 uncropped.tif]

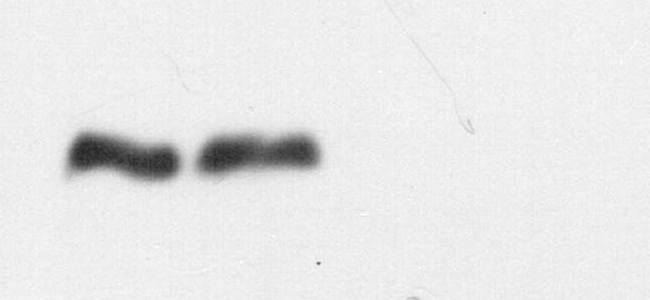

Supplement: Figure 4—source data 1. [file elife-86168-fig4-data1.zip › Figure 4 source data 1/Fig.4I Western si lsd1 set7 rescue 1 anti-set7 uncropped.tif]

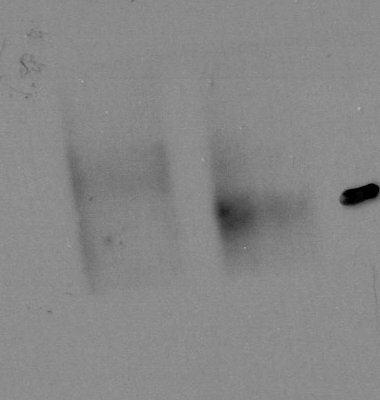

Supplement: Figure 4—source data 1. [file elife-86168-fig4-data1.zip › Figure 4 source data 1/Fig.4H 20200614 293-set7 l3-ip ip anti-ezh2-k20me.tif]
