# Supplementary material for: A methylation-phosphorylation switch controls EZH2 stability and hematopoiesis
Source: eLife. 2024 Feb 12;13:e86168. doi: 10.7554/eLife.86168 (PMC10901513; doi:10.7554/eLife.86168)

Fig.2-figure supplement -2A- EZH2

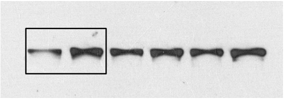

Fig.2-figure supplement -2A-Flag-L3MBTL3

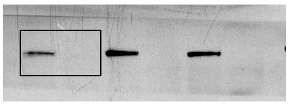

Fig.2-figure supplement -2A- GAPDH

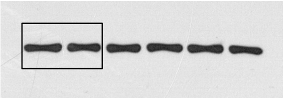

Fig.2-figure supplement -2B- EZH2

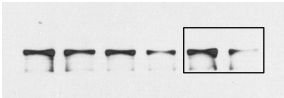

Fig.2-figure supplement -2B-Flag-L3MBTL3

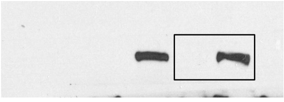

Fig.2-figure supplement -2B- GAPDH

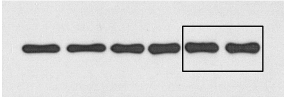

Supplement: Figure 2—figure supplement 2—source data 1. [file elife-86168-fig2-figsupp2-data1.zip › Figure 2-figure supplement2 source data 1/Figure2-figure supplement 2.pdf]
