# Supplementary material for: A methylation-phosphorylation switch controls EZH2 stability and hematopoiesis
Source: eLife. 2024 Feb 12;13:e86168. doi: 10.7554/eLife.86168 (PMC10901513; doi:10.7554/eLife.86168)

Figure 3B-DCAF5 PCR

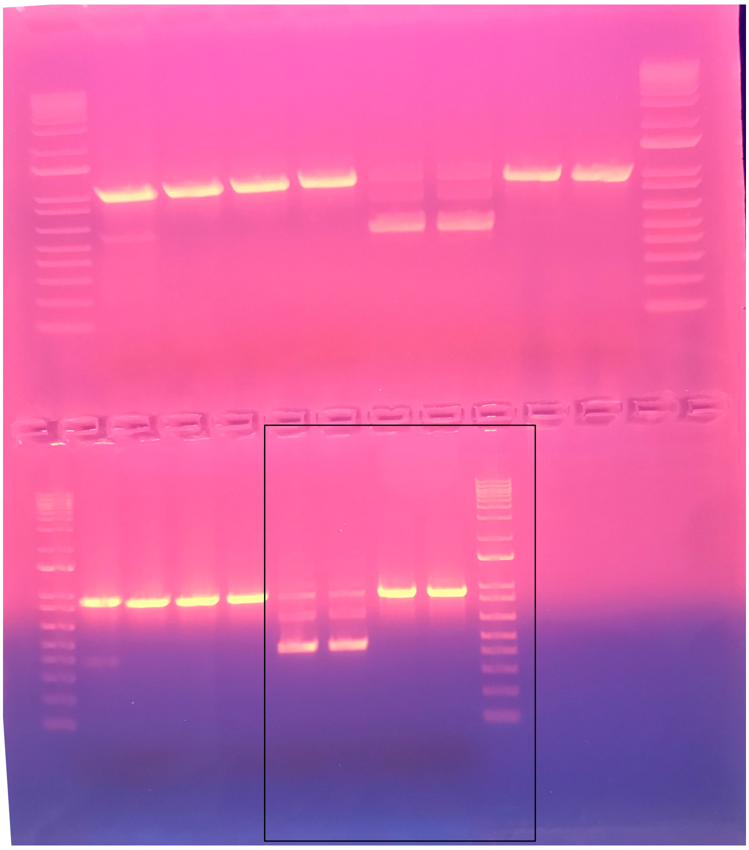

Figure 3C-EZH2

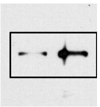

Figure 3C-DCAF5

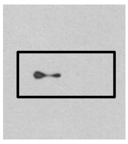

Figure 3C-Actin

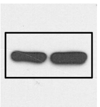

Figure 3C-H3K27me3

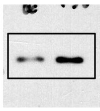

Figure 3C-H3

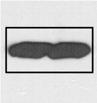

Figure 3E-Flag-EZH2

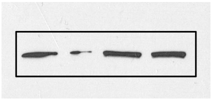

Figure 3E-Actin

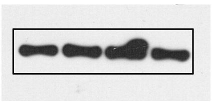

Figure 3E-DCAF5

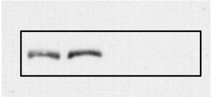

Figure 3E-LSD1

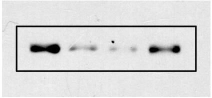

Figure 3F-Flag-EZH2

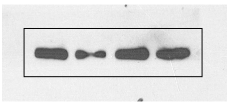

Figure 3F-Actin

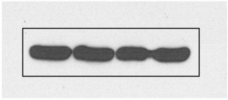

Figure 3F-DCAF5

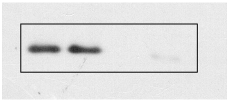

Figure 3F-LSD1

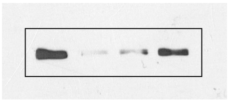

Supplement: Figure 3—source data 1. [file elife-86168-fig3-data1.zip › Figure 3 source data 1/Figure 3-annotated source data.pdf]
