# Supplementary material for: A methylation-phosphorylation switch controls EZH2 stability and hematopoiesis
Source: eLife. 2024 Feb 12;13:e86168. doi: 10.7554/eLife.86168 (PMC10901513; doi:10.7554/eLife.86168)

Figure 4C-EZH2-K20me

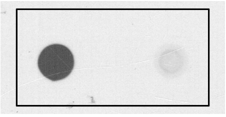

Figure 4C-EZH2

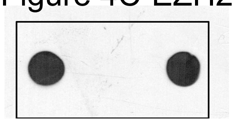

Figure 4D-EZH2

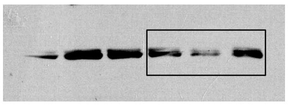

Figure 4D-EZH2K20me

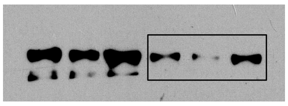

Figure 4D-LSD1

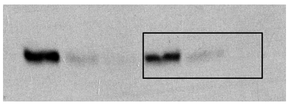

Figure 4D-H3K27me3

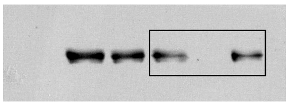

Figure 4D-H3

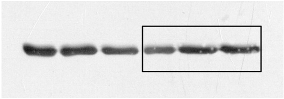

Figure 4E-EZH2

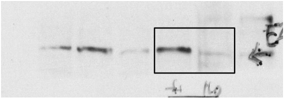

Figure 4E-EZH2K20me

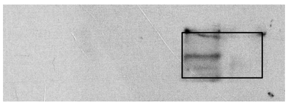

Figure 4E-LSD1

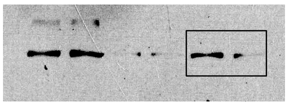

Figure 4E-H3K27me3

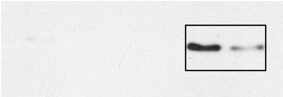

Figure 4E-H3

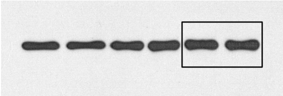

Figure 4F-EZH-K20me

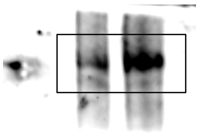

Figure 4F-EZH2

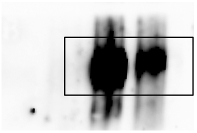

Figure 4F-L3MBTL3

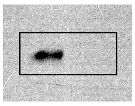

Figure 4F-EZH2-K20me

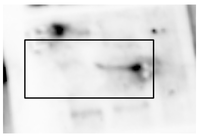

Figure 4F-EZH2

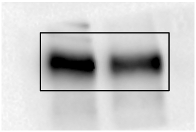

Figure 4G-EZH2

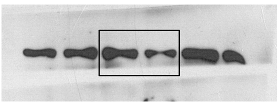

Figure 4G-SET7

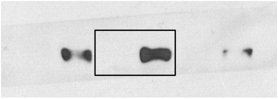

Figure 4G-H3K27me3

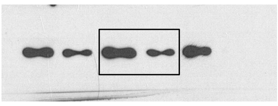

Figure 4G-H3

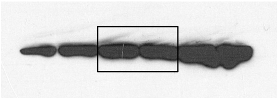

Figure 4H-EZH2-K20me

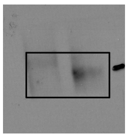

Figure 4H-L3MBTL3

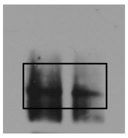

Figure 4H-EZH2

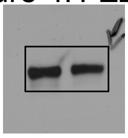

Figure 4H-Actin

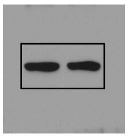

Figure 4H-SET7

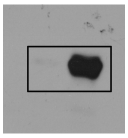

Figure 4H-L3MBTL3

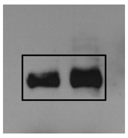

Figure 4I-Flag-EZH2

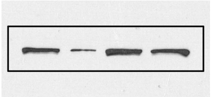

Figure 4I-Actin

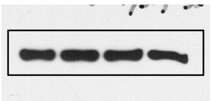

Figure 4I-SET7

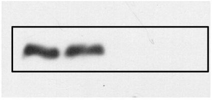

Figure 4I-LSD1

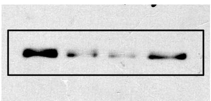

Supplement: Figure 4—source data 1. [file elife-86168-fig4-data1.zip › Figure 4 source data 1/Figure 4-annotated source data.pdf]
