# Supplementary figures and images for: A methylation-phosphorylation switch controls EZH2 stability and hematopoiesis (part 3 of 7)
Source: eLife. 2024 Feb 12;13:e86168. doi: 10.7554/eLife.86168 (PMC10901513; doi:10.7554/eLife.86168)

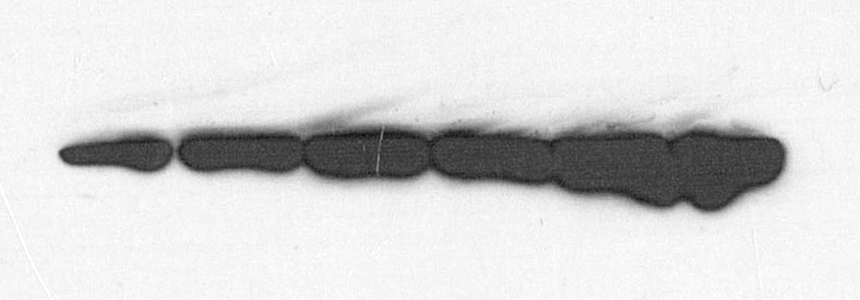

Supplement: Figure 4—source data 1. [file elife-86168-fig4-data1.zip › Figure 4 source data 1/Fig.4G 20230326 HCT116 transfect with set7 check EZH2 ANti-H3 uncropped.tif]

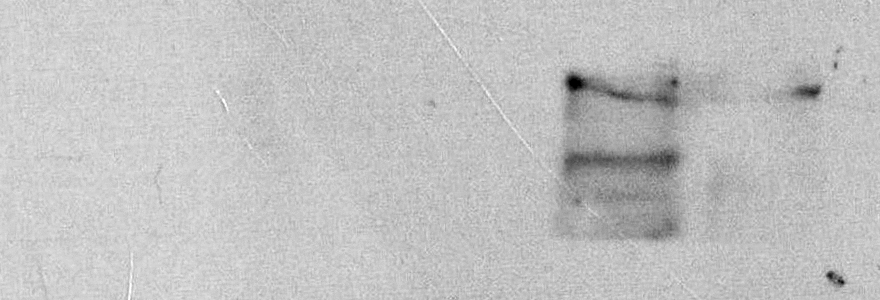

Supplement: Figure 4—source data 1. [file elife-86168-fig4-data1.zip › Figure 4 source data 1/Fig.4E 202303 Nestin-cre lsd1flox Check EZH2K20me anti-EZH2-K20me uncropped.tif]

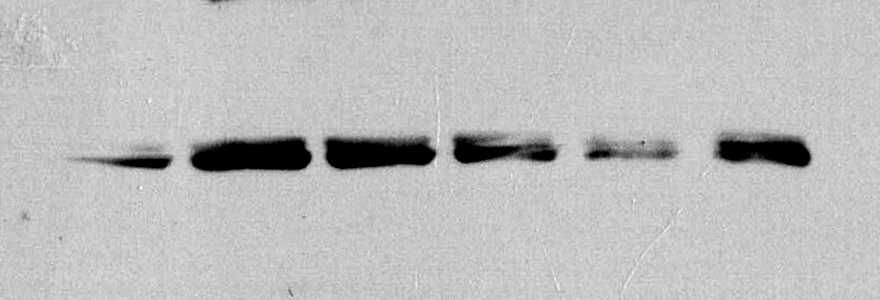

Supplement: Figure 4—source data 1. [file elife-86168-fig4-data1.zip › Figure 4 source data 1/Fig.4D hct116 si LSD1 -MG132 anti-ezh2 Uncropped.tif]

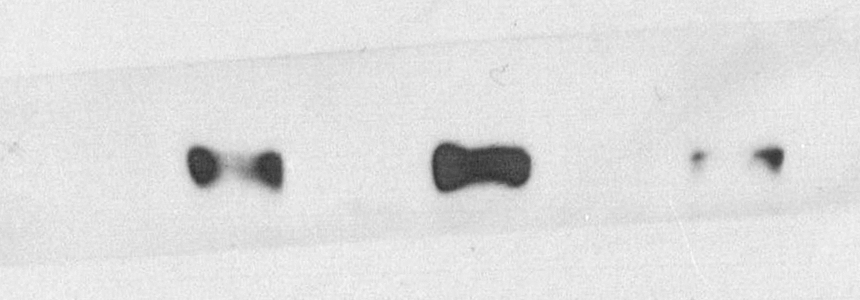

Supplement: Figure 4—source data 1. [file elife-86168-fig4-data1.zip › Figure 4 source data 1/Fig.4G 20230326 HCT116 transfect with set7 check EZH2 ANti-set7 uncropped.tif]

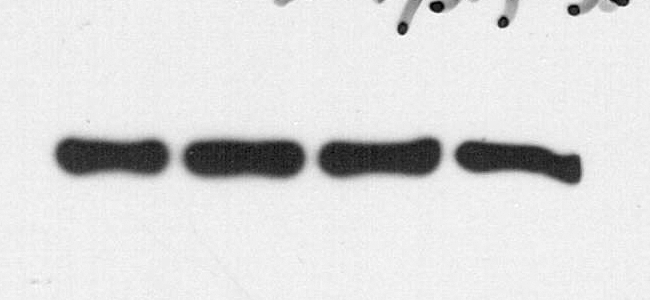

Supplement: Figure 4—source data 1. [file elife-86168-fig4-data1.zip › Figure 4 source data 1/Fig.4I Western si lsd1 set7 rescue 1 anti-actin uncropped.tif]

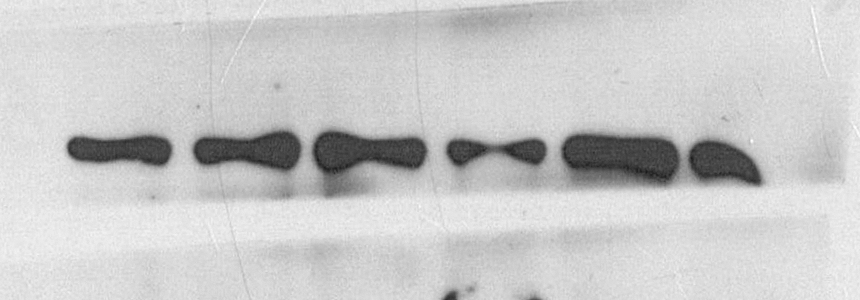

Supplement: Figure 4—source data 1. [file elife-86168-fig4-data1.zip › Figure 4 source data 1/Fig.4G 20230326 HCT116 transfect with set7 check EZH2 ANti-ezh2 uncropped.tif]

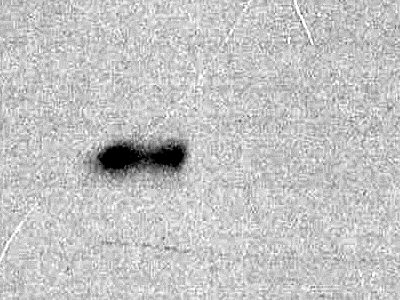

Supplement: Figure 4—source data 1. [file elife-86168-fig4-data1.zip › Figure 4 source data 1/Fig.4F mouse e15 embryo wt l3-ko ip EZH2 input anti-l3mbtl3 uncropped.tif]

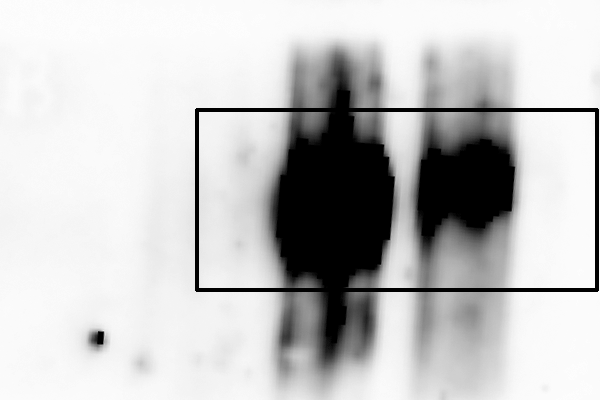

Supplement: Figure 4—source data 1. [file elife-86168-fig4-data1.zip › Figure 4 source data 1/annotated/Fig.4F mouse e15 embryo wt l3-ko ip EZH2 ip anti-ezh2 uncropped.tif]

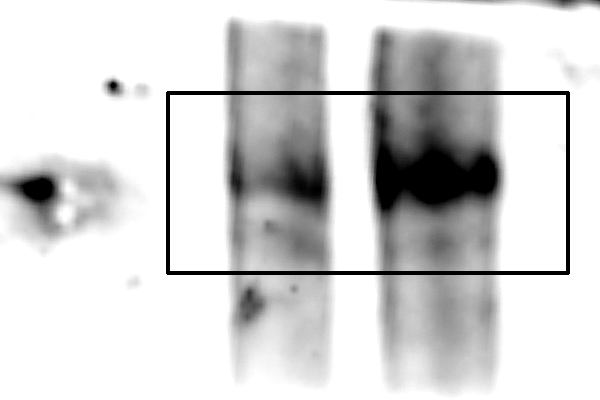

Supplement: Figure 4—source data 1. [file elife-86168-fig4-data1.zip › Figure 4 source data 1/annotated/Fig.4F mouse e15 embryo wt l3-ko ip EZH2 ip anti-ezh2-k20me uncropped.tif]

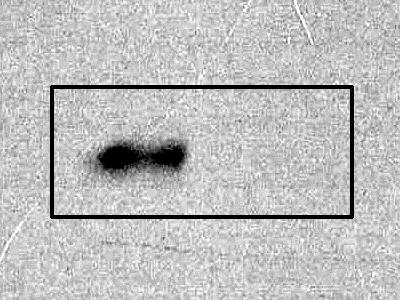

Supplement: Figure 4—source data 1. [file elife-86168-fig4-data1.zip › Figure 4 source data 1/annotated/Fig.4F mouse e15 embryo wt l3-ko ip EZH2 input anti-l3mbtl3 uncropped.tif]

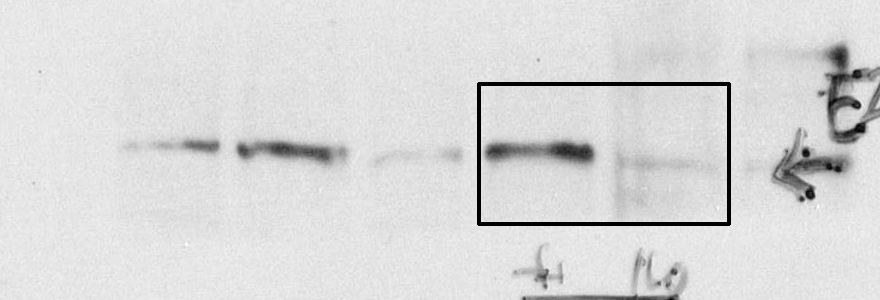

Supplement: Figure 4—source data 1. [file elife-86168-fig4-data1.zip › Figure 4 source data 1/annotated/Fig.4E 202303 Nestin-cre lsd1flox Check EZH2K20me anti-EZH2 uncropped.tif]

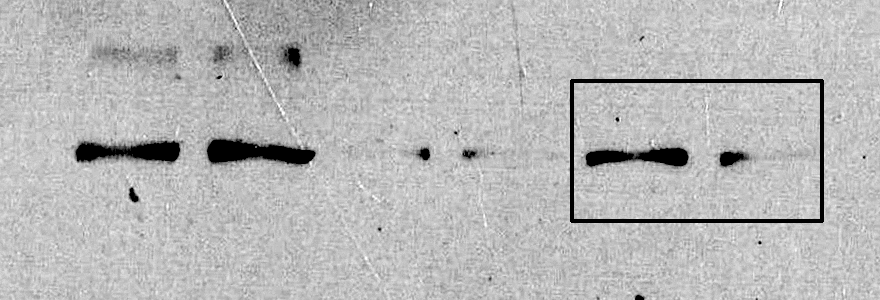

Supplement: Figure 4—source data 1. [file elife-86168-fig4-data1.zip › Figure 4 source data 1/annotated/Fig.4E 202303 Nestin-cre lsd1flox Check EZH2K20me anti-LSD1 uncropped.tif]

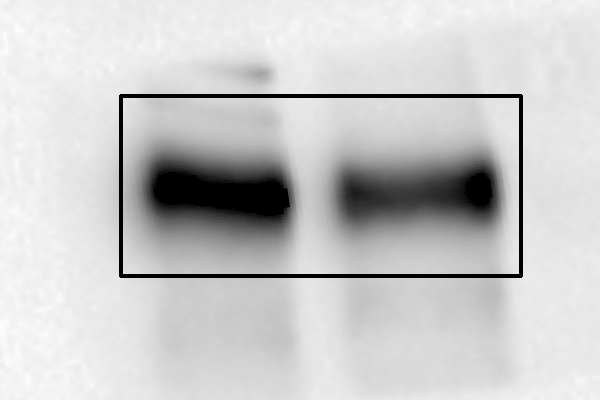

Supplement: Figure 4—source data 1. [file elife-86168-fig4-data1.zip › Figure 4 source data 1/annotated/Fig.4F mouse e15 embryo wt l3-ko ip EZH2 input anti-ezh2 uncropped.tif]

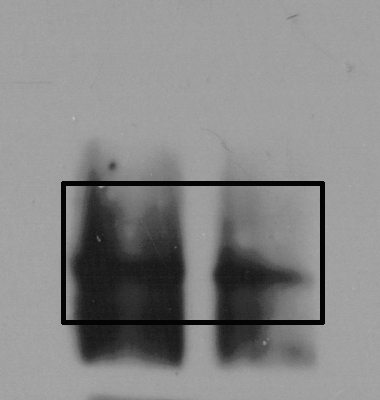

Supplement: Figure 4—source data 1. [file elife-86168-fig4-data1.zip › Figure 4 source data 1/annotated/Fig.4H 20200614 293-set7 l3-ip ip anti-l3.tif]

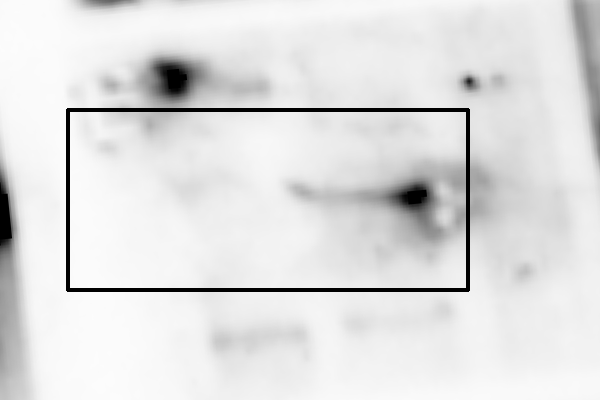

Supplement: Figure 4—source data 1. [file elife-86168-fig4-data1.zip › Figure 4 source data 1/annotated/Fig.4F mouse e15 embryo wt l3-ko ip EZH2 input anti-ezh2-k20me uncropped.tif]

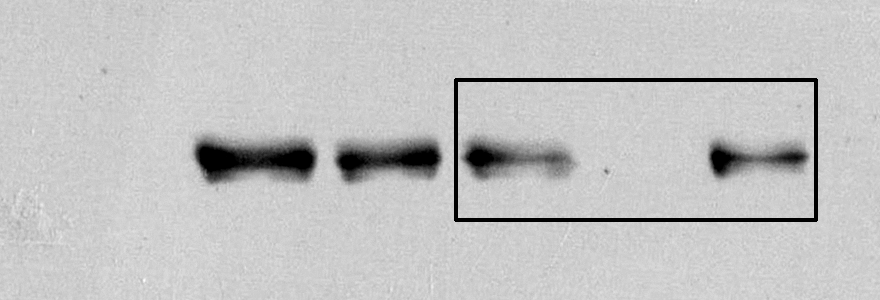

Supplement: Figure 4—source data 1. [file elife-86168-fig4-data1.zip › Figure 4 source data 1/annotated/Fig.4D hct116 si LSD1 -MG132 anti-H3K27me3 Uncropped.tif]

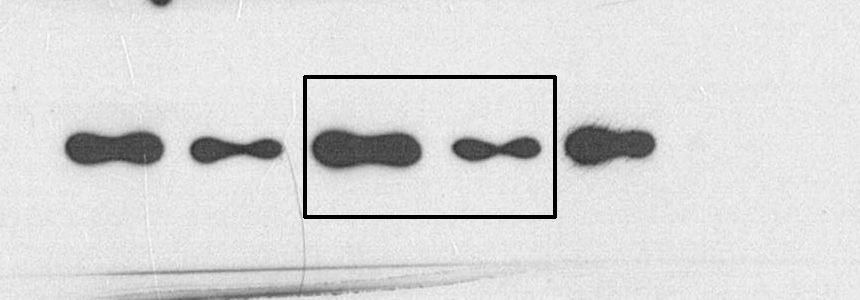

Supplement: Figure 4—source data 1. [file elife-86168-fig4-data1.zip › Figure 4 source data 1/annotated/Fig.4G 20230326 HCT116 transfect with set7 check EZH2 ANti-H3K27me3 uncropped.tif]

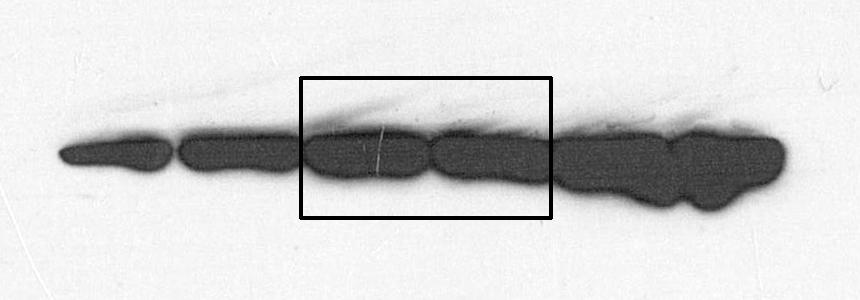

Supplement: Figure 4—source data 1. [file elife-86168-fig4-data1.zip › Figure 4 source data 1/annotated/Fig.4G 20230326 HCT116 transfect with set7 check EZH2 ANti-H3 uncropped.tif]

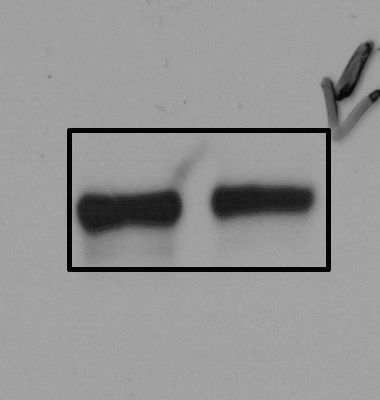

Supplement: Figure 4—source data 1. [file elife-86168-fig4-data1.zip › Figure 4 source data 1/annotated/Fig.4H 20200614 293-set7 l3-ip input anti-ezh2.tif]

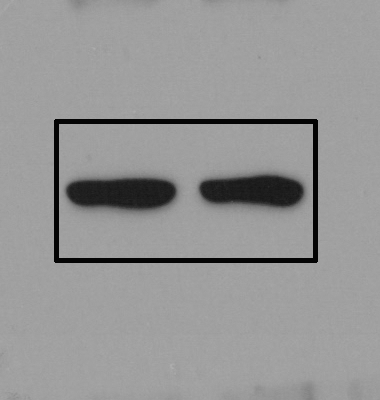

Supplement: Figure 4—source data 1. [file elife-86168-fig4-data1.zip › Figure 4 source data 1/annotated/Fig.4H 20200614 293-set7 l3-ip anti-actin.tif]

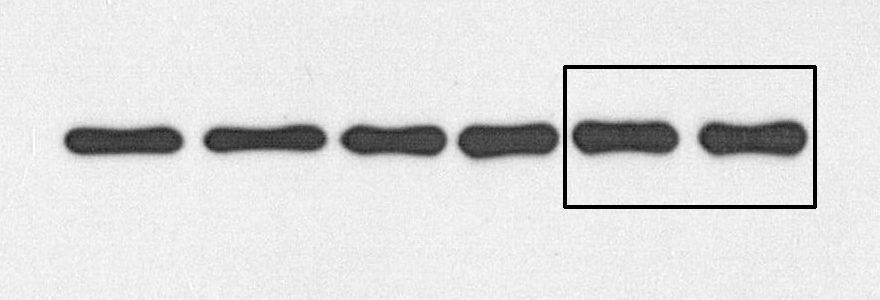

Supplement: Figure 4—source data 1. [file elife-86168-fig4-data1.zip › Figure 4 source data 1/annotated/Fig.4E 202303 Nestin-cre lsd1flox Check EZH2K20me anti-H3 uncropped.tif]

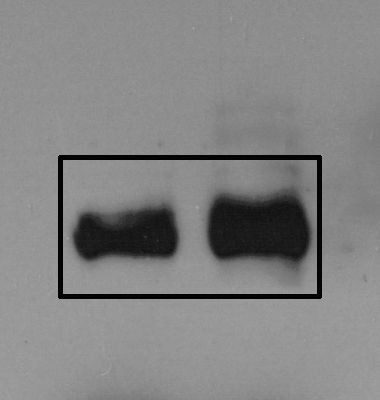

Supplement: Figure 4—source data 1. [file elife-86168-fig4-data1.zip › Figure 4 source data 1/annotated/Fig.4H 20200614 293-set7 l3-ip input anti-l3.tif]

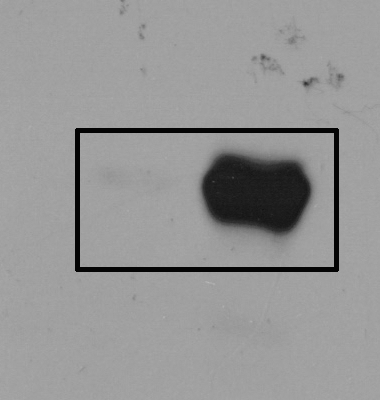

Supplement: Figure 4—source data 1. [file elife-86168-fig4-data1.zip › Figure 4 source data 1/annotated/Fig.4H 20200614 293-set7 l3-ip anti-set7.tif]

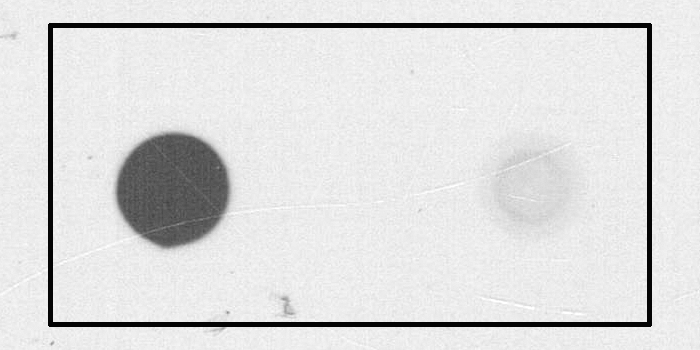

Supplement: Figure 4—source data 1. [file elife-86168-fig4-data1.zip › Figure 4 source data 1/annotated/Fig.4C 20220719 ezh2 gst- lsd1 demethylation anti-K20me uncropped.tif]

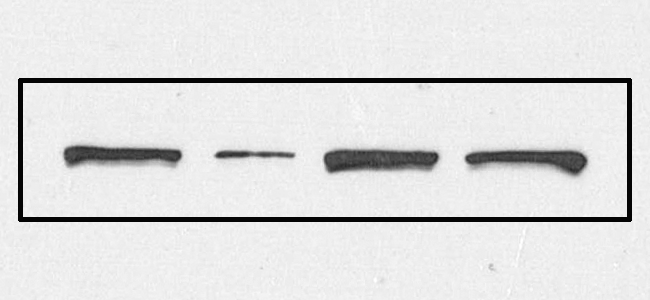

Supplement: Figure 4—source data 1. [file elife-86168-fig4-data1.zip › Figure 4 source data 1/annotated/Fig.4I Western si lsd1 set7 rescue 1 anti-flag-EZH2 uncropped.tif]

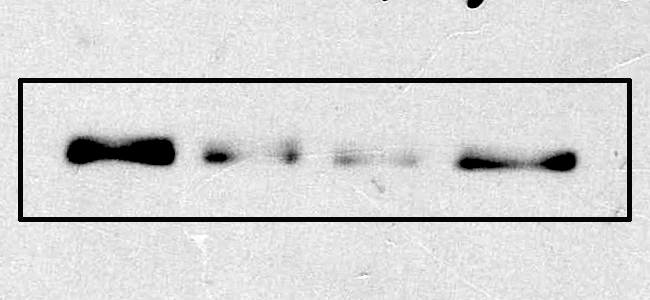

Supplement: Figure 4—source data 1. [file elife-86168-fig4-data1.zip › Figure 4 source data 1/annotated/Fig.4I Western si lsd1 set7 rescue 1 anti-lsd1 uncropped.tif]

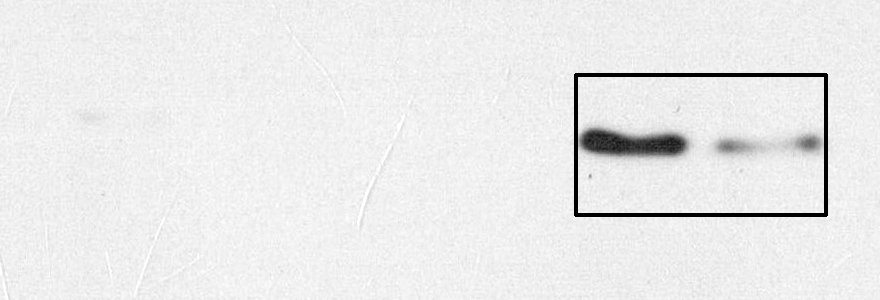

Supplement: Figure 4—source data 1. [file elife-86168-fig4-data1.zip › Figure 4 source data 1/annotated/Fig.4E 202303 Nestin-cre lsd1flox Check EZH2K20me anti-H3K27me3 uncropped.tif]

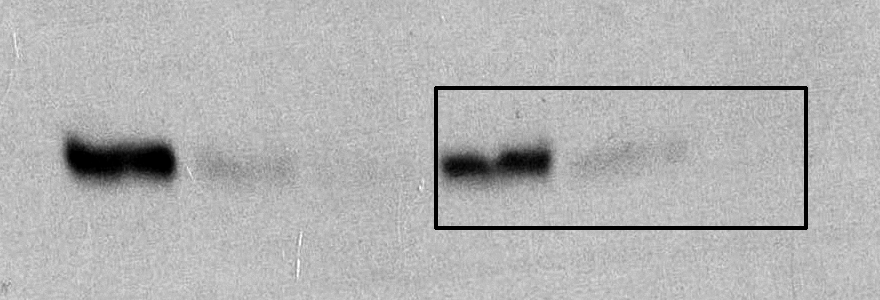

Supplement: Figure 4—source data 1. [file elife-86168-fig4-data1.zip › Figure 4 source data 1/annotated/Fig.4D hct116 si LSD1 -MG132 anti-LSD1 Uncropped.tif]

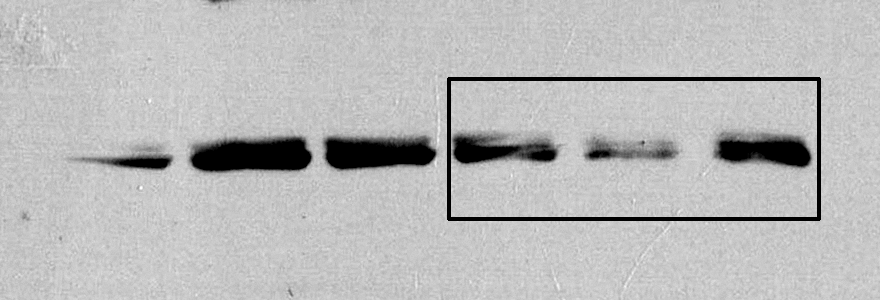

Supplement: Figure 4—source data 1. [file elife-86168-fig4-data1.zip › Figure 4 source data 1/annotated/Fig.4D hct116 si LSD1 -MG132 anti-ezh2 Uncropped.tif]

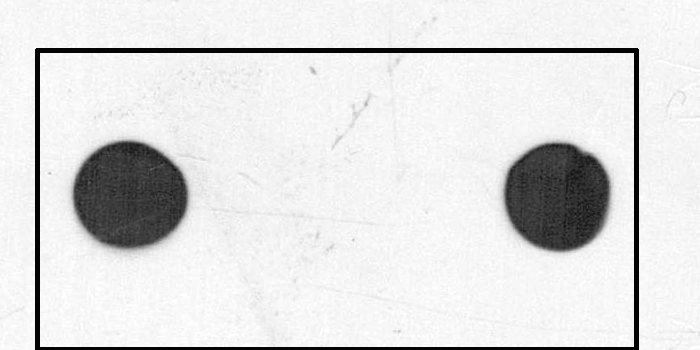

Supplement: Figure 4—source data 1. [file elife-86168-fig4-data1.zip › Figure 4 source data 1/annotated/Fig.4C 20220719 ezh2 gst- lsd1 demethylation anti-EZH2 uncropped.tif]

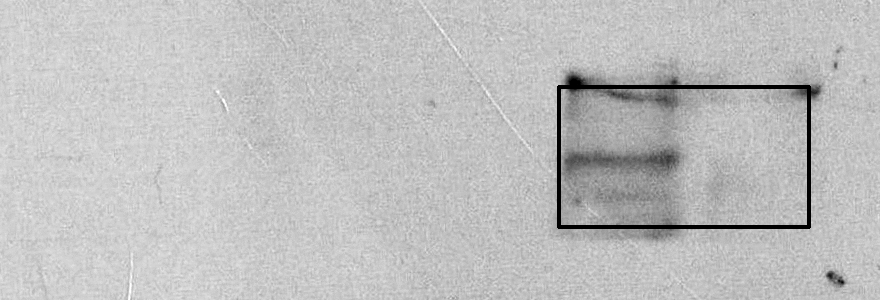

Supplement: Figure 4—source data 1. [file elife-86168-fig4-data1.zip › Figure 4 source data 1/annotated/Fig.4E 202303 Nestin-cre lsd1flox Check EZH2K20me anti-EZH2-K20me uncropped.tif]

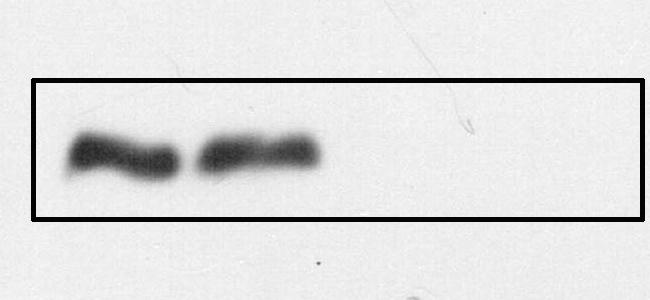

Supplement: Figure 4—source data 1. [file elife-86168-fig4-data1.zip › Figure 4 source data 1/annotated/Fig.4I Western si lsd1 set7 rescue 1 anti-set7 uncropped.tif]

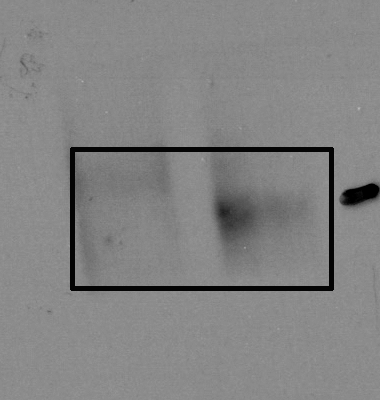

Supplement: Figure 4—source data 1. [file elife-86168-fig4-data1.zip › Figure 4 source data 1/annotated/Fig.4H 20200614 293-set7 l3-ip ip anti-ezh2-k20me.tif]

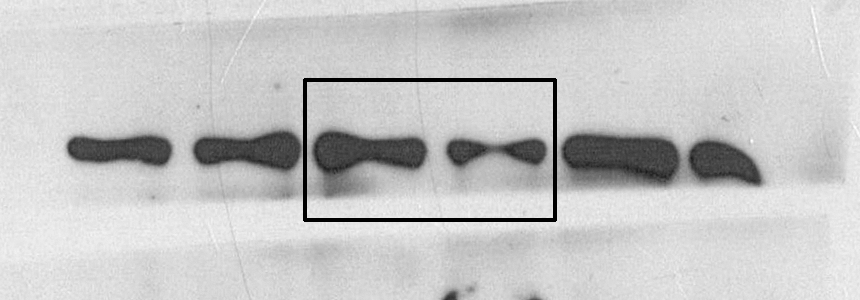

Supplement: Figure 4—source data 1. [file elife-86168-fig4-data1.zip › Figure 4 source data 1/annotated/Fig.4G 20230326 HCT116 transfect with set7 check EZH2 ANti-ezh2 uncropped.tif]

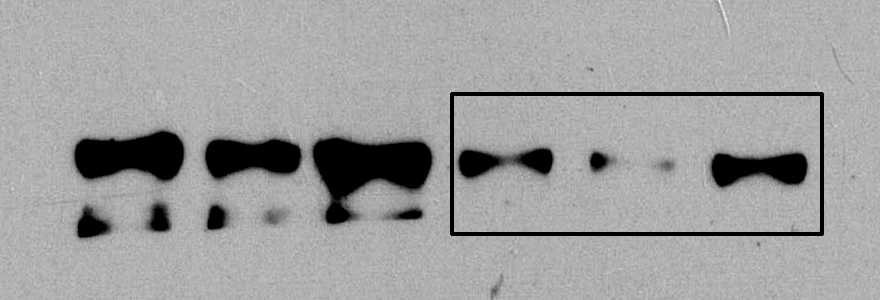

Supplement: Figure 4—source data 1. [file elife-86168-fig4-data1.zip › Figure 4 source data 1/annotated/Fig.4D hct116 si LSD1 -MG132 anti-ezh2K20me Uncropped.tif]

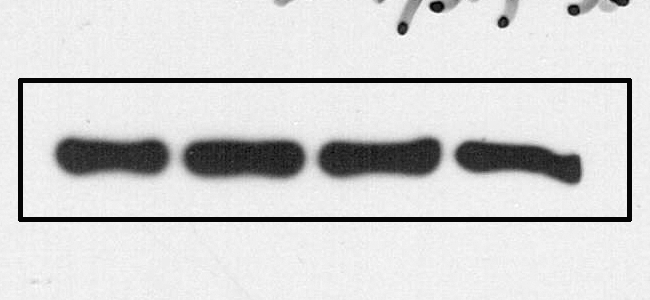

Supplement: Figure 4—source data 1. [file elife-86168-fig4-data1.zip › Figure 4 source data 1/annotated/Fig.4I Western si lsd1 set7 rescue 1 anti-actin uncropped.tif]

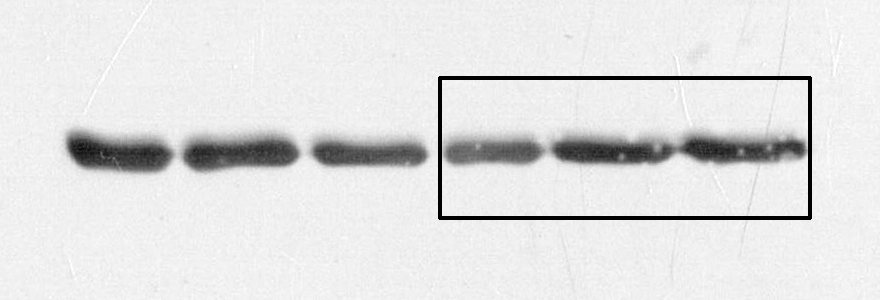

Supplement: Figure 4—source data 1. [file elife-86168-fig4-data1.zip › Figure 4 source data 1/annotated/Fig.4D hct116 si LSD1 -MG132 anti-H3 Uncropped.tif]

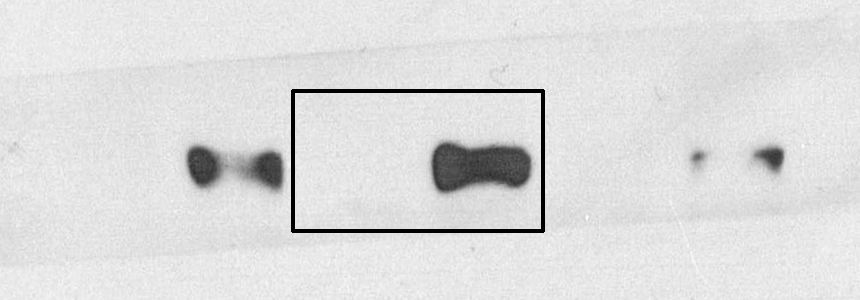

Supplement: Figure 4—source data 1. [file elife-86168-fig4-data1.zip › Figure 4 source data 1/annotated/Fig.4G 20230326 HCT116 transfect with set7 check EZH2 ANti-set7 uncropped.tif]

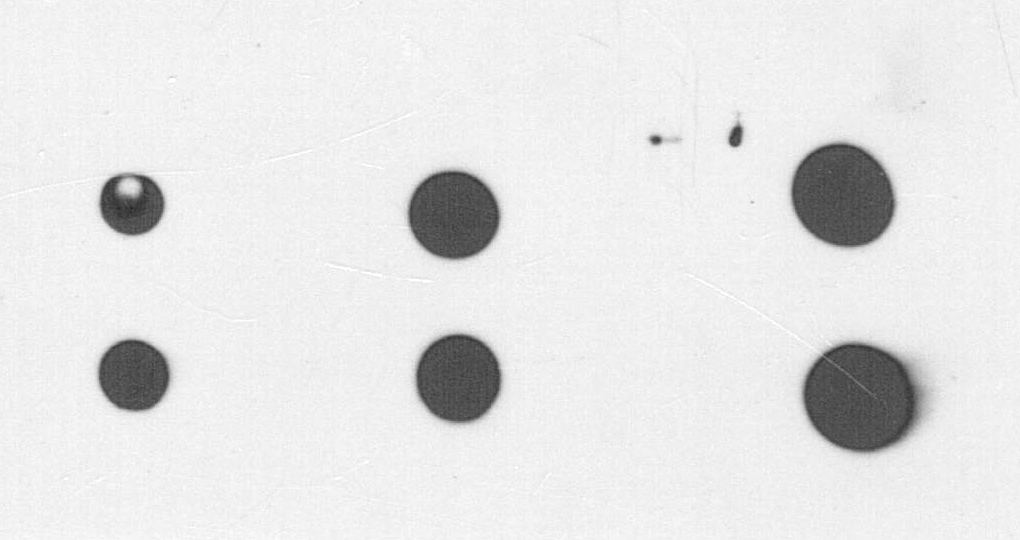

Supplement: Figure 4—figure supplement 1—source data 1. [file elife-86168-fig4-figsupp1-data1.zip › Figure 4-figure supplement 1 source data 1/20220722 ezh2 k20me peptide antibody test anti-EZH2 Cropped.tif]

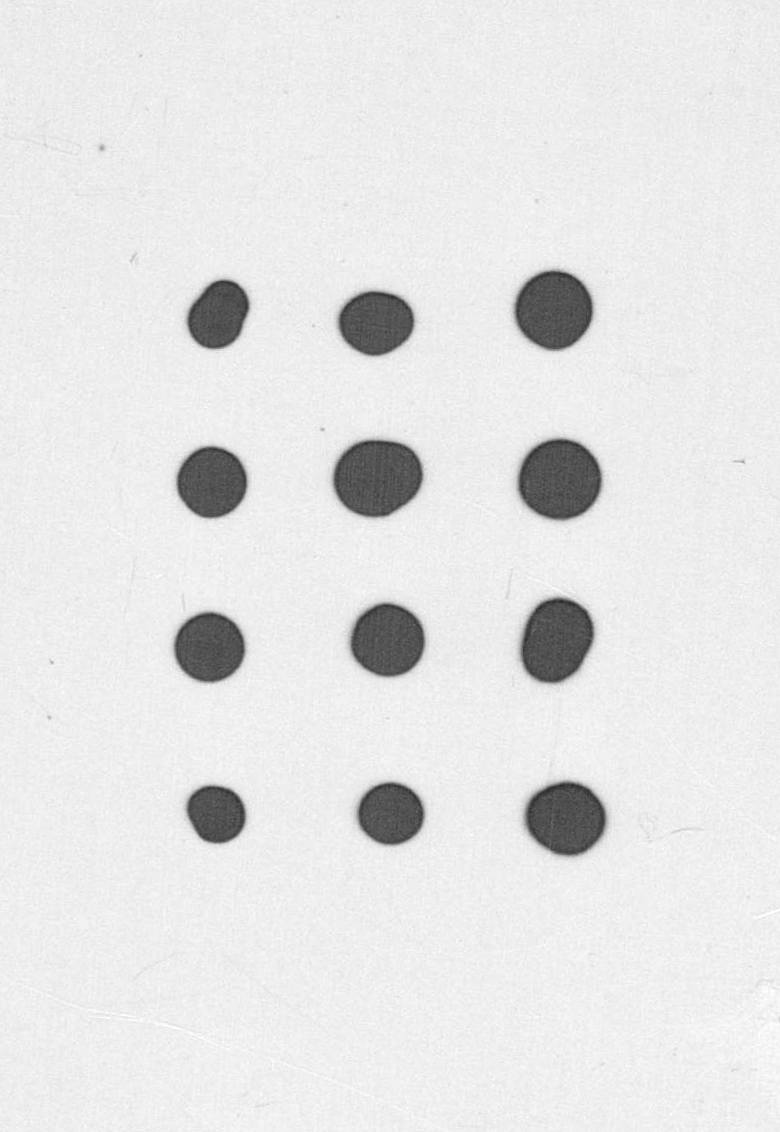

Supplement: Figure 4—figure supplement 1—source data 1. [file elife-86168-fig4-figsupp1-data1.zip › Figure 4-figure supplement 1 source data 1/dot blot EZH2 for k20me2 k20me3 k17me anti-EZH2 uncropped.tif]

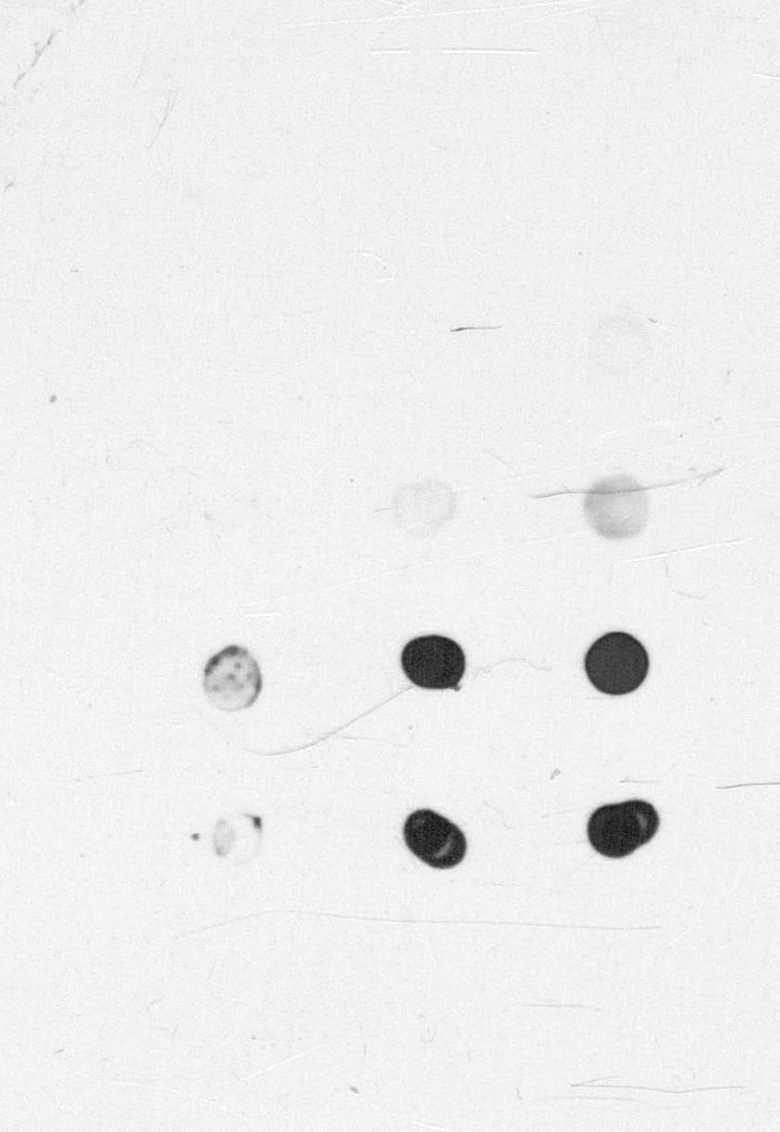

Supplement: Figure 4—figure supplement 1—source data 1. [file elife-86168-fig4-figsupp1-data1.zip › Figure 4-figure supplement 1 source data 1/dot blot EZH2 for k20me2 k20me3 k17me anti-EZH2-k20me uncropped.tif]

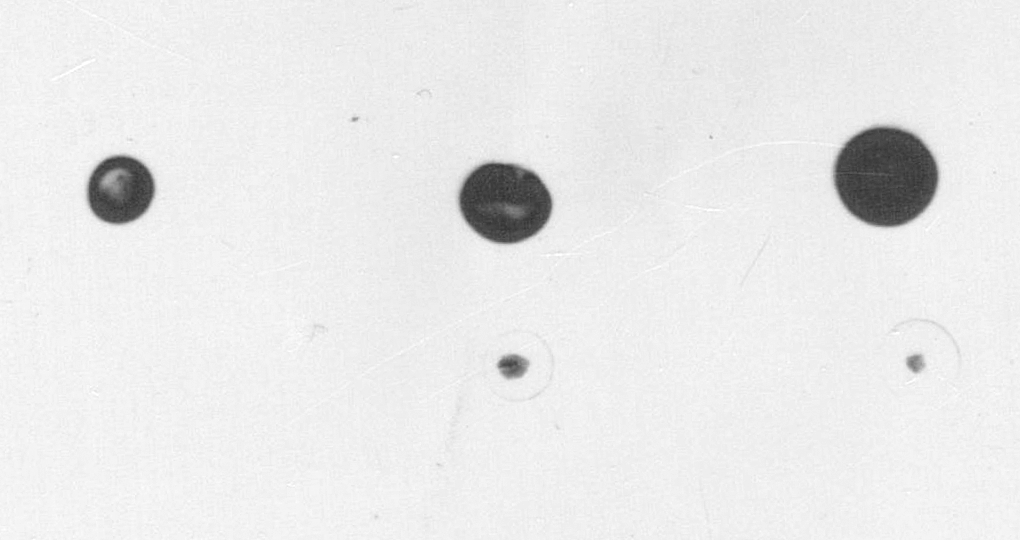

Supplement: Figure 4—figure supplement 1—source data 1. [file elife-86168-fig4-figsupp1-data1.zip › Figure 4-figure supplement 1 source data 1/20220722 ezh2 k20me peptide antibody test anti-EZH2-k20me Cropped.tif]

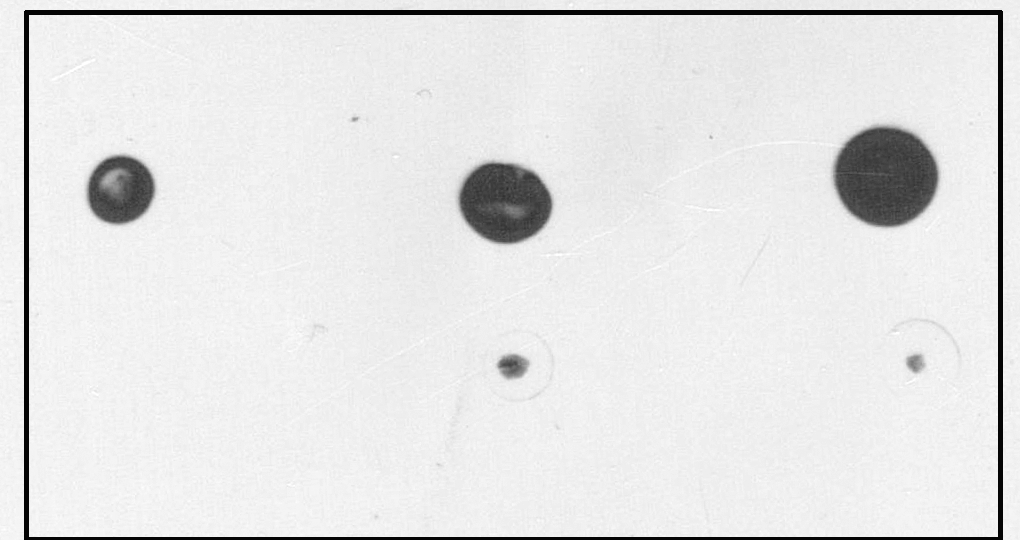

Supplement: Figure 4—figure supplement 1—source data 1. [file elife-86168-fig4-figsupp1-data1.zip › Figure 4-figure supplement 1 source data 1/annotated/Figure supplement1 ezh2 k20me peptide antibody test anti-EZH2-k20me unCropped.tif]

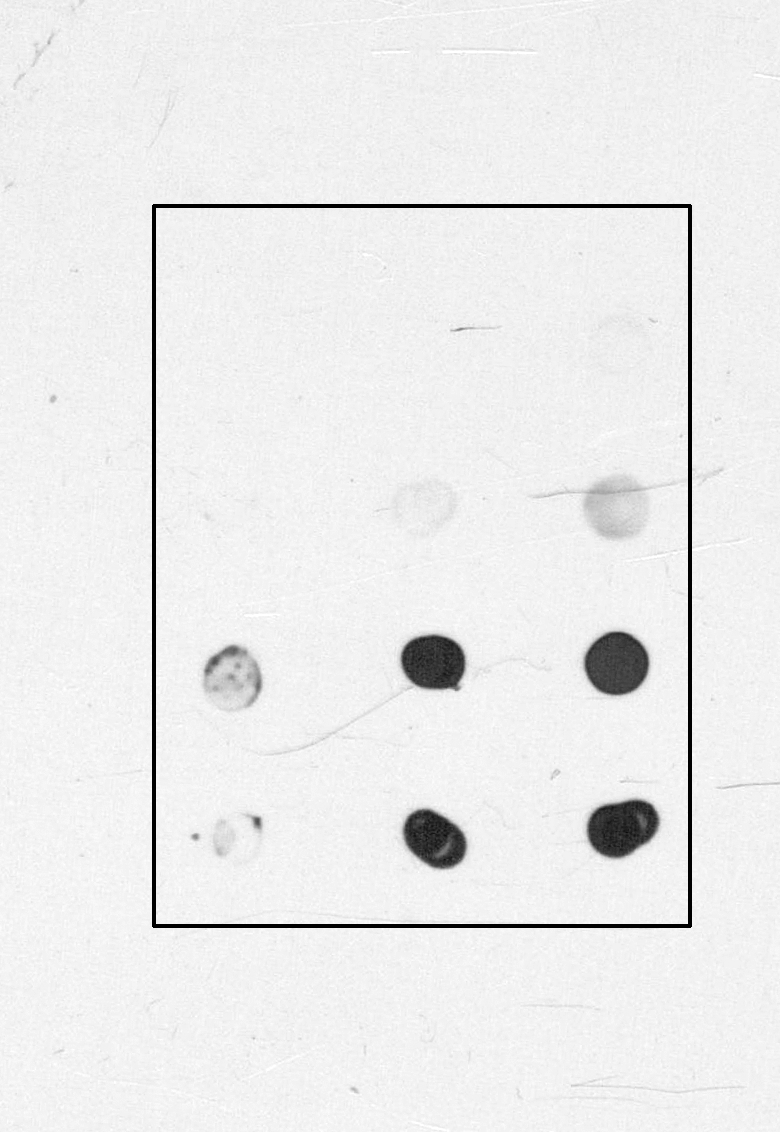

Supplement: Figure 4—figure supplement 1—source data 1. [file elife-86168-fig4-figsupp1-data1.zip › Figure 4-figure supplement 1 source data 1/annotated/dot blot EZH2 for k20me2 k20me3 k17me anti-EZH2-k20me uncropped.tif]

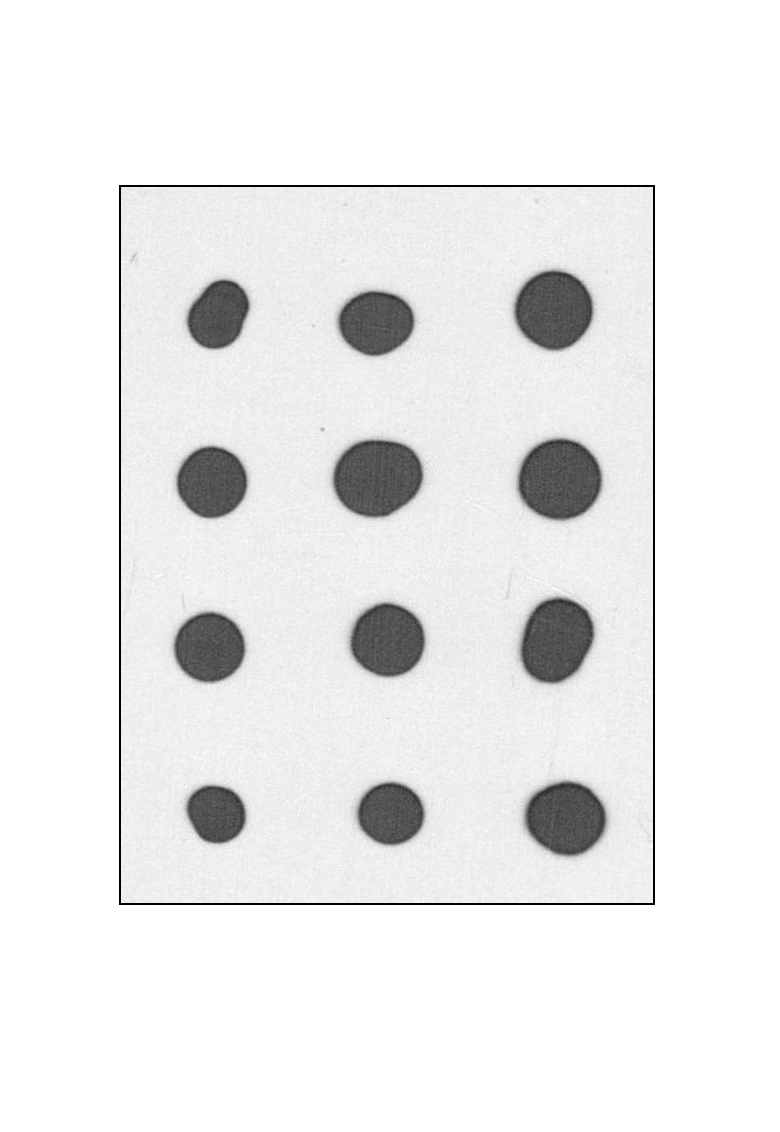

Supplement: Figure 4—figure supplement 1—source data 1. [file elife-86168-fig4-figsupp1-data1.zip › Figure 4-figure supplement 1 source data 1/annotated/dot blot EZH2 for k20me2 k20me3 k17me anti-EZH2 uncropped.tif]

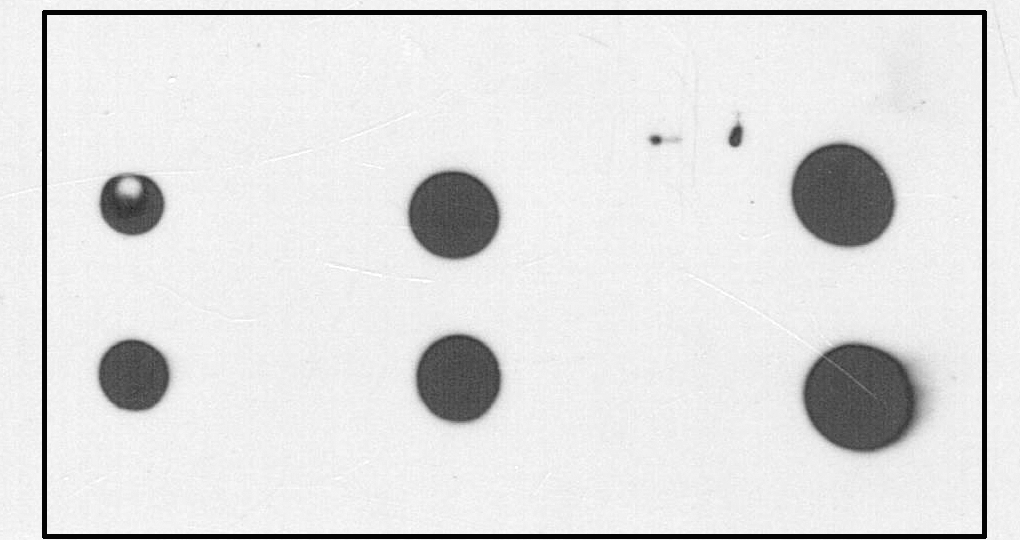

Supplement: Figure 4—figure supplement 1—source data 1. [file elife-86168-fig4-figsupp1-data1.zip › Figure 4-figure supplement 1 source data 1/annotated/Figure supplement1 ezh2 k20me peptide antibody test anti-EZH2 unCropped.tif]

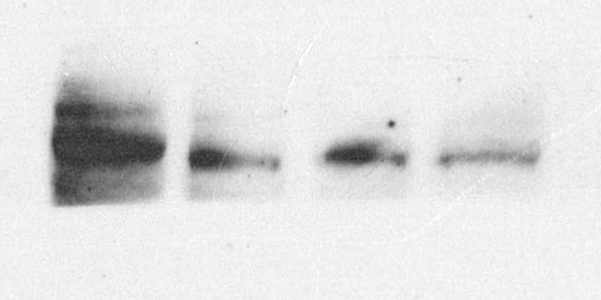

Supplement: Figure 4—figure supplement 2—source data 1. [file elife-86168-fig4-figsupp2-data1.zip › Figure 4-figure supplement 2 source data 1/Figure 4-figure supplement 2A Hct116 SI set7 check ezh2 anti-EZH2K20me Uncropped.tif]

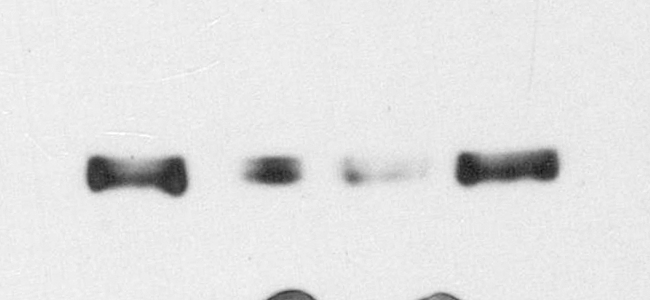

Supplement: Figure 4—figure supplement 2—source data 1. [file elife-86168-fig4-figsupp2-data1.zip › Figure 4-figure supplement 2 source data 1/Figure 4-figure supplement 2C HA-EZH2 si phf20l1 l3 anti-phf20l1 uncropped 2.tif]

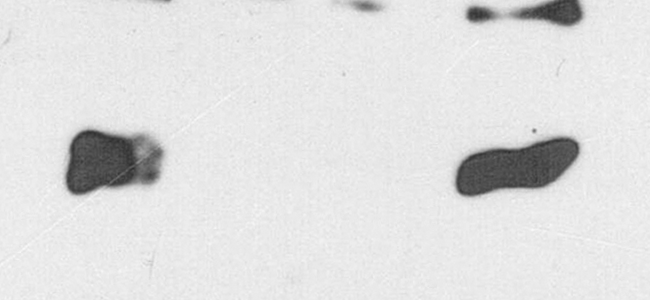

Supplement: Figure 4—figure supplement 2—source data 1. [file elife-86168-fig4-figsupp2-data1.zip › Figure 4-figure supplement 2 source data 1/Figure 4-figure supplement 2B HA-EZH2 si phf20l1 l3 anti-phf20l1 uncropped 3.tif]

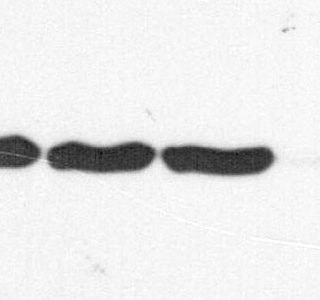

Supplement: Figure 4—figure supplement 2—source data 1. [file elife-86168-fig4-figsupp2-data1.zip › Figure 4-figure supplement 2 source data 1/Figure 4-figure supplement 2D 20220713 T47D +set7 check h3k27me3 ezh2 k20me s21p anti-actin uncropped.tif]

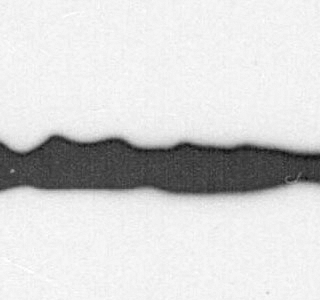

Supplement: Figure 4—figure supplement 2—source data 1. [file elife-86168-fig4-figsupp2-data1.zip › Figure 4-figure supplement 2 source data 1/Figure 4-figure supplement 2D 20220713 T47D +set7 check h3k27me3 ezh2 k20me s21p anti-H3 uncropped.tif]

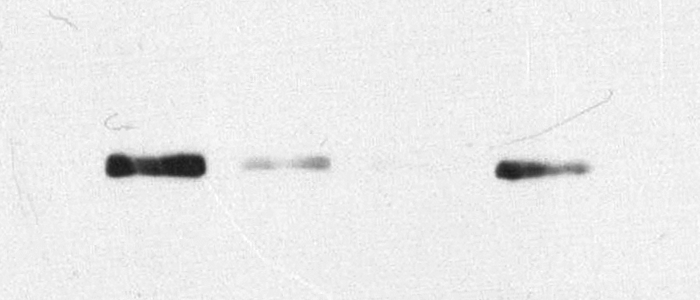

Supplement: Figure 4—figure supplement 2—source data 1. [file elife-86168-fig4-figsupp2-data1.zip › Figure 4-figure supplement 2 source data 1/Figure 4-figure supplement 2B Western si lsd1 SET7 #2 anti-LSD1 rep2 uncropped.tif]

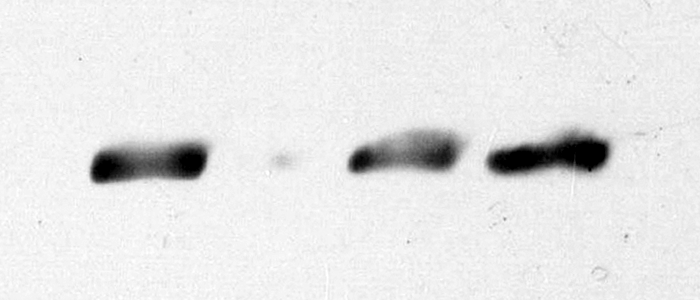

Supplement: Figure 4—figure supplement 2—source data 1. [file elife-86168-fig4-figsupp2-data1.zip › Figure 4-figure supplement 2 source data 1/Figure 4-figure supplement 2B Western si lsd1 SET7 #2 anti-flag-EZH2 rep2 uncropped.tif]

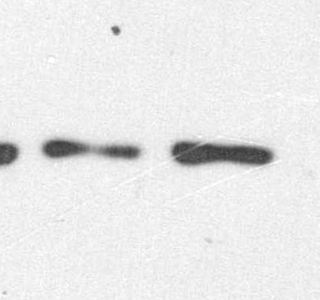

Supplement: Figure 4—figure supplement 2—source data 1. [file elife-86168-fig4-figsupp2-data1.zip › Figure 4-figure supplement 2 source data 1/Figure 4-figure supplement 2D20220713 T47D +set7 check h3k27me3 ezh2 k20me s21p anti-EZH2-K20me 3 uncropped.tif]

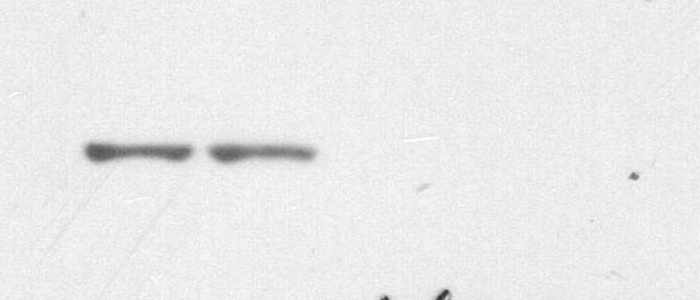

Supplement: Figure 4—figure supplement 2—source data 1. [file elife-86168-fig4-figsupp2-data1.zip › Figure 4-figure supplement 2 source data 1/Figure 4-figure supplement 2B Western si lsd1 SET7 #2 anti-set7 rep2 uncropped.tif]

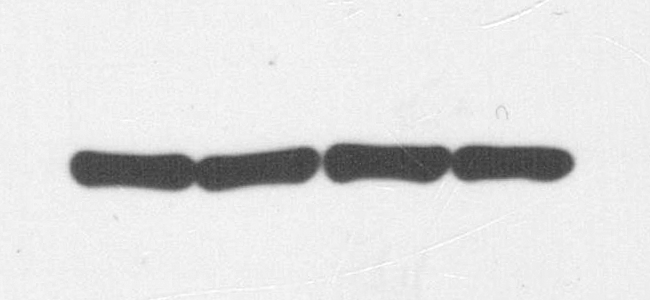

Supplement: Figure 4—figure supplement 2—source data 1. [file elife-86168-fig4-figsupp2-data1.zip › Figure 4-figure supplement 2 source data 1/Figure 4-figure supplement 2C HA-EZH2 si phf20l1 l3 anti-ACTIN uncropped 2.tif]

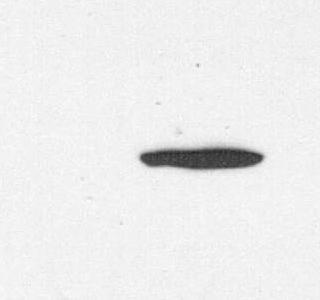

Supplement: Figure 4—figure supplement 2—source data 1. [file elife-86168-fig4-figsupp2-data1.zip › Figure 4-figure supplement 2 source data 1/Figure 4-figure supplement 2D 20220713 T47D +set7 check h3k27me3 ezh2 k20me s21p anti-SET7 uncropped.tif]

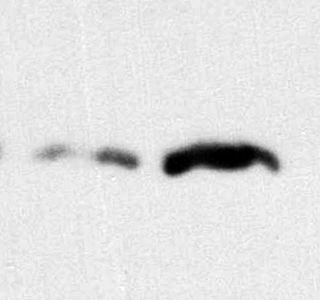

Supplement: Figure 4—figure supplement 2—source data 1. [file elife-86168-fig4-figsupp2-data1.zip › Figure 4-figure supplement 2 source data 1/Figure 4-figure supplement 2D 20220713 T47D +set7 check h3k27me3 ezh2 k20me s21p anti-H3K27me3 uncropped.tif]

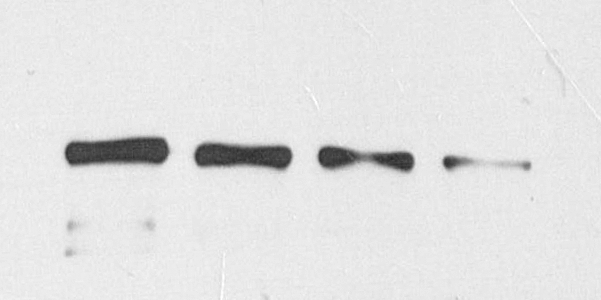

Supplement: Figure 4—figure supplement 2—source data 1. [file elife-86168-fig4-figsupp2-data1.zip › Figure 4-figure supplement 2 source data 1/Figure 4-figure supplement 2A Hct116 SI set7 check ezh2 anti-EZH2 Uncropped.tif]

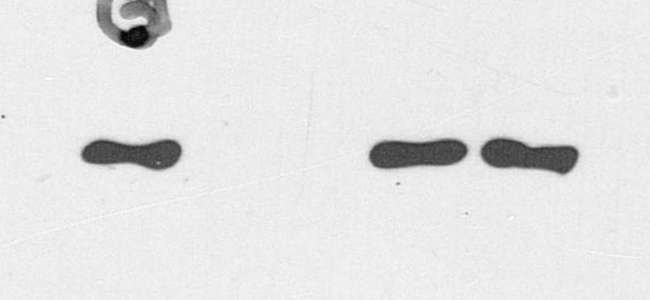

Supplement: Figure 4—figure supplement 2—source data 1. [file elife-86168-fig4-figsupp2-data1.zip › Figure 4-figure supplement 2 source data 1/Figure 4-figure supplement 2B HA-EZH2 si phf20l1 l3 anti-HA-EZH2 uncropped 3.tif]

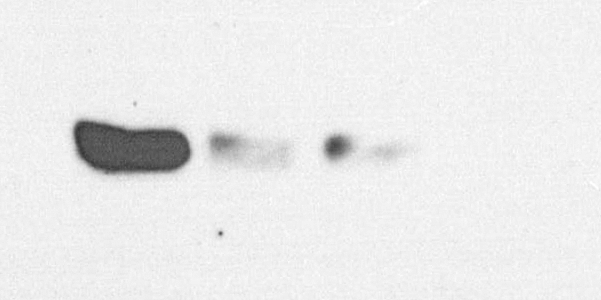

Supplement: Figure 4—figure supplement 2—source data 1. [file elife-86168-fig4-figsupp2-data1.zip › Figure 4-figure supplement 2 source data 1/Figure 4-figure supplement 2A Hct116 SI set7 check ezh2 anti-SET7 Uncropped.tif]

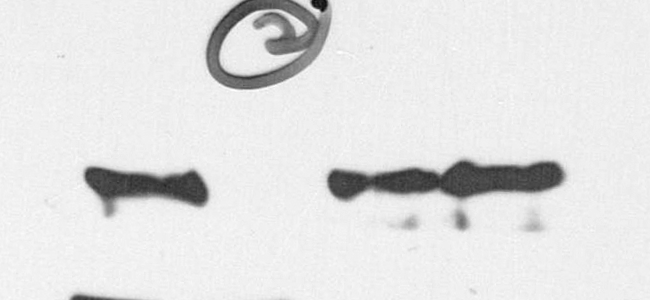

Supplement: Figure 4—figure supplement 2—source data 1. [file elife-86168-fig4-figsupp2-data1.zip › Figure 4-figure supplement 2 source data 1/Figure 4-figure supplement 2C HA-EZH2 si phf20l1 l3 anti-HA-EZH2 uncropped 2.tif]

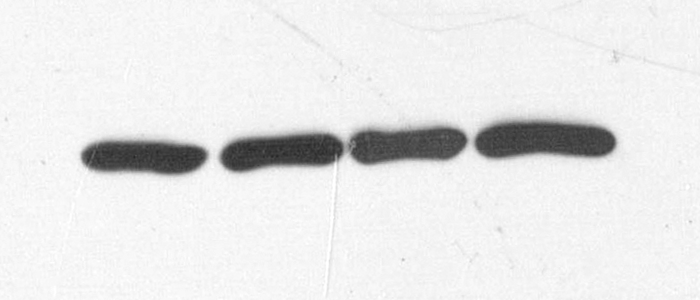

Supplement: Figure 4—figure supplement 2—source data 1. [file elife-86168-fig4-figsupp2-data1.zip › Figure 4-figure supplement 2 source data 1/Figure 4-figure supplement 2B Western si lsd1 SET7 #2 anti-Actin rep2 uncropped.tif]

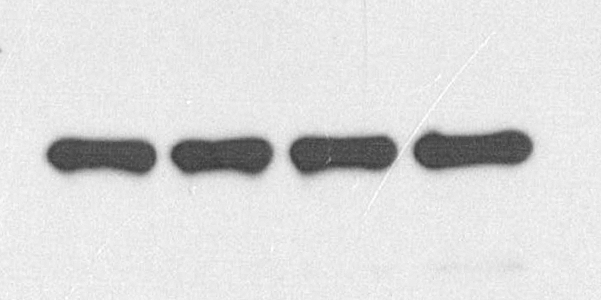

Supplement: Figure 4—figure supplement 2—source data 1. [file elife-86168-fig4-figsupp2-data1.zip › Figure 4-figure supplement 2 source data 1/Figure 4-figure supplement 2A Hct116 SI set7 check ezh2 anti-gapdH Uncropped.tif]

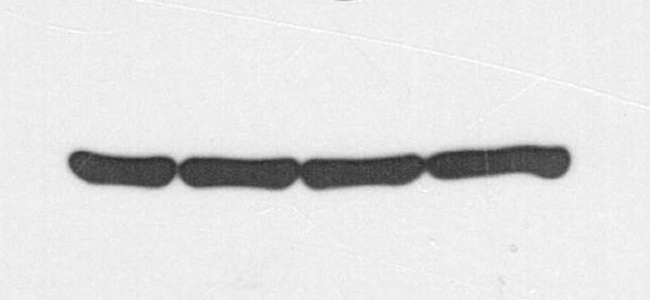

Supplement: Figure 4—figure supplement 2—source data 1. [file elife-86168-fig4-figsupp2-data1.zip › Figure 4-figure supplement 2 source data 1/Figure 4-figure supplement 2B HA-EZH2 si phf20l1 l3 anti-ACTIN uncropped 3.tif]

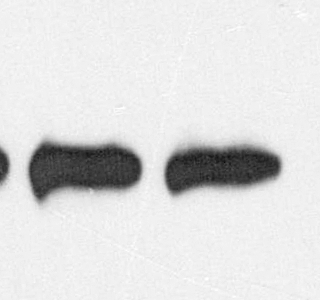

Supplement: Figure 4—figure supplement 2—source data 1. [file elife-86168-fig4-figsupp2-data1.zip › Figure 4-figure supplement 2 source data 1/Figure 4-figure supplement 2D 20220713 T47D +set7 check h3k27me3 ezh2 k20me s21p anti-EZH2 uncropped.tif]

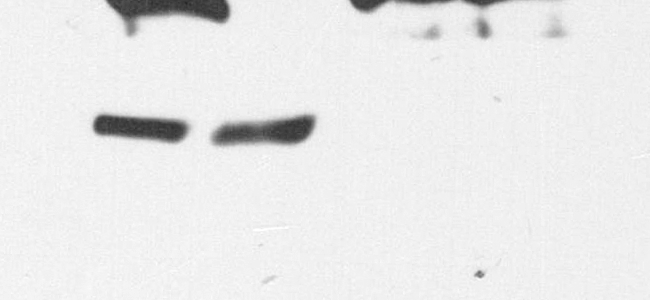

Supplement: Figure 4—figure supplement 2—source data 1. [file elife-86168-fig4-figsupp2-data1.zip › Figure 4-figure supplement 2 source data 1/Figure 4-figure supplement 2C HA-EZH2 si phf20l1 l3 anti-L3MBTL3 uncropped 2.tif]

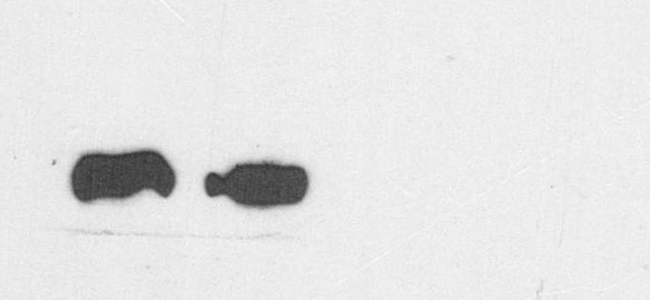

Supplement: Figure 4—figure supplement 2—source data 1. [file elife-86168-fig4-figsupp2-data1.zip › Figure 4-figure supplement 2 source data 1/Figure 4-figure supplement 2B HA-EZH2 si phf20l1 l3 anti-L3MBTL3 uncropped 3.tif]

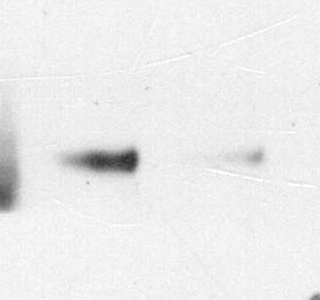

Supplement: Figure 4—figure supplement 2—source data 1. [file elife-86168-fig4-figsupp2-data1.zip › Figure 4-figure supplement 2 source data 1/Figure 4-figure supplement 2D 20220713 T47D +set7 check h3k27me3 ezh2 k20me s21p anti-EZH2-S21p uncropped.tif]

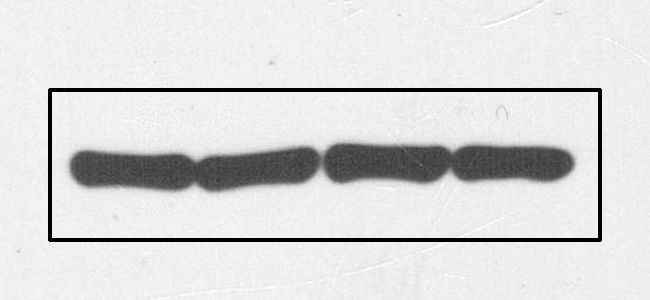

Supplement: Figure 4—figure supplement 2—source data 1. [file elife-86168-fig4-figsupp2-data1.zip › Figure 4-figure supplement 2 source data 1/annotated/Figure 4-figure supplement 2C HA-EZH2 si phf20l1 l3 anti-ACTIN uncropped 2.tif]

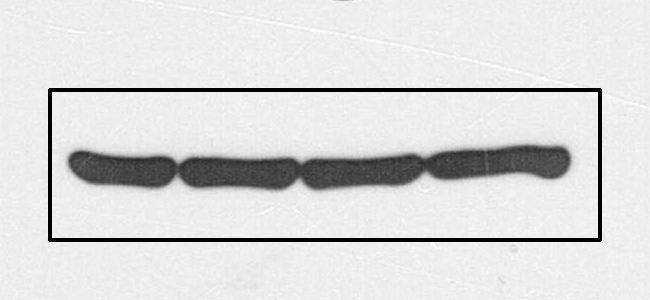

Supplement: Figure 4—figure supplement 2—source data 1. [file elife-86168-fig4-figsupp2-data1.zip › Figure 4-figure supplement 2 source data 1/annotated/Figure 4-figure supplement 2B HA-EZH2 si phf20l1 l3 anti-ACTIN uncropped 3.tif]

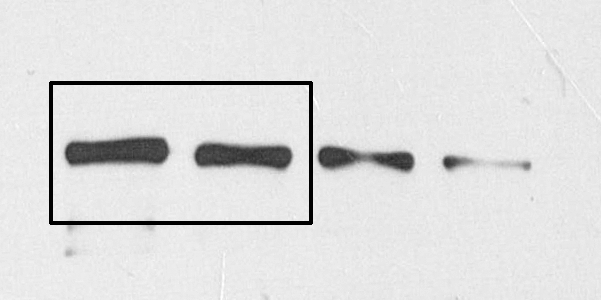

Supplement: Figure 4—figure supplement 2—source data 1. [file elife-86168-fig4-figsupp2-data1.zip › Figure 4-figure supplement 2 source data 1/annotated/Figure 4-figure supplement 2A Hct116 SI set7 check ezh2 anti-EZH2 Uncropped.tif]

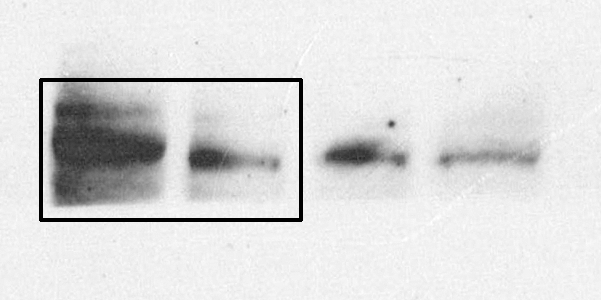

Supplement: Figure 4—figure supplement 2—source data 1. [file elife-86168-fig4-figsupp2-data1.zip › Figure 4-figure supplement 2 source data 1/annotated/Figure 4-figure supplement 2A Hct116 SI set7 check ezh2 anti-EZH2K20me Uncropped.tif]

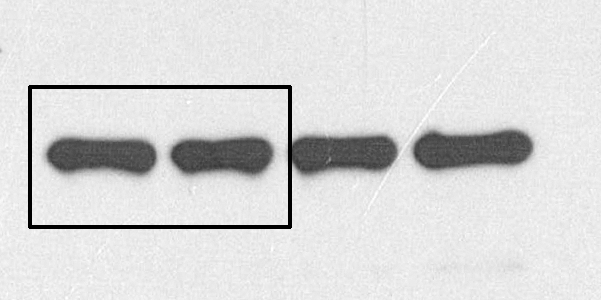

Supplement: Figure 4—figure supplement 2—source data 1. [file elife-86168-fig4-figsupp2-data1.zip › Figure 4-figure supplement 2 source data 1/annotated/Figure 4-figure supplement 2A Hct116 SI set7 check ezh2 anti-gapdH Uncropped.tif]

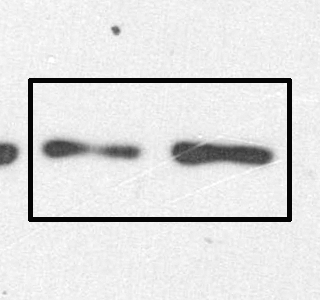

Supplement: Figure 4—figure supplement 2—source data 1. [file elife-86168-fig4-figsupp2-data1.zip › Figure 4-figure supplement 2 source data 1/annotated/Figure 4-figure supplement 2D20220713 T47D +set7 check h3k27me3 ezh2 k20me s21p anti-EZH2-K20me 3 uncropped.tif]

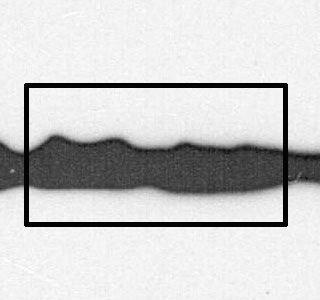

Supplement: Figure 4—figure supplement 2—source data 1. [file elife-86168-fig4-figsupp2-data1.zip › Figure 4-figure supplement 2 source data 1/annotated/Figure 4-figure supplement 2D20220713 T47D +set7 check h3k27me3 ezh2 k20me s21p anti-H3 uncropped.tif]

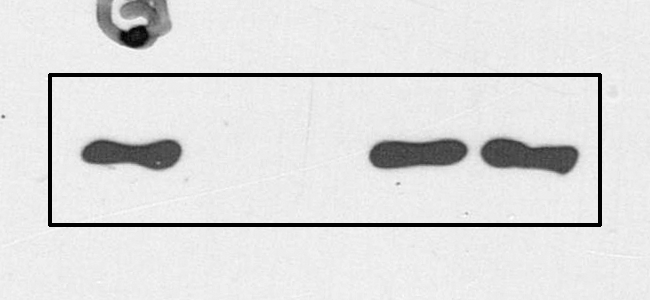

Supplement: Figure 4—figure supplement 2—source data 1. [file elife-86168-fig4-figsupp2-data1.zip › Figure 4-figure supplement 2 source data 1/annotated/Figure 4-figure supplement 2B HA-EZH2 si phf20l1 l3 anti-HA-EZH2 uncropped 3.tif]

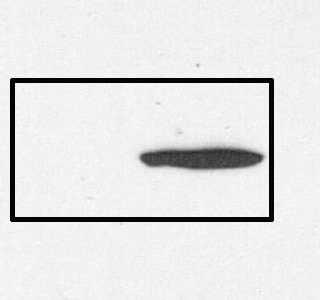

Supplement: Figure 4—figure supplement 2—source data 1. [file elife-86168-fig4-figsupp2-data1.zip › Figure 4-figure supplement 2 source data 1/annotated/Figure 4-figure supplement 2D 20220713 T47D +set7 check h3k27me3 ezh2 k20me s21p anti-SET7 uncropped.tif]

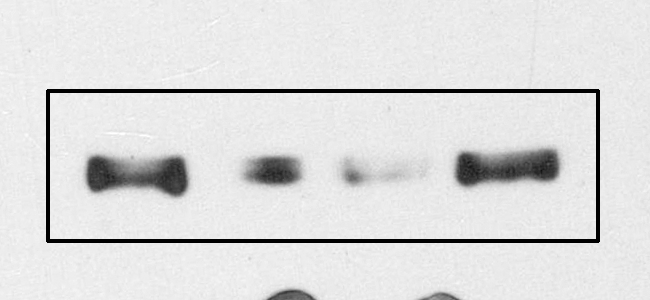

Supplement: Figure 4—figure supplement 2—source data 1. [file elife-86168-fig4-figsupp2-data1.zip › Figure 4-figure supplement 2 source data 1/annotated/Figure 4-figure supplement 2C HA-EZH2 si phf20l1 l3 anti-phf20l1 uncropped 2.tif]

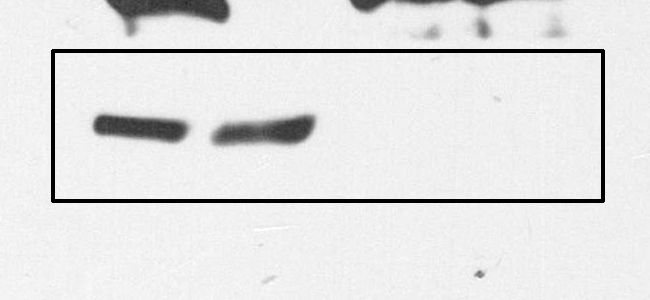

Supplement: Figure 4—figure supplement 2—source data 1. [file elife-86168-fig4-figsupp2-data1.zip › Figure 4-figure supplement 2 source data 1/annotated/Figure 4-figure supplement 2C HA-EZH2 si phf20l1 l3 anti-L3MBTL3 uncropped 2.tif]

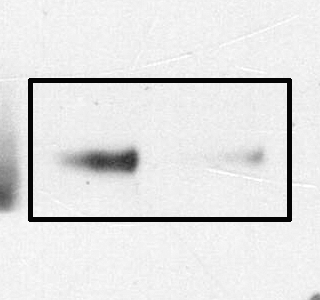

Supplement: Figure 4—figure supplement 2—source data 1. [file elife-86168-fig4-figsupp2-data1.zip › Figure 4-figure supplement 2 source data 1/annotated/Figure 4-figure supplement 2D20220713 T47D +set7 check h3k27me3 ezh2 k20me s21p anti-EZH2-S21p uncropped.tif]

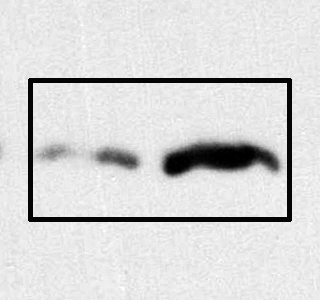

Supplement: Figure 4—figure supplement 2—source data 1. [file elife-86168-fig4-figsupp2-data1.zip › Figure 4-figure supplement 2 source data 1/annotated/Figure 4-figure supplement 2D 20220713 T47D +set7 check h3k27me3 ezh2 k20me s21p anti-H3K27me3 uncropped.tif]

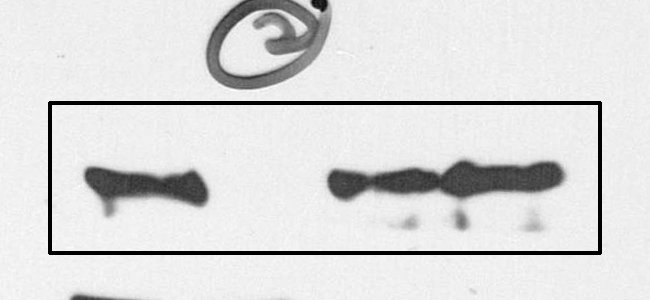

Supplement: Figure 4—figure supplement 2—source data 1. [file elife-86168-fig4-figsupp2-data1.zip › Figure 4-figure supplement 2 source data 1/annotated/Figure 4-figure supplement 2C HA-EZH2 si phf20l1 l3 anti-HA-EZH2 uncropped 2.tif]

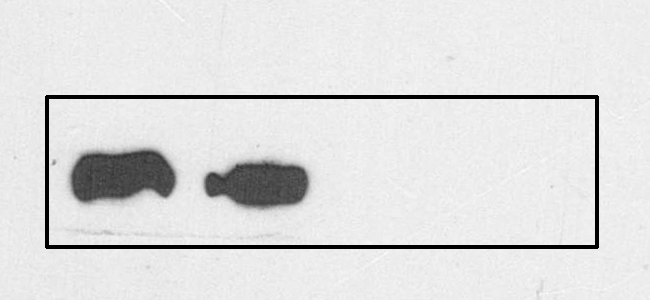

Supplement: Figure 4—figure supplement 2—source data 1. [file elife-86168-fig4-figsupp2-data1.zip › Figure 4-figure supplement 2 source data 1/annotated/Figure 4-figure supplement 2B HA-EZH2 si phf20l1 l3 anti-L3MBTL3 uncropped 3.tif]

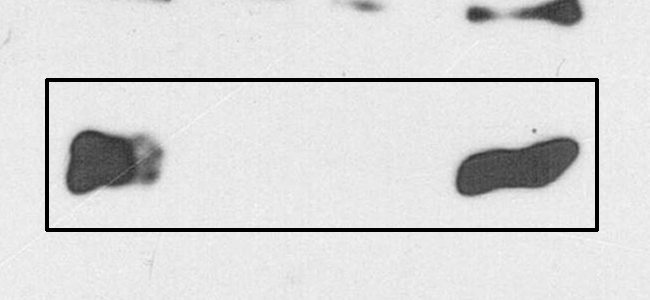

Supplement: Figure 4—figure supplement 2—source data 1. [file elife-86168-fig4-figsupp2-data1.zip › Figure 4-figure supplement 2 source data 1/annotated/Figure 4-figure supplement 2B HA-EZH2 si phf20l1 l3 anti-phf20l1 uncropped 3.tif]

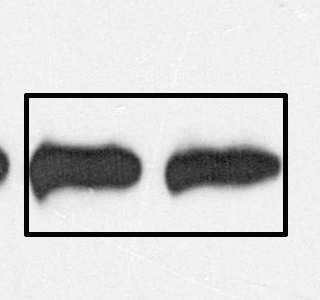

Supplement: Figure 4—figure supplement 2—source data 1. [file elife-86168-fig4-figsupp2-data1.zip › Figure 4-figure supplement 2 source data 1/annotated/Figure 4-figure supplement 2D 20220713 T47D +set7 check h3k27me3 ezh2 k20me s21p anti-EZH2 uncropped.tif]

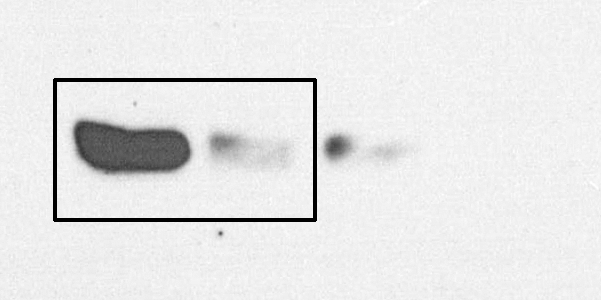

Supplement: Figure 4—figure supplement 2—source data 1. [file elife-86168-fig4-figsupp2-data1.zip › Figure 4-figure supplement 2 source data 1/annotated/Figure 4-figure supplement 2A Hct116 SI set7 check ezh2 anti-SET7 Uncropped.tif]

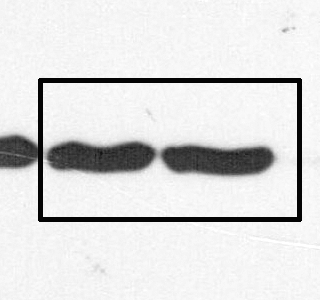

Supplement: Figure 4—figure supplement 2—source data 1. [file elife-86168-fig4-figsupp2-data1.zip › Figure 4-figure supplement 2 source data 1/annotated/Figure 4-figure supplement 2D 20220713 T47D +set7 check h3k27me3 ezh2 k20me s21p anti-actin uncropped.tif]

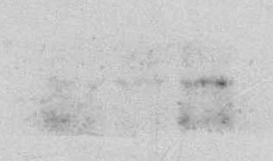

Supplement: Figure 5—source data 1. [file elife-86168-fig5-data1.zip › Figure 5 source data 1/Fig.5A mouse brain e14 e18 p0 check EZH2 K20me anti-ezh2-s21p uncropped.tif]

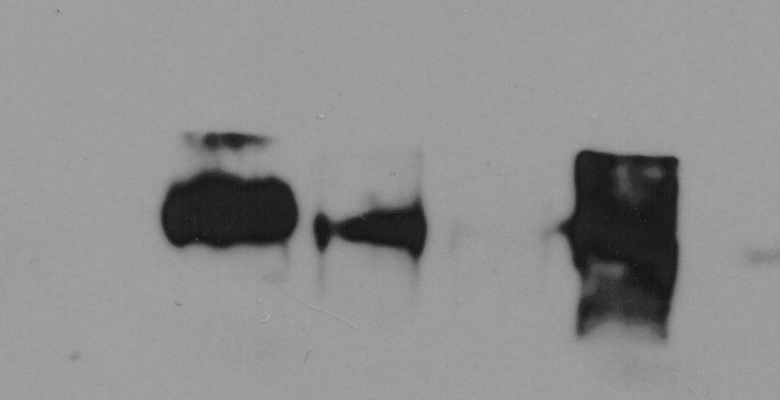

Supplement: Figure 5—source data 1. [file elife-86168-fig5-data1.zip › Figure 5 source data 1/Fig.5C 20200306 EZH2 ip anti-EZH2.tif]

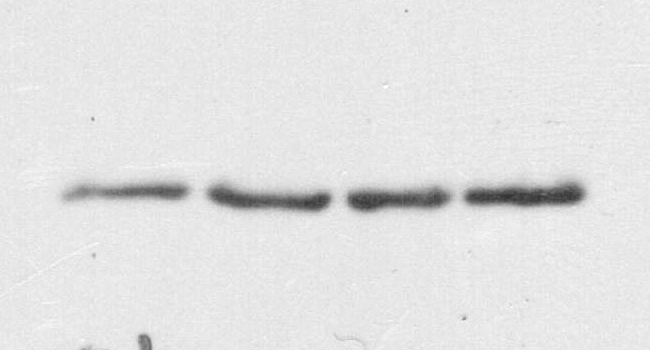

Supplement: Figure 5—source data 1. [file elife-86168-fig5-data1.zip › Figure 5 source data 1/Fig.5D 20220502 293t l3 ip with ezh2 transfect with set7 wt h297a input anti-l3 uncropped.tif]

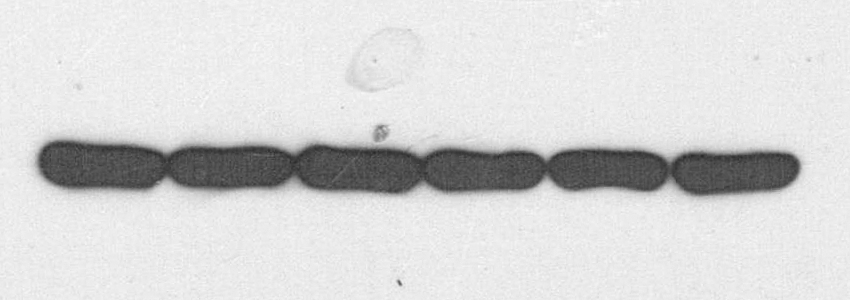

Supplement: Figure 5—source data 1. [file elife-86168-fig5-data1.zip › Figure 5 source data 1/Fig.5F 20220614 G401 EZH2 WT K20R S21A SI LSD1-2 Anti-actin Uncropped.tif]

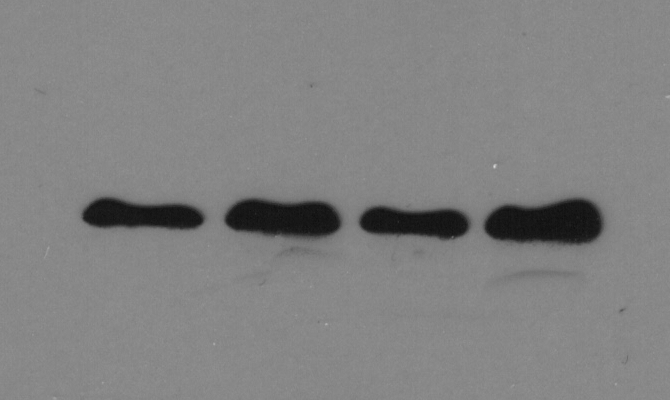

Supplement: Figure 5—source data 1. [file elife-86168-fig5-data1.zip › Figure 5 source data 1/Fig.5H ezh2 wt k20r ub input anti-SET7.tif]

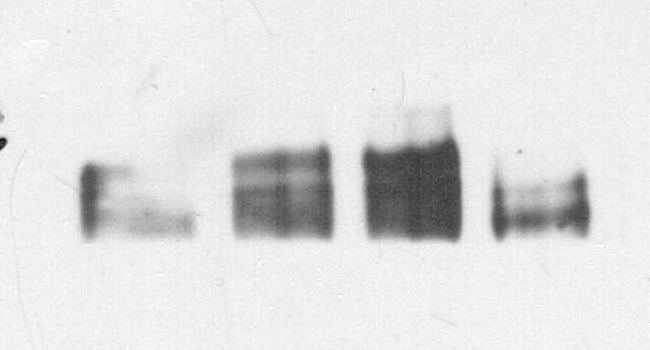

Supplement: Figure 5—source data 1. [file elife-86168-fig5-data1.zip › Figure 5 source data 1/Fig.5D 20220502 293t l3 ip with ezh2 transfect with set7 wt h297a iP-L3 anti-EZH2 uncropped.tif]

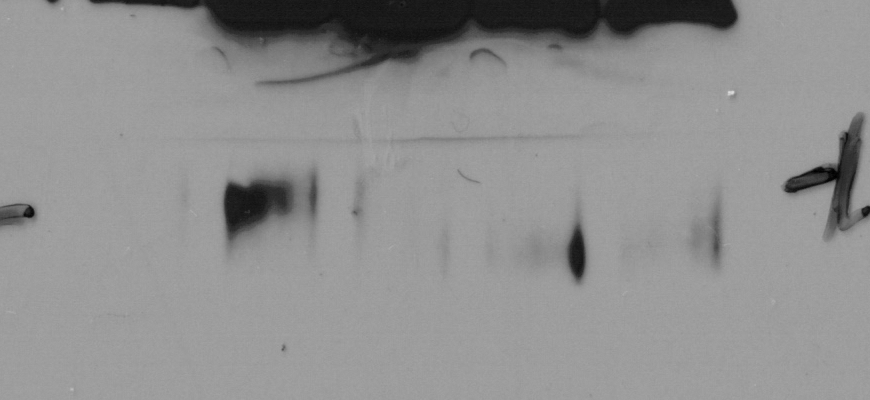

Supplement: Figure 5—source data 1. [file elife-86168-fig5-data1.zip › Figure 5 source data 1/Fig.5E 20200710 h1299-ezh2 ip-ha IP l3mbtl3.tif]

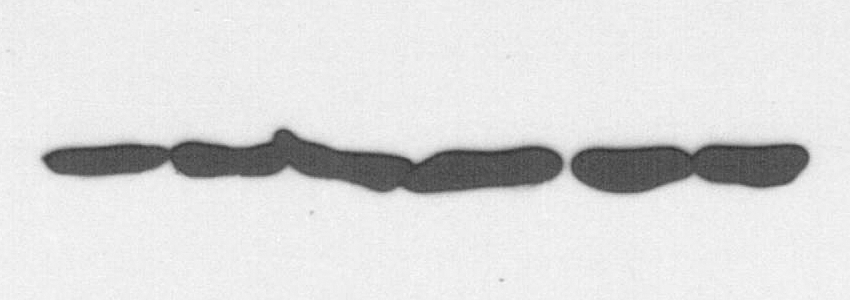

Supplement: Figure 5—source data 1. [file elife-86168-fig5-data1.zip › Figure 5 source data 1/Fig.5F 20220614 G401 EZH2 WT K20R S21A SI LSD1-2 Anti-H3 Uncropped.tif]

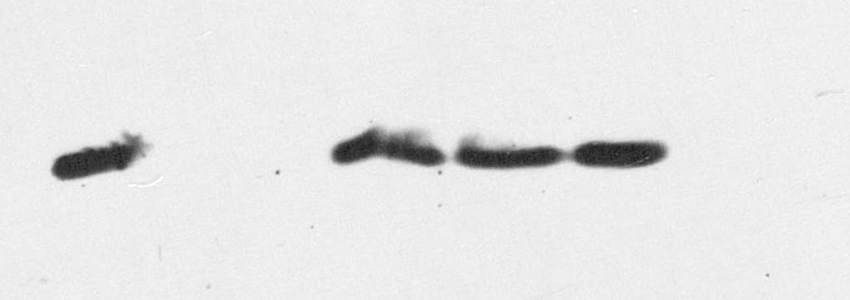

Supplement: Figure 5—source data 1. [file elife-86168-fig5-data1.zip › Figure 5 source data 1/Fig.5F 20220614 G401 EZH2 WT K20R S21A SI LSD1-2 Anti-H3k27me3 Uncropped.tif]

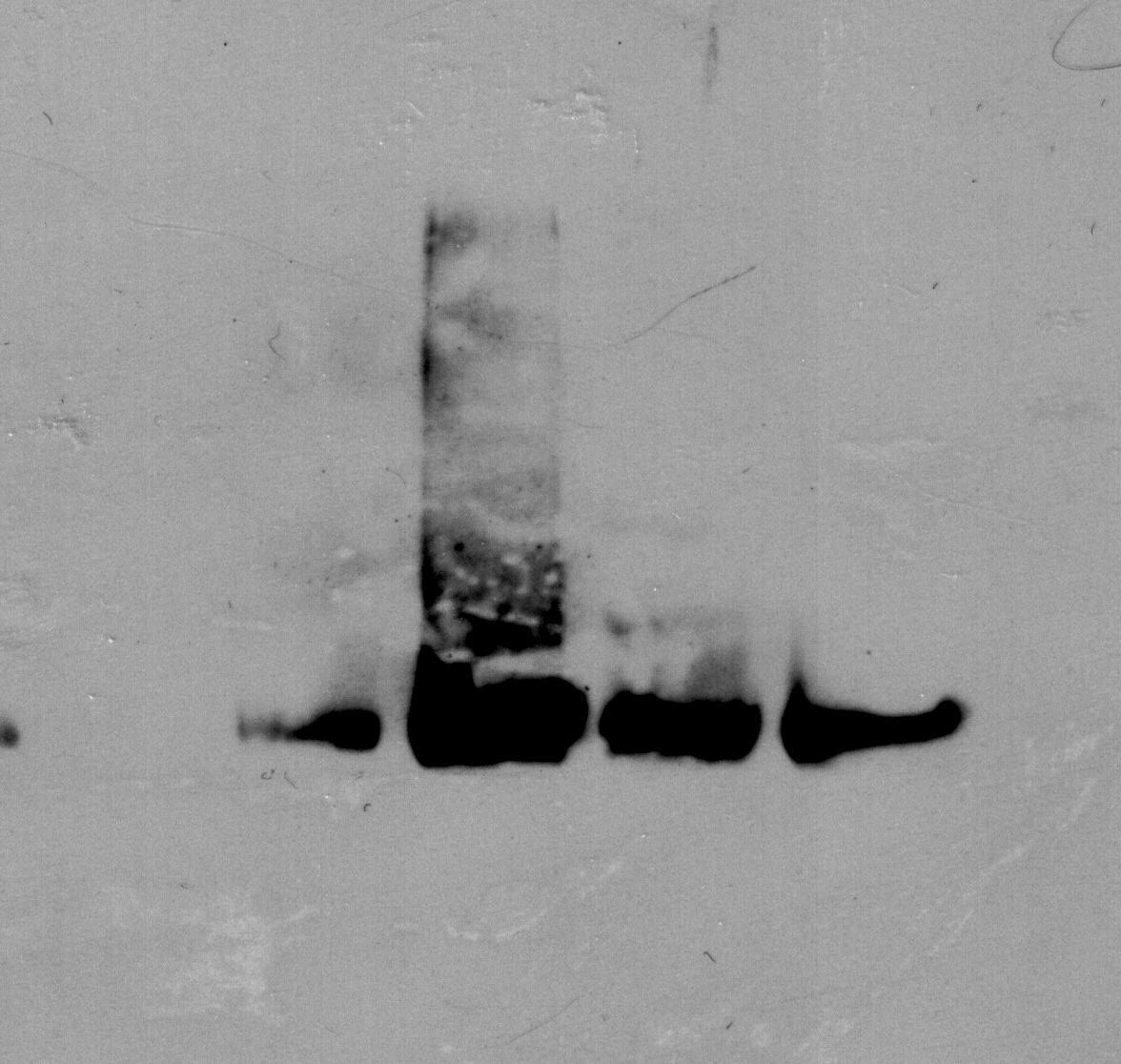

Supplement: Figure 5—source data 1. [file elife-86168-fig5-data1.zip › Figure 5 source data 1/Fig.5H ezh2 wt k20r ub gfp-IP anti-GFP-EZH2 1.tif]

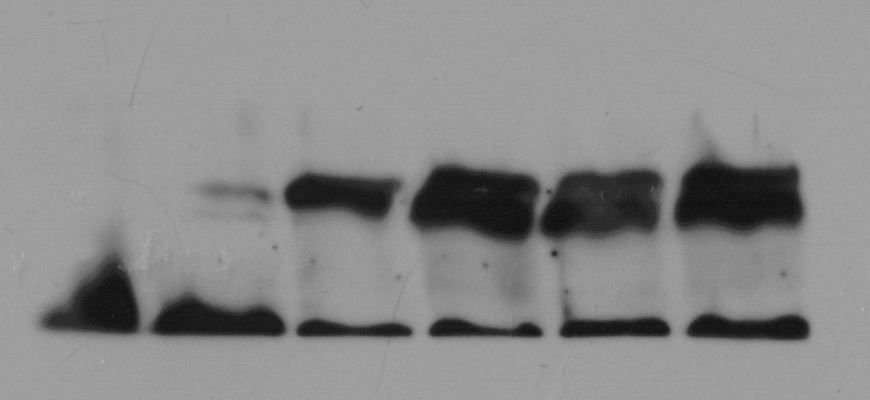

Supplement: Figure 5—source data 1. [file elife-86168-fig5-data1.zip › Figure 5 source data 1/Fig.5E 20200710 h1299-ezh2 ip-ha input ha-ezh2.tif]

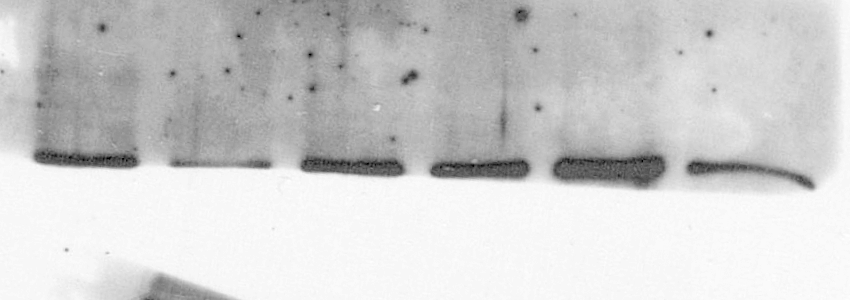

Supplement: Figure 5—source data 1. [file elife-86168-fig5-data1.zip › Figure 5 source data 1/Fig.5F 20220614 G401 EZH2 WT K20R S21A SI LSD1-2 Anti-HA-EZH2 Uncropped.tif]
