# Supplementary material for: A methylation-phosphorylation switch controls EZH2 stability and hematopoiesis
Source: eLife. 2024 Feb 12;13:e86168. doi: 10.7554/eLife.86168 (PMC10901513; doi:10.7554/eLife.86168)

Figure 4-figure supplement 1-EZH2

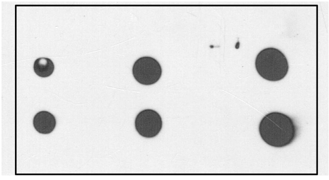

Figure 4-figure supplement 1-EZH2-K20me

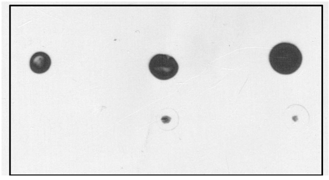

Figure 4-figure supplement 1-EZH2

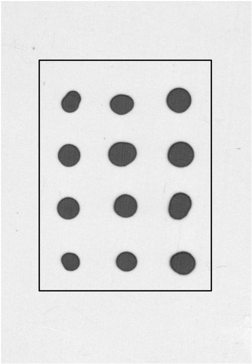

Figure 4-figure supplement 1-EZH2-K20me

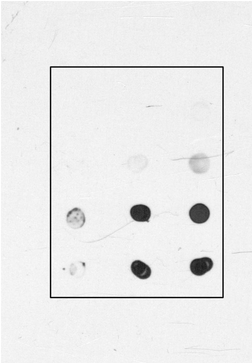

Supplement: Figure 4—figure supplement 1—source data 1. [file elife-86168-fig4-figsupp1-data1.zip › Figure 4-figure supplement 1 source data 1/Figure 4-figure supplement 1.pdf]
