# Supplementary material for: A methylation-phosphorylation switch controls EZH2 stability and hematopoiesis
Source: eLife. 2024 Feb 12;13:e86168. doi: 10.7554/eLife.86168 (PMC10901513; doi:10.7554/eLife.86168)

Fig.4-figure supplement 2A-EZH2

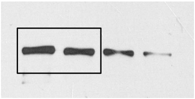

Fig.4-figure supplement 2A-EZH2-K20me

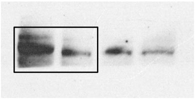

Fig.4-figure supplement 2A-SET7

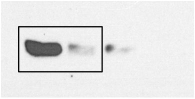

Fig.4-figure supplement 2A-GAPDH

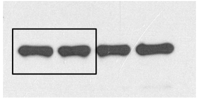

Figure 4-figure supplement 2B-Flag-EZH2

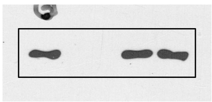

Figure 4-figure supplement 2B-PHF20L1

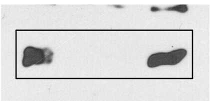

Figure 4-figure supplement 2B-L3MBTL3

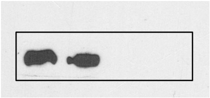

Figure 4-figure supplement 2B-Actin

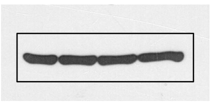

Figure 4-figure supplement 2C-Flag-EZH2

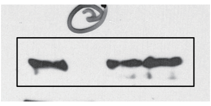

Figure 4-figure supplement 2C-PHF20L1

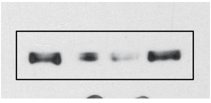

Figure 4-figure supplement 2C-L3MBTL3

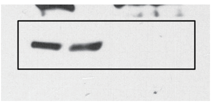

Figure 4-figure supplement 2C-Actin

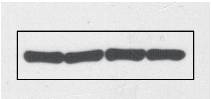

Fig.4-figure supplement 2D-EZH2

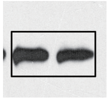

Fig.4-figure supplement 2D-EZH2-K20me

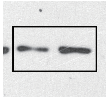

Fig.4-figure supplement 2D-EZH2-S21p

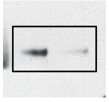

Fig.4-figure supplement 2D-Actin

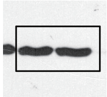

Fig.4-figure supplement 2D-H3K27me3

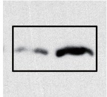

Fig.4-figure supplement 2D-H3

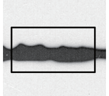

Supplement: Figure 4—figure supplement 2—source data 1. [file elife-86168-fig4-figsupp2-data1.zip › Figure 4-figure supplement 2 source data 1/Figure 4-figure supplement 2.pdf]
