# Supplementary material for: A methylation-phosphorylation switch controls EZH2 stability and hematopoiesis
Source: eLife. 2024 Feb 12;13:e86168. doi: 10.7554/eLife.86168 (PMC10901513; doi:10.7554/eLife.86168)

Figure 5A-EZH2

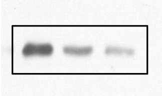

Figure 5A-EZH2-K20me

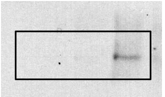

Figure 5A-EZH2-S21p

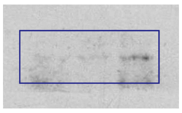

Figure 5A-Actin

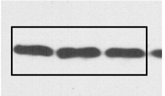

Figure 5A-H3K27me3

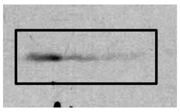

Figure 5A-H3

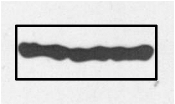

Figure 5C-EZH2

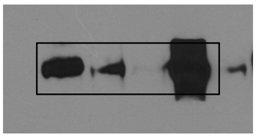

Figure 5C-L3MBTL3

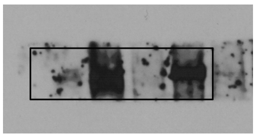

IP

Figure 5D-EZH2

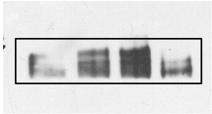

Figure 5D-L3MBTL3

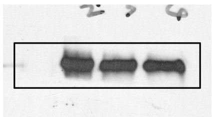

Input

Figure 5D-EZH2

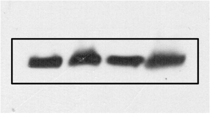

Figure 5D-SET7

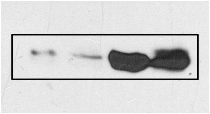

Figure 5D-L3MBTL3

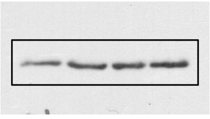

Figure 5D-Actin

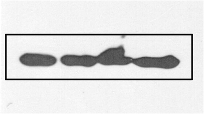

IP

Figure 5E-L3MBTL3

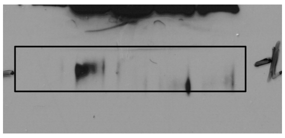

Figure 5E-HA-EZH2

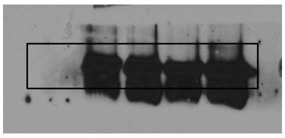

Figure 5E-SET7

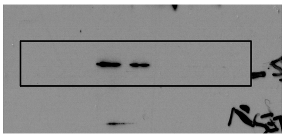

Input

Figure 5E-HA-EZH2

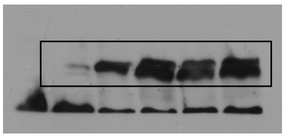

Figure 5E-L3MBTL3

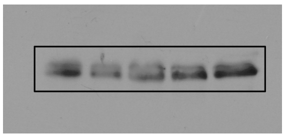

Figure 5E-Actin

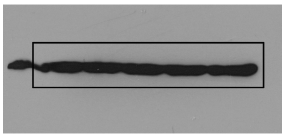

Figure 5F-Flag-EZH2

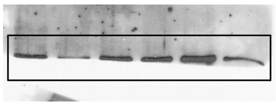

Figure 5F-Endogenous EZH2

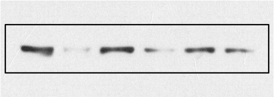

Figure 5F-EZH2-K20me

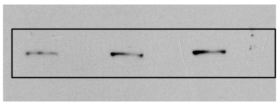

Figure 5F-LSD1

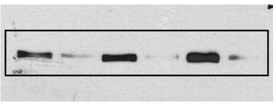

Figure 5F-Actin

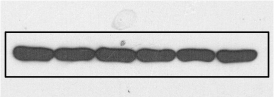

Figure 5F-H3K27me3

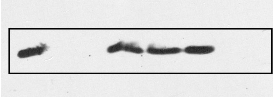

Figure 5F-H3

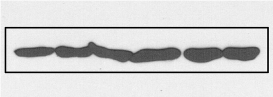

IP

Figure 5G-GFP

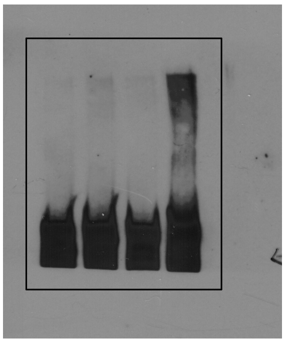

Input

Figure 5G-SET7

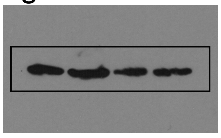

Figure 5G-Flag-DCAF5

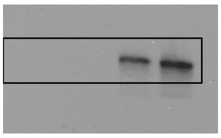

Figure 5G-L3MBTL3

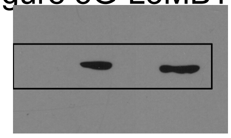

Figure 5G-GFP-EZH2

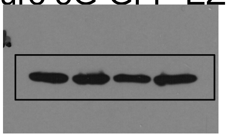

Figure 5G-Actin

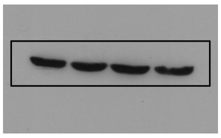

IP

Figure 5H-GFP

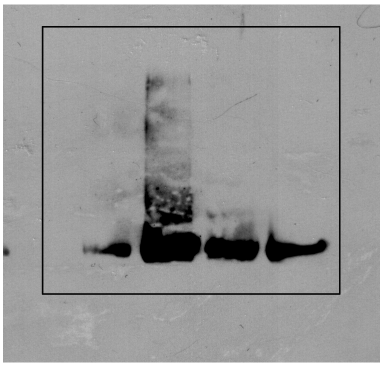

Input

Figure 5H-SET7

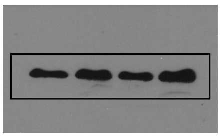

Figure 5H-Flag-DCAF5

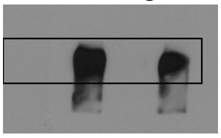

Figure 5H-L3MBTL3

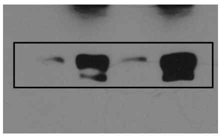

Figure 5H-GFP-EZH2

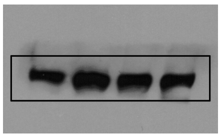

Figure 5H-Actin

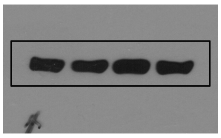

Supplement: Figure 5—source data 1. [file elife-86168-fig5-data1.zip › Figure 5 source data 1/Figure 5-annotated source data .pdf]
