# Supplementary figures and images for: A methylation-phosphorylation switch controls EZH2 stability and hematopoiesis (part 4 of 7)
Source: eLife. 2024 Feb 12;13:e86168. doi: 10.7554/eLife.86168 (PMC10901513; doi:10.7554/eLife.86168)

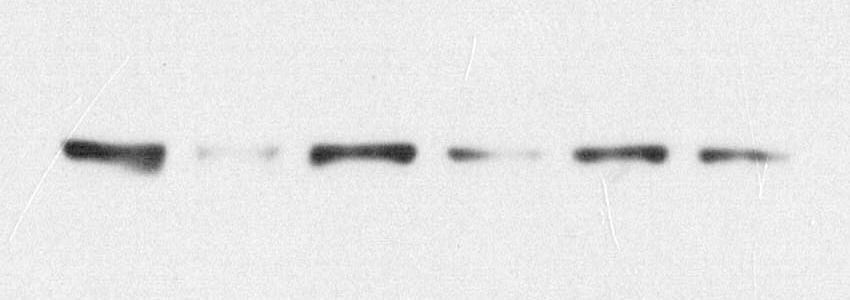

Supplement: Figure 5—source data 1. [file elife-86168-fig5-data1.zip › Figure 5 source data 1/Fig.5F EZH2 WT K20R S21A SI LSD1-2 Anti-EZH2 Uncropped.tif]

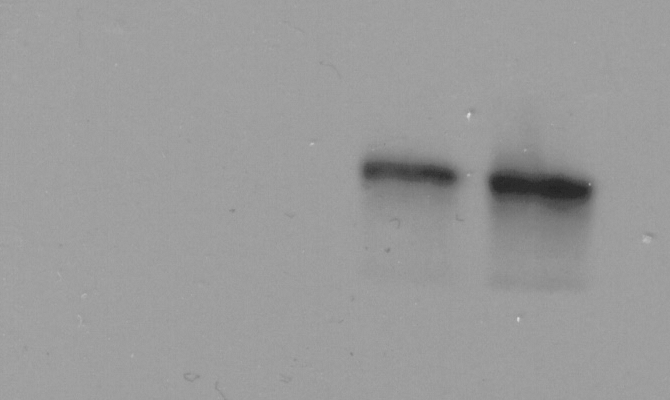

Supplement: Figure 5—source data 1. [file elife-86168-fig5-data1.zip › Figure 5 source data 1/Fig.5G EZH2-UB Anti-Flag-DCAF5.tif]

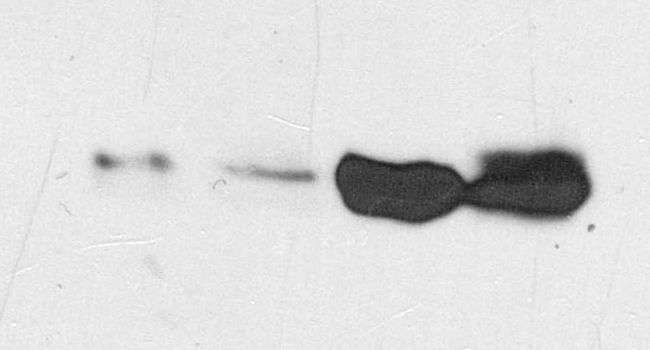

Supplement: Figure 5—source data 1. [file elife-86168-fig5-data1.zip › Figure 5 source data 1/Fig.5D 20220502 293t l3 ip with ezh2 transfect with set7 wt h297a input anti-SET7 uncropped.tif]

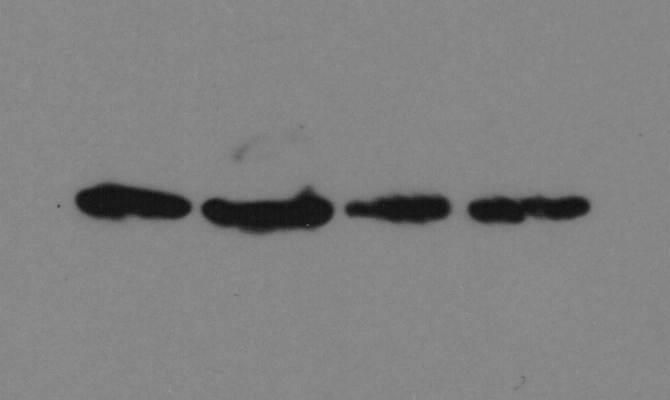

Supplement: Figure 5—source data 1. [file elife-86168-fig5-data1.zip › Figure 5 source data 1/Fig.5G UB Anti-SET7.tif]

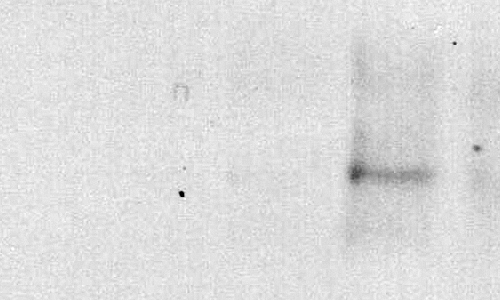

Supplement: Figure 5—source data 1. [file elife-86168-fig5-data1.zip › Figure 5 source data 1/Fig.5A 20210811 mouse brain e14 e18 p0 check EZH2 K20me anti-ezh2-k20me uncropped.tif]

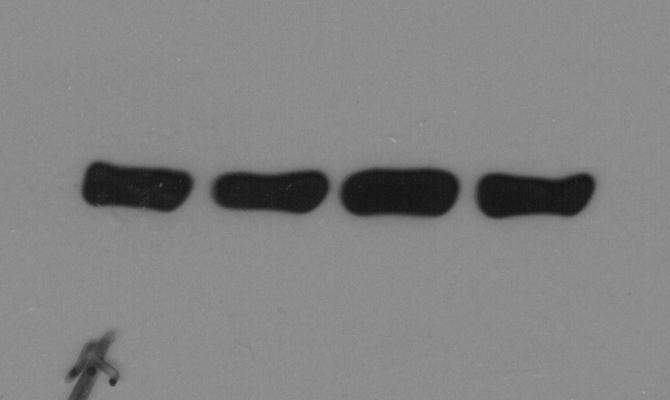

Supplement: Figure 5—source data 1. [file elife-86168-fig5-data1.zip › Figure 5 source data 1/Fig.5H ezh2 wt k20r ub input anti-Actin.tif]

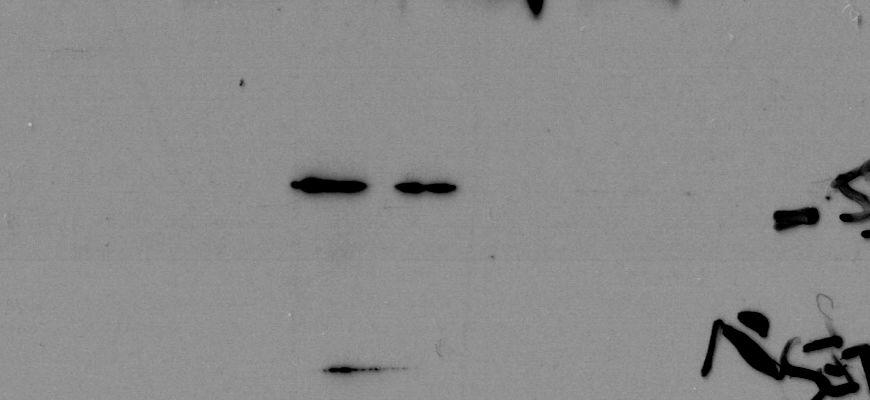

Supplement: Figure 5—source data 1. [file elife-86168-fig5-data1.zip › Figure 5 source data 1/Fig.5E 20200710 h1299-ezh2 ip-ha input set7.tif]

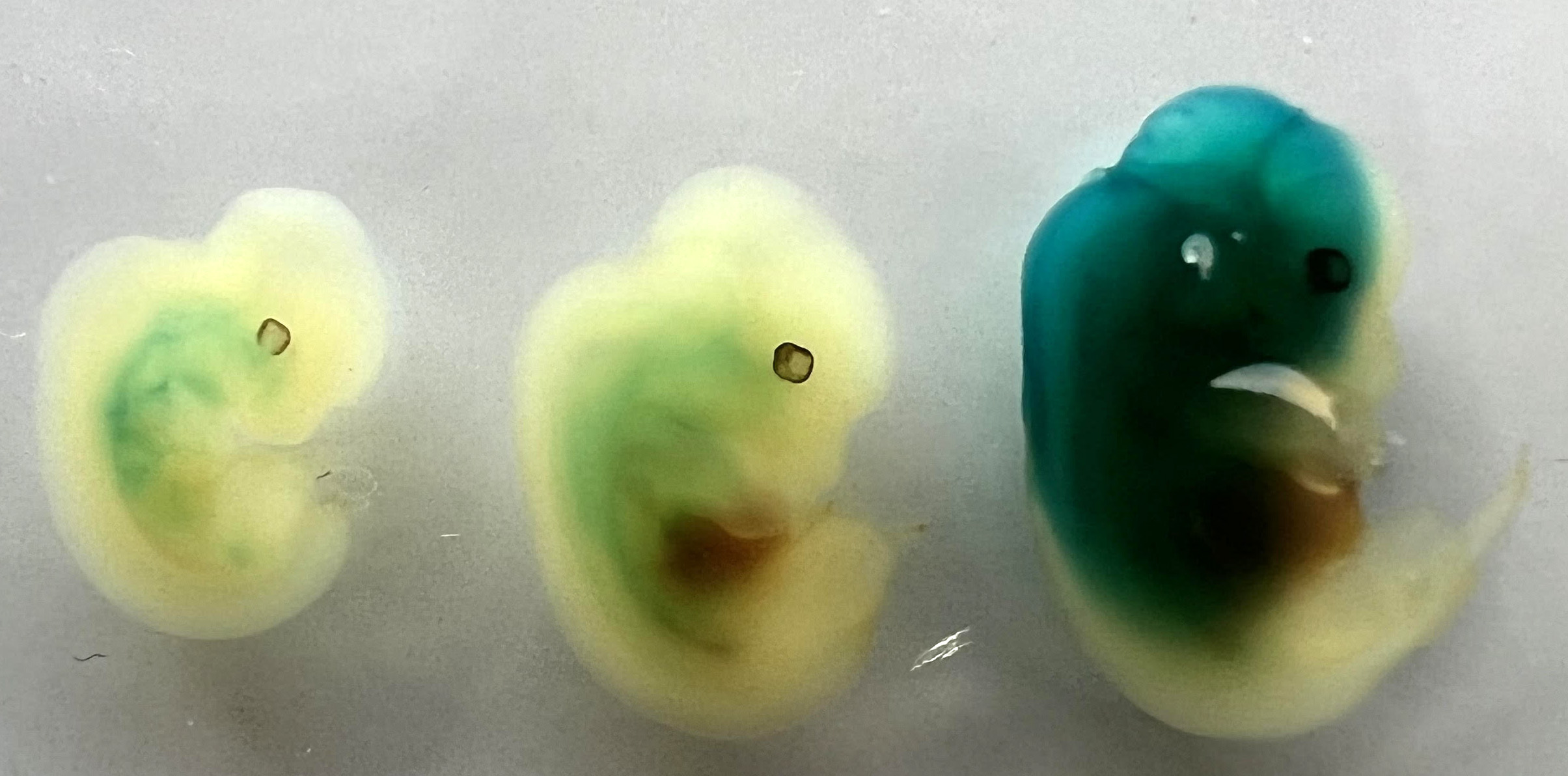

Supplement: Figure 5—source data 1. [file elife-86168-fig5-data1.zip › Figure 5 source data 1/Fig.5B l3-lacz.tiff]

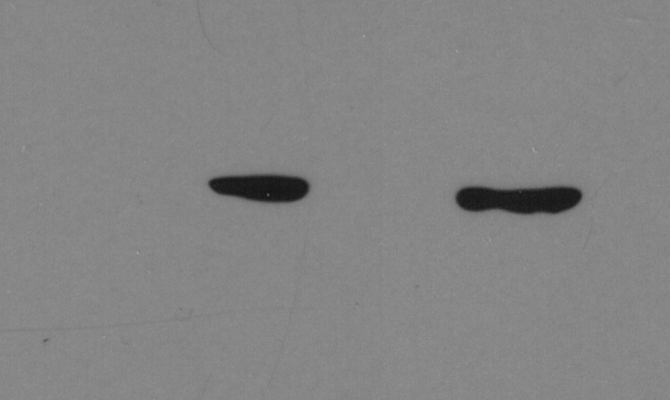

Supplement: Figure 5—source data 1. [file elife-86168-fig5-data1.zip › Figure 5 source data 1/Fig.5G EZH2-UB Anti-L3MBTL3.tif]

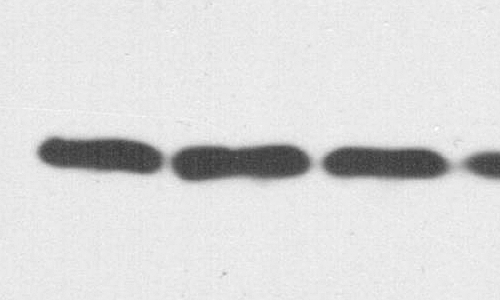

Supplement: Figure 5—source data 1. [file elife-86168-fig5-data1.zip › Figure 5 source data 1/Fig.5A 20210811 mouse brain e14 e18 p0 check EZH2 K20me anti-actin uncropped.tif]

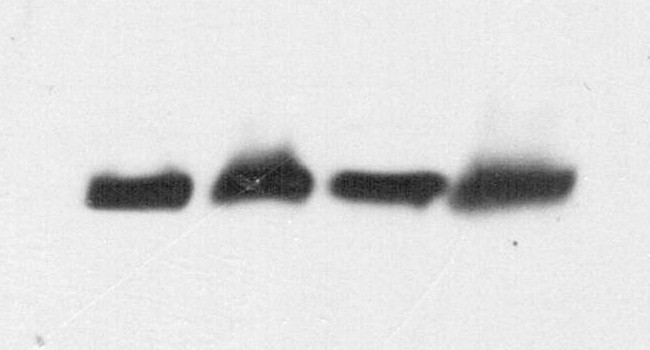

Supplement: Figure 5—source data 1. [file elife-86168-fig5-data1.zip › Figure 5 source data 1/Fig.5D 20220502 293t l3 ip with ezh2 transfect with set7 wt h297a input anti-EZH2 uncropped.tif]

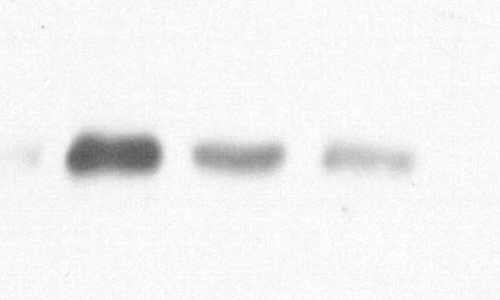

Supplement: Figure 5—source data 1. [file elife-86168-fig5-data1.zip › Figure 5 source data 1/Fig.5A 20210811 mouse brain e14 e18 p0 check EZH2 K20me anti-ezh2 uncropped.tif]

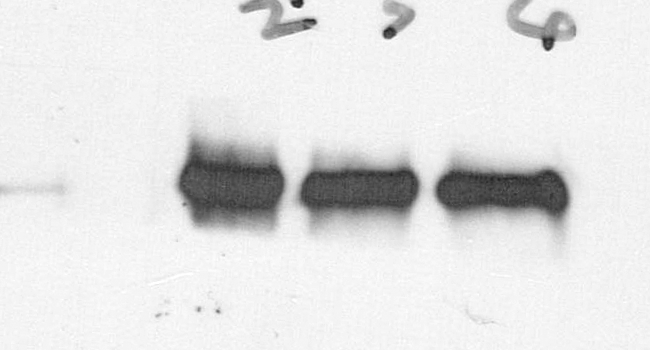

Supplement: Figure 5—source data 1. [file elife-86168-fig5-data1.zip › Figure 5 source data 1/Fig.5D 20220502 293t l3 ip with ezh2 transfect with set7 wt h297a iP-L3 anti-L3 uncropped.tif]

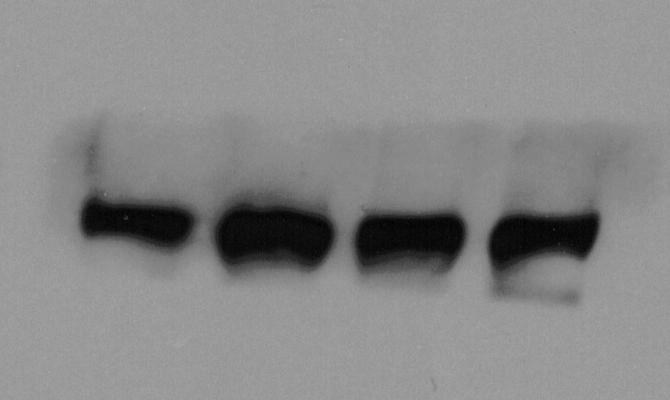

Supplement: Figure 5—source data 1. [file elife-86168-fig5-data1.zip › Figure 5 source data 1/Fig.5H ezh2 wt k20r ub input anti-EGFP-EZH2.tif]

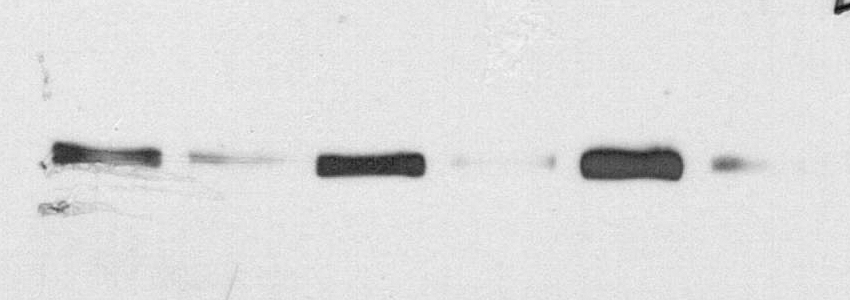

Supplement: Figure 5—source data 1. [file elife-86168-fig5-data1.zip › Figure 5 source data 1/Fig.5F 20220614 G401 EZH2 WT K20R S21A SI LSD1-2 Anti-lsd1 Uncropped.tif]

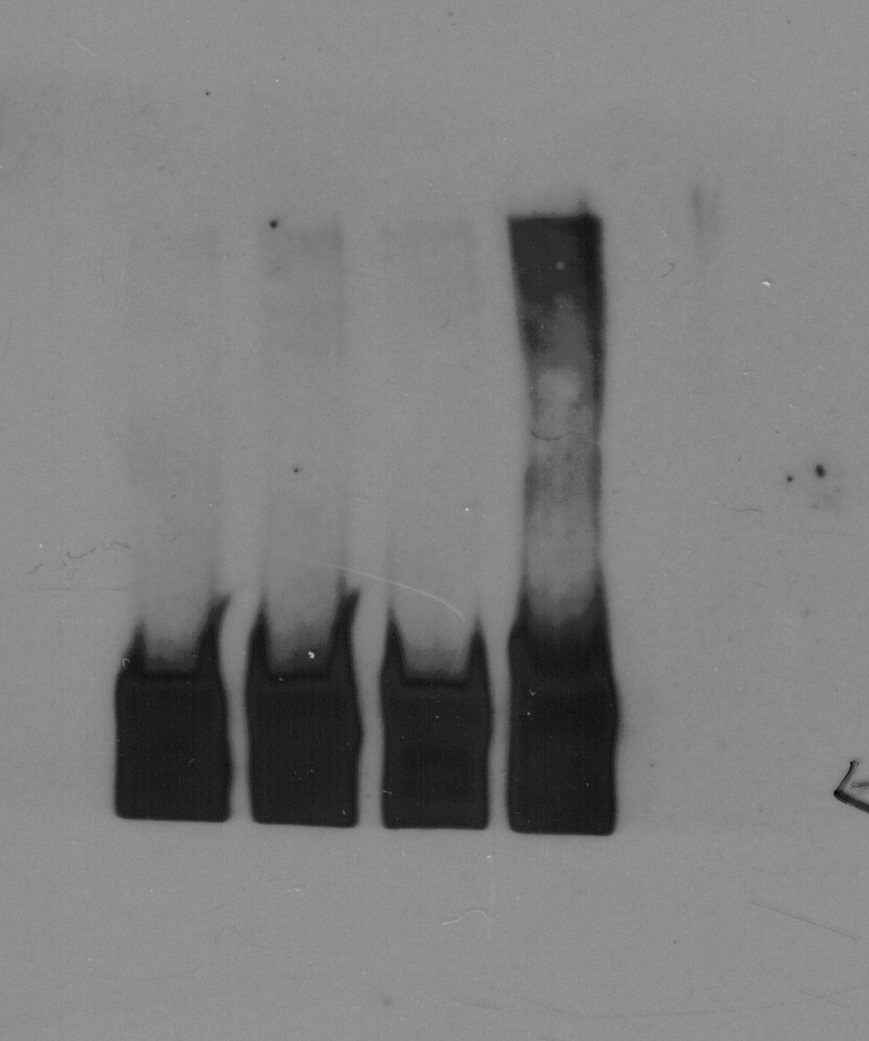

Supplement: Figure 5—source data 1. [file elife-86168-fig5-data1.zip › Figure 5 source data 1/Fig.5G EZH2-UB IP Anti-GFP-EZH2.tif]

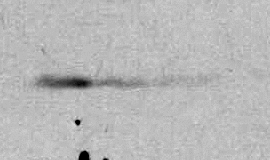

Supplement: Figure 5—source data 1. [file elife-86168-fig5-data1.zip › Figure 5 source data 1/Fig.5A 20210811 mouse brain e14 e18 p0 check EZH2 K20me anti-H3K27me3 uncropped.tif]

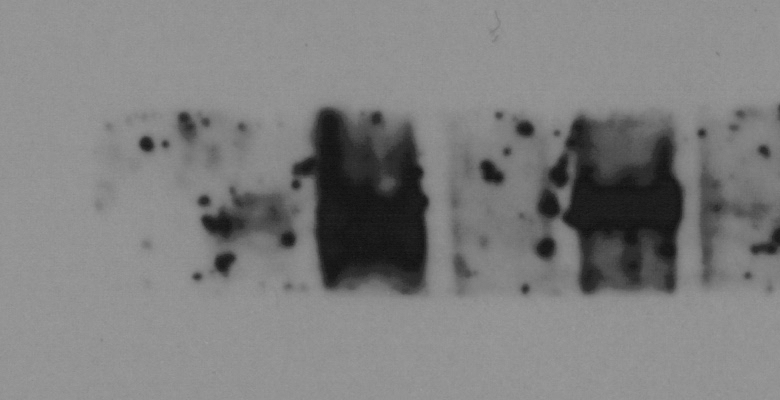

Supplement: Figure 5—source data 1. [file elife-86168-fig5-data1.zip › Figure 5 source data 1/Fig.5C 20200306 L3MBTL3 ip anti-L3.tif]

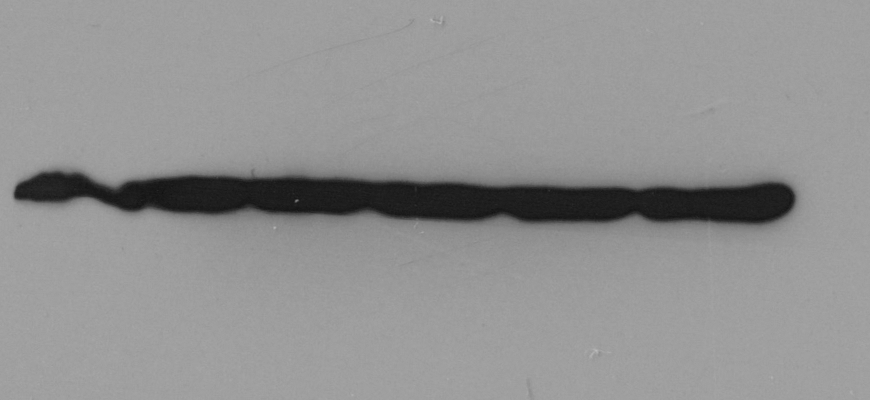

Supplement: Figure 5—source data 1. [file elife-86168-fig5-data1.zip › Figure 5 source data 1/Fig.5E 20200710 h1299-ezh2 ip-ha input Actin.tif]

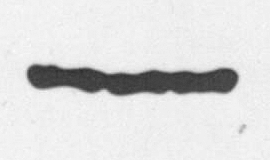

Supplement: Figure 5—source data 1. [file elife-86168-fig5-data1.zip › Figure 5 source data 1/Fig.5A 20210811 mouse brain e14 e18 p0 check EZH2 K20me anti-H3 uncropped.tif]

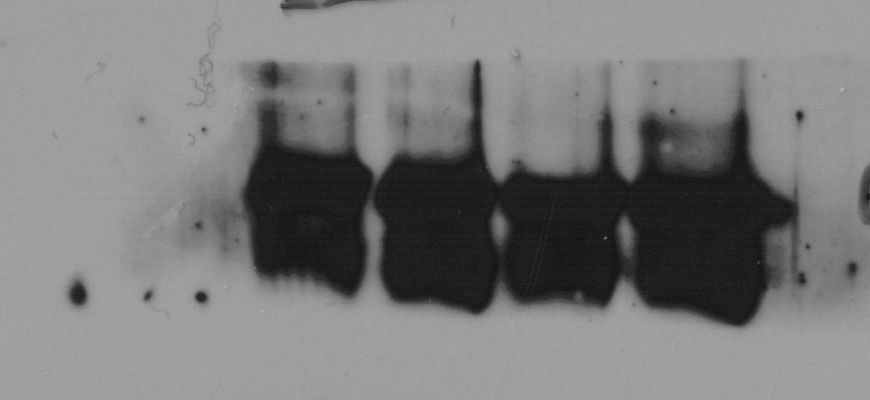

Supplement: Figure 5—source data 1. [file elife-86168-fig5-data1.zip › Figure 5 source data 1/Fig.5E 20200710 h1299-ezh2 ip-ha IP ha-ezh2.tif]

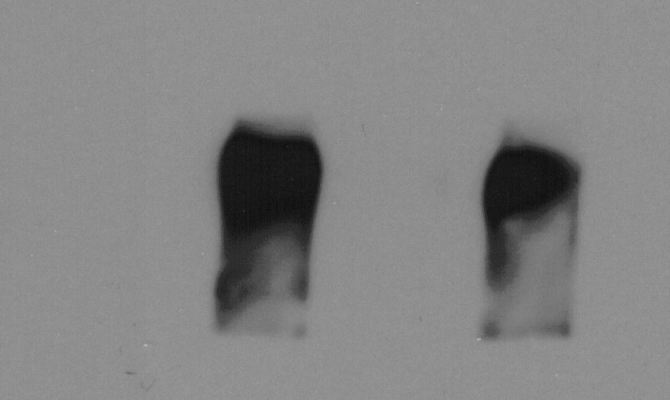

Supplement: Figure 5—source data 1. [file elife-86168-fig5-data1.zip › Figure 5 source data 1/Fig.5H ezh2 wt k20r ub input anti-flag-D5.tif]

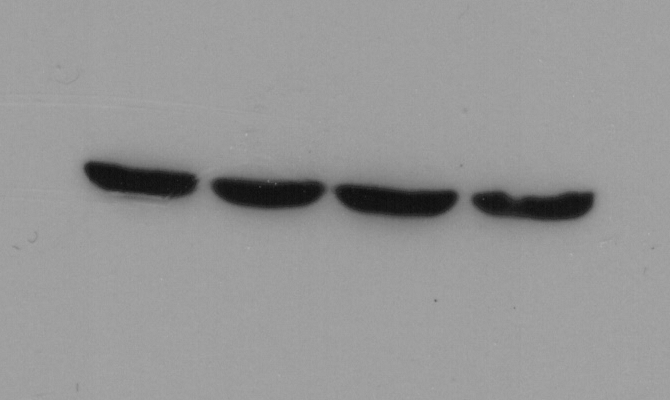

Supplement: Figure 5—source data 1. [file elife-86168-fig5-data1.zip › Figure 5 source data 1/Fig.5G EZH2-UB Anti-Actin.tif]

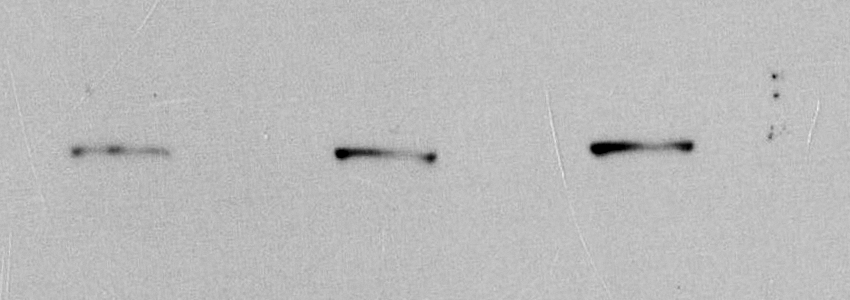

Supplement: Figure 5—source data 1. [file elife-86168-fig5-data1.zip › Figure 5 source data 1/Fig.5F G401 EZH2 WT K20R S21A SI LSD1-2 Anti-EZH2-K20me Uncropped.tif]

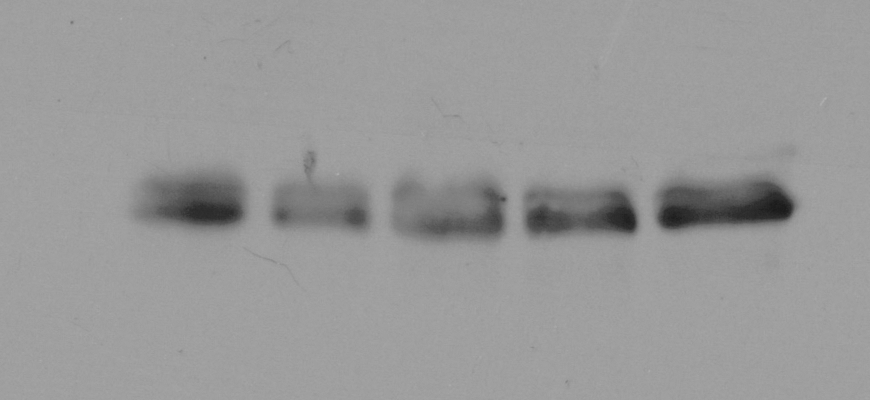

Supplement: Figure 5—source data 1. [file elife-86168-fig5-data1.zip › Figure 5 source data 1/Fig.5E 20200710 h1299-ezh2 ip-ha input L3MBTL3.tif]

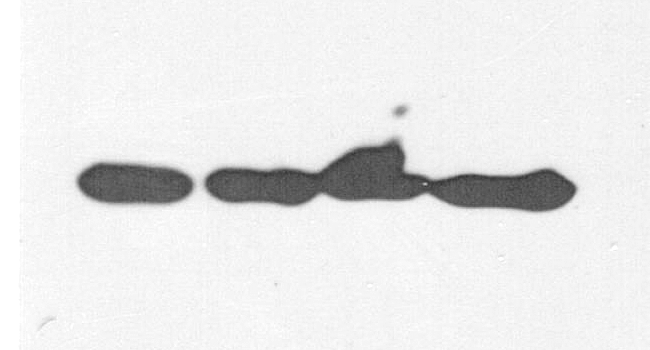

Supplement: Figure 5—source data 1. [file elife-86168-fig5-data1.zip › Figure 5 source data 1/Fig.5D 20220502 293t l3 ip with ezh2 transfect with set7 wt h297a anti-actin uncropped.tif]

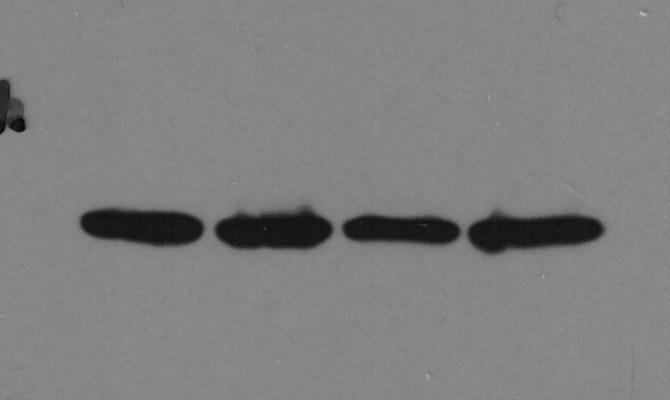

Supplement: Figure 5—source data 1. [file elife-86168-fig5-data1.zip › Figure 5 source data 1/Fig.5G EZH2-UB Anti-EGFP-EZH2.tif]

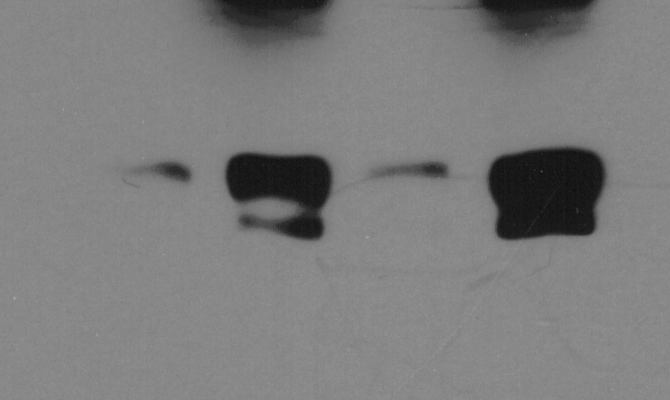

Supplement: Figure 5—source data 1. [file elife-86168-fig5-data1.zip › Figure 5 source data 1/Fig.5H ezh2 wt k20r ub input anti-L3.tif]

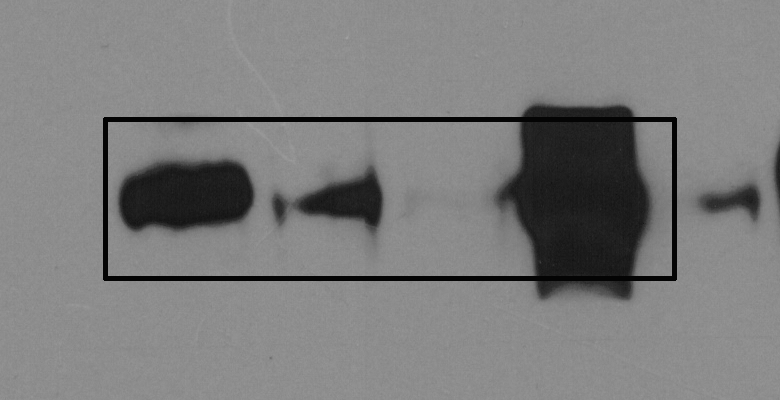

Supplement: Figure 5—source data 1. [file elife-86168-fig5-data1.zip › Figure 5 source data 1/annotated/Fig.5C 20200306 EZH2 ip anti-EZH2.tif]

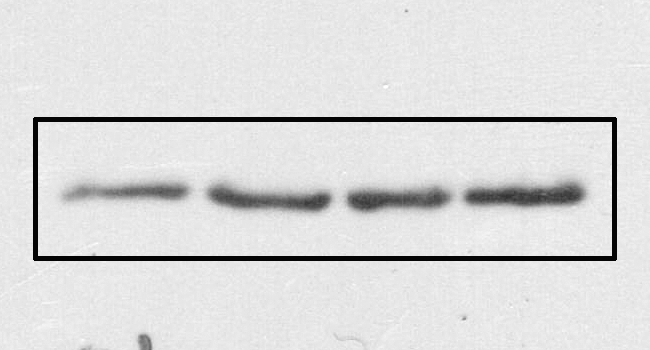

Supplement: Figure 5—source data 1. [file elife-86168-fig5-data1.zip › Figure 5 source data 1/annotated/Fig.5D 20220502 293t l3 ip with ezh2 transfect with set7 wt h297a input anti-l3 uncropped.tif]

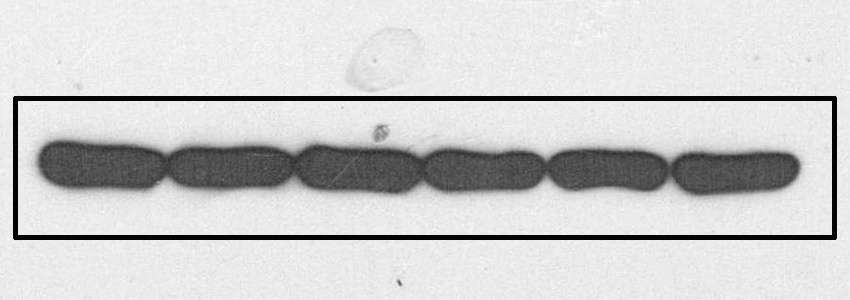

Supplement: Figure 5—source data 1. [file elife-86168-fig5-data1.zip › Figure 5 source data 1/annotated/Fig.5F 20220614 G401 EZH2 WT K20R S21A SI LSD1-2 Anti-actin Uncropped.tif]

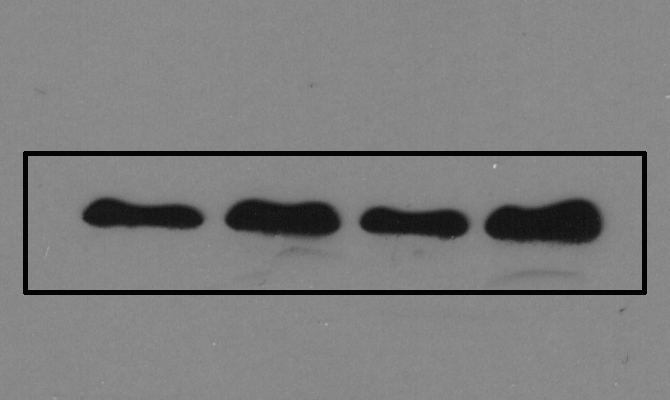

Supplement: Figure 5—source data 1. [file elife-86168-fig5-data1.zip › Figure 5 source data 1/annotated/Fig.5H ezh2 wt k20r ub input anti-SET7.tif]

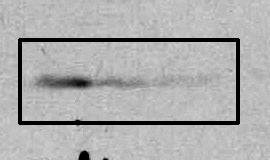

Supplement: Figure 5—source data 1. [file elife-86168-fig5-data1.zip › Figure 5 source data 1/annotated/Fig.5A mouse brain e14 e18 p0 check EZH2 K20me anti-H3K27me3 uncropped.tif]

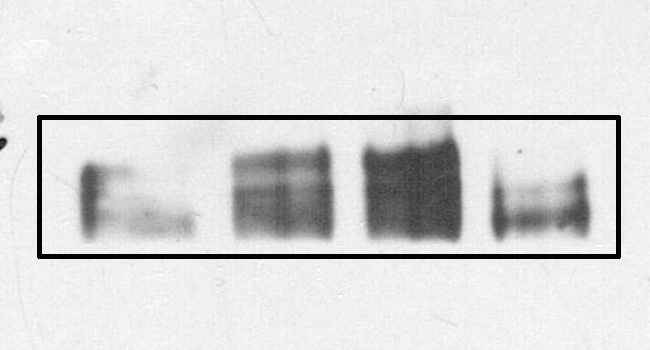

Supplement: Figure 5—source data 1. [file elife-86168-fig5-data1.zip › Figure 5 source data 1/annotated/Fig.5D 20220502 293t l3 ip with ezh2 transfect with set7 wt h297a iP-L3 anti-EZH2 uncropped.tif]

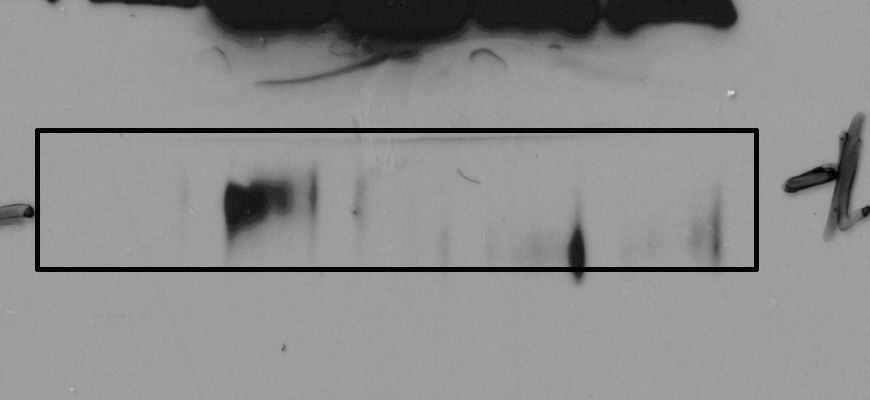

Supplement: Figure 5—source data 1. [file elife-86168-fig5-data1.zip › Figure 5 source data 1/annotated/Fig.5E 20200710 h1299-ezh2 ip-ha IP l3mbtl3.tif]

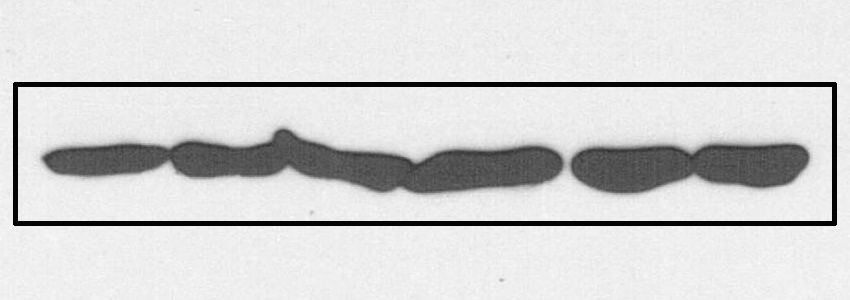

Supplement: Figure 5—source data 1. [file elife-86168-fig5-data1.zip › Figure 5 source data 1/annotated/Fig.5F 20220614 G401 EZH2 WT K20R S21A SI LSD1-2 Anti-H3 Uncropped.tif]

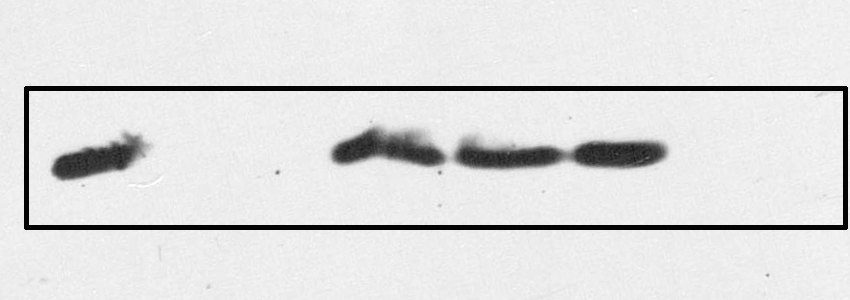

Supplement: Figure 5—source data 1. [file elife-86168-fig5-data1.zip › Figure 5 source data 1/annotated/Fig.5F 20220614 G401 EZH2 WT K20R S21A SI LSD1-2 Anti-H3k27me3 Uncropped.tif]

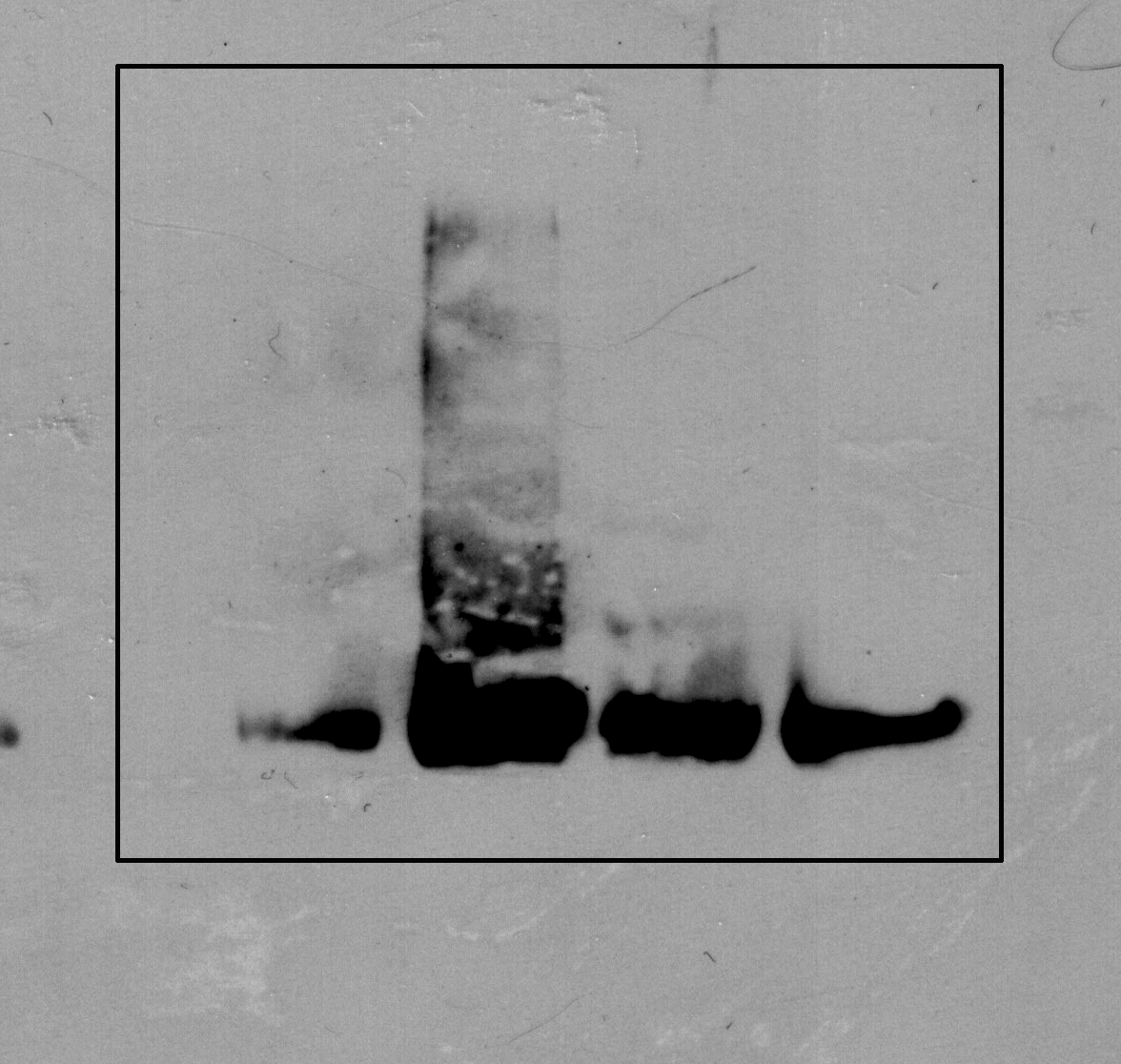

Supplement: Figure 5—source data 1. [file elife-86168-fig5-data1.zip › Figure 5 source data 1/annotated/Fig.5H ezh2 wt k20r ub gfp-IP anti-GFP-EZH2 1.tif]

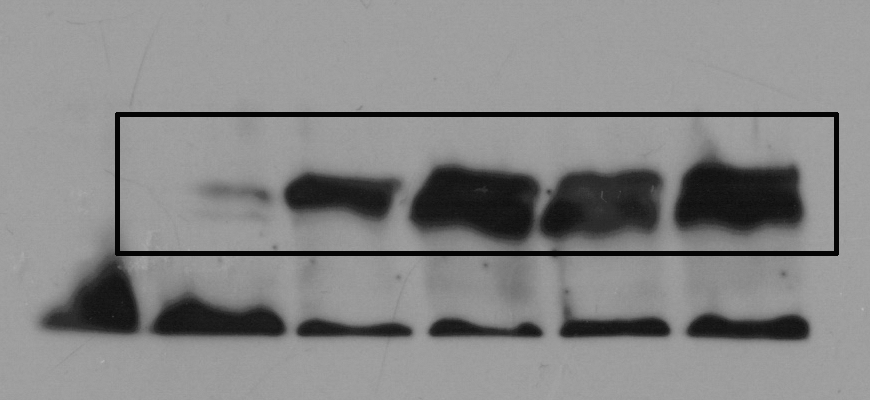

Supplement: Figure 5—source data 1. [file elife-86168-fig5-data1.zip › Figure 5 source data 1/annotated/Fig.5E 20200710 h1299-ezh2 ip-ha input ha-ezh2.tif]

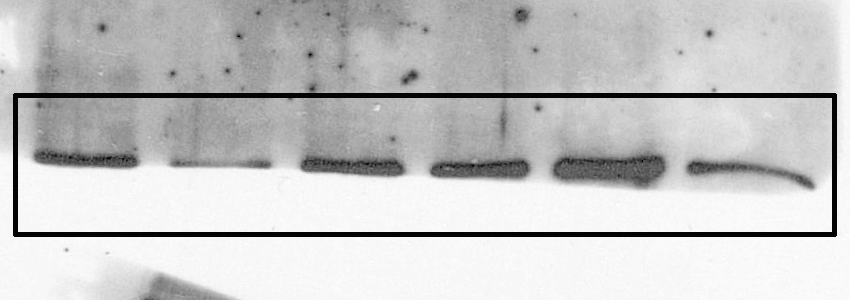

Supplement: Figure 5—source data 1. [file elife-86168-fig5-data1.zip › Figure 5 source data 1/annotated/Fig.5F 20220614 G401 EZH2 WT K20R S21A SI LSD1-2 Anti-HA-EZH2 Uncropped.tif]

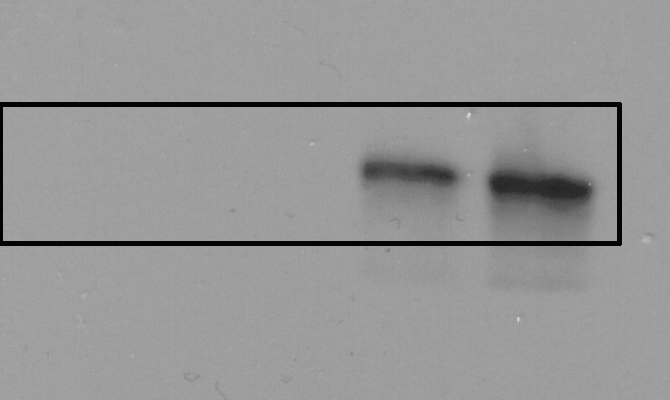

Supplement: Figure 5—source data 1. [file elife-86168-fig5-data1.zip › Figure 5 source data 1/annotated/Fig.5G EZH2-UB Anti-Flag-DCAF5.tif]

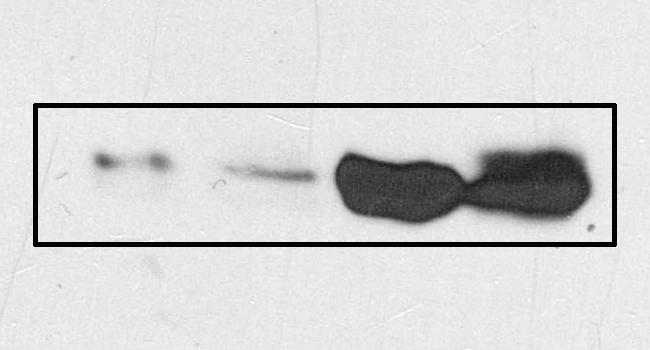

Supplement: Figure 5—source data 1. [file elife-86168-fig5-data1.zip › Figure 5 source data 1/annotated/Fig.5D 20220502 293t l3 ip with ezh2 transfect with set7 wt h297a input anti-SET7 uncropped.tif]

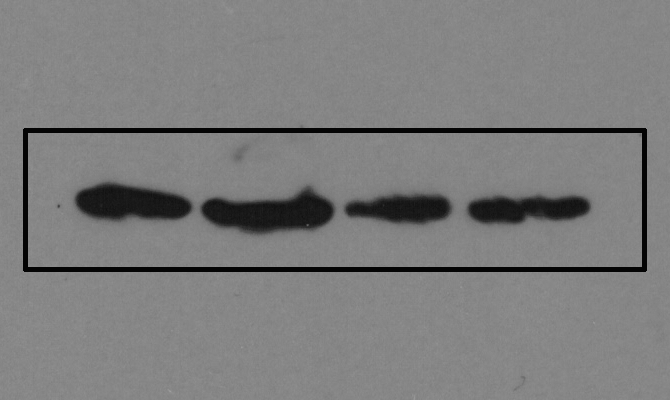

Supplement: Figure 5—source data 1. [file elife-86168-fig5-data1.zip › Figure 5 source data 1/annotated/Fig.5G UB Anti-SET7.tif]

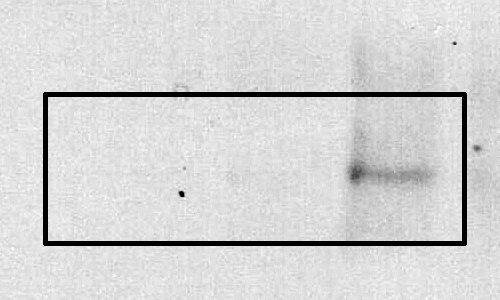

Supplement: Figure 5—source data 1. [file elife-86168-fig5-data1.zip › Figure 5 source data 1/annotated/Fig.5A 20210811 mouse brain e14 e18 p0 check EZH2 K20me anti-ezh2-k20me uncropped.tif]

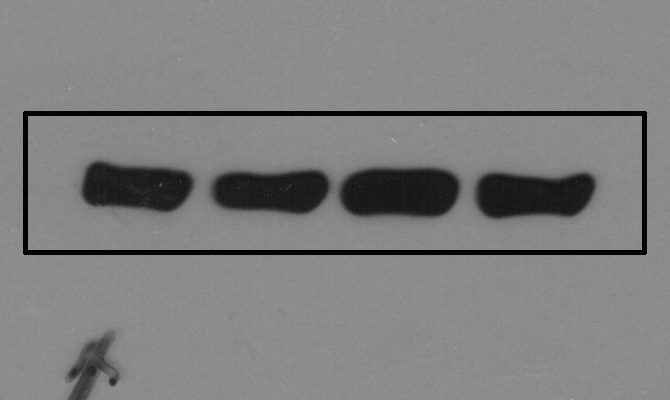

Supplement: Figure 5—source data 1. [file elife-86168-fig5-data1.zip › Figure 5 source data 1/annotated/Fig.5H ezh2 wt k20r ub input anti-Actin.tif]

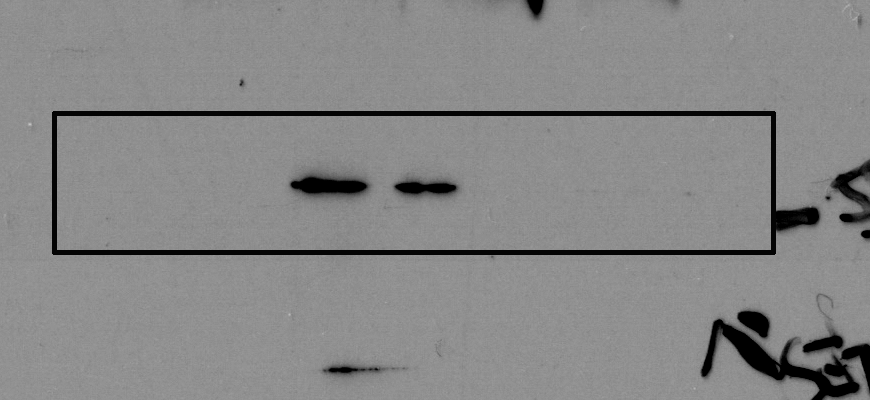

Supplement: Figure 5—source data 1. [file elife-86168-fig5-data1.zip › Figure 5 source data 1/annotated/Fig.5E 20200710 h1299-ezh2 ip-ha input set7.tif]

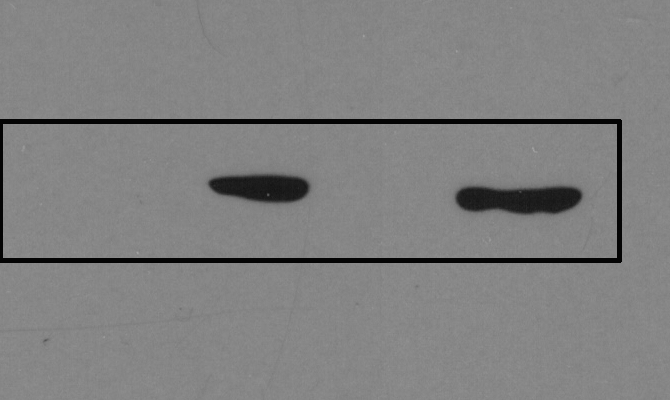

Supplement: Figure 5—source data 1. [file elife-86168-fig5-data1.zip › Figure 5 source data 1/annotated/Fig.5G EZH2-UB Anti-L3MBTL3.tif]

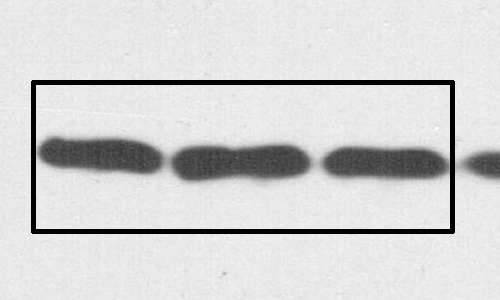

Supplement: Figure 5—source data 1. [file elife-86168-fig5-data1.zip › Figure 5 source data 1/annotated/Fig.5A 20210811 mouse brain e14 e18 p0 check EZH2 K20me anti-actin uncropped.tif]

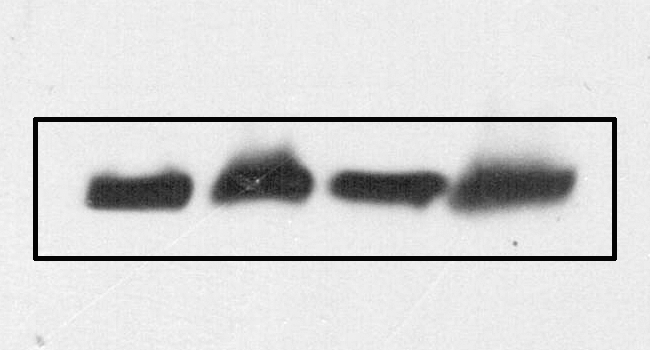

Supplement: Figure 5—source data 1. [file elife-86168-fig5-data1.zip › Figure 5 source data 1/annotated/Fig.5D 20220502 293t l3 ip with ezh2 transfect with set7 wt h297a input anti-EZH2 uncropped.tif]

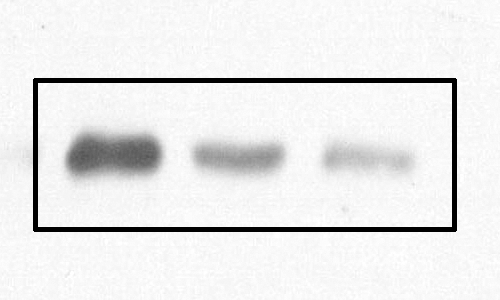

Supplement: Figure 5—source data 1. [file elife-86168-fig5-data1.zip › Figure 5 source data 1/annotated/Fig.5A 20210811 mouse brain e14 e18 p0 check EZH2 K20me anti-ezh2 uncropped.tif]

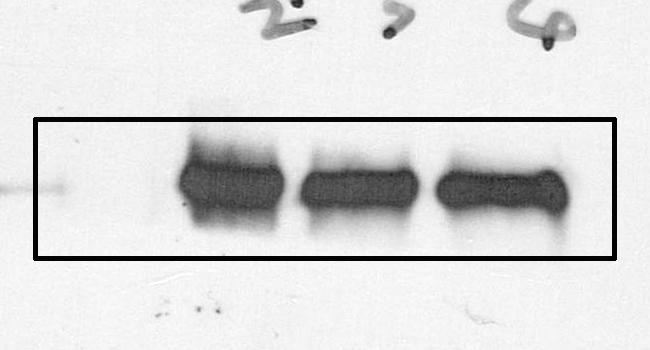

Supplement: Figure 5—source data 1. [file elife-86168-fig5-data1.zip › Figure 5 source data 1/annotated/Fig.5D 20220502 293t l3 ip with ezh2 transfect with set7 wt h297a iP-L3 anti-L3 uncropped.tif]

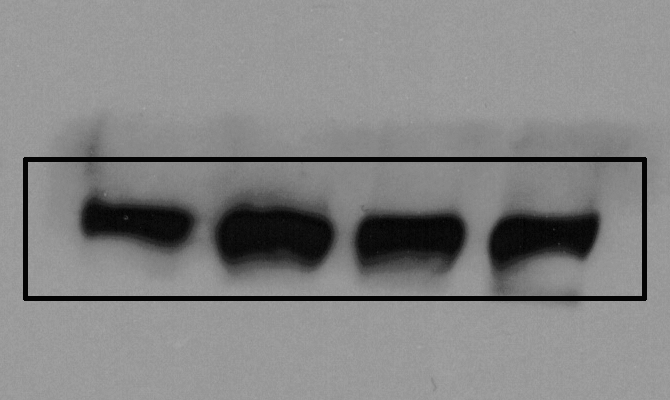

Supplement: Figure 5—source data 1. [file elife-86168-fig5-data1.zip › Figure 5 source data 1/annotated/Fig.5H ezh2 wt k20r ub input anti-EGFP-EZH2.tif]

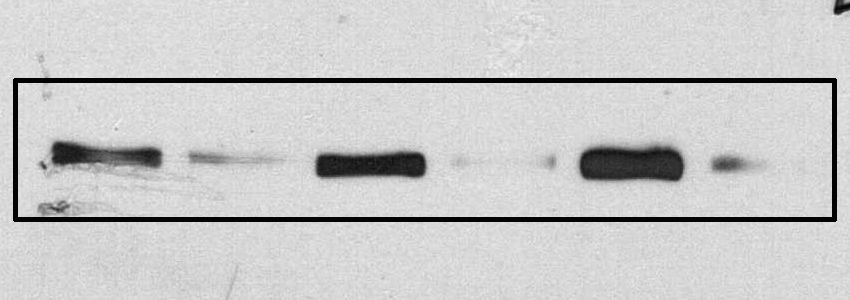

Supplement: Figure 5—source data 1. [file elife-86168-fig5-data1.zip › Figure 5 source data 1/annotated/Fig.5F 20220614 G401 EZH2 WT K20R S21A SI LSD1-2 Anti-lsd1 Uncropped.tif]

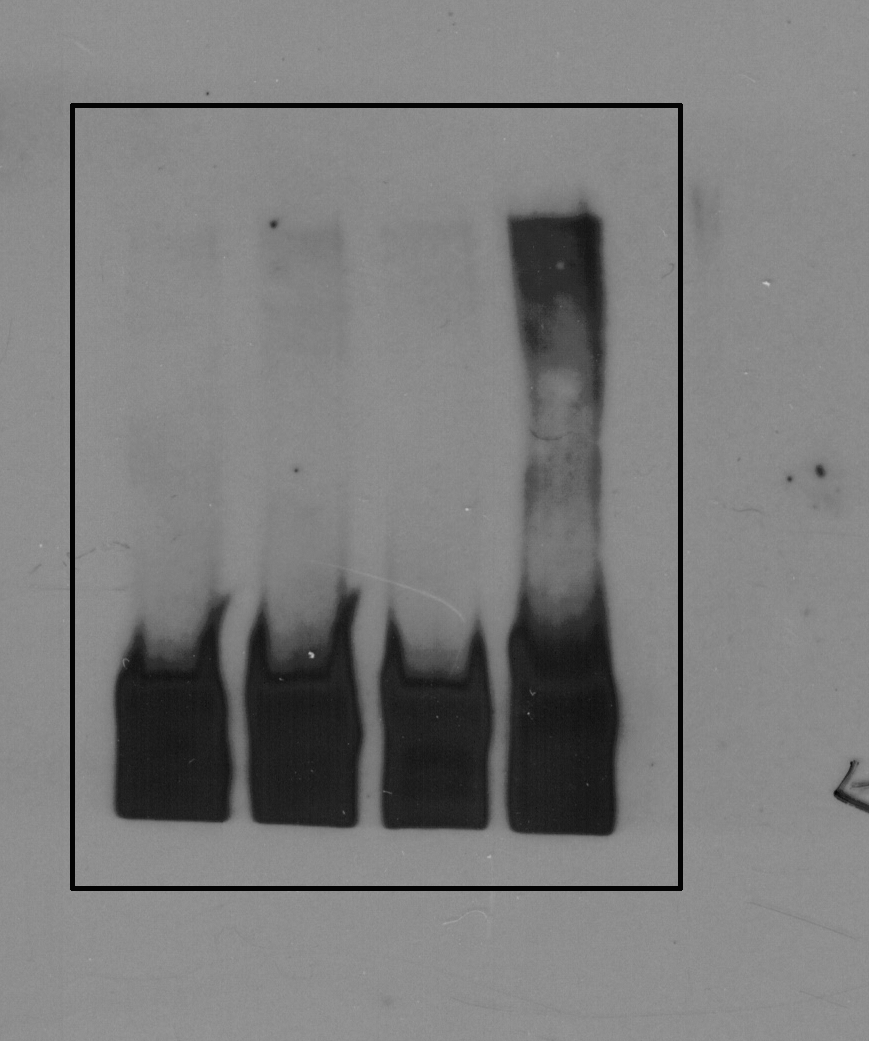

Supplement: Figure 5—source data 1. [file elife-86168-fig5-data1.zip › Figure 5 source data 1/annotated/Fig.5G EZH2-UB IP Anti-GFP-EZH2.tif]

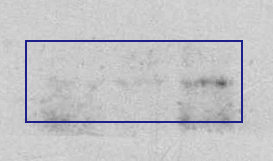

Supplement: Figure 5—source data 1. [file elife-86168-fig5-data1.zip › Figure 5 source data 1/annotated/Fig.5A 20210811 mouse brain e14 e18 p0 check EZH2 K20me anti-ezh2-s21p uncropped.tif]

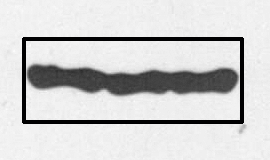

Supplement: Figure 5—source data 1. [file elife-86168-fig5-data1.zip › Figure 5 source data 1/annotated/Fig.5A mouse brain e14 e18 p0 check EZH2 K20me anti-H3 uncropped.tif]

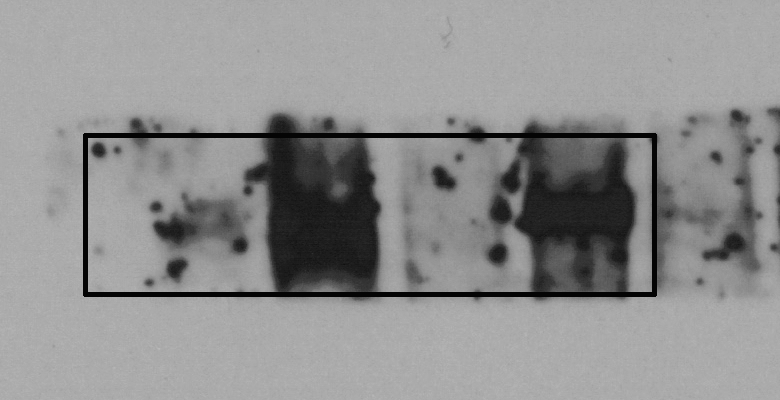

Supplement: Figure 5—source data 1. [file elife-86168-fig5-data1.zip › Figure 5 source data 1/annotated/Fig.5C 20200306 L3MBTL3 ip anti-L3.tif]

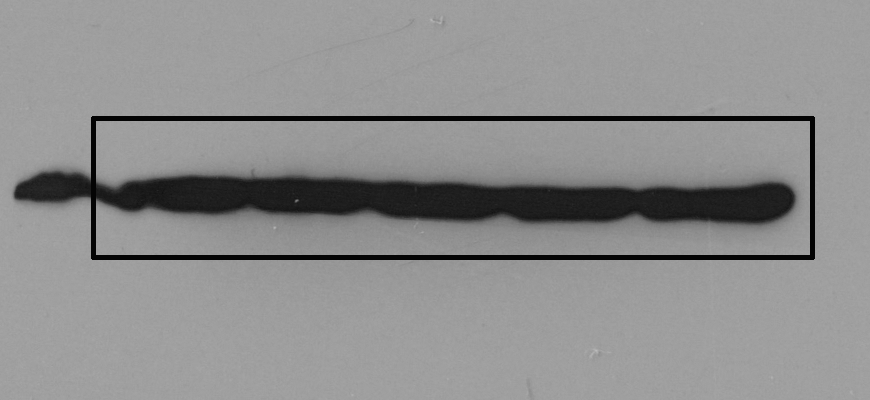

Supplement: Figure 5—source data 1. [file elife-86168-fig5-data1.zip › Figure 5 source data 1/annotated/Fig.5E 20200710 h1299-ezh2 ip-ha input Actin.tif]

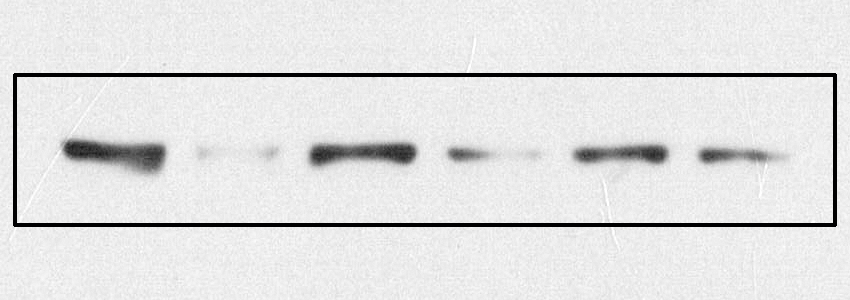

Supplement: Figure 5—source data 1. [file elife-86168-fig5-data1.zip › Figure 5 source data 1/annotated/Fig.5F G401 EZH2 WT K20R S21A SI LSD1-2 Anti-EZH2 Uncropped.tif]

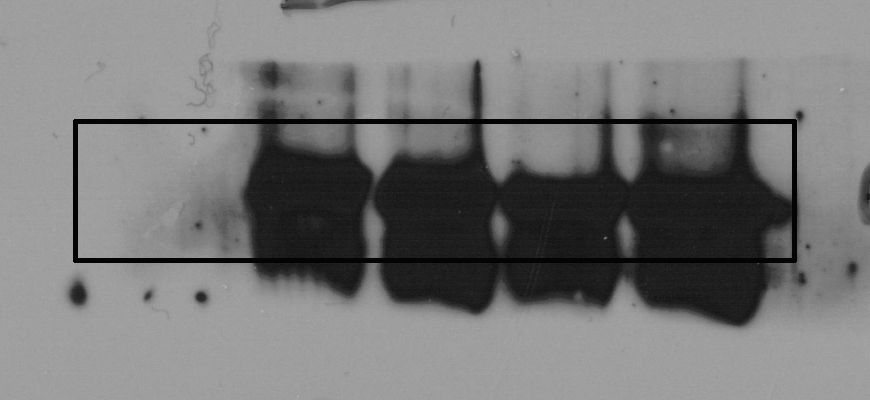

Supplement: Figure 5—source data 1. [file elife-86168-fig5-data1.zip › Figure 5 source data 1/annotated/Fig.5E 20200710 h1299-ezh2 ip-ha IP ha-ezh2.tif]

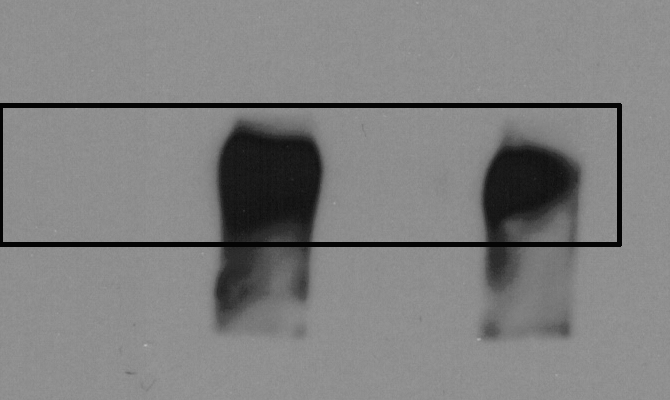

Supplement: Figure 5—source data 1. [file elife-86168-fig5-data1.zip › Figure 5 source data 1/annotated/Fig.5H ezh2 wt k20r ub input anti-flag-D5.tif]

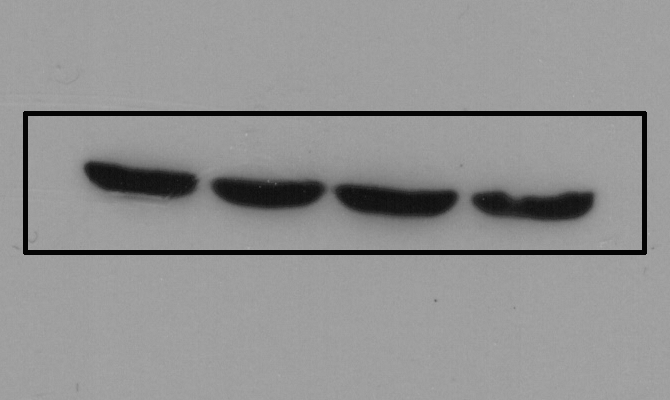

Supplement: Figure 5—source data 1. [file elife-86168-fig5-data1.zip › Figure 5 source data 1/annotated/Fig.5G EZH2-UB Anti-Actin.tif]

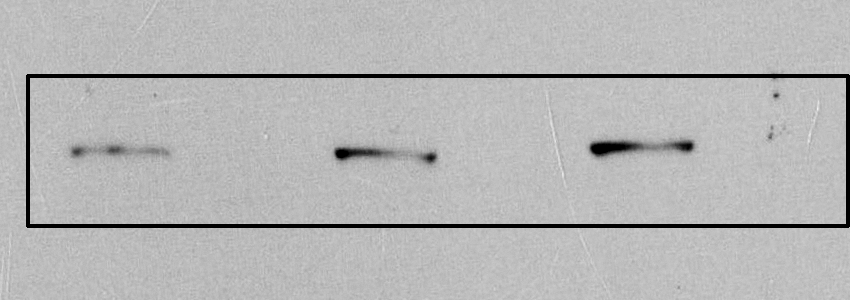

Supplement: Figure 5—source data 1. [file elife-86168-fig5-data1.zip › Figure 5 source data 1/annotated/Fig.5F G401 EZH2 WT K20R S21A SI LSD1-2 Anti-EZH2-K20me Uncropped.tif]

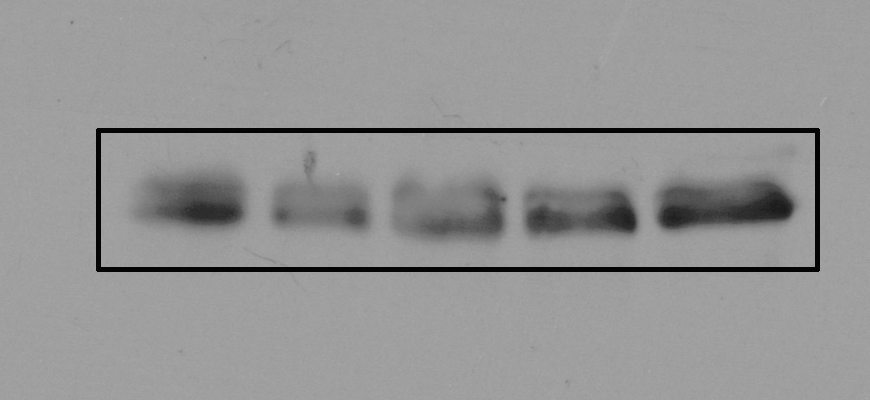

Supplement: Figure 5—source data 1. [file elife-86168-fig5-data1.zip › Figure 5 source data 1/annotated/Fig.5E 20200710 h1299-ezh2 ip-ha input L3MBTL3.tif]

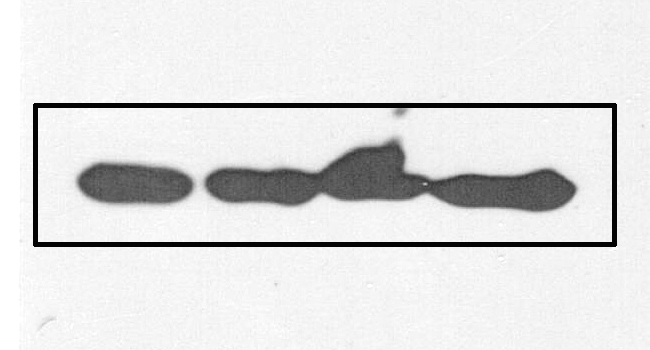

Supplement: Figure 5—source data 1. [file elife-86168-fig5-data1.zip › Figure 5 source data 1/annotated/Fig.5D 20220502 293t l3 ip with ezh2 transfect with set7 wt h297a anti-actin uncropped.tif]

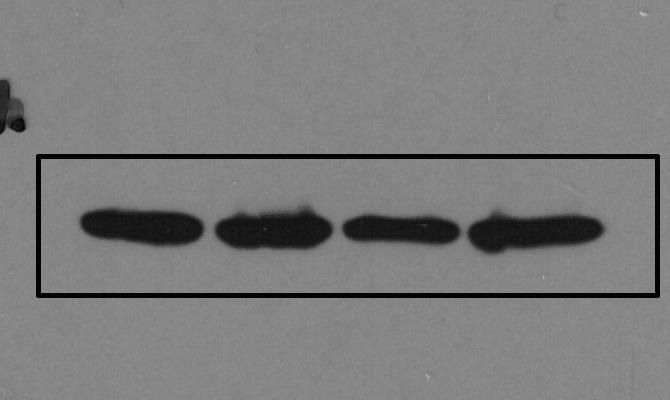

Supplement: Figure 5—source data 1. [file elife-86168-fig5-data1.zip › Figure 5 source data 1/annotated/Fig.5G EZH2-UB Anti-EGFP-EZH2.tif]

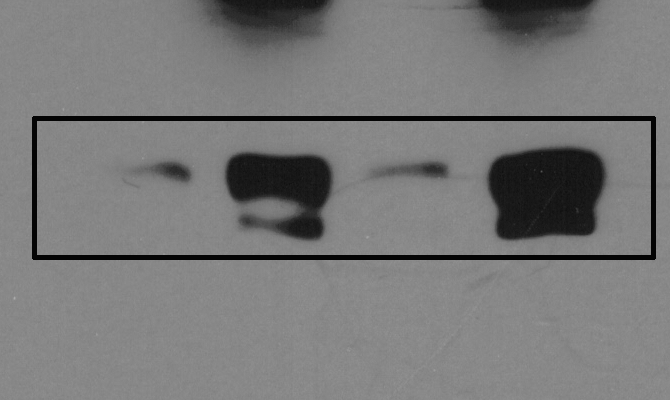

Supplement: Figure 5—source data 1. [file elife-86168-fig5-data1.zip › Figure 5 source data 1/annotated/Fig.5H ezh2 wt k20r ub input anti-L3.tif]

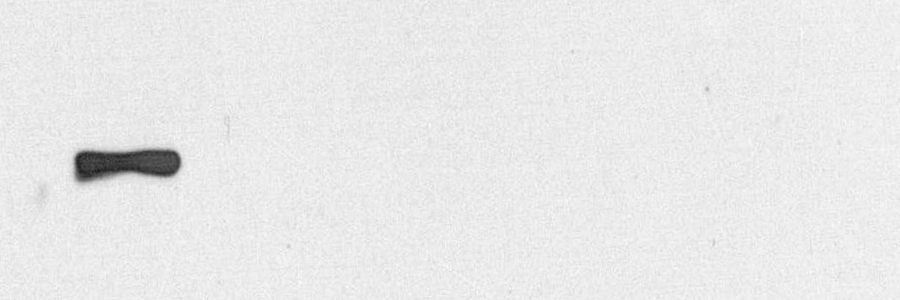

Supplement: Figure 6—source data 1. [file elife-86168-fig6-data1.zip › Figure 6 source data 1/Figure6C PA1 MK2206 0-6 UM anti-ps473AKT uncropped.tif]

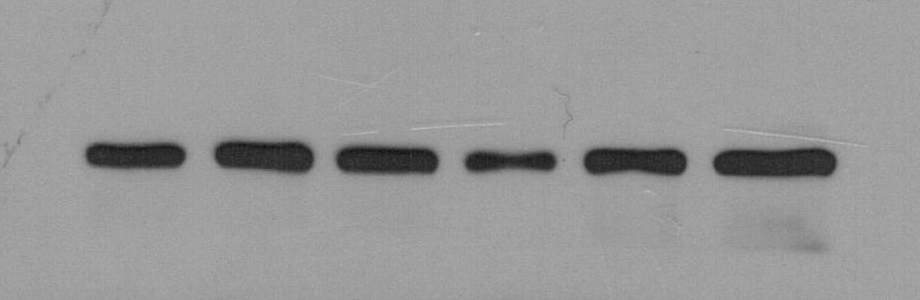

Supplement: Figure 6—source data 1. [file elife-86168-fig6-data1.zip › Figure 6 source data 1/Fig.6F ezh2-k20r akt mk2206 5h anti-ezh2 uncropped.tif]

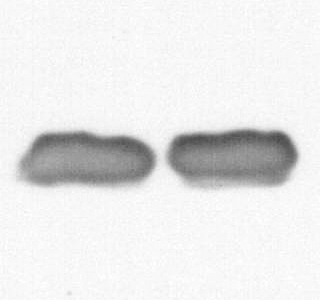

Supplement: Figure 6—source data 1. [file elife-86168-fig6-data1.zip › Figure 6 source data 1/Fig.6B mef wt +mk2206 check k20me s21p anti-akt uncropped.tif]

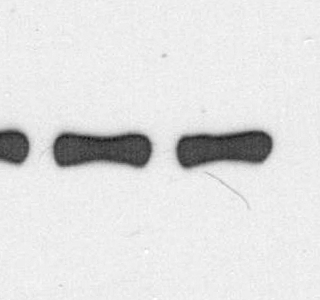

Supplement: Figure 6—source data 1. [file elife-86168-fig6-data1.zip › Figure 6 source data 1/Fig.6E l3-ko mef treat with AKT inhibitor anti-EZH2 UNcropped.tif]

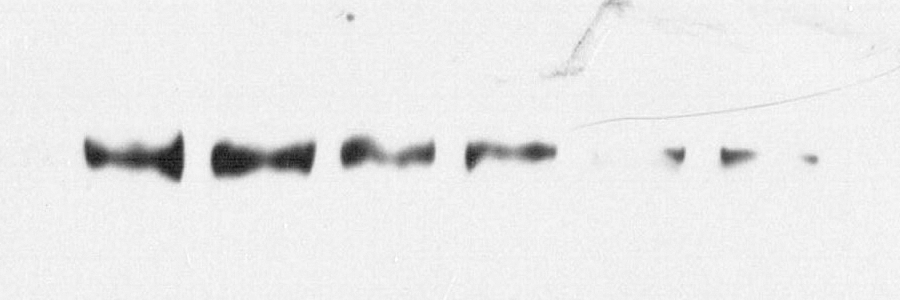

Supplement: Figure 6—source data 1. [file elife-86168-fig6-data1.zip › Figure 6 source data 1/Figure6C PA1 MK2206 0-6 UM anti-ezh2 uncropped.tif]

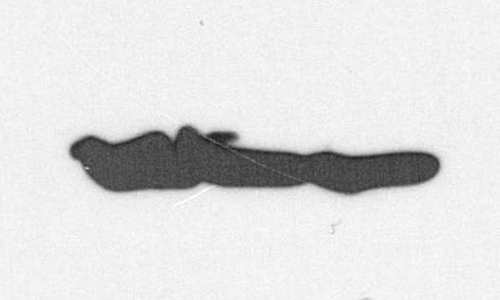

Supplement: Figure 6—source data 1. [file elife-86168-fig6-data1.zip › Figure 6 source data 1/Fig.6H mef wt k2or ip ezh2 with h3 input anti-H3 Uncropped.tif]

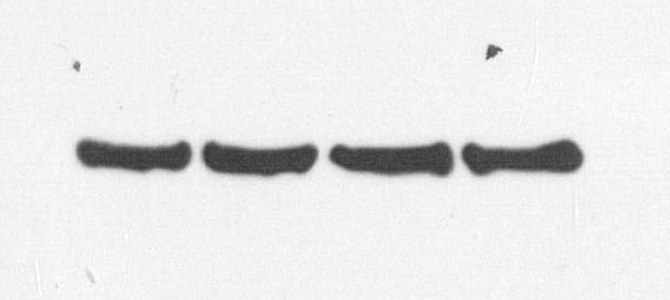

Supplement: Figure 6—source data 1. [file elife-86168-fig6-data1.zip › Figure 6 source data 1/Fig.6G MEF-EZH2 HET HOMO P1 P2 P3 check EZH2 Anti-actin Uncropped.tif]

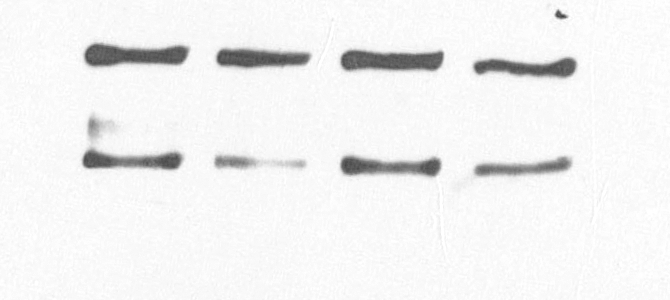

Supplement: Figure 6—source data 1. [file elife-86168-fig6-data1.zip › Figure 6 source data 1/Fig.6G MEF-EZH2 HET HOMO P1 P2 P3 check EZH2 Anti-pS473-akt Uncropped.tif]

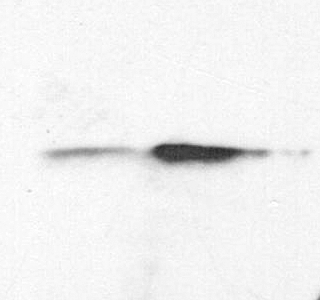

Supplement: Figure 6—source data 1. [file elife-86168-fig6-data1.zip › Figure 6 source data 1/Fig.6B mef wt +mk2206 check k20me s21p anti-EZH2-K20me uncropped.tif]

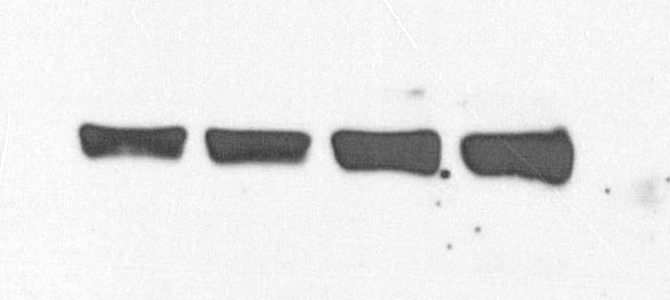

Supplement: Figure 6—source data 1. [file elife-86168-fig6-data1.zip › Figure 6 source data 1/Fig.6G MEF-EZH2 HET HOMO P1 P2 P3 check EZH2 Anti-akt Uncropped.tif]

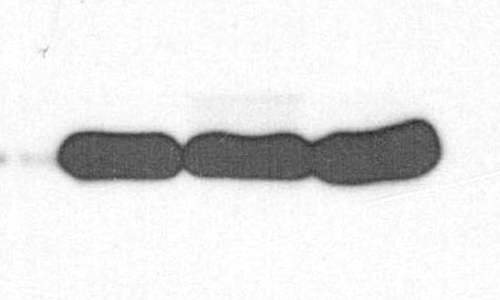

Supplement: Figure 6—source data 1. [file elife-86168-fig6-data1.zip › Figure 6 source data 1/Fig.6H MEF-K20R IP EZH2 With suz12 eed input anti-ezh2 uncropped.tif]

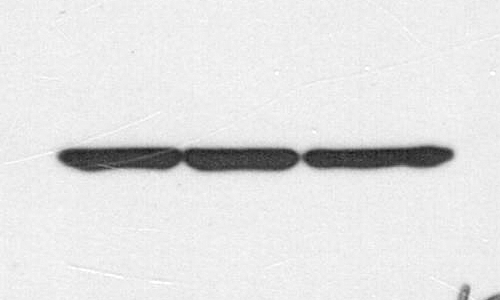

Supplement: Figure 6—source data 1. [file elife-86168-fig6-data1.zip › Figure 6 source data 1/Fig.6I mef-k20r ip ezh2 with h3 input anti-AKT uncropped.tif]

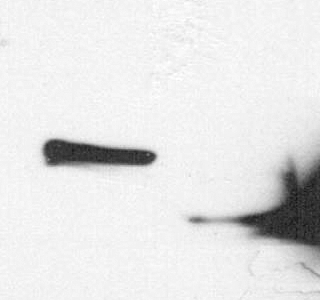

Supplement: Figure 6—source data 1. [file elife-86168-fig6-data1.zip › Figure 6 source data 1/Fig.6B mef wt +mk2206 check k20me s21p anti-pS473-akt uncropped.tif]

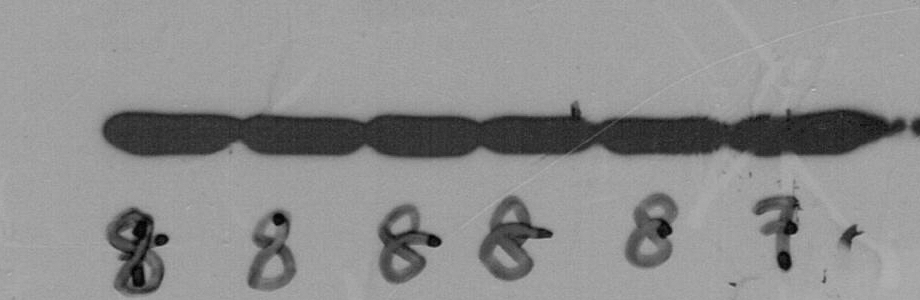

Supplement: Figure 6—source data 1. [file elife-86168-fig6-data1.zip › Figure 6 source data 1/Fig.6F ezh2-k20r akt mk2206 5h anti-actin uncropped.tif]

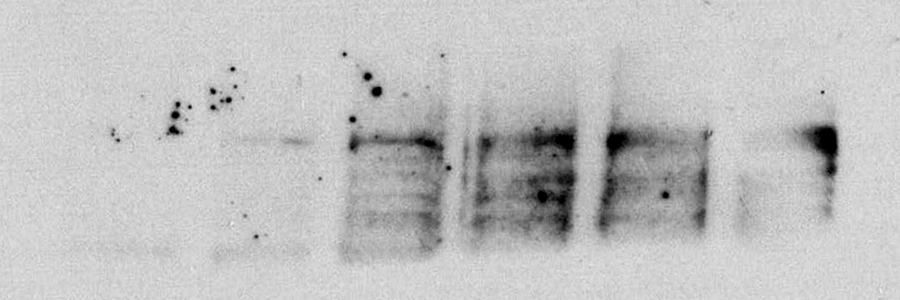

Supplement: Figure 6—source data 1. [file elife-86168-fig6-data1.zip › Figure 6 source data 1/Figure6C PA1 MK2206 0-6 UM anti-ezh2k20me uncropped.tif]

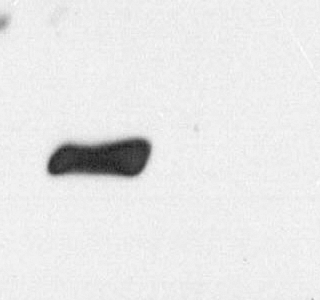

Supplement: Figure 6—source data 1. [file elife-86168-fig6-data1.zip › Figure 6 source data 1/Fig.6A t47d treated with mk2206 4 um anti-pS473akt uncropped.tif]

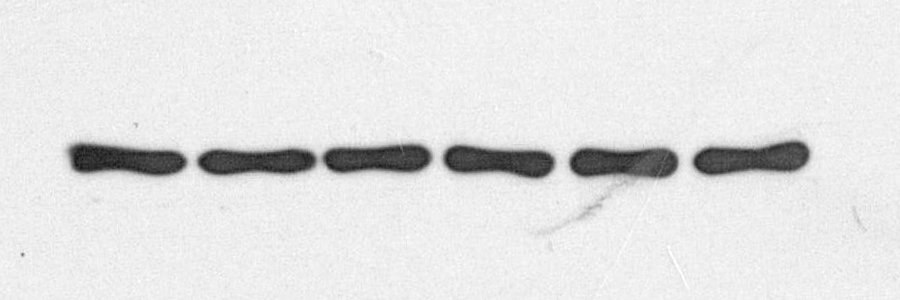

Supplement: Figure 6—source data 1. [file elife-86168-fig6-data1.zip › Figure 6 source data 1/Figure6C PA1 MK2206 0-6 UM anti-AKT uncropped.tif]

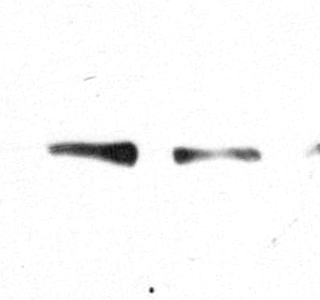

Supplement: Figure 6—source data 1. [file elife-86168-fig6-data1.zip › Figure 6 source data 1/Fig.6D t47d transfection with pkh3-l3 d5 treat with mk2206 for 4h anti-H3K27me3 uncropped.tif]

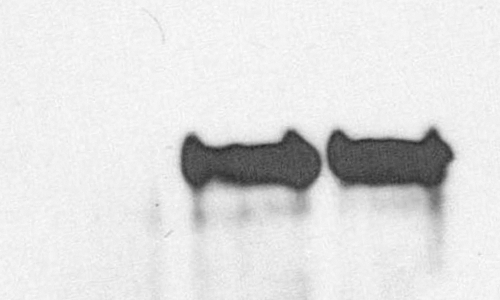

Supplement: Figure 6—source data 1. [file elife-86168-fig6-data1.zip › Figure 6 source data 1/Fig.6H MEF-K20R IP EZH2 With suz12 eed ip anti-eed 1 uncropped.tif]

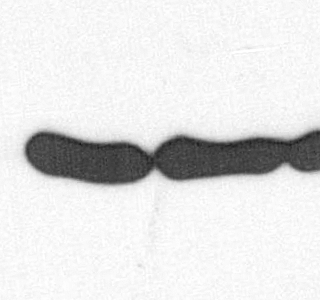

Supplement: Figure 6—source data 1. [file elife-86168-fig6-data1.zip › Figure 6 source data 1/Fig.6D t47d transfection with pkh3-l3 d5 treat with mk2206 for 4h anti-HA-l3 uncropped.tif]

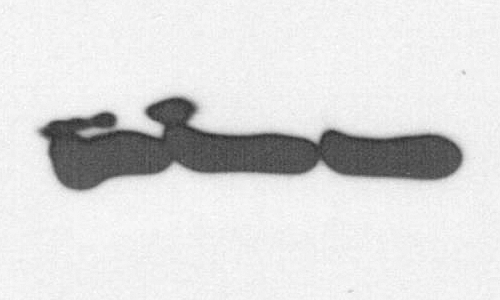

Supplement: Figure 6—source data 1. [file elife-86168-fig6-data1.zip › Figure 6 source data 1/Fig.6H mef wt k2or ip ezh2 with h3 input anti-actin Uncropped.tif]

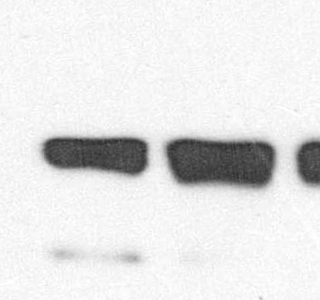

Supplement: Figure 6—source data 1. [file elife-86168-fig6-data1.zip › Figure 6 source data 1/Fig.6D t47d transfection with pkh3-l3 d5 treat with mk2206 for 4h anti-Akt uncropped.tif]

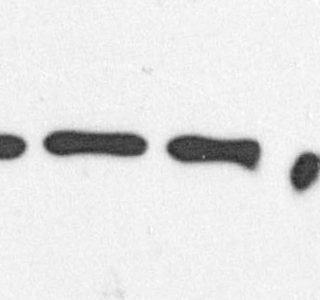

Supplement: Figure 6—source data 1. [file elife-86168-fig6-data1.zip › Figure 6 source data 1/Fig.6A t47d treated with mk2206 4 um anti-ezh2 uncropped.tif]

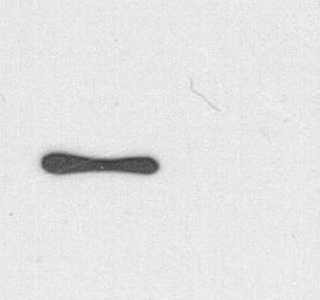

Supplement: Figure 6—source data 1. [file elife-86168-fig6-data1.zip › Figure 6 source data 1/Fig.6E l3-ko mef treat with AKT inhibitor anti-pS473akt UNcropped.tif]

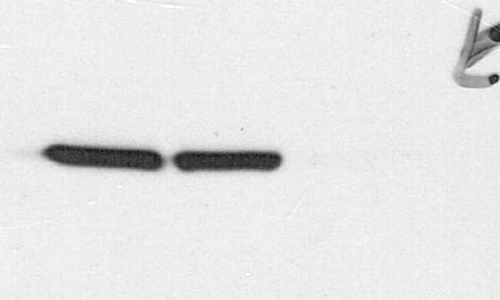

Supplement: Figure 6—source data 1. [file elife-86168-fig6-data1.zip › Figure 6 source data 1/Fig.6I mef-k20r ip ezh2 with h3 input anti-pS473AKT uncropped.tif]

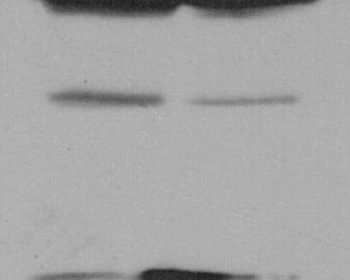

Supplement: Figure 6—source data 1. [file elife-86168-fig6-data1.zip › Figure 6 source data 1/Fig.6B mef wt +mk2206 check k20me s21p anti-H3-k27me3 uncropped.tif]

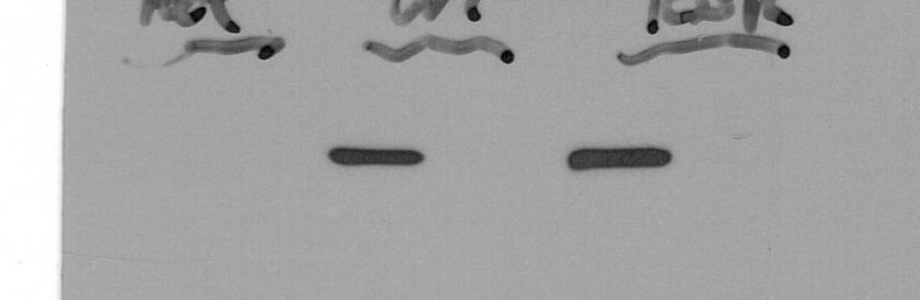

Supplement: Figure 6—source data 1. [file elife-86168-fig6-data1.zip › Figure 6 source data 1/Fig.6F ezh2-k20r akt mk2206 5h anti-akt-phosphation uncropped.tif]

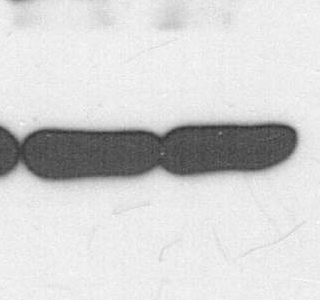

Supplement: Figure 6—source data 1. [file elife-86168-fig6-data1.zip › Figure 6 source data 1/Fig.6El3-ko mef treat with AKT inhibitor anti-H3 UNcropped.tif]

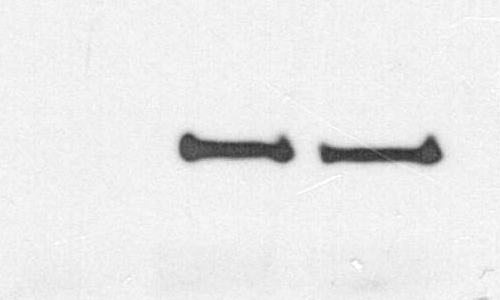

Supplement: Figure 6—source data 1. [file elife-86168-fig6-data1.zip › Figure 6 source data 1/Fig.6H MEF-K20R IP EZH2 With suz12 eed ip anti-suz12 uncropped.tif]

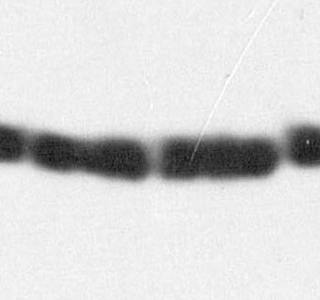

Supplement: Figure 6—source data 1. [file elife-86168-fig6-data1.zip › Figure 6 source data 1/Fig.6A t47d treated with mk2206 4 um anti-h3 uncropped.tif]

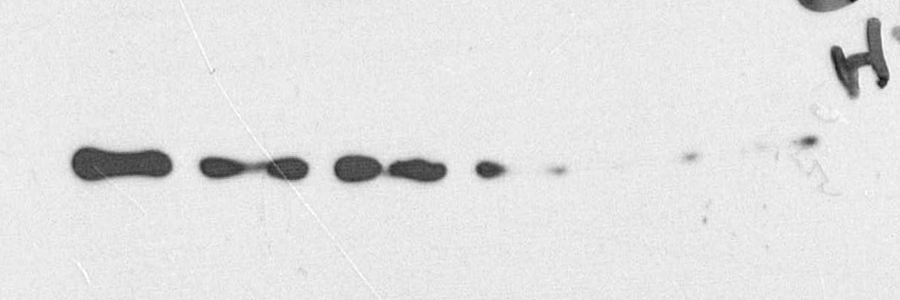

Supplement: Figure 6—source data 1. [file elife-86168-fig6-data1.zip › Figure 6 source data 1/Figure6C PA1 MK2206 0-6 UM anti-H3K27me3 uncropped.tif]

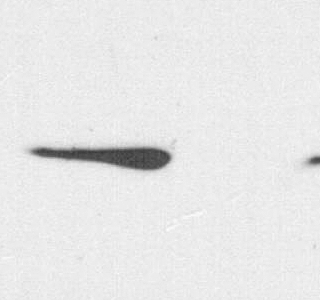

Supplement: Figure 6—source data 1. [file elife-86168-fig6-data1.zip › Figure 6 source data 1/Fig.6D t47d transfection with pkh3-l3 d5 treat with mk2206 for 4h anti-pS473Akt uncropped.tif]

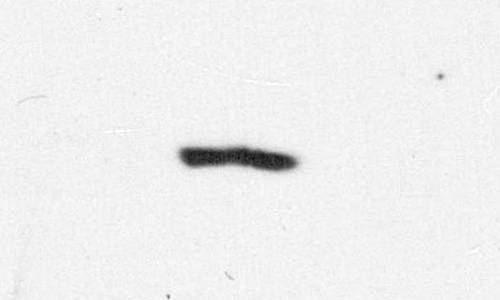

Supplement: Figure 6—source data 1. [file elife-86168-fig6-data1.zip › Figure 6 source data 1/Fig.6H mef wt k2or ip ezh2 with h3 ip anti-H3 Uncropped.tif]
