# Supplementary material for: A methylation-phosphorylation switch controls EZH2 stability and hematopoiesis
Source: eLife. 2024 Feb 12;13:e86168. doi: 10.7554/eLife.86168 (PMC10901513; doi:10.7554/eLife.86168)

Figure 6A-H3K27me3

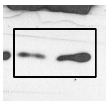

Figure 6A-H3

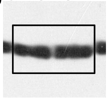

Figure 6A-AKT

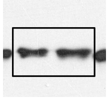

Figure 6A-pS473-AKT

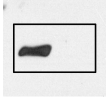

Figure 6A-EZH2

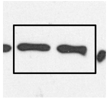

Figure 6A-Actin

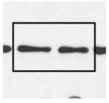

Figure 6B-H3K27me3

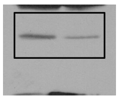

Figure 6B-H3

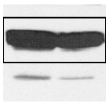

Figure 6B-EZH2-K20me

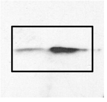

Figure 6B-EZH2-S21p

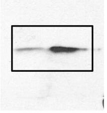

Figure 6B-EZH2

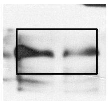

Figure 6B-pS473-AKT

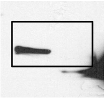

Figure 6B-pan-AKT

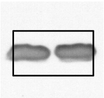

Figure 6C-ZH2

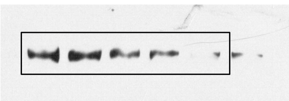

Figure 6C-EZH2-K20me

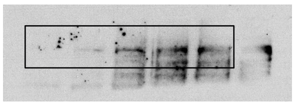

Figure 6C-EZH2-S21p

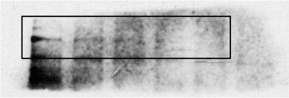

Figure 6C-p-AKT

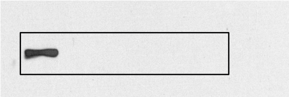

Figure 6C-AKT

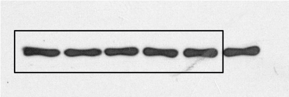

Figure 6C-H3K27

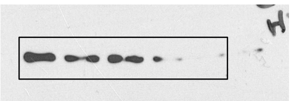

Figure 6C-H3

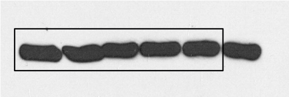

Figure 6D-H3K27me3

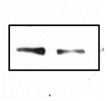

Figure 6D-H3

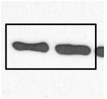

Figure 6D-EZH2

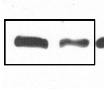

Figure 6D-HA-L3MBTL3

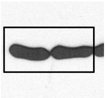

Figure 6D-pS473-AKT

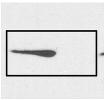

Figure 6D-AKT

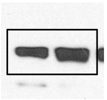

Figure 6E-H3K27me3

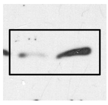

Figure 6E-H3

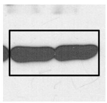

Figure 6E-EZH2

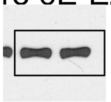

Figure 6E-pS473-AKT

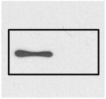

Figure 6E-AKT

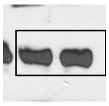

Figure 6F-H3K27me3

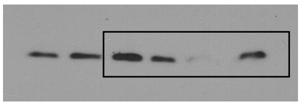

Figure 6F-H3

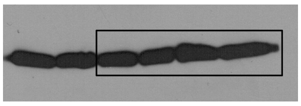

Figure 6F-EZH2

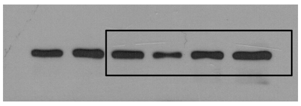

Figure 6F-pS473-AKT

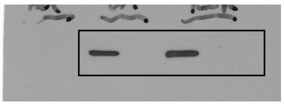

Figure 6F-AKT

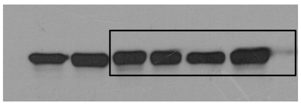

Figure 6F-Actin

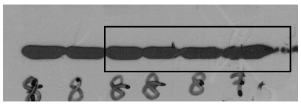

Figure 6G-H3K27me3

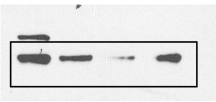

Figure 6G-H3

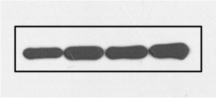

Figure 6G-EZH2

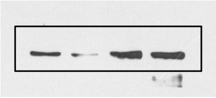

Figure 6G-pS473-AKT

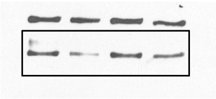

Figure 6G-AKT

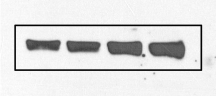

Figure 6G-Actin

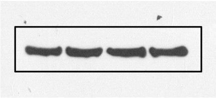

Left panel

IP

Figure 6H-EZH2

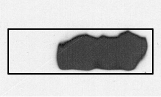

Figure 6H-H3

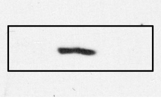

Input

Figure 6H-EZH2

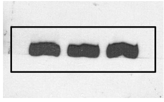

Figure 6H-H3

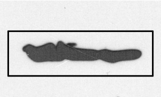

Figure 6H-Actin

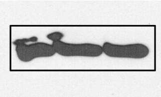

Right panel

IP

Figure 6H-EZH2

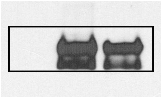

Figure 6H-EED

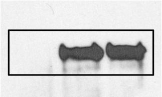

Figure 6H-SUZ12

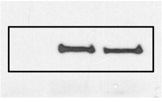

Input

Figure 6H-EZH2

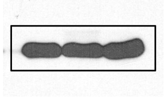

Figure 6H-Actin

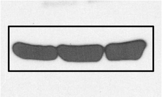

IP

Figure 6I-EZH2

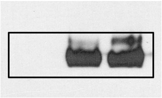

Figure 6I-H3

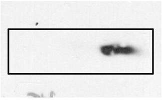

Input

Figure 6I-EZH2

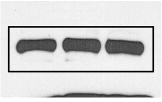

Figure 6I-AKT

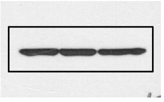

Figure 6I-H3

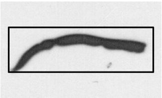

Figure 6I-pS473-AKT

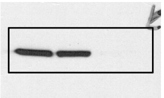

Figure 6I-AKT

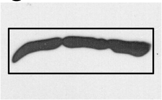

Supplement: Figure 6—source data 1. [file elife-86168-fig6-data1.zip › Figure 6 source data 1/Figure 6-annotated source data .pdf]
