# Supplementary figures and images for: A methylation-phosphorylation switch controls EZH2 stability and hematopoiesis (part 5 of 7)
Source: eLife. 2024 Feb 12;13:e86168. doi: 10.7554/eLife.86168 (PMC10901513; doi:10.7554/eLife.86168)

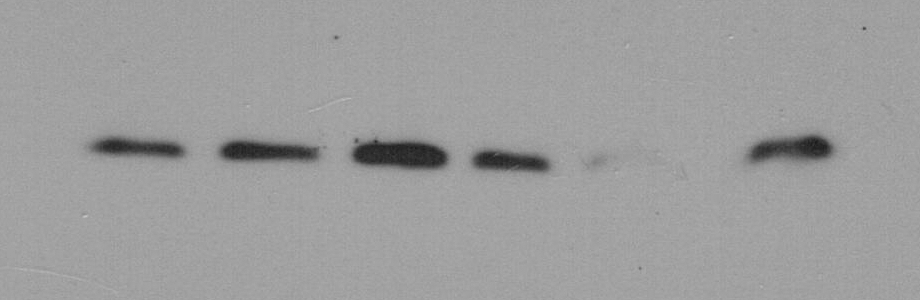

Supplement: Figure 6—source data 1. [file elife-86168-fig6-data1.zip › Figure 6 source data 1/Fig.6F ezh2-k20r akt mk2206 5h anti-h3k27me3 uncropped.tif]

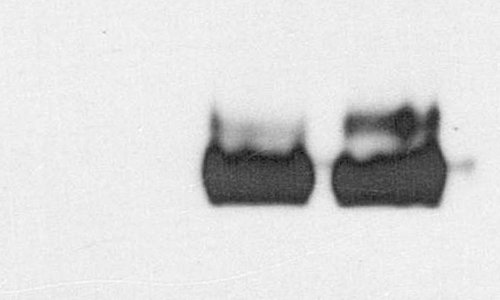

Supplement: Figure 6—source data 1. [file elife-86168-fig6-data1.zip › Figure 6 source data 1/Fig.6I mef-k20r ip ezh2 with h3 iP anti-EZH2 uncropped.tif]

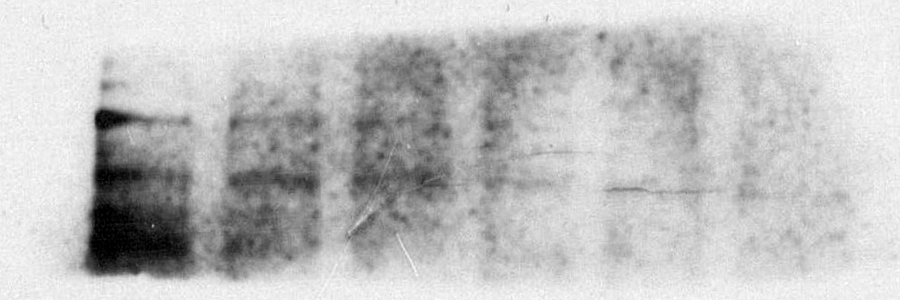

Supplement: Figure 6—source data 1. [file elife-86168-fig6-data1.zip › Figure 6 source data 1/Figure6C PA1 MK2206 0-6 UM anti-ezh2s21p uncropped.tif]

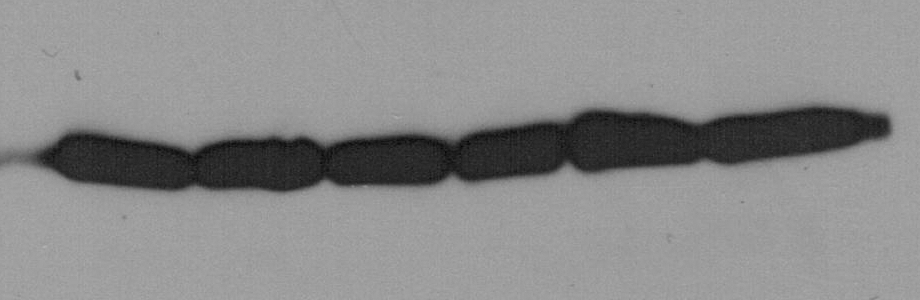

Supplement: Figure 6—source data 1. [file elife-86168-fig6-data1.zip › Figure 6 source data 1/Fig.6F ezh2-k20r akt mk2206 5h anti-H3 uncropped.tif]

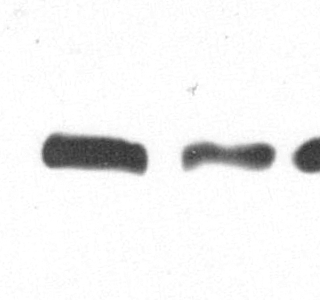

Supplement: Figure 6—source data 1. [file elife-86168-fig6-data1.zip › Figure 6 source data 1/Fig.6D t47d transfection with pkh3-l3 d5 treat with mk2206 for 4h anti-EZH2 uncropped.tif]

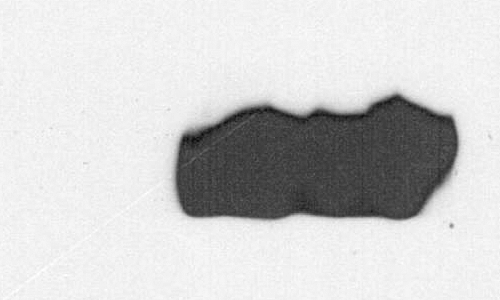

Supplement: Figure 6—source data 1. [file elife-86168-fig6-data1.zip › Figure 6 source data 1/Fig.6H mef wt k2or ip ezh2 with h3 ip anti-EZH2 Uncropped.tif]

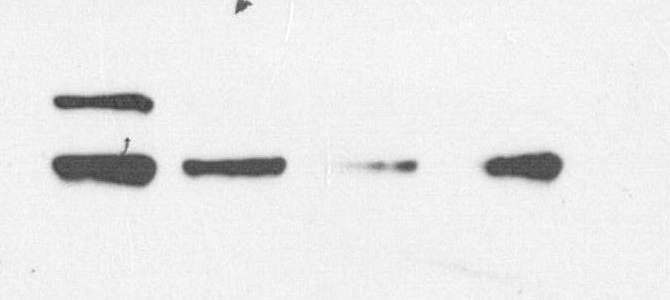

Supplement: Figure 6—source data 1. [file elife-86168-fig6-data1.zip › Figure 6 source data 1/Fig.6G MEF-EZH2 HET HOMO P1 P2 P3 check EZH2 Anti-H3K27me3 2 Uncropped.tif]

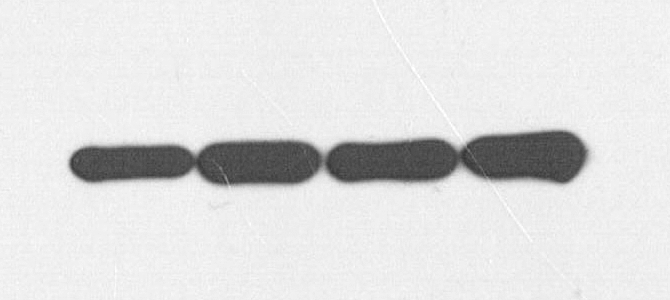

Supplement: Figure 6—source data 1. [file elife-86168-fig6-data1.zip › Figure 6 source data 1/Fig.6G MEF-EZH2 HET HOMO P1 P2 P3 check EZH2 Anti-H3 Uncropped.tif]

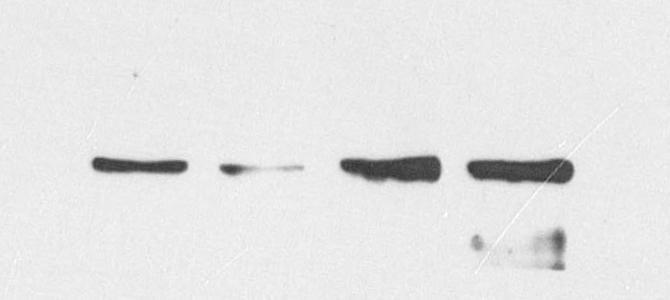

Supplement: Figure 6—source data 1. [file elife-86168-fig6-data1.zip › Figure 6 source data 1/Fig.6G MEF-EZH2 HET HOMO P1 P2 P3 check EZH2 Anti-EZH2 Uncropped.tif]

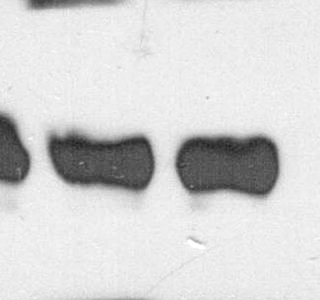

Supplement: Figure 6—source data 1. [file elife-86168-fig6-data1.zip › Figure 6 source data 1/Fig.6E l3-ko mef treat with AKT inhibitor anti-akt UNcropped.tif]

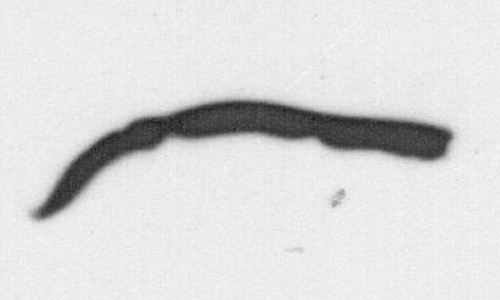

Supplement: Figure 6—source data 1. [file elife-86168-fig6-data1.zip › Figure 6 source data 1/Fig.6I mef-k20r ip ezh2 with h3 input anti-H3 uncropped.tif]

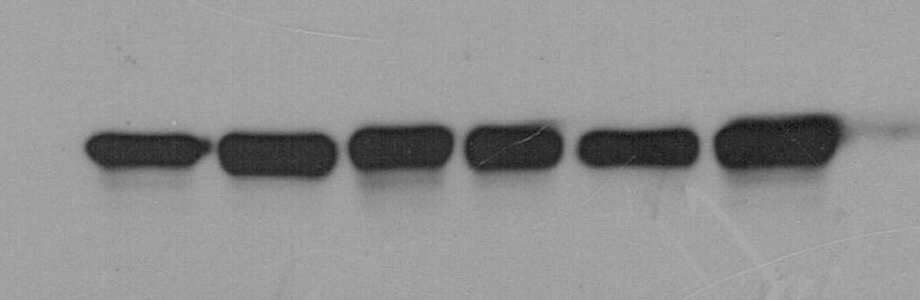

Supplement: Figure 6—source data 1. [file elife-86168-fig6-data1.zip › Figure 6 source data 1/Fig.6F ezh2-k20r akt mk2206 5h anti-akt-pan uncropped.tif]

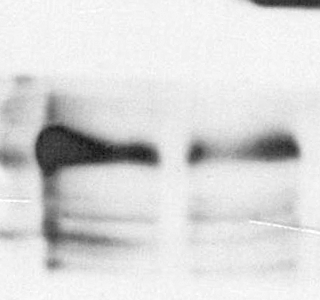

Supplement: Figure 6—source data 1. [file elife-86168-fig6-data1.zip › Figure 6 source data 1/Fig.6B mef wt +mk2206 check k20me s21p anti-EZH2 uncropped.tif]

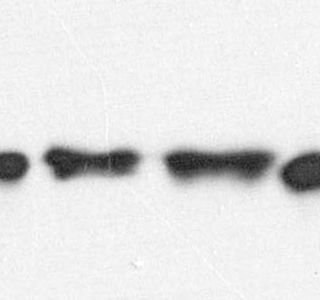

Supplement: Figure 6—source data 1. [file elife-86168-fig6-data1.zip › Figure 6 source data 1/Fi.6A t47d treated with mk2206 4 um anti-akt uncropped.tif]

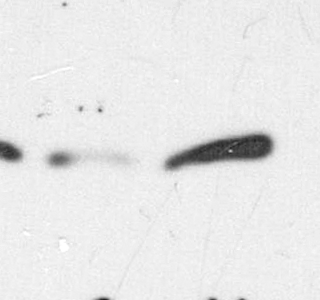

Supplement: Figure 6—source data 1. [file elife-86168-fig6-data1.zip › Figure 6 source data 1/Fig.6Emef treat with AKT inhibitor anti-H3K27me3 UNcropped.tif]

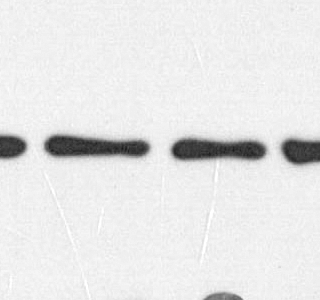

Supplement: Figure 6—source data 1. [file elife-86168-fig6-data1.zip › Figure 6 source data 1/Fig.6A t47d treated with mk2206 4 um anti-actin uncropped.tif]

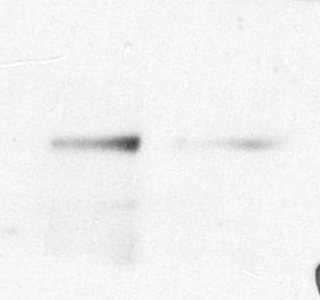

Supplement: Figure 6—source data 1. [file elife-86168-fig6-data1.zip › Figure 6 source data 1/Fig.6B mef wt +mk2206 check k20me s21p anti-EZH2-S21P uncropped.tif]

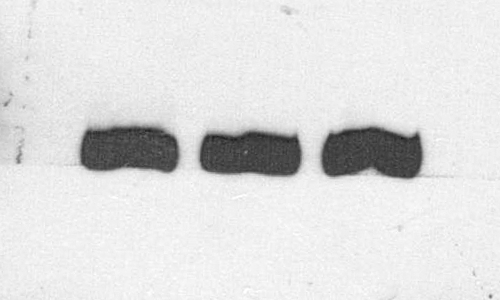

Supplement: Figure 6—source data 1. [file elife-86168-fig6-data1.zip › Figure 6 source data 1/Fig.6H mef wt k2or ip ezh2 with h3 input anti-EZH2 Uncropped.tif]

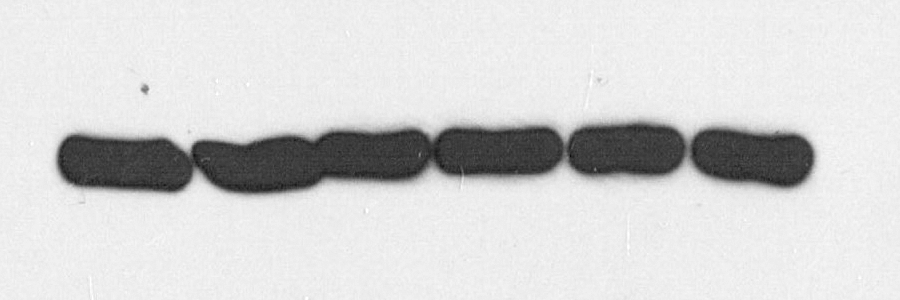

Supplement: Figure 6—source data 1. [file elife-86168-fig6-data1.zip › Figure 6 source data 1/Figure6C PA1 MK2206 0-6 UM anti-H3 uncropped.tif]

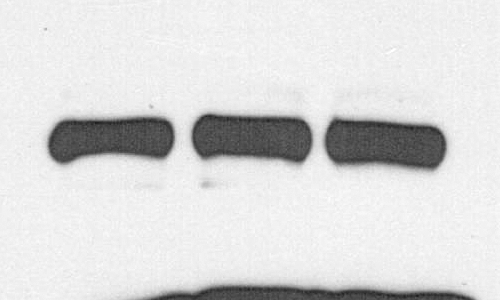

Supplement: Figure 6—source data 1. [file elife-86168-fig6-data1.zip › Figure 6 source data 1/Fig.6I mef-k20r ip ezh2 with h3 input anti-EZH2 uncropped.tif]

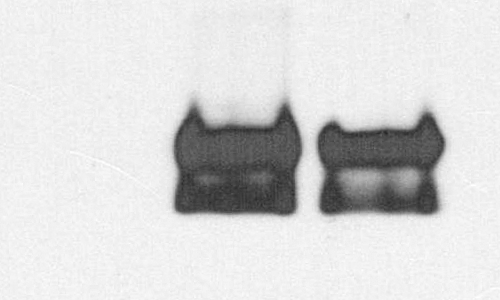

Supplement: Figure 6—source data 1. [file elife-86168-fig6-data1.zip › Figure 6 source data 1/Fig.6H MEF-K20R IP EZH2 With suz12 eed ip anti-ezh2 uncropped.tif]

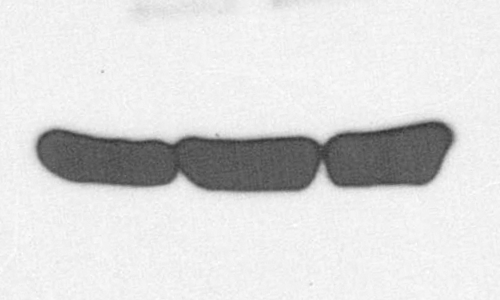

Supplement: Figure 6—source data 1. [file elife-86168-fig6-data1.zip › Figure 6 source data 1/Fig.6H MEF-K20R IP EZH2 With suz12 eed input anti-actin uncropped.tif]

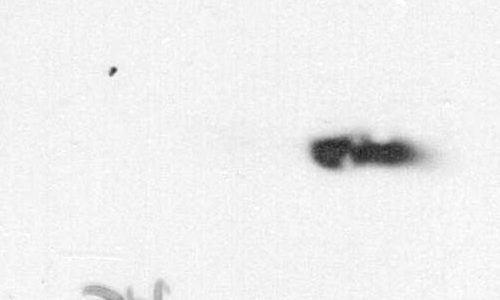

Supplement: Figure 6—source data 1. [file elife-86168-fig6-data1.zip › Figure 6 source data 1/Fig.6I mef-k20r ip ezh2 with h3 iP anti-H3 uncropped.tif]

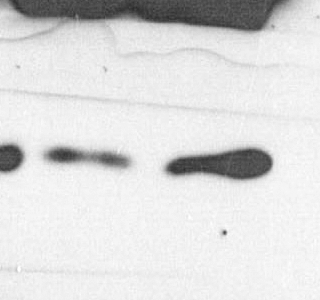

Supplement: Figure 6—source data 1. [file elife-86168-fig6-data1.zip › Figure 6 source data 1/Fig.6A t47d treated with mk2206 4 um anti-H3K27ME3 uncropped.tif]

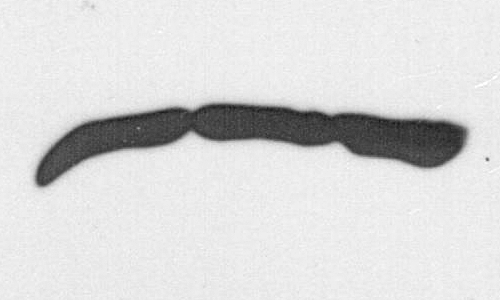

Supplement: Figure 6—source data 1. [file elife-86168-fig6-data1.zip › Figure 6 source data 1/Fig.6I mef-k20r ip ezh2 with h3 input anti-actin uncropped.tif]

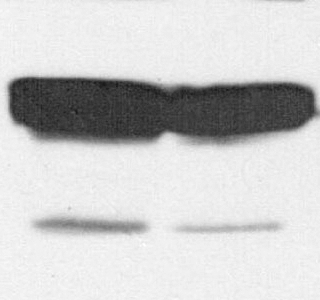

Supplement: Figure 6—source data 1. [file elife-86168-fig6-data1.zip › Figure 6 source data 1/Fig.6B mef wt +mk2206 check k20me s21p anti-H3 uncropped.tif]

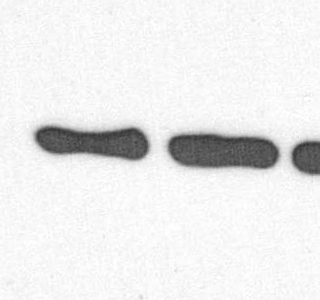

Supplement: Figure 6—source data 1. [file elife-86168-fig6-data1.zip › Figure 6 source data 1/Fig.6D t47d transfection with pkh3-l3 d5 treat with mk2206 for 4h anti-H3 uncropped.tif]

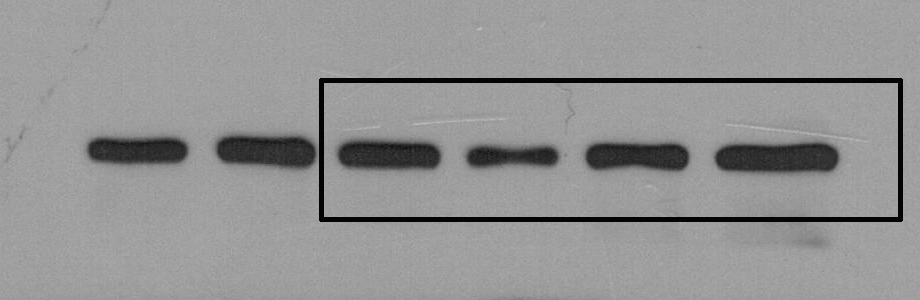

Supplement: Figure 6—source data 1. [file elife-86168-fig6-data1.zip › Figure 6 source data 1/annotated/Fig.6F ezh2-k20r akt mk2206 5h anti-ezh2 uncropped.tif]

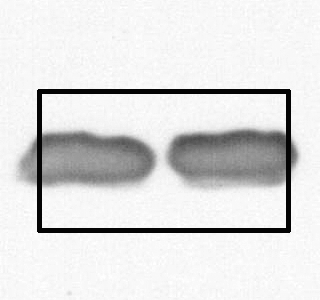

Supplement: Figure 6—source data 1. [file elife-86168-fig6-data1.zip › Figure 6 source data 1/annotated/Fig.6B mef wt +mk2206 check k20me s21p anti-akt uncropped.tif]

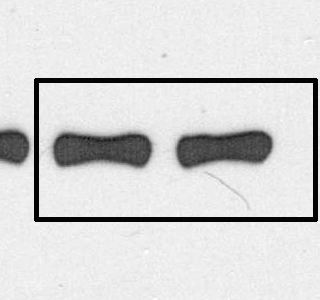

Supplement: Figure 6—source data 1. [file elife-86168-fig6-data1.zip › Figure 6 source data 1/annotated/Fig.6E l3-ko mef treat with AKT inhibitor anti-EZH2 UNcropped.tif]

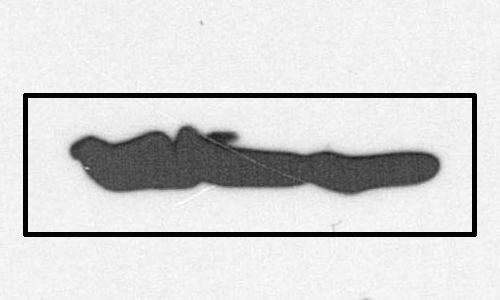

Supplement: Figure 6—source data 1. [file elife-86168-fig6-data1.zip › Figure 6 source data 1/annotated/Fig.6H mef wt k2or ip ezh2 with h3 input anti-H3 Uncropped.tif]

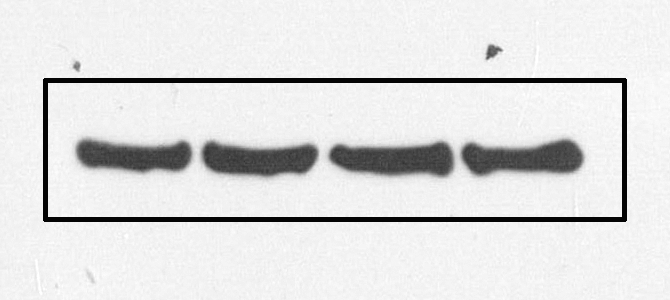

Supplement: Figure 6—source data 1. [file elife-86168-fig6-data1.zip › Figure 6 source data 1/annotated/Fig.6G MEF-EZH2 HET HOMO P1 P2 P3 check EZH2 Anti-actin Uncropped.tif]

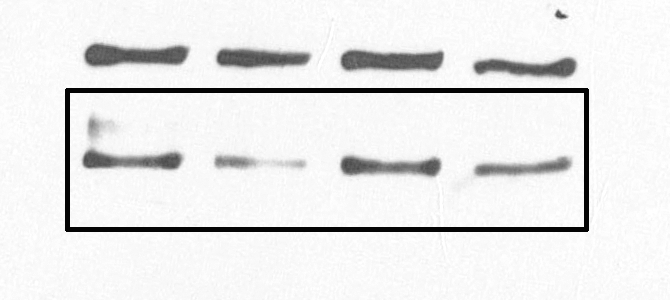

Supplement: Figure 6—source data 1. [file elife-86168-fig6-data1.zip › Figure 6 source data 1/annotated/Fig.6G MEF-EZH2 HET HOMO P1 P2 P3 check EZH2 Anti-pS473-akt Uncropped.tif]

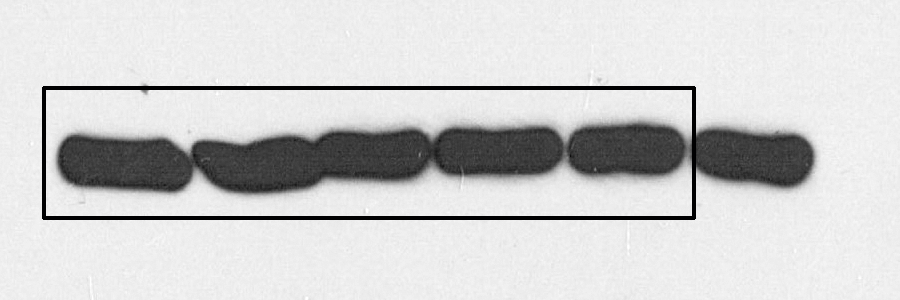

Supplement: Figure 6—source data 1. [file elife-86168-fig6-data1.zip › Figure 6 source data 1/annotated/Figure6C PA1 MK2206 0-6 UM anti-H3 uncropped.tif]

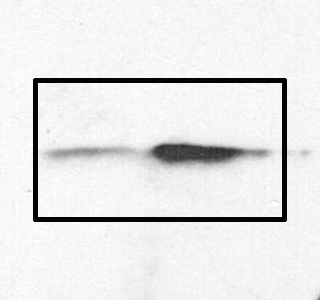

Supplement: Figure 6—source data 1. [file elife-86168-fig6-data1.zip › Figure 6 source data 1/annotated/Fig.6B mef wt +mk2206 check k20me s21p anti-EZH2-K20me uncropped.tif]

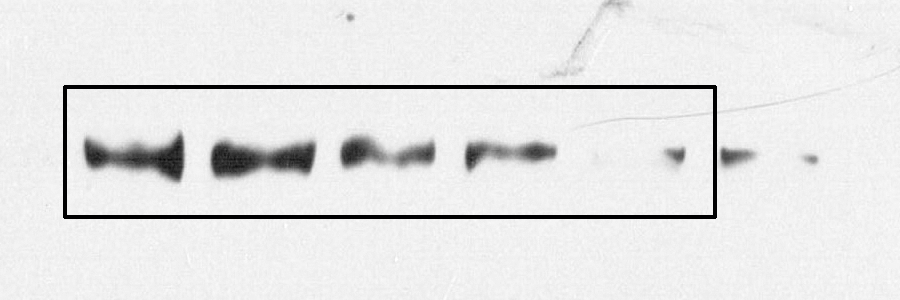

Supplement: Figure 6—source data 1. [file elife-86168-fig6-data1.zip › Figure 6 source data 1/annotated/Figure6C PA1 MK2206 0-6 UM anti-ezh2 uncropped.tif]

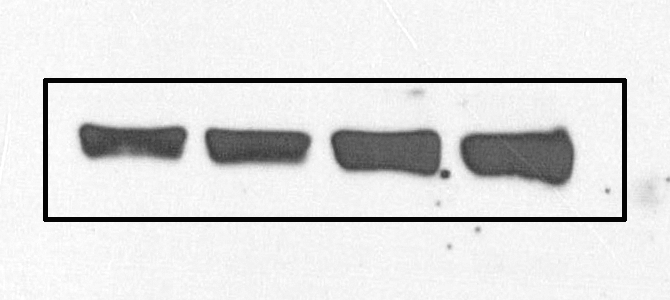

Supplement: Figure 6—source data 1. [file elife-86168-fig6-data1.zip › Figure 6 source data 1/annotated/Fig.6G MEF-EZH2 HET HOMO P1 P2 P3 check EZH2 Anti-akt Uncropped.tif]

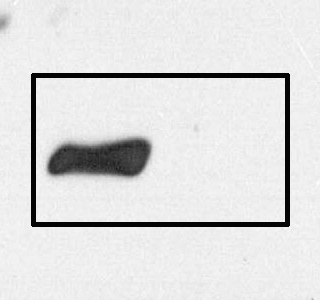

Supplement: Figure 6—source data 1. [file elife-86168-fig6-data1.zip › Figure 6 source data 1/annotated/Fig.6A t47d treated with mk2206 4 um anti-pS473akt uncropped.tif]

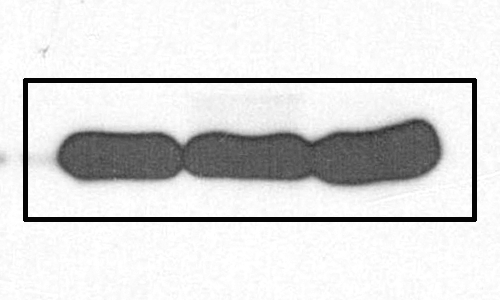

Supplement: Figure 6—source data 1. [file elife-86168-fig6-data1.zip › Figure 6 source data 1/annotated/Fig.6H MEF-K20R IP EZH2 With suz12 eed input anti-ezh2 uncropped.tif]

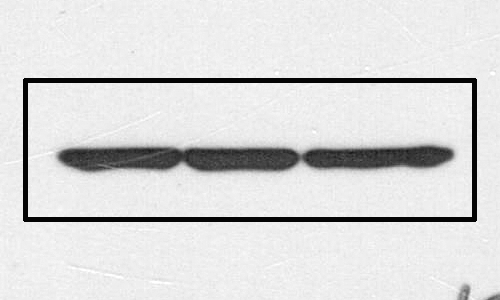

Supplement: Figure 6—source data 1. [file elife-86168-fig6-data1.zip › Figure 6 source data 1/annotated/Fig.6I mef-k20r ip ezh2 with h3 input anti-AKT uncropped.tif]

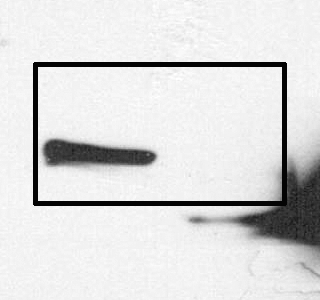

Supplement: Figure 6—source data 1. [file elife-86168-fig6-data1.zip › Figure 6 source data 1/annotated/Fig.6B mef wt +mk2206 check k20me s21p anti-pS473-akt uncropped.tif]

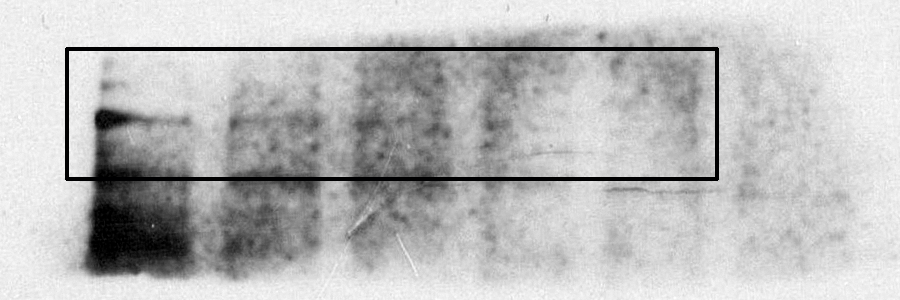

Supplement: Figure 6—source data 1. [file elife-86168-fig6-data1.zip › Figure 6 source data 1/annotated/Figure6C PA1 MK2206 0-6 UM anti-ezh2s21p uncropped.tif]

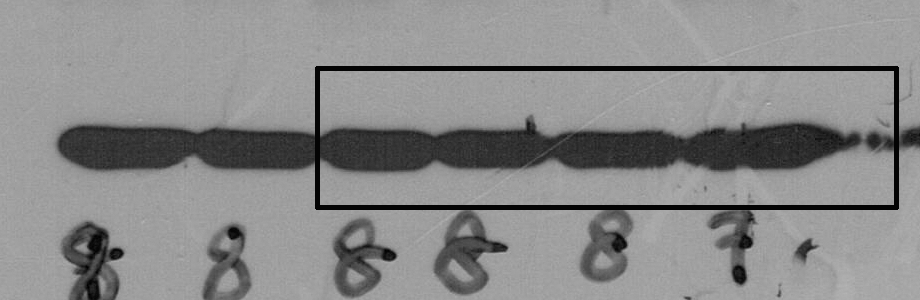

Supplement: Figure 6—source data 1. [file elife-86168-fig6-data1.zip › Figure 6 source data 1/annotated/Fig.6F ezh2-k20r akt mk2206 5h anti-actin uncropped.tif]

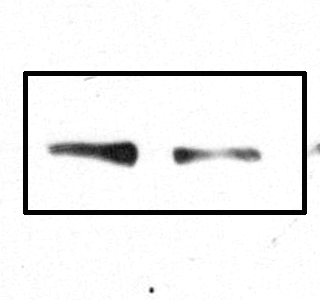

Supplement: Figure 6—source data 1. [file elife-86168-fig6-data1.zip › Figure 6 source data 1/annotated/Fig.6D t47d transfection with pkh3-l3 d5 treat with mk2206 for 4h anti-H3K27me3 uncropped.tif]

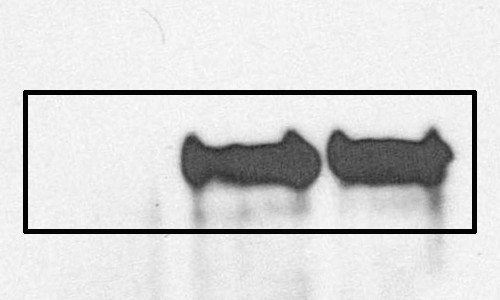

Supplement: Figure 6—source data 1. [file elife-86168-fig6-data1.zip › Figure 6 source data 1/annotated/Fig.6H MEF-K20R IP EZH2 With suz12 eed ip anti-eed 1 uncropped.tif]

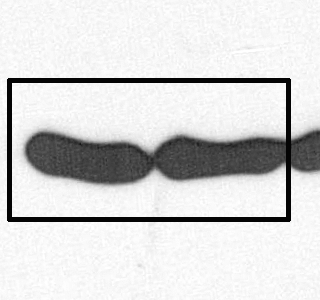

Supplement: Figure 6—source data 1. [file elife-86168-fig6-data1.zip › Figure 6 source data 1/annotated/Fig.6D t47d transfection with pkh3-l3 d5 treat with mk2206 for 4h anti-HA-l3 uncropped.tif]

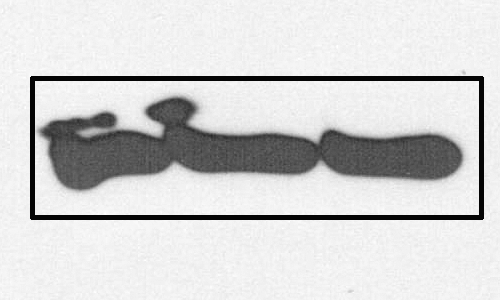

Supplement: Figure 6—source data 1. [file elife-86168-fig6-data1.zip › Figure 6 source data 1/annotated/Fig.6H mef wt k2or ip ezh2 with h3 input anti-actin Uncropped.tif]

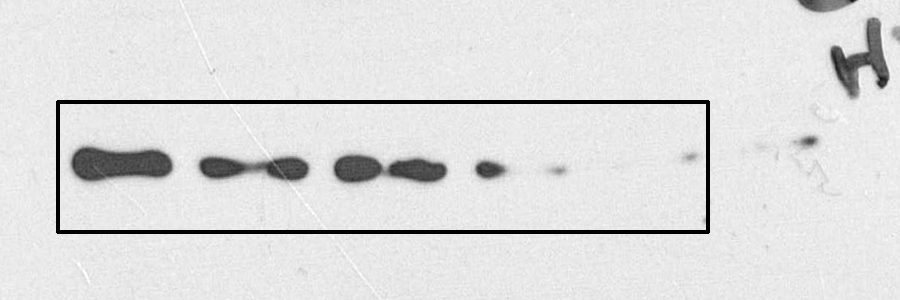

Supplement: Figure 6—source data 1. [file elife-86168-fig6-data1.zip › Figure 6 source data 1/annotated/Figure6C PA1 MK2206 0-6 UM anti-H3K27me3 uncropped.tif]

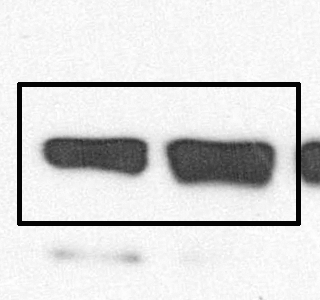

Supplement: Figure 6—source data 1. [file elife-86168-fig6-data1.zip › Figure 6 source data 1/annotated/Fig.6D t47d transfection with pkh3-l3 d5 treat with mk2206 for 4h anti-Akt uncropped.tif]

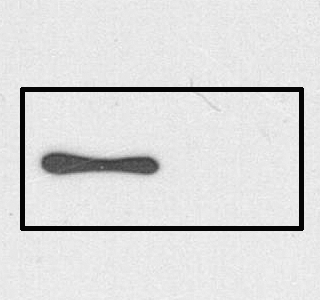

Supplement: Figure 6—source data 1. [file elife-86168-fig6-data1.zip › Figure 6 source data 1/annotated/Fig.6E l3-ko mef treat with AKT inhibitor anti-pS473akt UNcropped.tif]

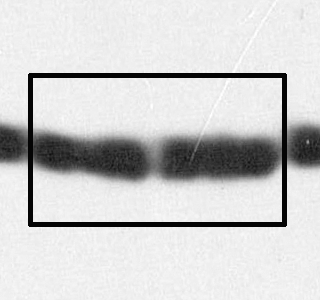

Supplement: Figure 6—source data 1. [file elife-86168-fig6-data1.zip › Figure 6 source data 1/annotated/Fig.6A t47d treated with mk2206 4 um anti-h3 uncropped.tif]

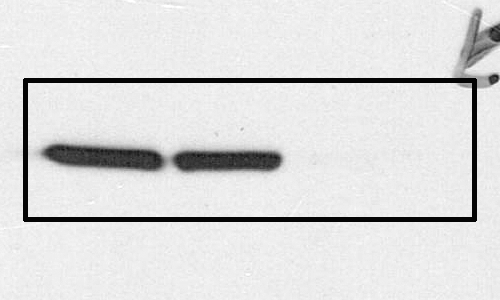

Supplement: Figure 6—source data 1. [file elife-86168-fig6-data1.zip › Figure 6 source data 1/annotated/Fig.6I mef-k20r ip ezh2 with h3 input anti-pS473AKT uncropped.tif]

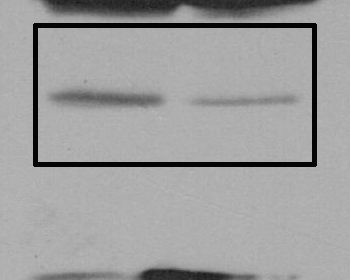

Supplement: Figure 6—source data 1. [file elife-86168-fig6-data1.zip › Figure 6 source data 1/annotated/Fig.6B mef wt +mk2206 check k20me s21p anti-H3-k27me3 uncropped.tif]

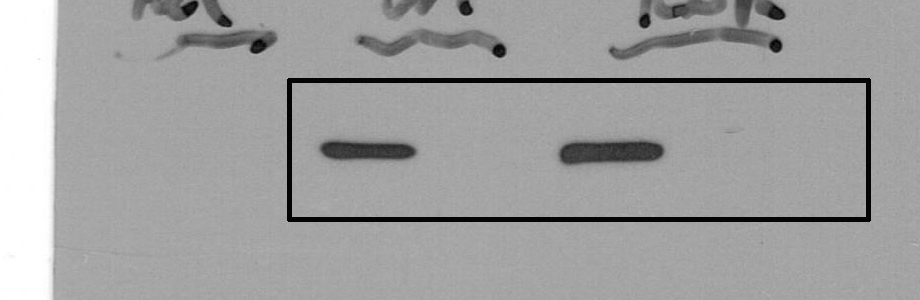

Supplement: Figure 6—source data 1. [file elife-86168-fig6-data1.zip › Figure 6 source data 1/annotated/Fig.6F ezh2-k20r akt mk2206 5h anti-akt-phosphation uncropped.tif]

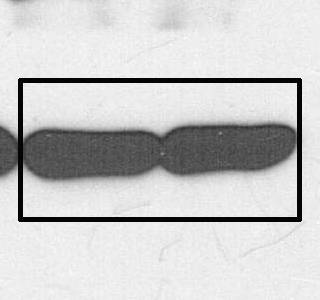

Supplement: Figure 6—source data 1. [file elife-86168-fig6-data1.zip › Figure 6 source data 1/annotated/Fig.6El3-ko mef treat with AKT inhibitor anti-H3 UNcropped.tif]

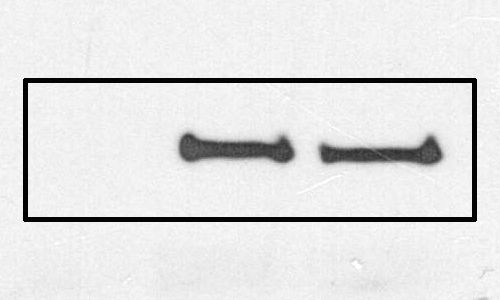

Supplement: Figure 6—source data 1. [file elife-86168-fig6-data1.zip › Figure 6 source data 1/annotated/Fig.6H MEF-K20R IP EZH2 With suz12 eed ip anti-suz12 uncropped.tif]

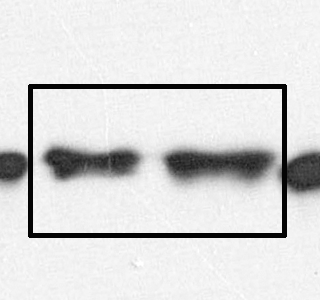

Supplement: Figure 6—source data 1. [file elife-86168-fig6-data1.zip › Figure 6 source data 1/annotated/Fig.6A t47d treated with mk2206 4 um anti-akt uncropped.tif]

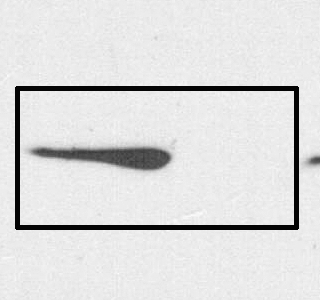

Supplement: Figure 6—source data 1. [file elife-86168-fig6-data1.zip › Figure 6 source data 1/annotated/Fig.6D t47d transfection with pkh3-l3 d5 treat with mk2206 for 4h anti-pS473Akt uncropped.tif]

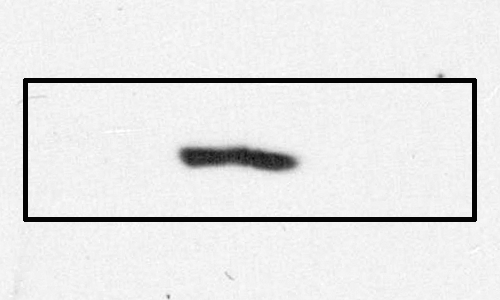

Supplement: Figure 6—source data 1. [file elife-86168-fig6-data1.zip › Figure 6 source data 1/annotated/Fig.6H mef wt k2or ip ezh2 with h3 ip anti-H3 Uncropped.tif]

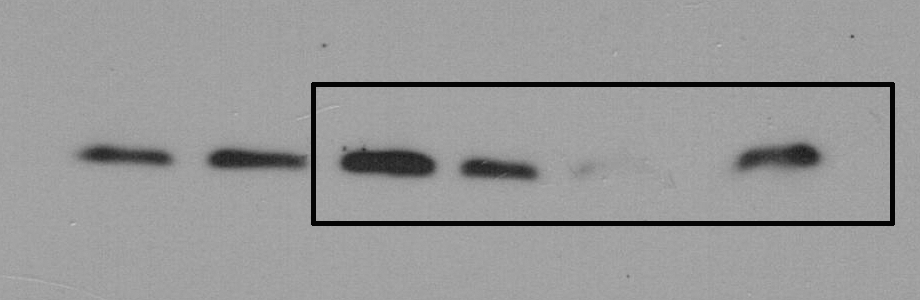

Supplement: Figure 6—source data 1. [file elife-86168-fig6-data1.zip › Figure 6 source data 1/annotated/Fig.6F ezh2-k20r akt mk2206 5h anti-h3k27me3 uncropped.tif]

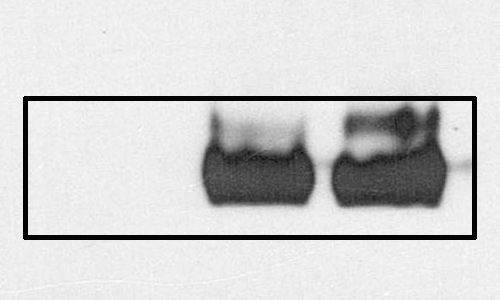

Supplement: Figure 6—source data 1. [file elife-86168-fig6-data1.zip › Figure 6 source data 1/annotated/Fig.6I mef-k20r ip ezh2 with h3 iP anti-EZH2 uncropped.tif]

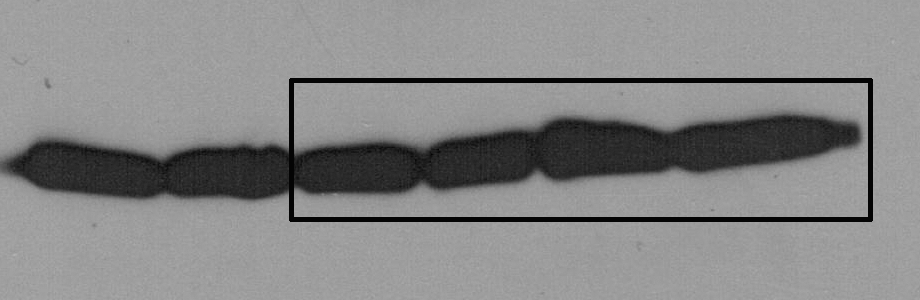

Supplement: Figure 6—source data 1. [file elife-86168-fig6-data1.zip › Figure 6 source data 1/annotated/Fig.6F ezh2-k20r akt mk2206 5h anti-H3 uncropped.tif]

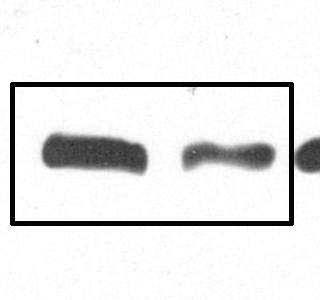

Supplement: Figure 6—source data 1. [file elife-86168-fig6-data1.zip › Figure 6 source data 1/annotated/Fig.6D t47d transfection with pkh3-l3 d5 treat with mk2206 for 4h anti-EZH2 uncropped.tif]

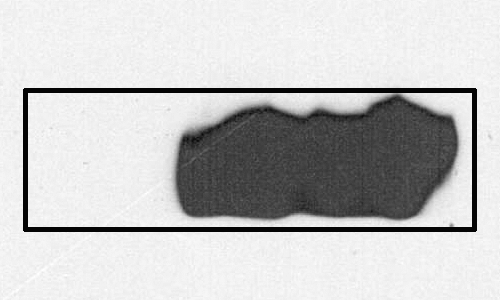

Supplement: Figure 6—source data 1. [file elife-86168-fig6-data1.zip › Figure 6 source data 1/annotated/Fig.6H mef wt k2or ip ezh2 with h3 ip anti-EZH2 Uncropped.tif]

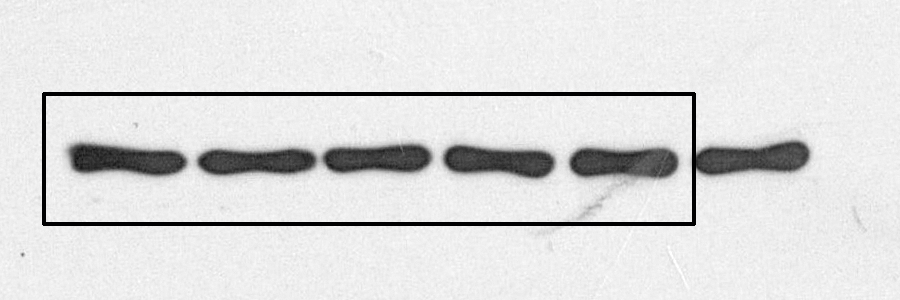

Supplement: Figure 6—source data 1. [file elife-86168-fig6-data1.zip › Figure 6 source data 1/annotated/Figure6C PA1 MK2206 0-6 UM anti-AKT uncropped.tif]

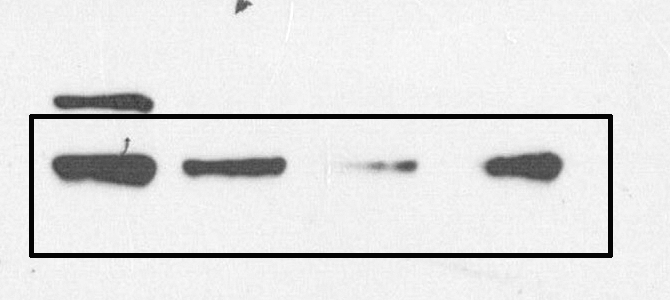

Supplement: Figure 6—source data 1. [file elife-86168-fig6-data1.zip › Figure 6 source data 1/annotated/Fig.6G MEF-EZH2 HET HOMO P1 P2 P3 check EZH2 Anti-H3K27me3 2 Uncropped.tif]

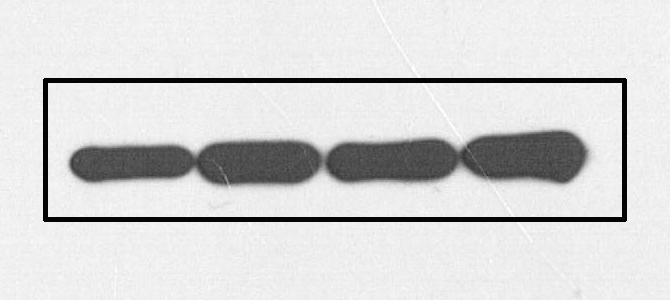

Supplement: Figure 6—source data 1. [file elife-86168-fig6-data1.zip › Figure 6 source data 1/annotated/Fig.6G MEF-EZH2 HET HOMO P1 P2 P3 check EZH2 Anti-H3 Uncropped.tif]

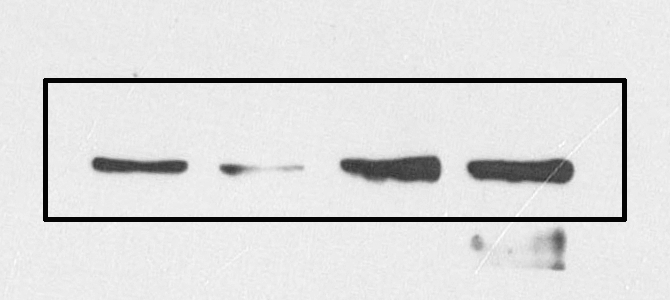

Supplement: Figure 6—source data 1. [file elife-86168-fig6-data1.zip › Figure 6 source data 1/annotated/Fig.6G MEF-EZH2 HET HOMO P1 P2 P3 check EZH2 Anti-EZH2 Uncropped.tif]

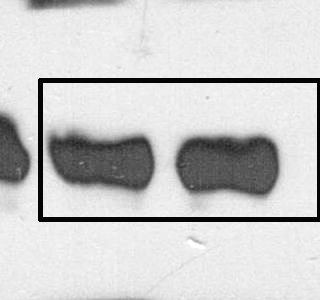

Supplement: Figure 6—source data 1. [file elife-86168-fig6-data1.zip › Figure 6 source data 1/annotated/Fig.6E l3-ko mef treat with AKT inhibitor anti-akt UNcropped.tif]

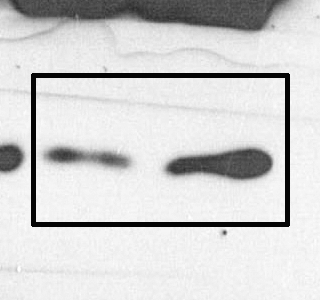

Supplement: Figure 6—source data 1. [file elife-86168-fig6-data1.zip › Figure 6 source data 1/annotated/Fig.6A t47d treated with mk2206 4 um anti-H3K27ME3 uncropped.tif]

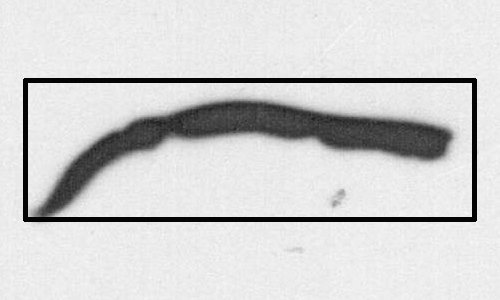

Supplement: Figure 6—source data 1. [file elife-86168-fig6-data1.zip › Figure 6 source data 1/annotated/Fig.6I mef-k20r ip ezh2 with h3 input anti-H3 uncropped.tif]

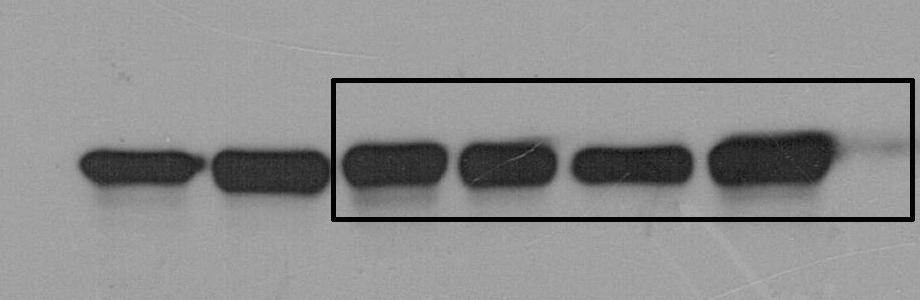

Supplement: Figure 6—source data 1. [file elife-86168-fig6-data1.zip › Figure 6 source data 1/annotated/Fig.6F ezh2-k20r akt mk2206 5h anti-akt-pan uncropped.tif]

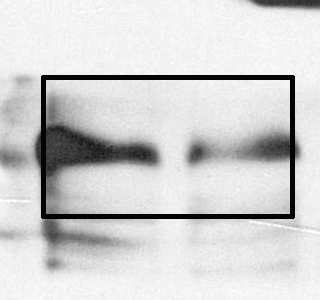

Supplement: Figure 6—source data 1. [file elife-86168-fig6-data1.zip › Figure 6 source data 1/annotated/Fig.6B mef wt +mk2206 check k20me s21p anti-EZH2 uncropped.tif]

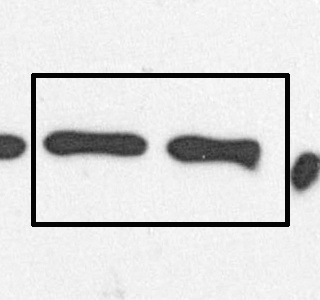

Supplement: Figure 6—source data 1. [file elife-86168-fig6-data1.zip › Figure 6 source data 1/annotated/Fig.6A t47d treated with mk2206 4 um anti-ezh2 uncropped.tif]

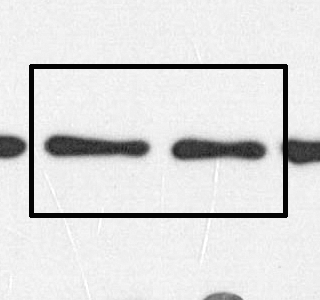

Supplement: Figure 6—source data 1. [file elife-86168-fig6-data1.zip › Figure 6 source data 1/annotated/Fig.6A t47d treated with mk2206 4 um anti-actin uncropped.tif]

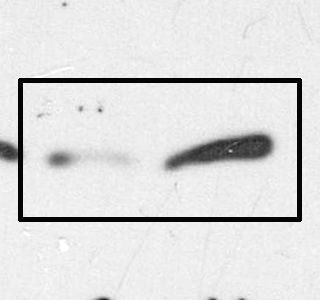

Supplement: Figure 6—source data 1. [file elife-86168-fig6-data1.zip › Figure 6 source data 1/annotated/Fig.6Emef treat with AKT inhibitor anti-H3K27me3 UNcropped.tif]

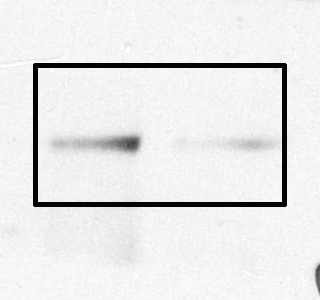

Supplement: Figure 6—source data 1. [file elife-86168-fig6-data1.zip › Figure 6 source data 1/annotated/Fig.6B mef wt +mk2206 check k20me s21p anti-EZH2-S21P uncropped.tif]

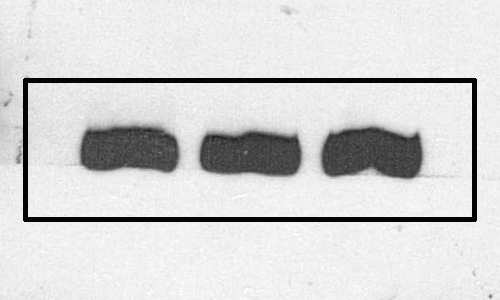

Supplement: Figure 6—source data 1. [file elife-86168-fig6-data1.zip › Figure 6 source data 1/annotated/Fig.6H mef wt k2or ip ezh2 with h3 input anti-EZH2 Uncropped.tif]

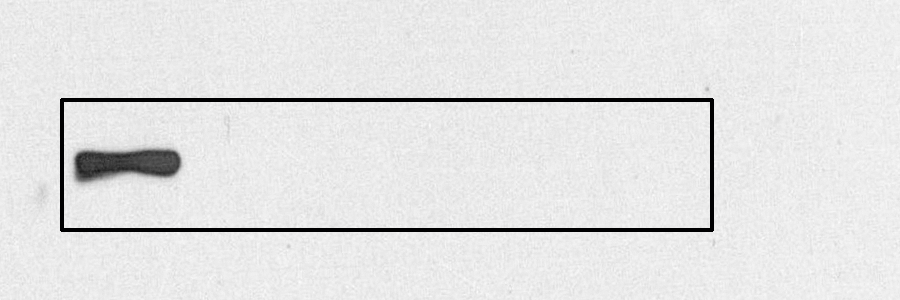

Supplement: Figure 6—source data 1. [file elife-86168-fig6-data1.zip › Figure 6 source data 1/annotated/Figure6C PA1 MK2206 0-6 UM anti-ps473AKT uncropped.tif]

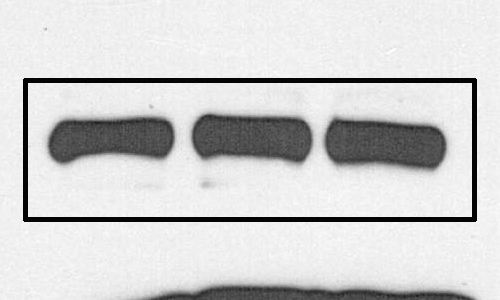

Supplement: Figure 6—source data 1. [file elife-86168-fig6-data1.zip › Figure 6 source data 1/annotated/Fig.6I mef-k20r ip ezh2 with h3 input anti-EZH2 uncropped.tif]

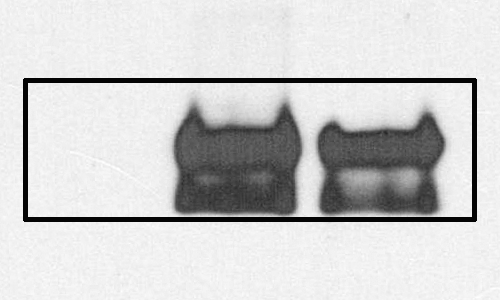

Supplement: Figure 6—source data 1. [file elife-86168-fig6-data1.zip › Figure 6 source data 1/annotated/Fig.6H MEF-K20R IP EZH2 With suz12 eed ip anti-ezh2 uncropped.tif]

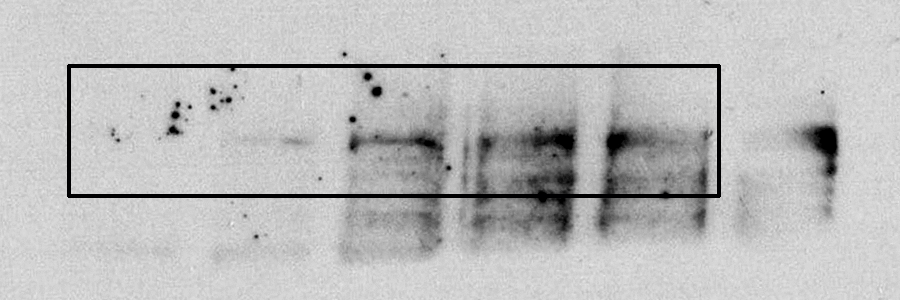

Supplement: Figure 6—source data 1. [file elife-86168-fig6-data1.zip › Figure 6 source data 1/annotated/Figure6C PA1 MK2206 0-6 UM anti-ezh2k20me uncropped.tif]

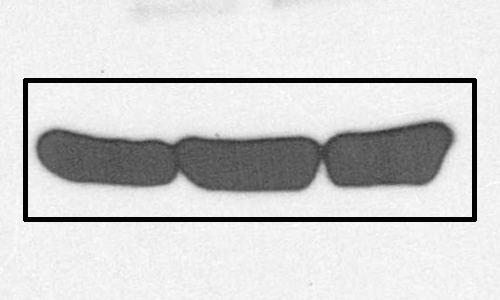

Supplement: Figure 6—source data 1. [file elife-86168-fig6-data1.zip › Figure 6 source data 1/annotated/Fig.6H MEF-K20R IP EZH2 With suz12 eed input anti-actin uncropped.tif]

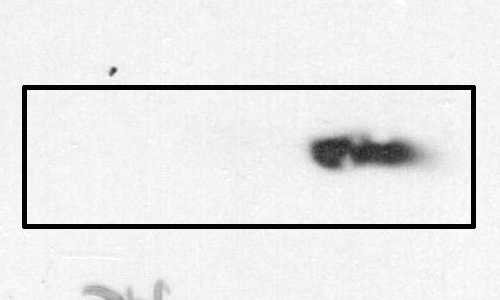

Supplement: Figure 6—source data 1. [file elife-86168-fig6-data1.zip › Figure 6 source data 1/annotated/Fig.6I mef-k20r ip ezh2 with h3 iP anti-H3 uncropped.tif]

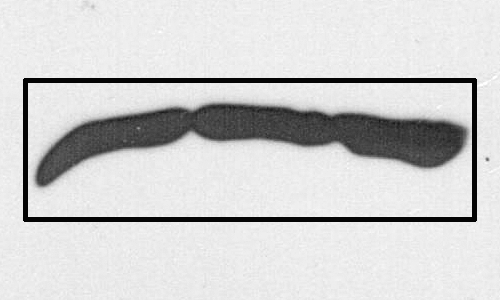

Supplement: Figure 6—source data 1. [file elife-86168-fig6-data1.zip › Figure 6 source data 1/annotated/Fig.6I mef-k20r ip ezh2 with h3 input anti-actin uncropped.tif]

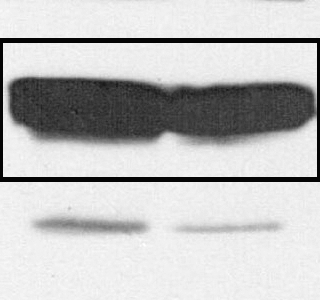

Supplement: Figure 6—source data 1. [file elife-86168-fig6-data1.zip › Figure 6 source data 1/annotated/Fig.6B mef wt +mk2206 check k20me s21p anti-H3 uncropped.tif]

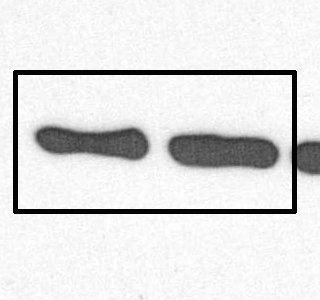

Supplement: Figure 6—source data 1. [file elife-86168-fig6-data1.zip › Figure 6 source data 1/annotated/Fig.6D t47d transfection with pkh3-l3 d5 treat with mk2206 for 4h anti-H3 uncropped.tif]

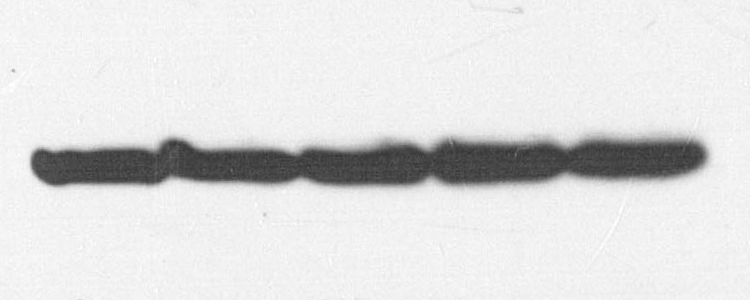

Supplement: Figure 6—figure supplement 1—source data 1. [file elife-86168-fig6-figsupp1-data1.zip › Figure 6-figure supplement1 source data 1/figure supplement 1 G401-k20r chx si luc anti--actin uncropped.tif]

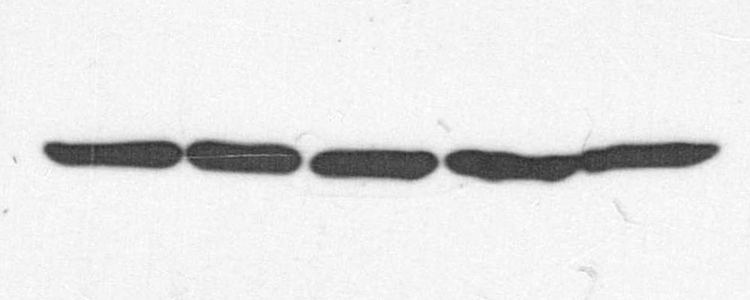

Supplement: Figure 6—figure supplement 1—source data 1. [file elife-86168-fig6-figsupp1-data1.zip › Figure 6-figure supplement1 source data 1/figure supplement 1 G401-ezh2 si lUC chx anti-ACTIN uncropped.tif]

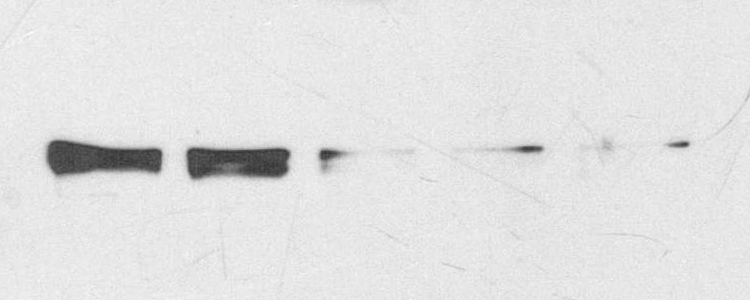

Supplement: Figure 6—figure supplement 1—source data 1. [file elife-86168-fig6-figsupp1-data1.zip › Figure 6-figure supplement1 source data 1/figure supplement 1 G401-ezh2 si lsd1 chx anti-ha-ezh2 uncropped.tif]

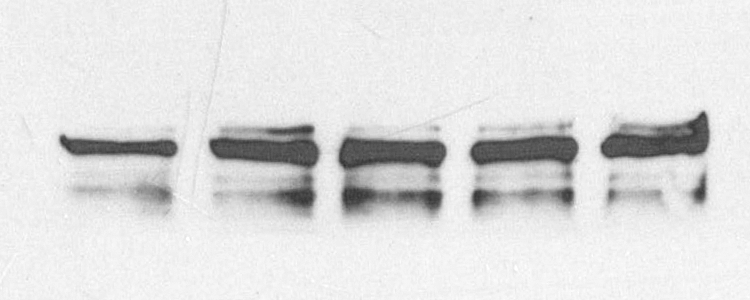

Supplement: Figure 6—figure supplement 1—source data 1. [file elife-86168-fig6-figsupp1-data1.zip › Figure 6-figure supplement1 source data 1/figure supplement 1 G401-k20r chx si luc lsd1 anti-HA-EZH2 uncropped.tif]

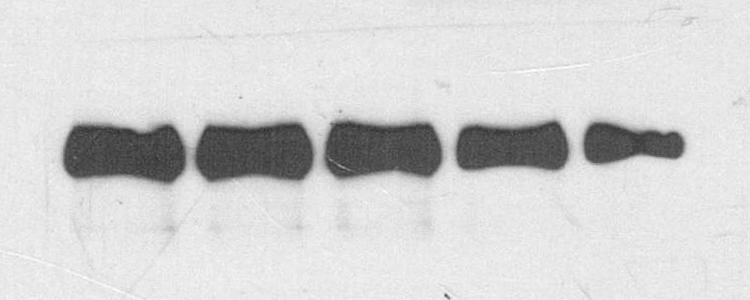

Supplement: Figure 6—figure supplement 1—source data 1. [file elife-86168-fig6-figsupp1-data1.zip › Figure 6-figure supplement1 source data 1/figure supplement 1 G401-ezh2 si lUC chx anti-ha-ezh2 uncropped.tif]

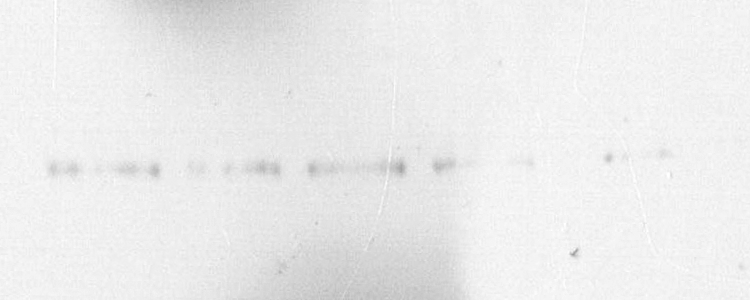

Supplement: Figure 6—figure supplement 1—source data 1. [file elife-86168-fig6-figsupp1-data1.zip › Figure 6-figure supplement1 source data 1/figure supplement 1 G401-S21A chx si lsd1 anti-lsd1 uncropped.tif]

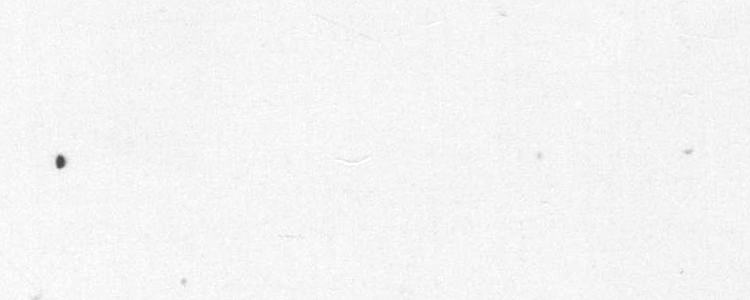

Supplement: Figure 6—figure supplement 1—source data 1. [file elife-86168-fig6-figsupp1-data1.zip › Figure 6-figure supplement1 source data 1/figure supplement 1 G401-ezh2 si lsd1 chx anti-LSD1 uncropped.tif]

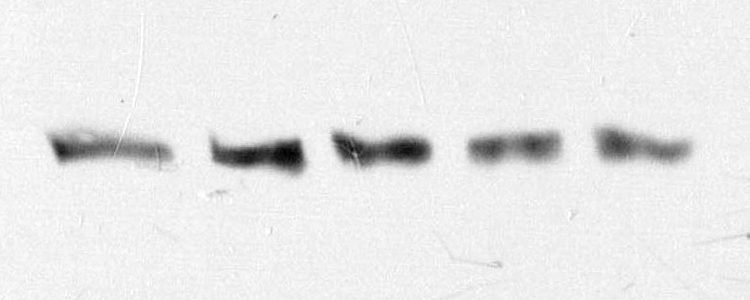

Supplement: Figure 6—figure supplement 1—source data 1. [file elife-86168-fig6-figsupp1-data1.zip › Figure 6-figure supplement1 source data 1/figure supplement 1 G401-k20r chx si luc anti-lsd1 1 uncropped.tif]

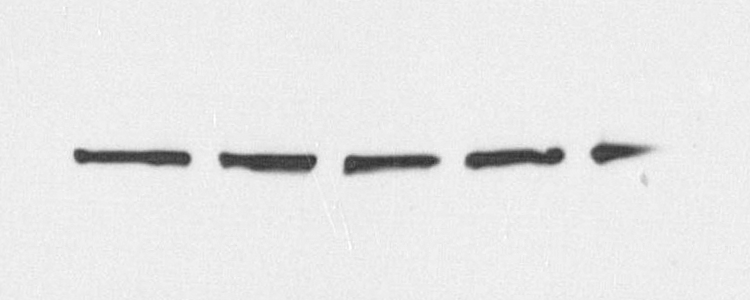

Supplement: Figure 6—figure supplement 1—source data 1. [file elife-86168-fig6-figsupp1-data1.zip › Figure 6-figure supplement1 source data 1/figure supplement 1 G401-S21A chx si luc anti-ha-ezh2 uncropped.tif]

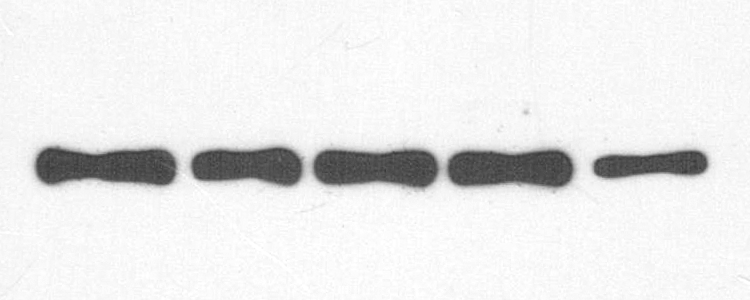

Supplement: Figure 6—figure supplement 1—source data 1. [file elife-86168-fig6-figsupp1-data1.zip › Figure 6-figure supplement1 source data 1/figure supplement 1 G401-ezh2 si luc chx anti-LSD1 uncropped.tif]

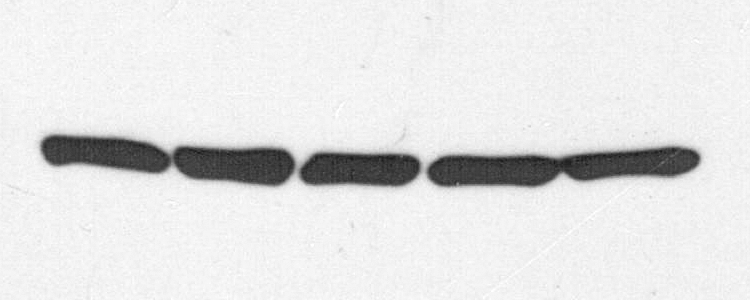

Supplement: Figure 6—figure supplement 1—source data 1. [file elife-86168-fig6-figsupp1-data1.zip › Figure 6-figure supplement1 source data 1/figure supplement 1 G401-ezh2 si lsd1 chx anti-ACTIN uncropped.tif]

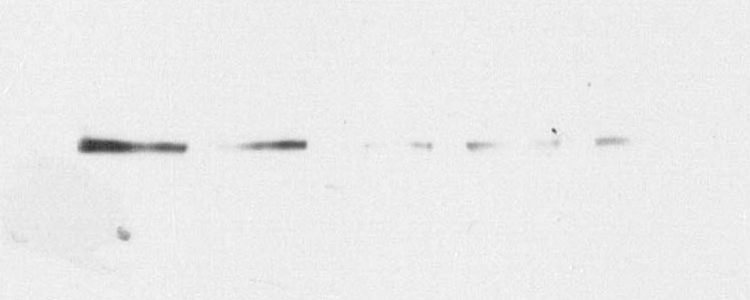

Supplement: Figure 6—figure supplement 1—source data 1. [file elife-86168-fig6-figsupp1-data1.zip › Figure 6-figure supplement1 source data 1/figure supplement 1 G401-S21A chx si lsd1 anti-ha-ezh2 uncropped.tif]

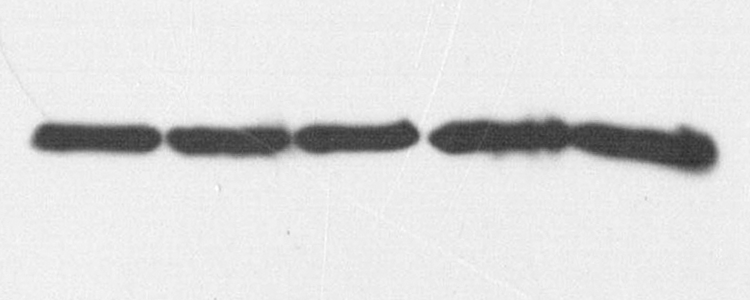

Supplement: Figure 6—figure supplement 1—source data 1. [file elife-86168-fig6-figsupp1-data1.zip › Figure 6-figure supplement1 source data 1/figure supplement 1 G401-S21A chx si luc anti-HA-actin uncropped.tif]
