# Supplementary material for: A methylation-phosphorylation switch controls EZH2 stability and hematopoiesis
Source: eLife. 2024 Feb 12;13:e86168. doi: 10.7554/eLife.86168 (PMC10901513; doi:10.7554/eLife.86168)

si-Luc

Figure 6-figure supplement 1-HA-EZH2

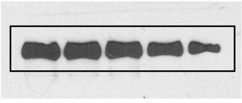

Figure 6-figure supplement 1-LSD1

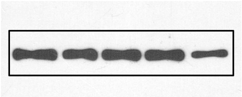

Figure 6-figure supplement 1-Actin

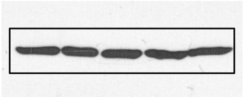

si-LSD1

Figure 6-figure supplement 1-HA-EZH2

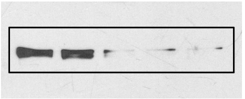

Figure 6-figure supplement 1-LSD1

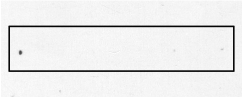

Figure 6-figure supplement 1-Actin

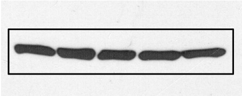

si-Luc

Figure 6-figure supplement 1-HA-EZH2-K20R

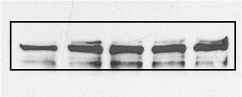

Figure 6-figure supplement 1-LSD1

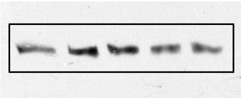

Figure 6-figure supplement 1-Actin

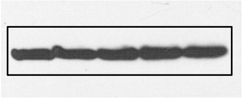

si-LSD1

Figure 6-figure supplement 1-HA-EZH2-K20R

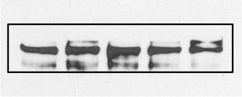

Figure 6-figure supplement 1-LSD1

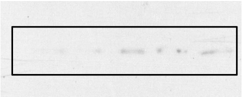

Figure 6-figure supplement 1-Actin

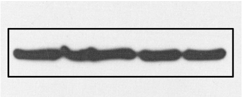

si-Luc

Figure 6-figure supplement 1-HA-EZH2-K20R

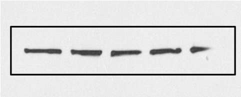

Figure 6-figure supplement 1-LSD1

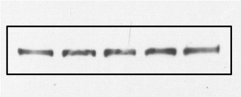

Figure 6-figure supplement 1-Actin

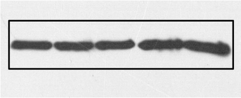

si-LSD1

Figure 6-figure supplement 1-HA-EZH2-K20R

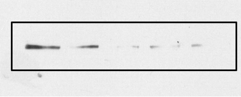

Figure 6-figure supplement 1-LSD1

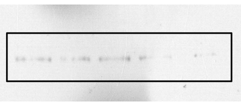

Figure 6-figure supplement 1-Actin

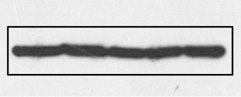

Supplement: Figure 6—figure supplement 1—source data 1. [file elife-86168-fig6-figsupp1-data1.zip › Figure 6-figure supplement1 source data 1/Figure 6-figure supplement 1.pdf]
