# Supplementary figures and images for: A methylation-phosphorylation switch controls EZH2 stability and hematopoiesis (part 6 of 7)
Source: eLife. 2024 Feb 12;13:e86168. doi: 10.7554/eLife.86168 (PMC10901513; doi:10.7554/eLife.86168)

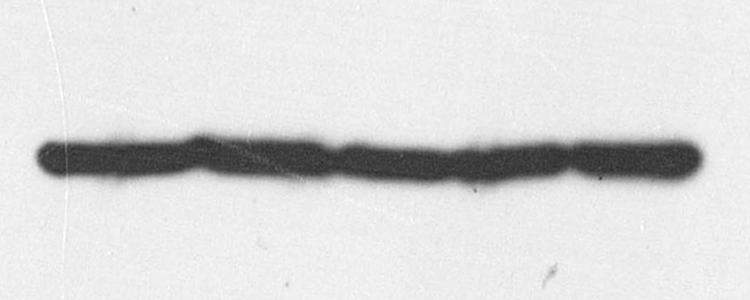

Supplement: Figure 6—figure supplement 1—source data 1. [file elife-86168-fig6-figsupp1-data1.zip › Figure 6-figure supplement1 source data 1/figure supplement 1 G401-S21A chx si lsd1 anti-actin uncropped.tif]

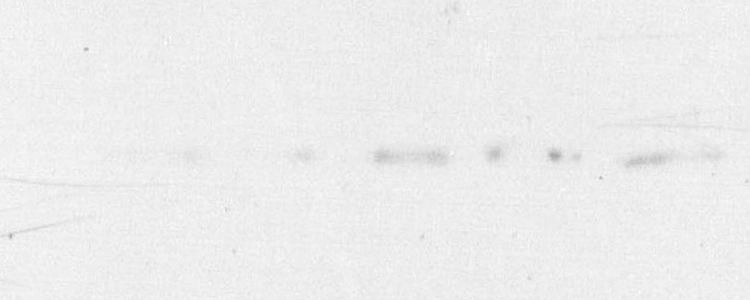

Supplement: Figure 6—figure supplement 1—source data 1. [file elife-86168-fig6-figsupp1-data1.zip › Figure 6-figure supplement1 source data 1/figure supplement 1 G401-k20r chx si lsd1 anti-lsd1 uncropped.tif]

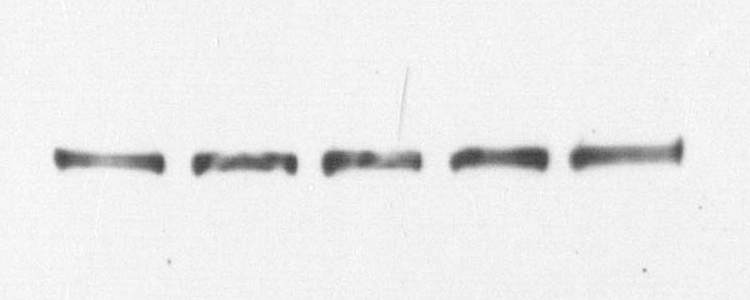

Supplement: Figure 6—figure supplement 1—source data 1. [file elife-86168-fig6-figsupp1-data1.zip › Figure 6-figure supplement1 source data 1/figure supplement 1 G401-S21A chx si luc anti-lsd1 uncropped.tif]

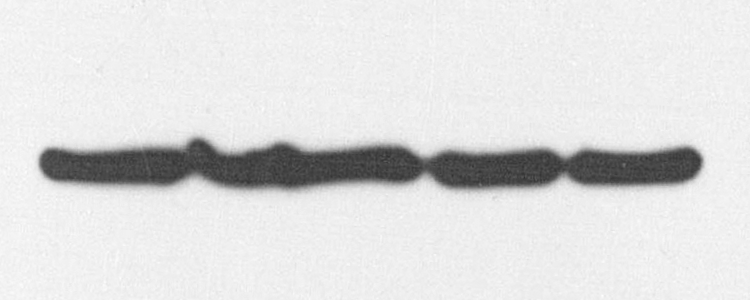

Supplement: Figure 6—figure supplement 1—source data 1. [file elife-86168-fig6-figsupp1-data1.zip › Figure 6-figure supplement1 source data 1/figure supplement 1 G401-k20r chx si lsd1 anti-actin uncropped.tif]

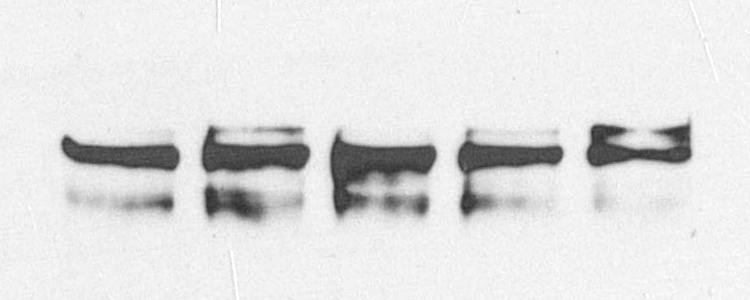

Supplement: Figure 6—figure supplement 1—source data 1. [file elife-86168-fig6-figsupp1-data1.zip › Figure 6-figure supplement1 source data 1/figure supplement 1 G401-k20r chx si lsd1 anti-HA-EZH2 uncropped.tif]

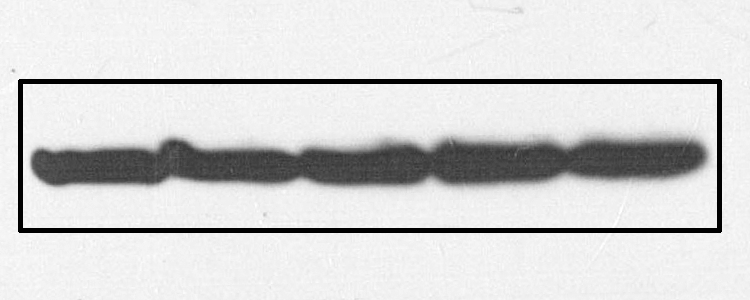

Supplement: Figure 6—figure supplement 1—source data 1. [file elife-86168-fig6-figsupp1-data1.zip › Figure 6-figure supplement1 source data 1/annotated/figure supplement 1 G401-k20r chx si luc anti--actin uncropped.tif]

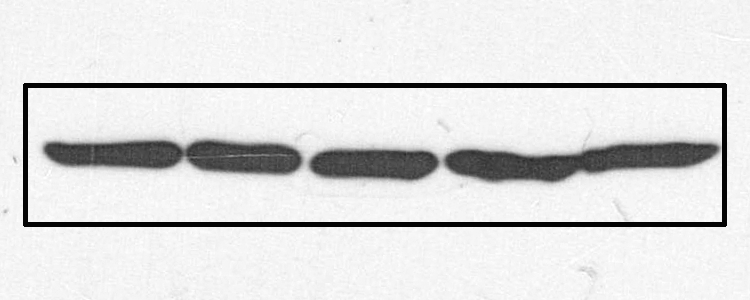

Supplement: Figure 6—figure supplement 1—source data 1. [file elife-86168-fig6-figsupp1-data1.zip › Figure 6-figure supplement1 source data 1/annotated/figure supplement 1 G401-ezh2 si lUC chx anti-ACTIN uncropped.tif]

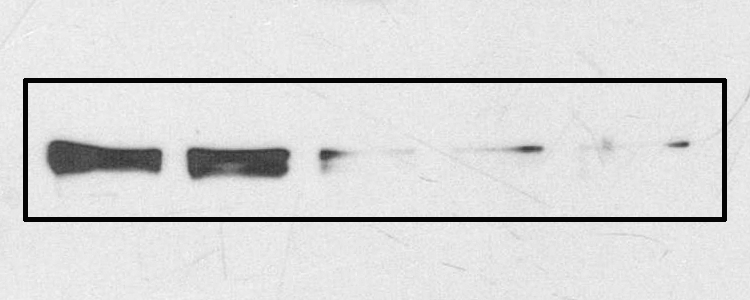

Supplement: Figure 6—figure supplement 1—source data 1. [file elife-86168-fig6-figsupp1-data1.zip › Figure 6-figure supplement1 source data 1/annotated/figure supplement 1 G401-ezh2 si lsd1 chx anti-ha-ezh2 uncropped.tif]

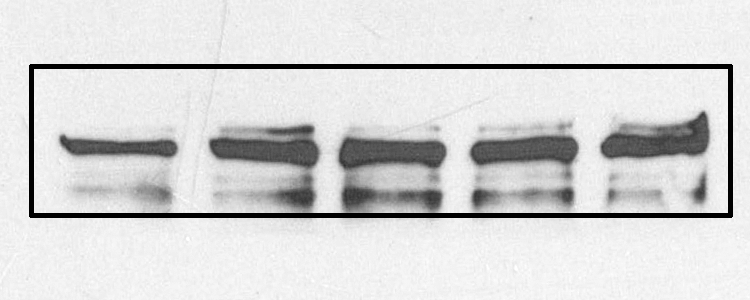

Supplement: Figure 6—figure supplement 1—source data 1. [file elife-86168-fig6-figsupp1-data1.zip › Figure 6-figure supplement1 source data 1/annotated/figure supplement 1 G401-k20r chx si luc lsd1 anti-HA-EZH2 uncropped.tif]

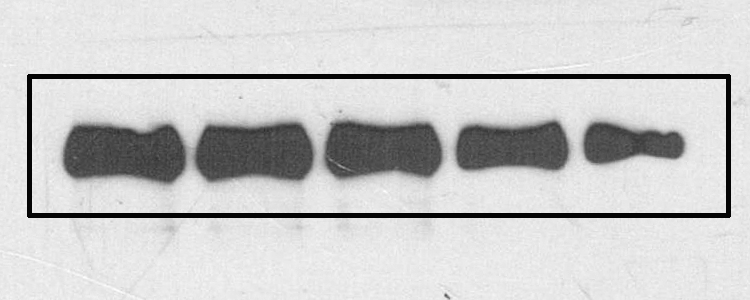

Supplement: Figure 6—figure supplement 1—source data 1. [file elife-86168-fig6-figsupp1-data1.zip › Figure 6-figure supplement1 source data 1/annotated/figure supplement 1 G401-ezh2 si lUC chx anti-ha-ezh2 uncropped.tif]

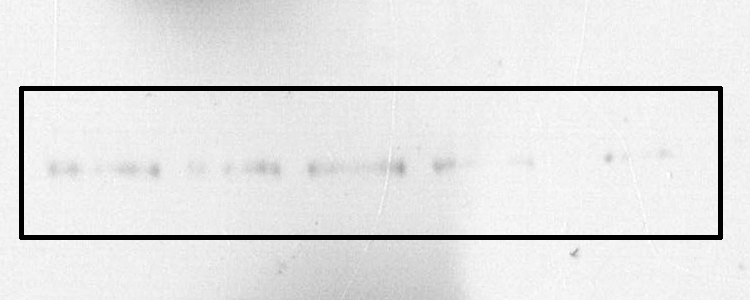

Supplement: Figure 6—figure supplement 1—source data 1. [file elife-86168-fig6-figsupp1-data1.zip › Figure 6-figure supplement1 source data 1/annotated/figure supplement 1 G401-S21A chx si lsd1 anti-lsd1 uncropped.tif]

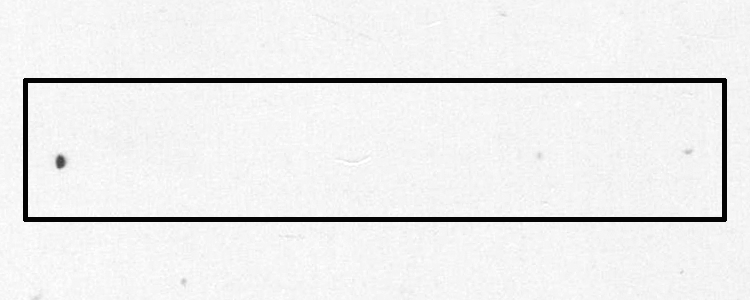

Supplement: Figure 6—figure supplement 1—source data 1. [file elife-86168-fig6-figsupp1-data1.zip › Figure 6-figure supplement1 source data 1/annotated/figure supplement 1 G401-ezh2 si lsd1 chx anti-LSD1 uncropped.tif]

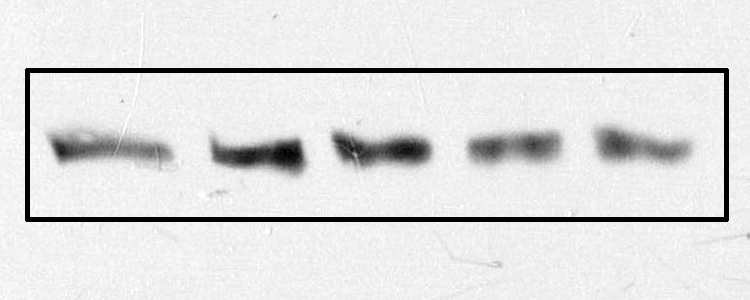

Supplement: Figure 6—figure supplement 1—source data 1. [file elife-86168-fig6-figsupp1-data1.zip › Figure 6-figure supplement1 source data 1/annotated/figure supplement 1 G401-k20r chx si luc anti-lsd1 1 uncropped.tif]

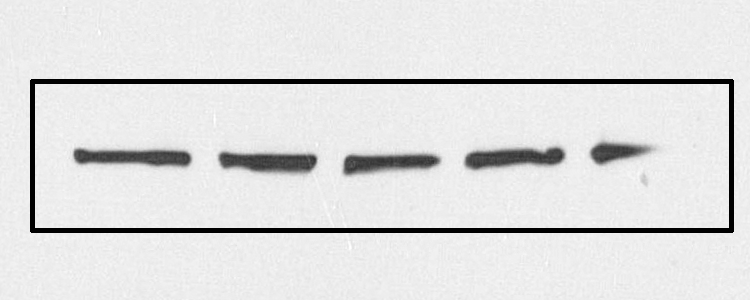

Supplement: Figure 6—figure supplement 1—source data 1. [file elife-86168-fig6-figsupp1-data1.zip › Figure 6-figure supplement1 source data 1/annotated/figure supplement 1 G401-S21A chx si luc anti-ha-ezh2 uncropped.tif]

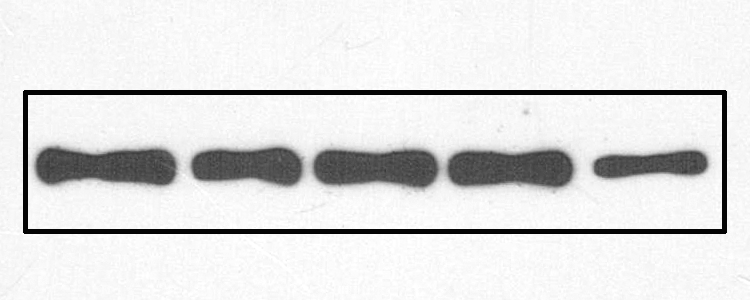

Supplement: Figure 6—figure supplement 1—source data 1. [file elife-86168-fig6-figsupp1-data1.zip › Figure 6-figure supplement1 source data 1/annotated/figure supplement 1 G401-ezh2 si luc chx anti-LSD1 uncropped.tif]

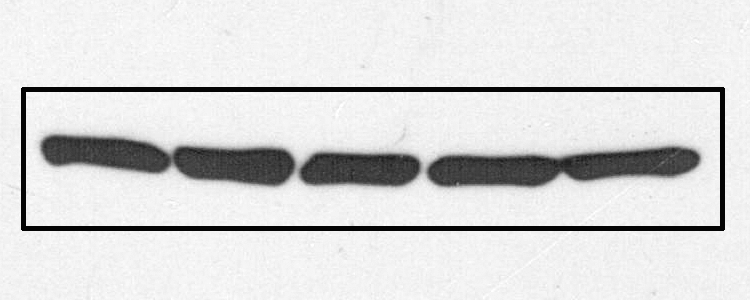

Supplement: Figure 6—figure supplement 1—source data 1. [file elife-86168-fig6-figsupp1-data1.zip › Figure 6-figure supplement1 source data 1/annotated/figure supplement 1 G401-ezh2 si lsd1 chx anti-ACTIN uncropped.tif]

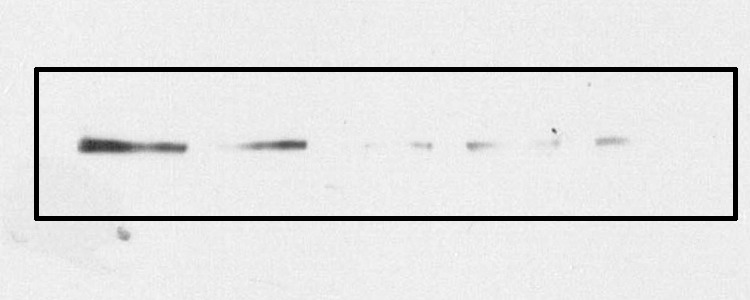

Supplement: Figure 6—figure supplement 1—source data 1. [file elife-86168-fig6-figsupp1-data1.zip › Figure 6-figure supplement1 source data 1/annotated/figure supplement 1 G401-S21A chx si lsd1 anti-ha-ezh2 uncropped.tif]

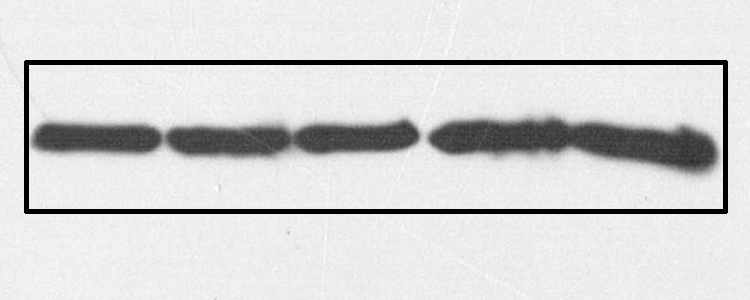

Supplement: Figure 6—figure supplement 1—source data 1. [file elife-86168-fig6-figsupp1-data1.zip › Figure 6-figure supplement1 source data 1/annotated/figure supplement 1 G401-S21A chx si luc anti-HA-actin uncropped.tif]

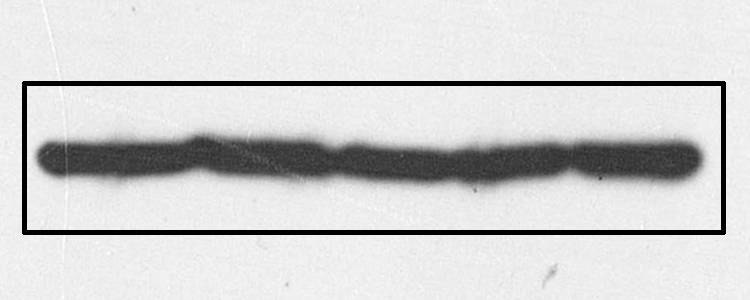

Supplement: Figure 6—figure supplement 1—source data 1. [file elife-86168-fig6-figsupp1-data1.zip › Figure 6-figure supplement1 source data 1/annotated/figure supplement 1 G401-S21A chx si lsd1 anti-actin uncropped.tif]

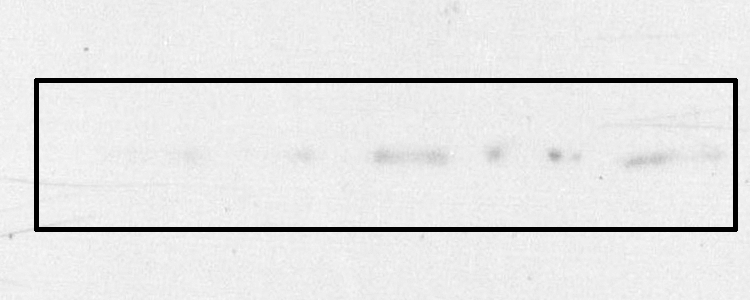

Supplement: Figure 6—figure supplement 1—source data 1. [file elife-86168-fig6-figsupp1-data1.zip › Figure 6-figure supplement1 source data 1/annotated/figure supplement 1 G401-k20r chx si lsd1 anti-lsd1 uncropped.tif]

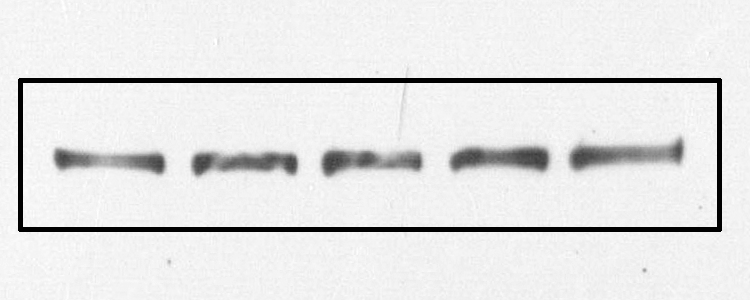

Supplement: Figure 6—figure supplement 1—source data 1. [file elife-86168-fig6-figsupp1-data1.zip › Figure 6-figure supplement1 source data 1/annotated/figure supplement 1 G401-S21A chx si luc anti-lsd1 uncropped.tif]

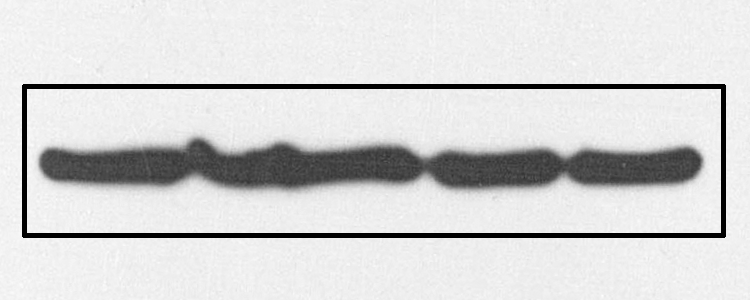

Supplement: Figure 6—figure supplement 1—source data 1. [file elife-86168-fig6-figsupp1-data1.zip › Figure 6-figure supplement1 source data 1/annotated/figure supplement 1 G401-k20r chx si lsd1 anti-actin uncropped.tif]

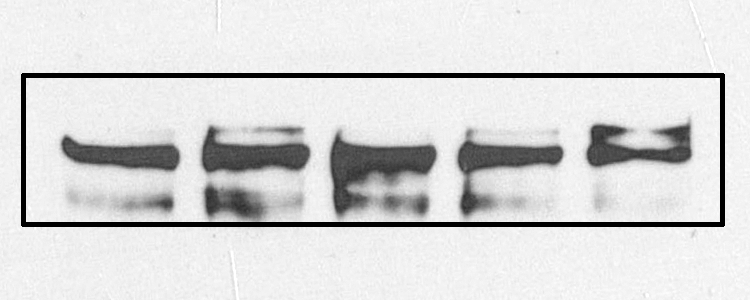

Supplement: Figure 6—figure supplement 1—source data 1. [file elife-86168-fig6-figsupp1-data1.zip › Figure 6-figure supplement1 source data 1/annotated/figure supplement 1 G401-k20r chx si lsd1 anti-HA-EZH2 uncropped.tif]

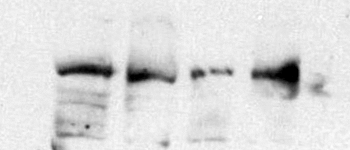

Supplement: Figure 6—figure supplement 2—source data 1. [file elife-86168-fig6-figsupp2-data1.zip › Figure 6-figure supplement2 source data 1/figure supplement 2C H1299 treat with mk2206 for 4h check ezh2 anti-EZH2K20me uncropped.tif]

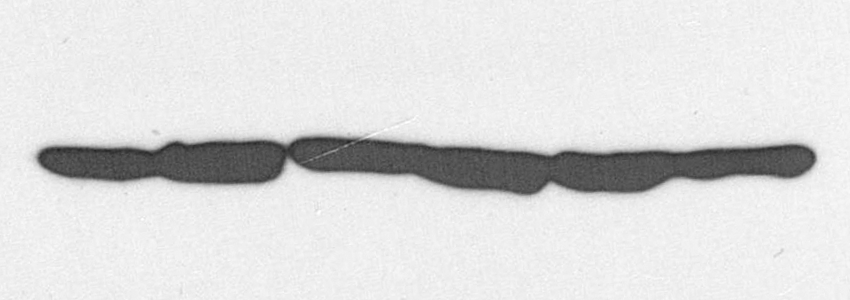

Supplement: Figure 6—figure supplement 2—source data 1. [file elife-86168-fig6-figsupp2-data1.zip › Figure 6-figure supplement2 source data 1/figure supplement 2A 20220627 cell detecting l3 lsd1 anti-actin uncropped.tif]

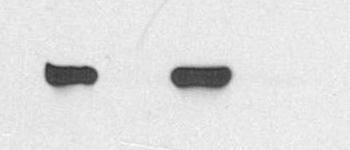

Supplement: Figure 6—figure supplement 2—source data 1. [file elife-86168-fig6-figsupp2-data1.zip › Figure 6-figure supplement2 source data 1/figure supplement 2C H1299 treat with mk2206 for 4h check ezh2 anti-pS473AKT uncropped.tif]

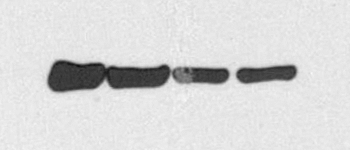

Supplement: Figure 6—figure supplement 2—source data 1. [file elife-86168-fig6-figsupp2-data1.zip › Figure 6-figure supplement2 source data 1/figure supplement 2C H1299 treat with mk2206 for 4h check ezh2 anti-AKT uncropped.tif]

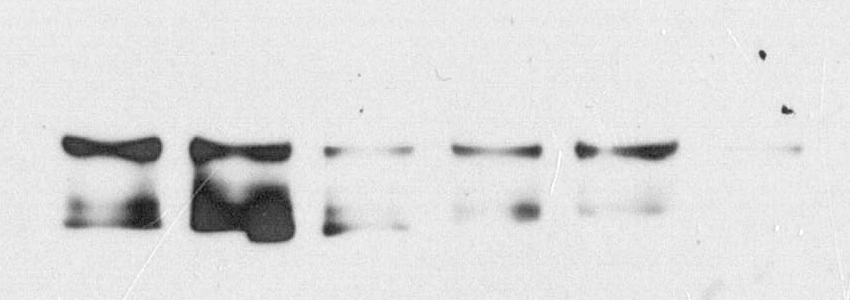

Supplement: Figure 6—figure supplement 2—source data 1. [file elife-86168-fig6-figsupp2-data1.zip › Figure 6-figure supplement2 source data 1/figure supplement 2A 20230313 293t hct116 h1299 pa1 t47d h520 check ezh2 anti-EZH2 Low exposure Uncropped.tif]

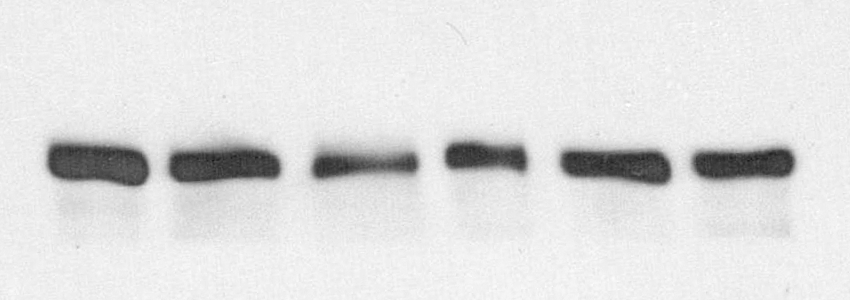

Supplement: Figure 6—figure supplement 2—source data 1. [file elife-86168-fig6-figsupp2-data1.zip › Figure 6-figure supplement2 source data 1/figure supplement 2A 20220627 cell detecting l3 lsd1 anti-lsd1 uncropped.tif]

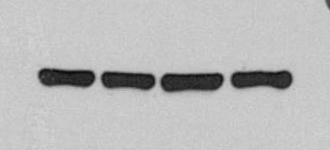

Supplement: Figure 6—figure supplement 2—source data 1. [file elife-86168-fig6-figsupp2-data1.zip › Figure 6-figure supplement2 source data 1/figure supplement 2B T47D treat with mk2206 for 4h anti-ATK Uncropped.tif]

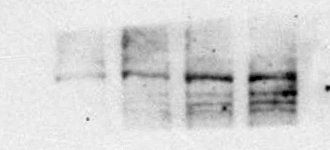

Supplement: Figure 6—figure supplement 2—source data 1. [file elife-86168-fig6-figsupp2-data1.zip › Figure 6-figure supplement2 source data 1/figure supplement 2B T47D treat with mk2206 for 4h anti-EZH2-K20me Uncropped.tif]

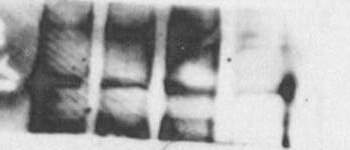

Supplement: Figure 6—figure supplement 2—source data 1. [file elife-86168-fig6-figsupp2-data1.zip › Figure 6-figure supplement2 source data 1/figure supplement 2C H1299 treat with mk2206 for 4h check ezh2 anti-EZH2S21p uncropped.tif]

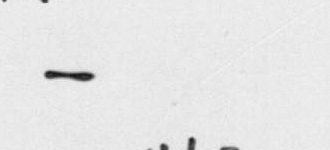

Supplement: Figure 6—figure supplement 2—source data 1. [file elife-86168-fig6-figsupp2-data1.zip › Figure 6-figure supplement2 source data 1/figure supplement 2B T47D treat with mk2206 for 4h anti-pS473ATK Uncropped.tif]

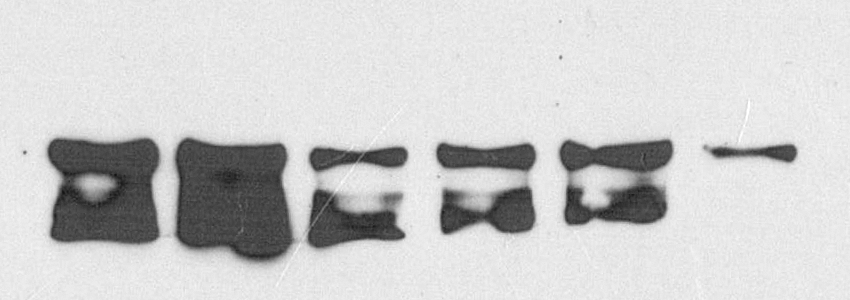

Supplement: Figure 6—figure supplement 2—source data 1. [file elife-86168-fig6-figsupp2-data1.zip › Figure 6-figure supplement2 source data 1/figure supplement 2A 20230313 293t hct116 h1299 pa1 t47d h520 check ezh2 anti-EZH2 Uncropped.tif]

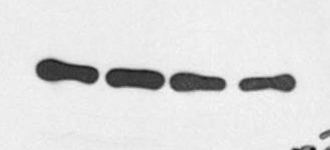

Supplement: Figure 6—figure supplement 2—source data 1. [file elife-86168-fig6-figsupp2-data1.zip › Figure 6-figure supplement2 source data 1/figure supplement 2B T47D treat with mk2206 for 4h anti-EZH2 Uncropped.tif]

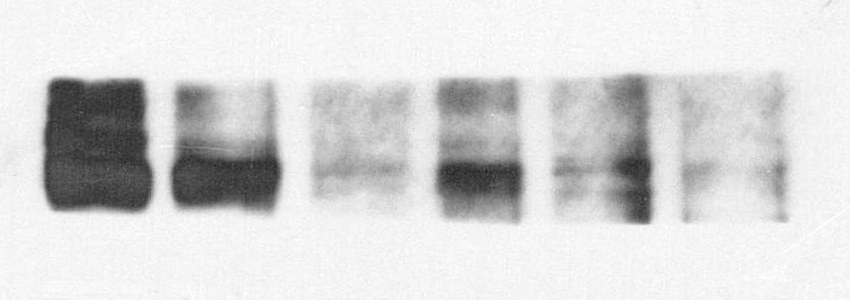

Supplement: Figure 6—figure supplement 2—source data 1. [file elife-86168-fig6-figsupp2-data1.zip › Figure 6-figure supplement2 source data 1/figure supplement 2A 20220627 cell detecting l3 lsd1 anti-l3 uncropped.tif]

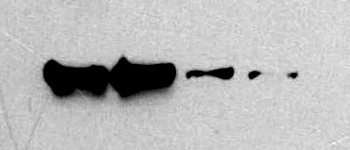

Supplement: Figure 6—figure supplement 2—source data 1. [file elife-86168-fig6-figsupp2-data1.zip › Figure 6-figure supplement2 source data 1/figure supplement 2C H1299 treat with mk2206 for 4h check ezh2 anti-EZH2 uncropped.tif]

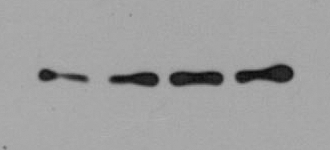

Supplement: Figure 6—figure supplement 2—source data 1. [file elife-86168-fig6-figsupp2-data1.zip › Figure 6-figure supplement2 source data 1/figure supplement 2B T47D treat with mk2206 for 4h anti-H3K27me3 Uncropped.tif]

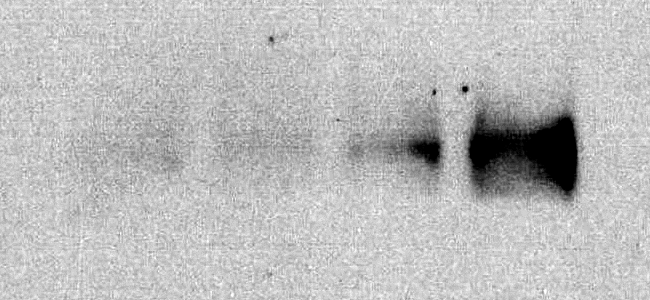

Supplement: Figure 6—figure supplement 2—source data 1. [file elife-86168-fig6-figsupp2-data1.zip › Figure 6-figure supplement2 source data 1/figure supplement 2C H1299 treat with mk2206 for 4h check ezh2 anti-EZH2K20me uncropped.tif]

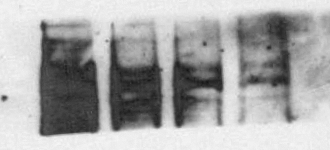

Supplement: Figure 6—figure supplement 2—source data 1. [file elife-86168-fig6-figsupp2-data1.zip › Figure 6-figure supplement2 source data 1/figure supplement 2B T47D treat with mk2206 for 4h anti-EZH2-S21p Uncropped.tif]

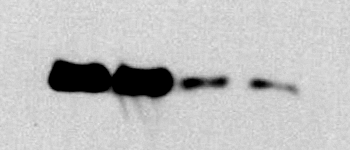

Supplement: Figure 6—figure supplement 2—source data 1. [file elife-86168-fig6-figsupp2-data1.zip › Figure 6-figure supplement2 source data 1/figure supplement 2C H1299 treat with mk2206 for 4h check ezh2 anti-H3K27me3 uncropped.tif]

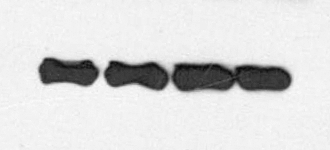

Supplement: Figure 6—figure supplement 2—source data 1. [file elife-86168-fig6-figsupp2-data1.zip › Figure 6-figure supplement2 source data 1/figure supplement 2B T47D treat with mk2206 for 4h anti-H3 Uncropped.tif]

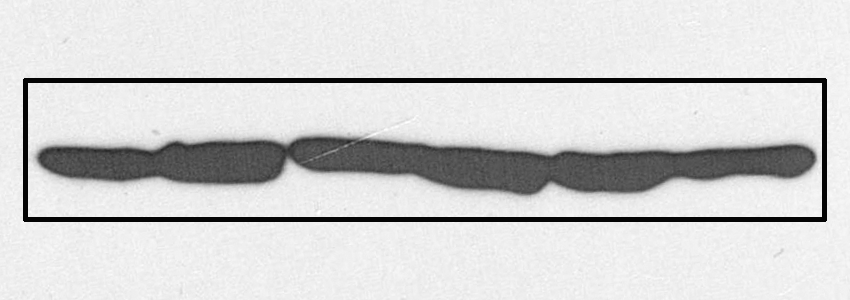

Supplement: Figure 6—figure supplement 2—source data 1. [file elife-86168-fig6-figsupp2-data1.zip › Figure 6-figure supplement2 source data 1/annotated/figure supplement 2A 20220627 cell detecting l3 lsd1 anti-actin uncropped.tif]

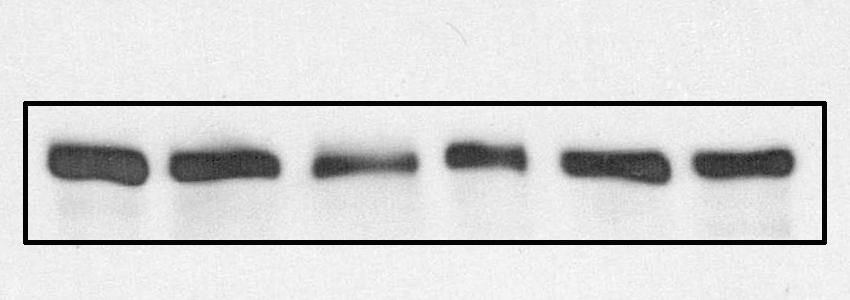

Supplement: Figure 6—figure supplement 2—source data 1. [file elife-86168-fig6-figsupp2-data1.zip › Figure 6-figure supplement2 source data 1/annotated/figure supplement 2A 20220627 cell detecting l3 lsd1 anti-lsd1 uncropped.tif]

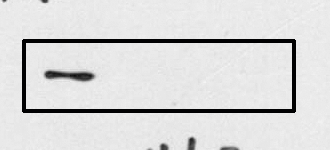

Supplement: Figure 6—figure supplement 2—source data 1. [file elife-86168-fig6-figsupp2-data1.zip › Figure 6-figure supplement2 source data 1/annotated/figure supplement 2B T47D treat with mk2206 for 4h anti-pS473ATK Uncropped.tif]

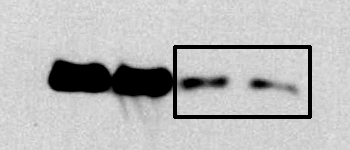

Supplement: Figure 6—figure supplement 2—source data 1. [file elife-86168-fig6-figsupp2-data1.zip › Figure 6-figure supplement2 source data 1/annotated/figure supplement 2C H1299 treat with mk2206 for 4h check ezh2 anti-H3K27me3 uncropped.tif]

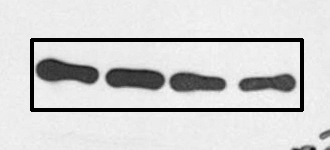

Supplement: Figure 6—figure supplement 2—source data 1. [file elife-86168-fig6-figsupp2-data1.zip › Figure 6-figure supplement2 source data 1/annotated/figure supplement 2B T47D treat with mk2206 for 4h anti-EZH2 Uncropped.tif]

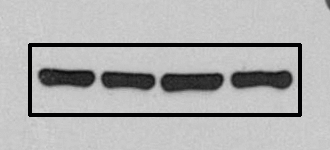

Supplement: Figure 6—figure supplement 2—source data 1. [file elife-86168-fig6-figsupp2-data1.zip › Figure 6-figure supplement2 source data 1/annotated/figure supplement 2B T47D treat with mk2206 for 4h anti-ATK uncropped.tif]

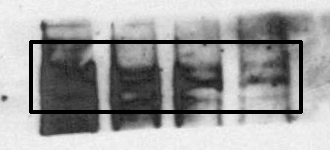

Supplement: Figure 6—figure supplement 2—source data 1. [file elife-86168-fig6-figsupp2-data1.zip › Figure 6-figure supplement2 source data 1/annotated/figure supplement 2B T47D treat with mk2206 for 4h anti-EZH2-S21p Uncropped.tif]

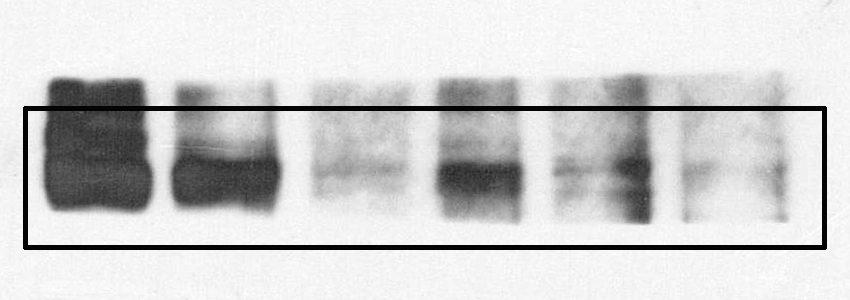

Supplement: Figure 6—figure supplement 2—source data 1. [file elife-86168-fig6-figsupp2-data1.zip › Figure 6-figure supplement2 source data 1/annotated/figure supplement 2A 20220627 cell detecting l3 lsd1 anti-l3 uncropped.tif]

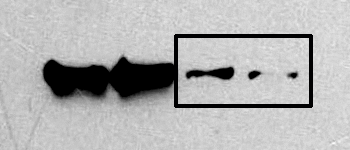

Supplement: Figure 6—figure supplement 2—source data 1. [file elife-86168-fig6-figsupp2-data1.zip › Figure 6-figure supplement2 source data 1/annotated/figure supplement 2C H1299 treat with mk2206 for 4h check ezh2 anti-EZH2 uncropped.tif]

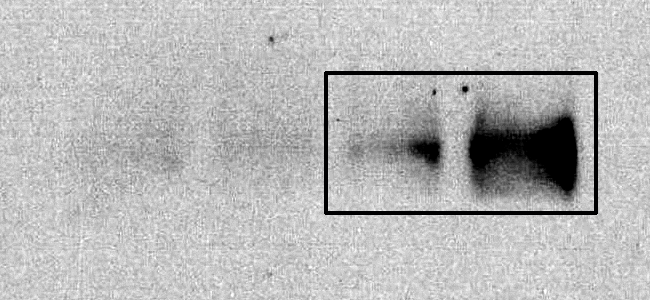

Supplement: Figure 6—figure supplement 2—source data 1. [file elife-86168-fig6-figsupp2-data1.zip › Figure 6-figure supplement2 source data 1/annotated/figure supplement 2C H1299 treat with mk2206 for 4h check ezh2 anti-EZH2K20me 1 UNcropped.tif]

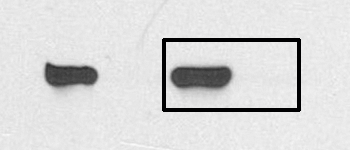

Supplement: Figure 6—figure supplement 2—source data 1. [file elife-86168-fig6-figsupp2-data1.zip › Figure 6-figure supplement2 source data 1/annotated/figure supplement 2C H1299 treat with mk2206 for 4h check ezh2 anti-pS473AKT uncropped.tif]

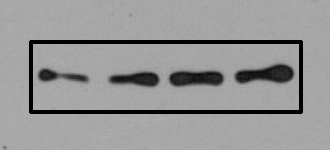

Supplement: Figure 6—figure supplement 2—source data 1. [file elife-86168-fig6-figsupp2-data1.zip › Figure 6-figure supplement2 source data 1/annotated/figure supplement 2B T47D treat with mk2206 for 4h anti-H3K27me3 Uncropped.tif]

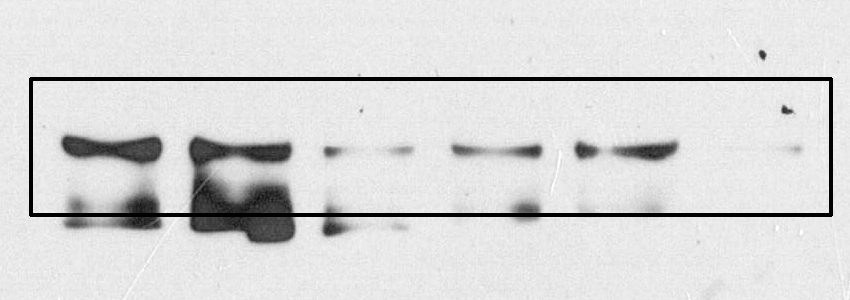

Supplement: Figure 6—figure supplement 2—source data 1. [file elife-86168-fig6-figsupp2-data1.zip › Figure 6-figure supplement2 source data 1/annotated/figure supplement 2A 20230313 293t hct116 h1299 pa1 t47d h520 check ezh2 anti-EZH2 Low exposure Uncropped.tif]

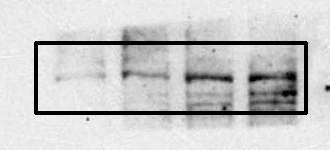

Supplement: Figure 6—figure supplement 2—source data 1. [file elife-86168-fig6-figsupp2-data1.zip › Figure 6-figure supplement2 source data 1/annotated/figure supplement 2B T47D treat with mk2206 for 4h anti-EZH2-K20me Uncropped.tif]

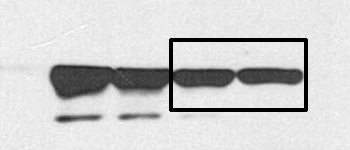

Supplement: Figure 6—figure supplement 2—source data 1. [file elife-86168-fig6-figsupp2-data1.zip › Figure 6-figure supplement2 source data 1/annotated/figure supplement 2C H1299 treat with mk2206 for 4h check ezh2 anti-H3 uncropped.tif]

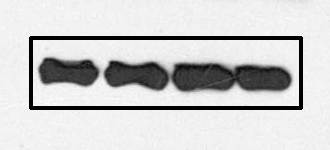

Supplement: Figure 6—figure supplement 2—source data 1. [file elife-86168-fig6-figsupp2-data1.zip › Figure 6-figure supplement2 source data 1/annotated/figure supplement 2B T47D treat with mk2206 for 4h anti-H3 uncropped.tif]

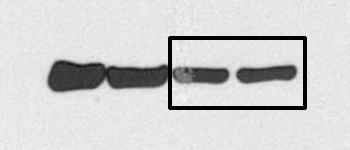

Supplement: Figure 6—figure supplement 2—source data 1. [file elife-86168-fig6-figsupp2-data1.zip › Figure 6-figure supplement2 source data 1/annotated/figure supplement 2C H1299 treat with mk2206 for 4h check ezh2 anti-AKT uncropped.tif]

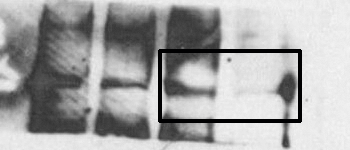

Supplement: Figure 6—figure supplement 2—source data 1. [file elife-86168-fig6-figsupp2-data1.zip › Figure 6-figure supplement2 source data 1/annotated/figure supplement 2C H1299 treat with mk2206 for 4h check ezh2 anti-EZH2S21p uncropped.tif]

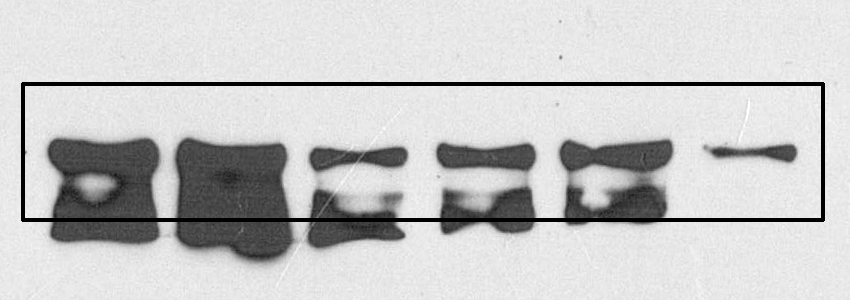

Supplement: Figure 6—figure supplement 2—source data 1. [file elife-86168-fig6-figsupp2-data1.zip › Figure 6-figure supplement2 source data 1/annotated/figure supplement 2A 20230313 293t hct116 h1299 pa1 t47d h520 check ezh2 anti-EZH2 UNcropped.tif]

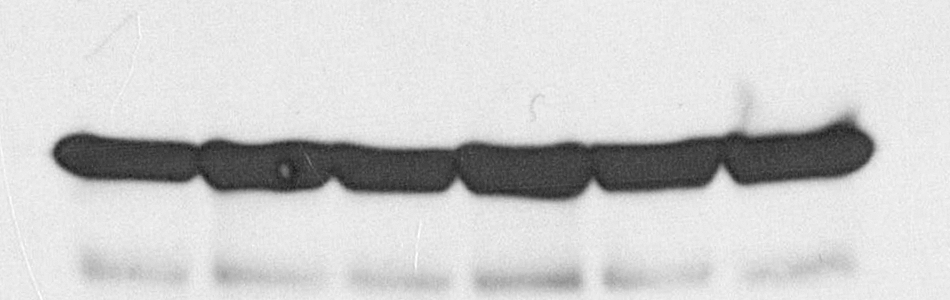

Supplement: Figure 6—figure supplement 4—source data 1. [file elife-86168-fig6-figsupp4-data1.zip › Figure 6-figure supplement4 source data/figure supplement4D MEFs treated with ta day9 anti-H3 Uncropped.tif]

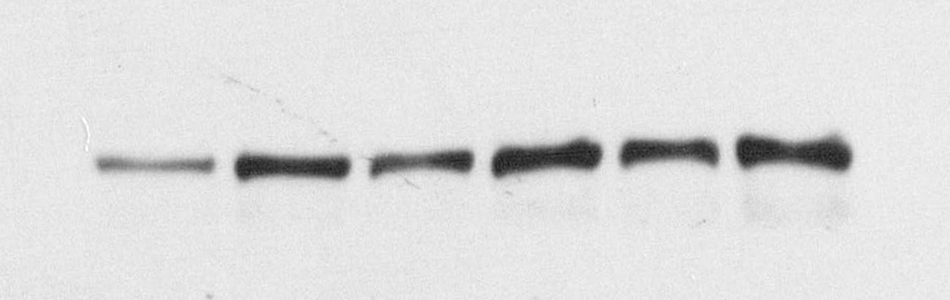

Supplement: Figure 6—figure supplement 4—source data 1. [file elife-86168-fig6-figsupp4-data1.zip › Figure 6-figure supplement4 source data/figure supplement4D MEFs treated with ta day9 anti-EZH2 Uncropped.tif]

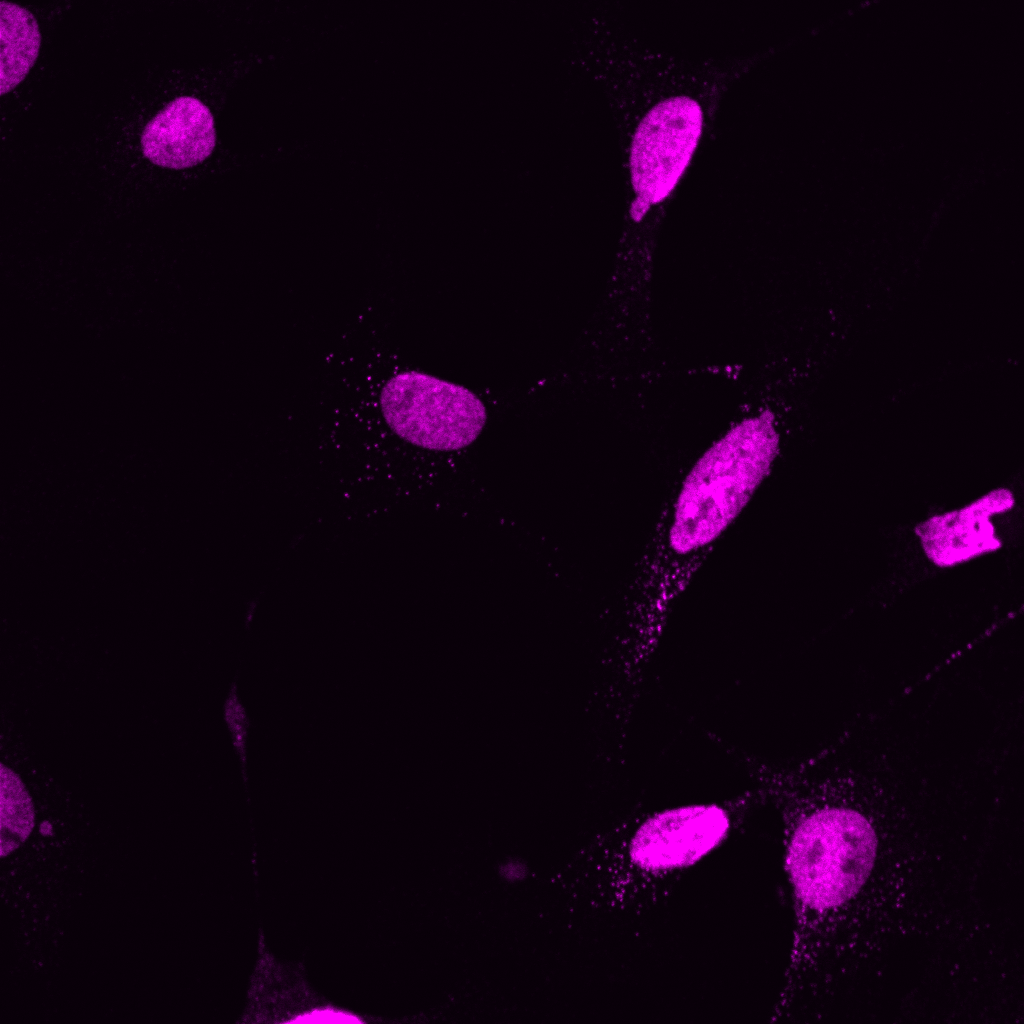

Supplement: Figure 6—figure supplement 4—source data 1. [file elife-86168-fig6-figsupp4-data1.zip › Figure 6-figure supplement4 source data/figure supplement4A mef ezh2 anti-ezh2 k20r 3c2.tif]

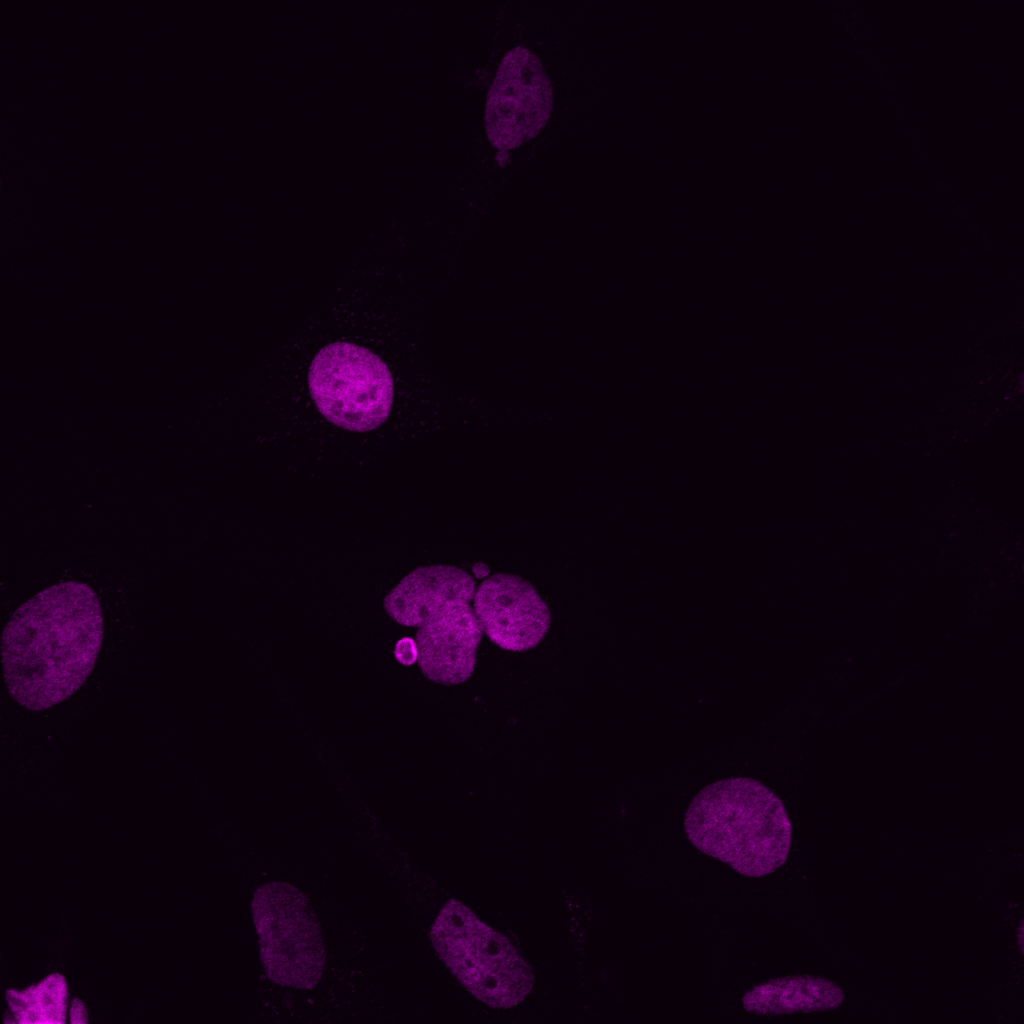

Supplement: Figure 6—figure supplement 4—source data 1. [file elife-86168-fig6-figsupp4-data1.zip › Figure 6-figure supplement4 source data/figure supplement4A mef ezh2 anti-ezh2 wt 1c2.tif]

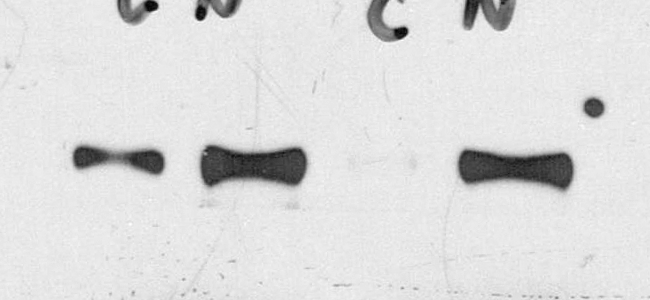

Supplement: Figure 6—figure supplement 4—source data 1. [file elife-86168-fig6-figsupp4-data1.zip › Figure 6-figure supplement4 source data/figure supplement4B MEF EZH2 Cytoplasmic nuclear anti-ezh2 uncropped.tif]

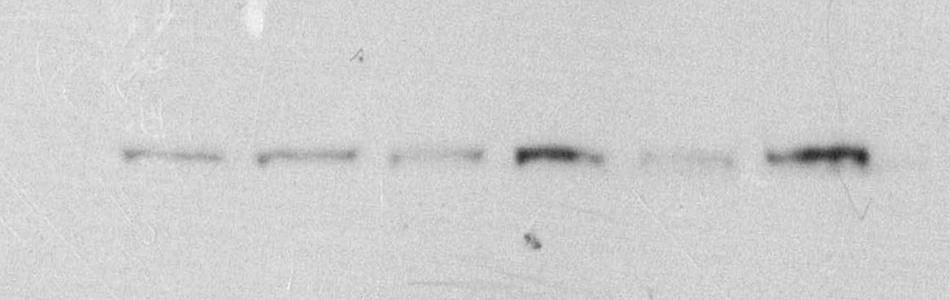

Supplement: Figure 6—figure supplement 4—source data 1. [file elife-86168-fig6-figsupp4-data1.zip › Figure 6-figure supplement4 source data/figure supplement4D MEFs treated with ta day9 anti-SUZ12 Uncropped.tif]

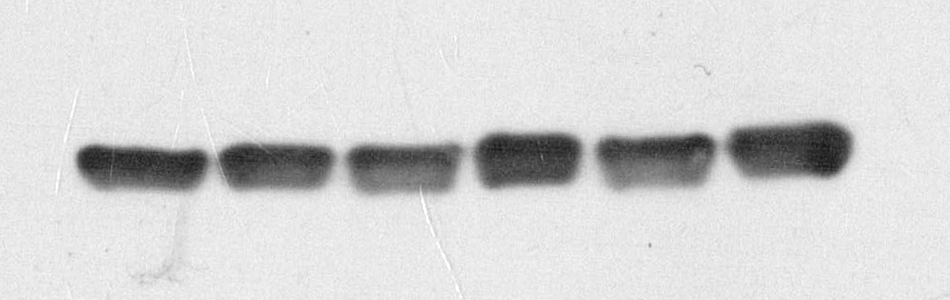

Supplement: Figure 6—figure supplement 4—source data 1. [file elife-86168-fig6-figsupp4-data1.zip › Figure 6-figure supplement4 source data/figure supplement4D MEFs treated with ta day9 anti-H3K27me3 Uncropped.tif]

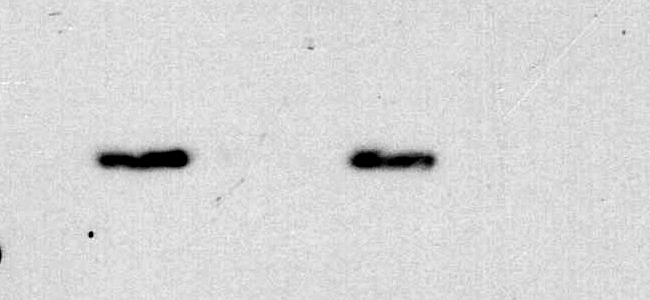

Supplement: Figure 6—figure supplement 4—source data 1. [file elife-86168-fig6-figsupp4-data1.zip › Figure 6-figure supplement4 source data/figure supplement4B MEF EZH2 Cytoplasmic nuclear anti-Tubulin uncropped.tif]

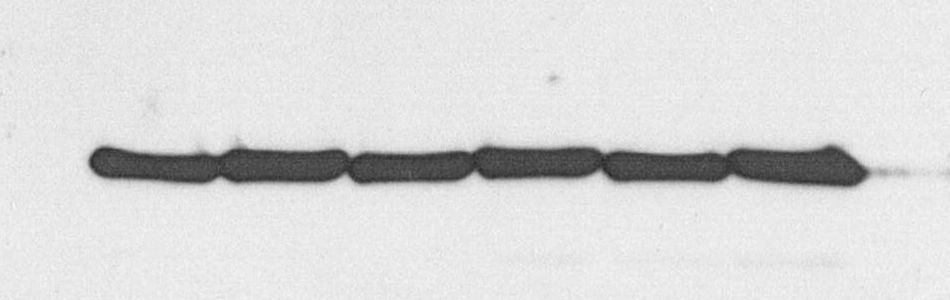

Supplement: Figure 6—figure supplement 4—source data 1. [file elife-86168-fig6-figsupp4-data1.zip › Figure 6-figure supplement4 source data/figure supplement4D MEFs treated with ta day9 anti-GAPDH Uncropped.tif]

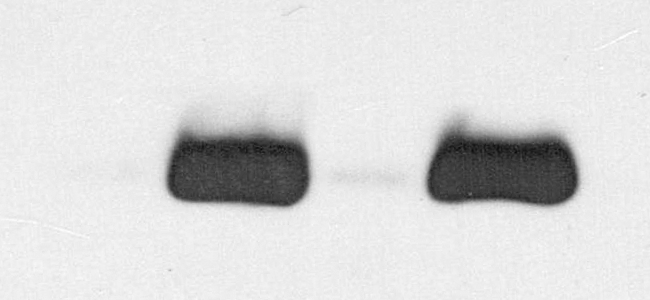

Supplement: Figure 6—figure supplement 4—source data 1. [file elife-86168-fig6-figsupp4-data1.zip › Figure 6-figure supplement4 source data/figure supplement4B MEF EZH2 Cytoplasmic nuclear anti-H3 uncropped.tif]

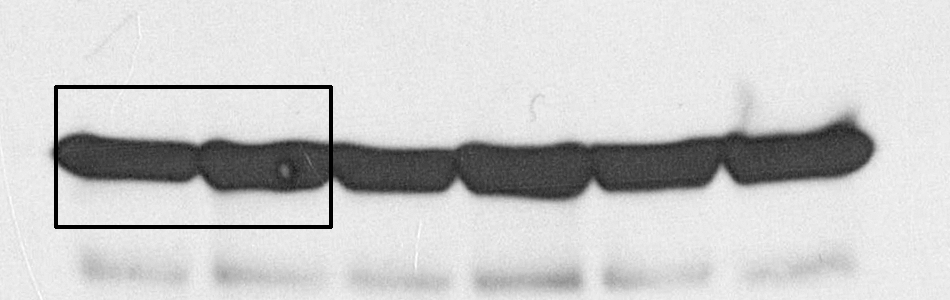

Supplement: Figure 6—figure supplement 4—source data 1. [file elife-86168-fig6-figsupp4-data1.zip › Figure 6-figure supplement4 source data/annotated/figure supplement4C MEFs treated with ta day9 anti-H3 Uncropped.tif]

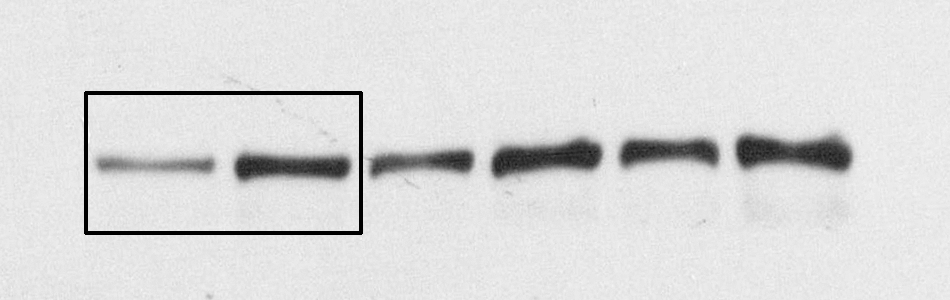

Supplement: Figure 6—figure supplement 4—source data 1. [file elife-86168-fig6-figsupp4-data1.zip › Figure 6-figure supplement4 source data/annotated/figure supplement4C MEFs treated with ta day9 anti-EZH2 Uncropped.tif]

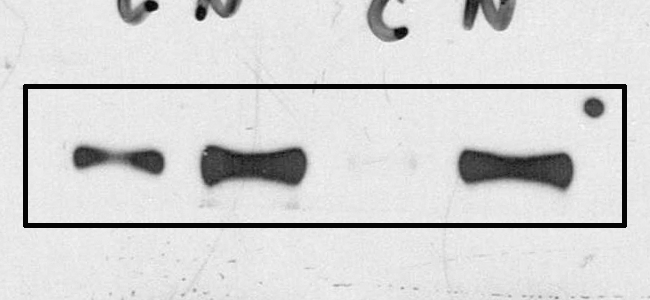

Supplement: Figure 6—figure supplement 4—source data 1. [file elife-86168-fig6-figsupp4-data1.zip › Figure 6-figure supplement4 source data/annotated/figure supplement4B MEF EZH2 Cytoplasmic nuclear anti-ezh2 uncropped.tif]

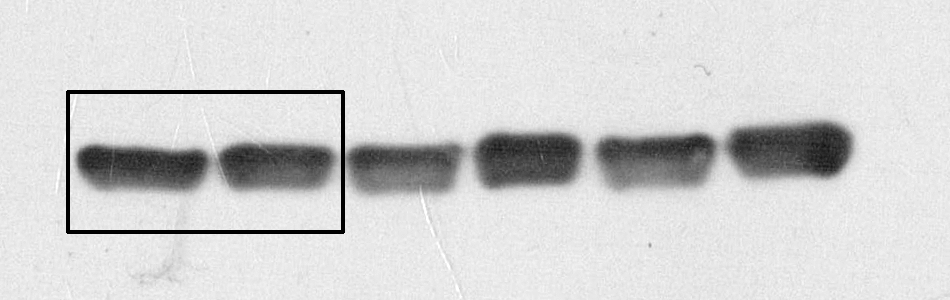

Supplement: Figure 6—figure supplement 4—source data 1. [file elife-86168-fig6-figsupp4-data1.zip › Figure 6-figure supplement4 source data/annotated/figure supplement4C MEFs treated with ta day9 anti-H3K27me3 Uncropped.tif]

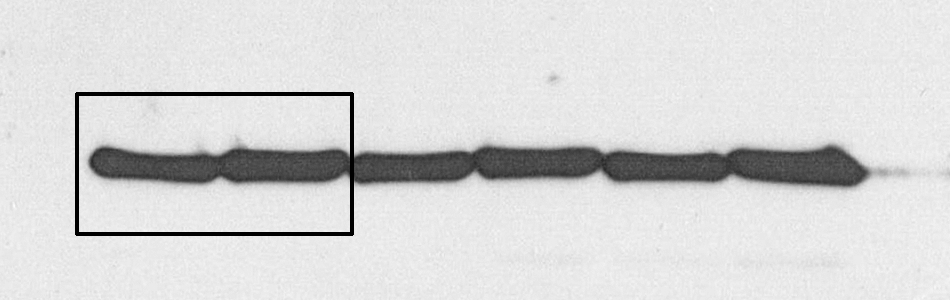

Supplement: Figure 6—figure supplement 4—source data 1. [file elife-86168-fig6-figsupp4-data1.zip › Figure 6-figure supplement4 source data/annotated/figure supplement4C MEFs treated with ta day9 anti-GAPDH Uncropped.tif]

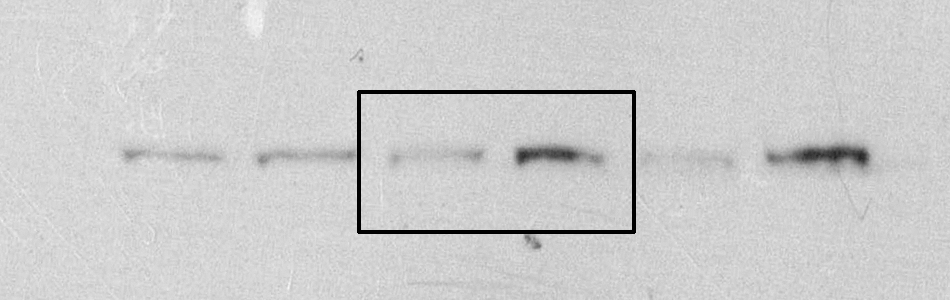

Supplement: Figure 6—figure supplement 4—source data 1. [file elife-86168-fig6-figsupp4-data1.zip › Figure 6-figure supplement4 source data/annotated/figure supplement4C MEFs treated with ta day9 anti-SUZ12 Uncropped.tif]

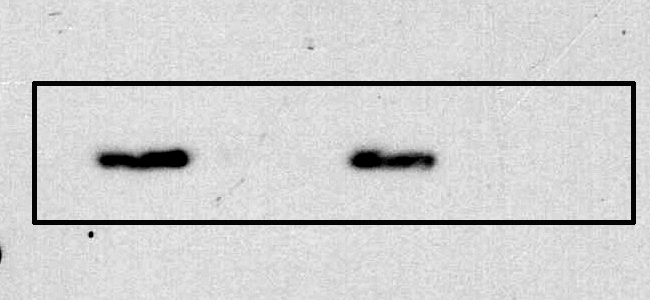

Supplement: Figure 6—figure supplement 4—source data 1. [file elife-86168-fig6-figsupp4-data1.zip › Figure 6-figure supplement4 source data/annotated/figure supplement4B MEF EZH2 Cytoplasmic nuclear anti-Tubulin uncropped.tif]

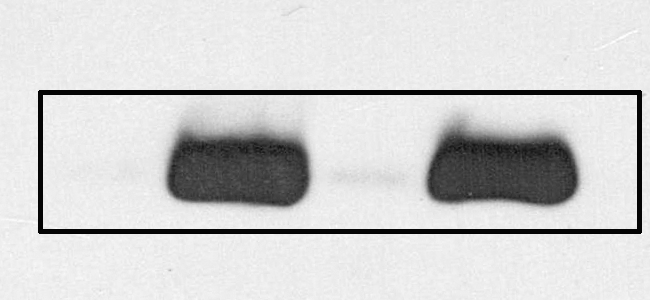

Supplement: Figure 6—figure supplement 4—source data 1. [file elife-86168-fig6-figsupp4-data1.zip › Figure 6-figure supplement4 source data/annotated/figure supplement4B MEF EZH2 Cytoplasmic nuclear anti-H3 uncropped.tif]

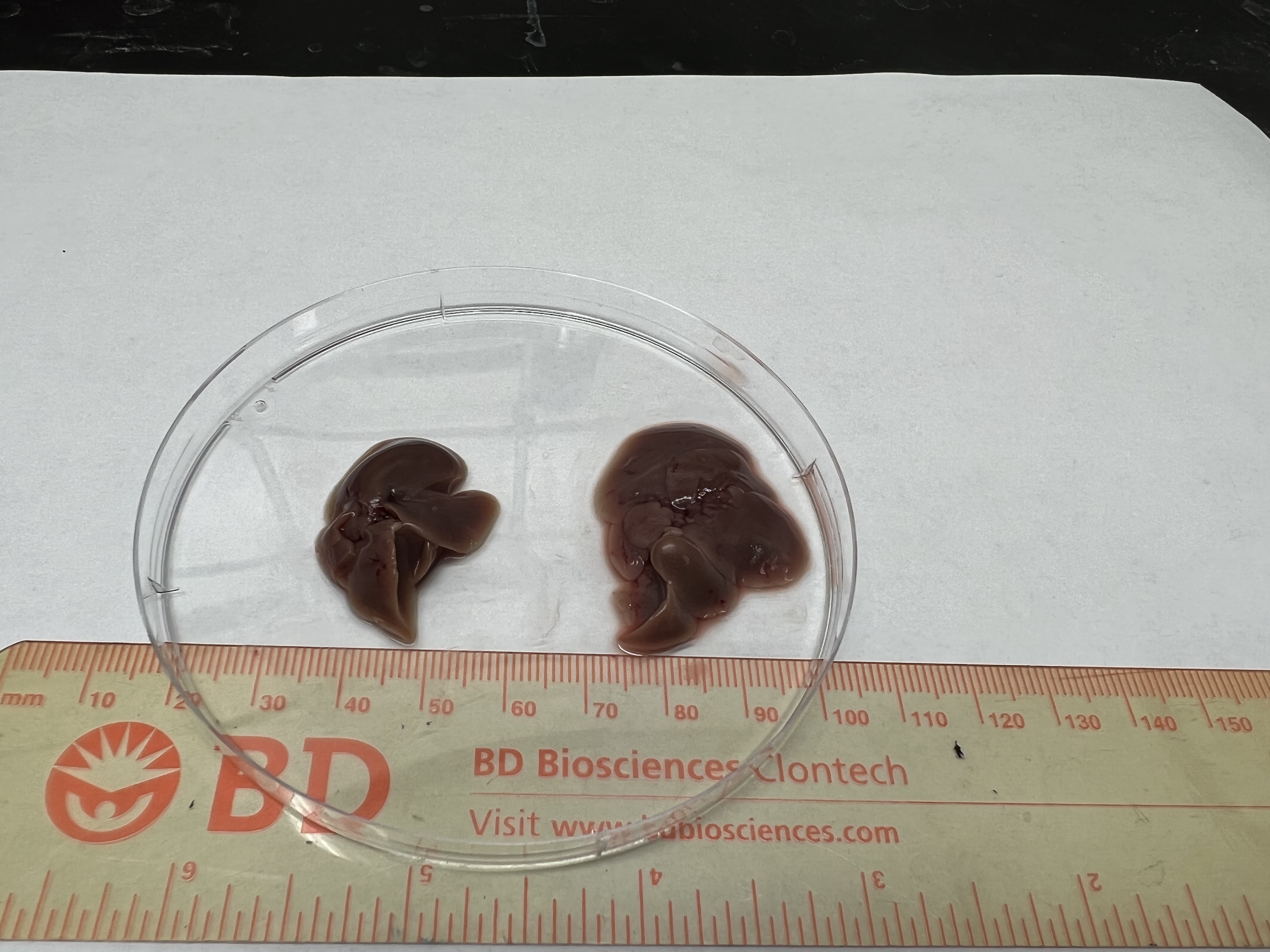

Supplement: Figure 7—source data 1. [file elife-86168-fig7-data1.zip › Figure 7 source data 1/Fig. 7A IMG-4046.jpg]

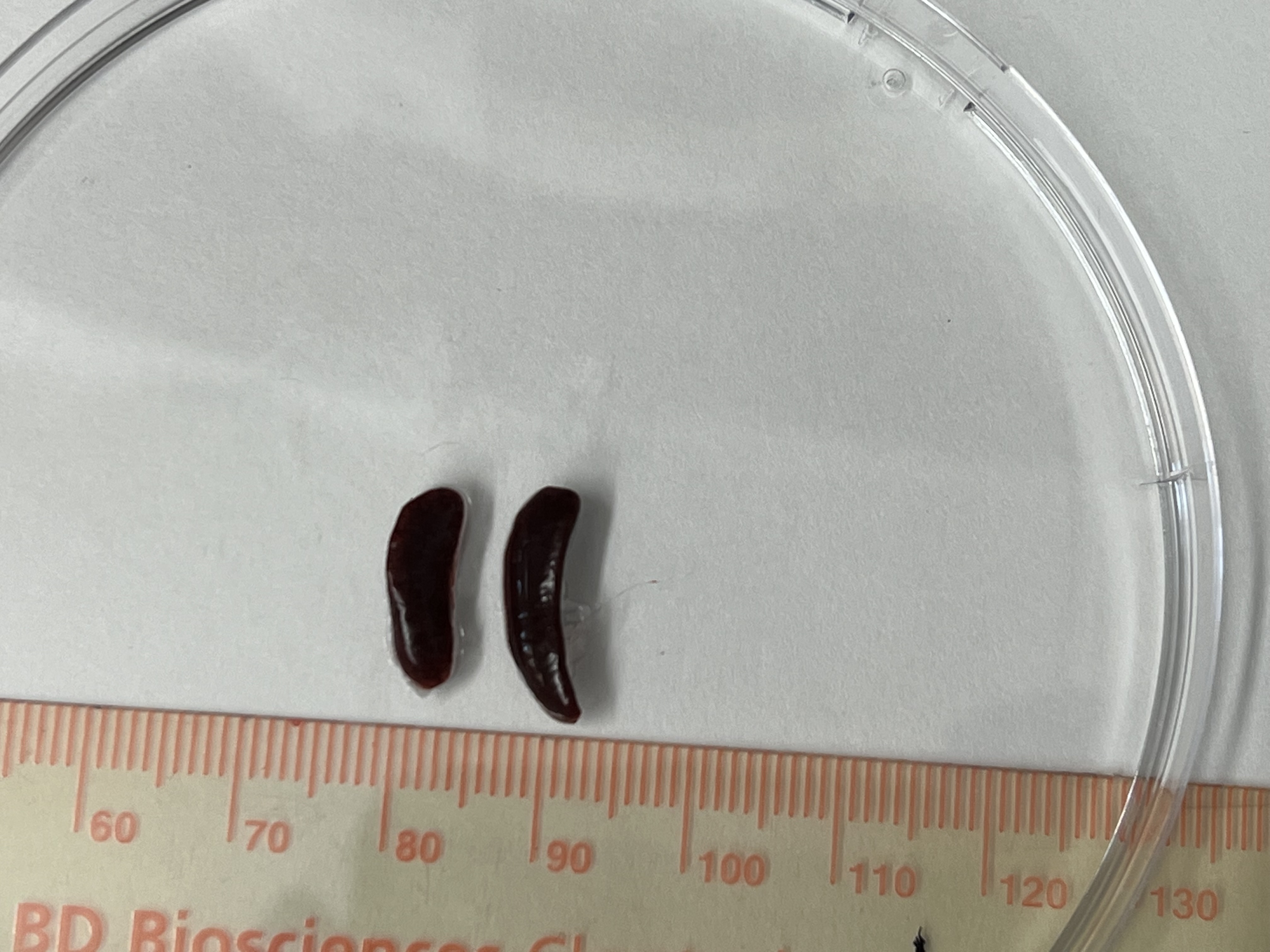

Supplement: Figure 7—source data 1. [file elife-86168-fig7-data1.zip › Figure 7 source data 1/Fig. 7B IMG-4024.jpg]

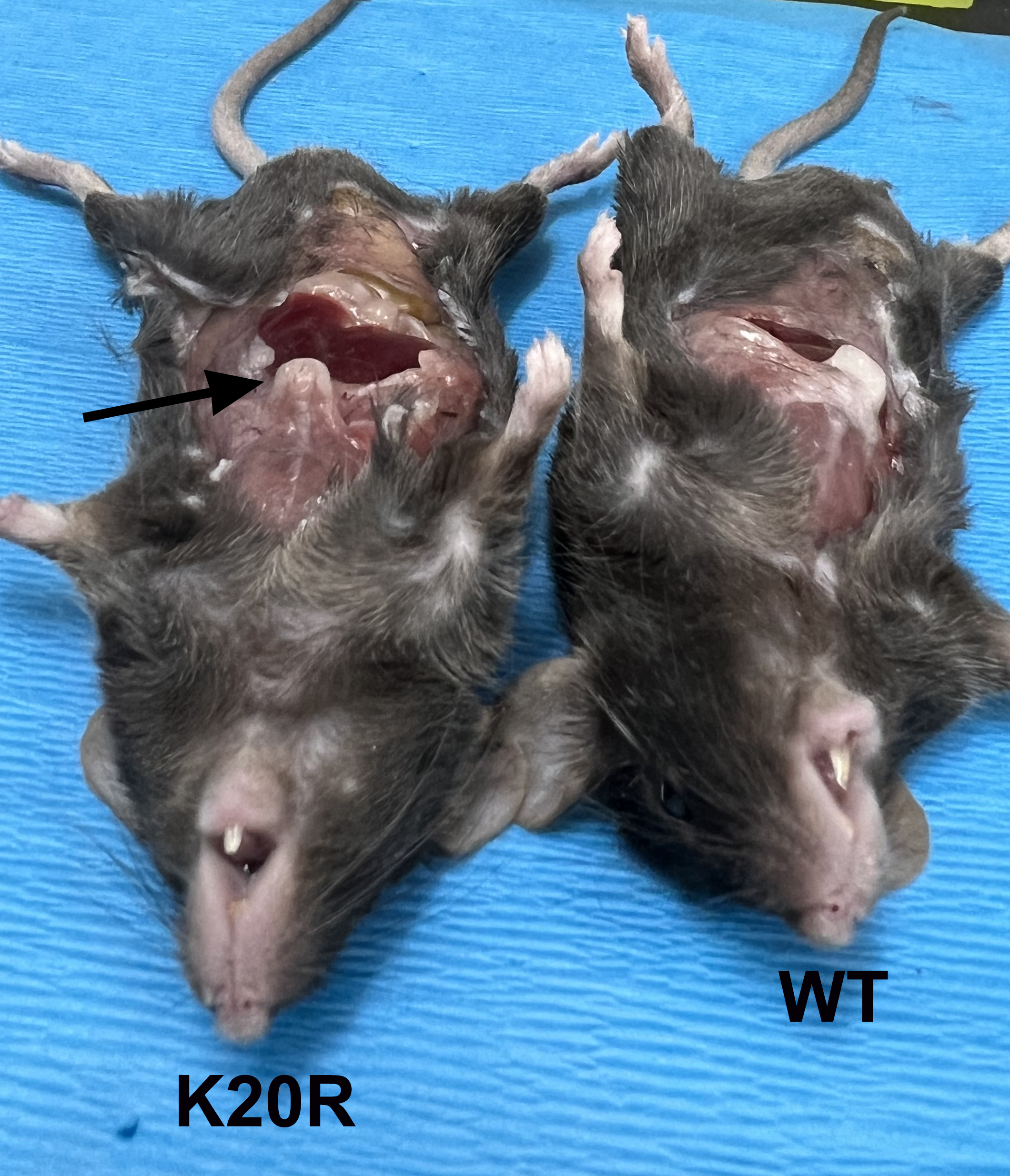

Supplement: Figure 7—figure supplement 2—source data 1. [file elife-86168-fig7-figsupp2-data1.zip › Figure 7-figure supplement2 source data 1/wt k20r mouse xiphoid process.tif]

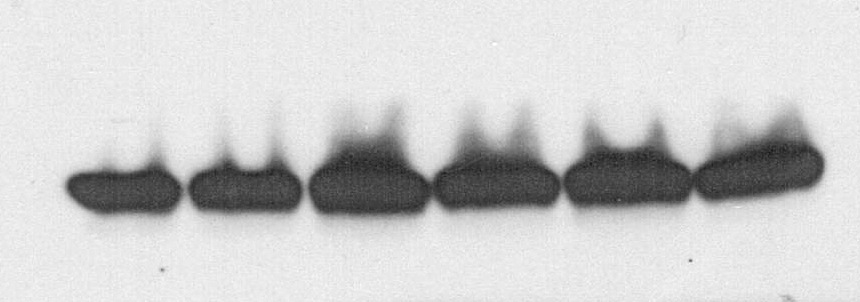

Supplement: Figure 8—source data 1. [file elife-86168-fig8-data1.zip › Figure 8 source data 1/Fig.8C 20220201 4months spleen ezh2 wt k20r anti-panAKT uncropped.tif]

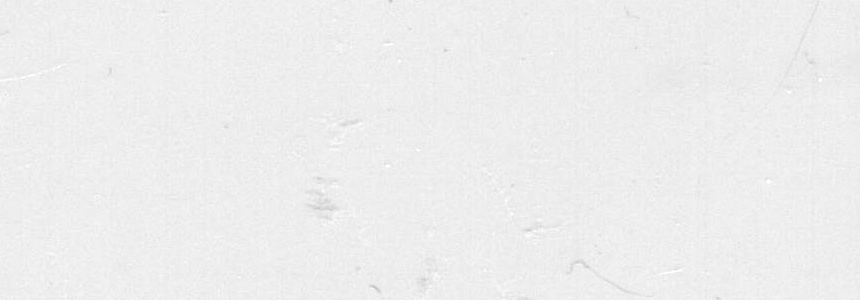

Supplement: Figure 8—source data 1. [file elife-86168-fig8-data1.zip › Figure 8 source data 1/Fig.8C 20220201 4months spleen ezh2 wt k20r anti-pS473 AKT uncropped.tif]

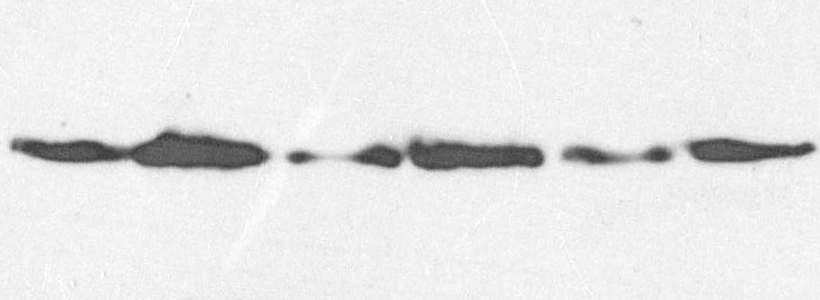

Supplement: Figure 8—source data 1. [file elife-86168-fig8-data1.zip › Figure 8 source data 1/Fig.8E k20r spleen 12weeks anti-EED 1 uncropped.tif]

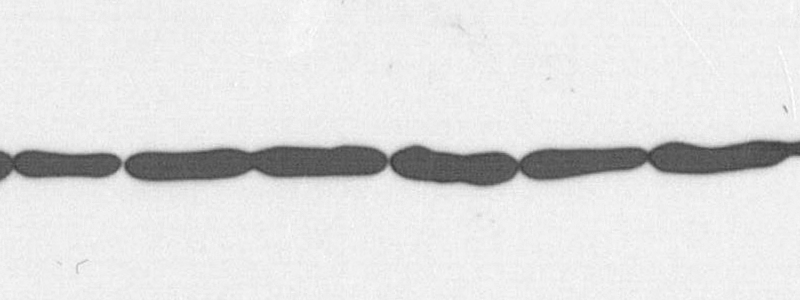

Supplement: Figure 8—source data 1. [file elife-86168-fig8-data1.zip › Figure 8 source data 1/Fig.8E k20r spleen 12weeks anti-actin uncropped.tif]

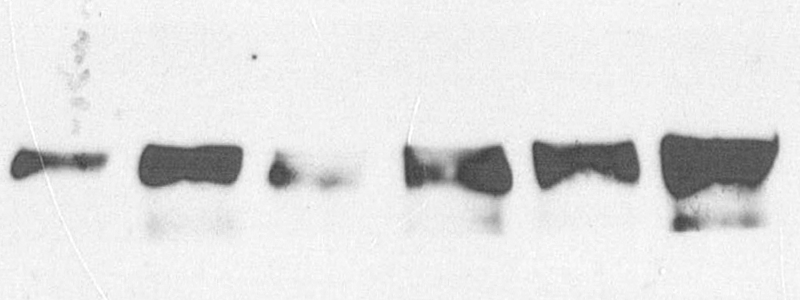

Supplement: Figure 8—source data 1. [file elife-86168-fig8-data1.zip › Figure 8 source data 1/Fig.8E k20r spleen 12weeks anti-EZH2 1 uncropped.tif]

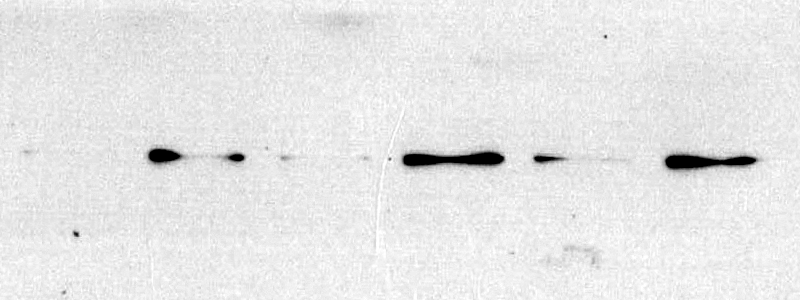

Supplement: Figure 8—source data 1. [file elife-86168-fig8-data1.zip › Figure 8 source data 1/Fig.8E k20r spleen 12weeks anti-SUZ12 uncropped.tif]

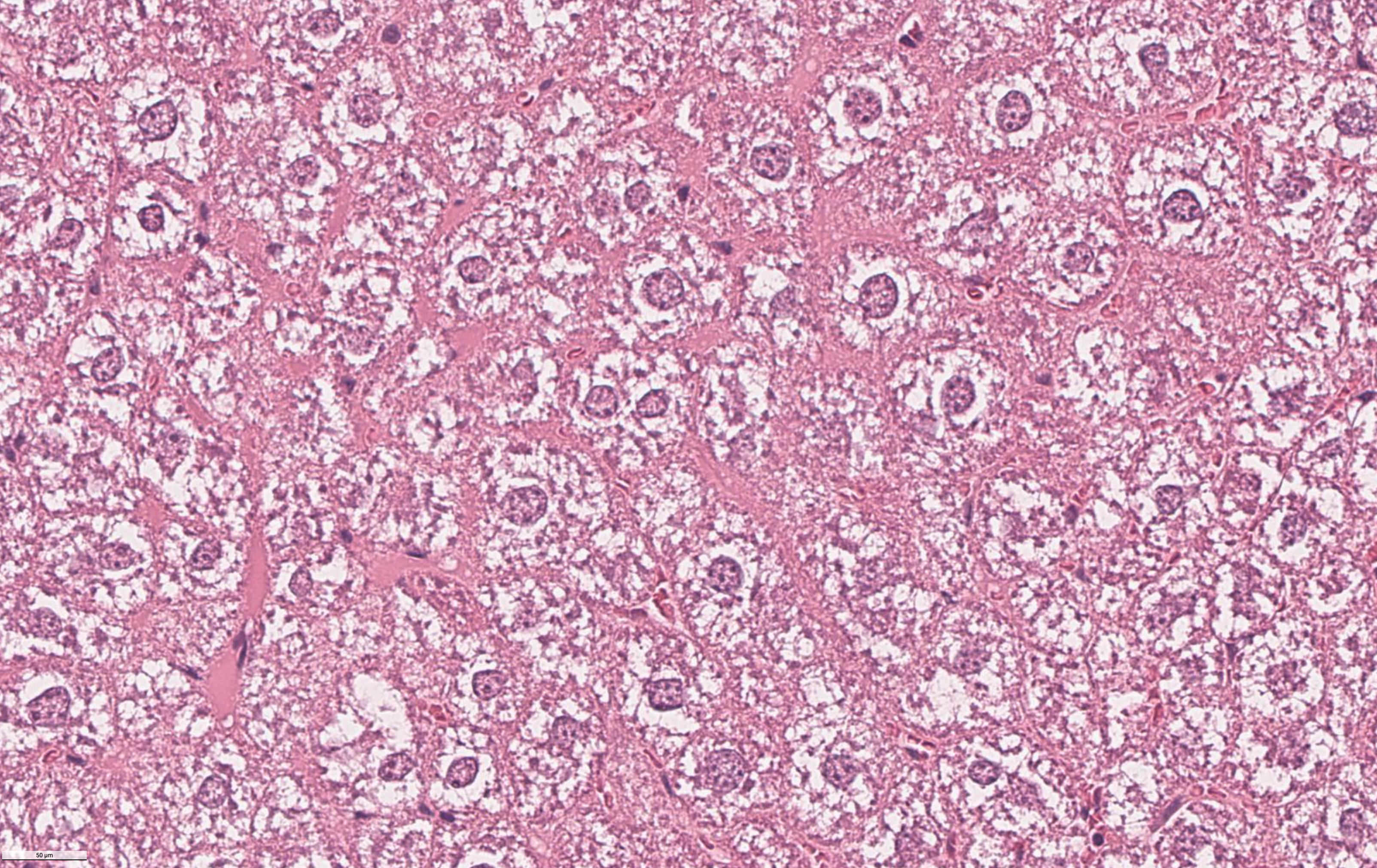

Supplement: Figure 8—source data 1. [file elife-86168-fig8-data1.zip › Figure 8 source data 1/Fig. 8A k20r liver.png]

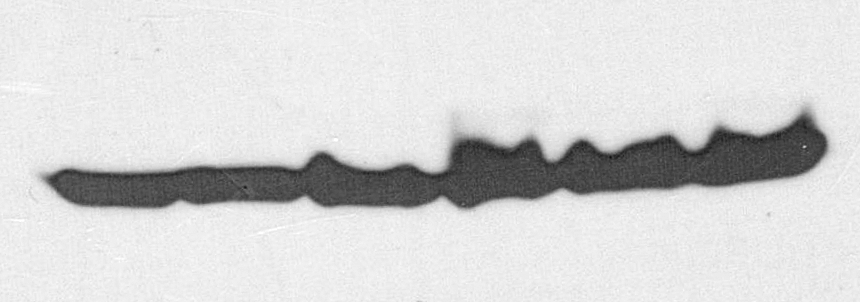

Supplement: Figure 8—source data 1. [file elife-86168-fig8-data1.zip › Figure 8 source data 1/Fig.8C 20220201 4months spleen ezh2 wt k20r anti-H3 uncropped.tif]

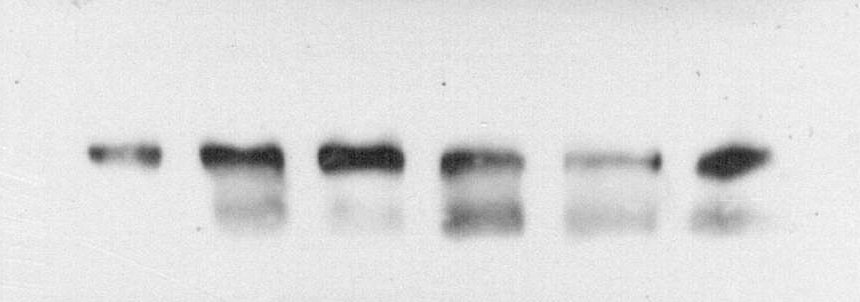

Supplement: Figure 8—source data 1. [file elife-86168-fig8-data1.zip › Figure 8 source data 1/Fig.8C 20220201 4months spleen ezh2 wt k20r anti-ezh2 uncropped.tif]

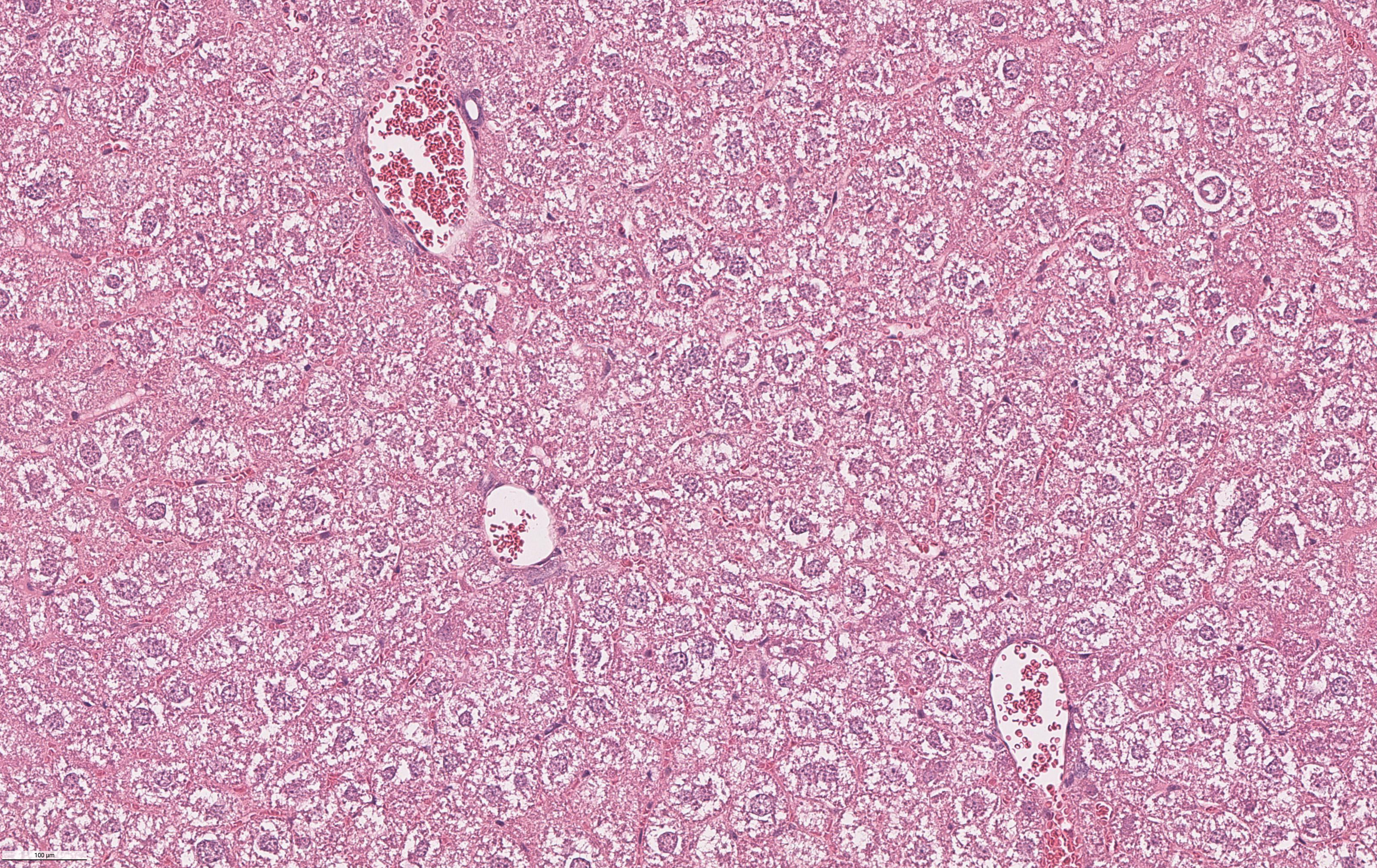

Supplement: Figure 8—source data 1. [file elife-86168-fig8-data1.zip › Figure 8 source data 1/Fig. 8A wt liver.png]

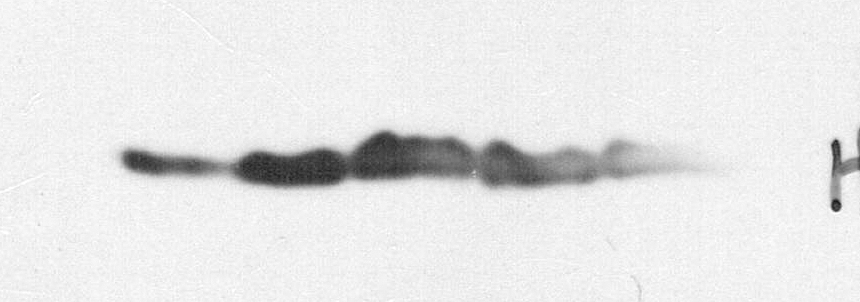

Supplement: Figure 8—source data 1. [file elife-86168-fig8-data1.zip › Figure 8 source data 1/Fig.8C 20220201 4months spleen ezh2 wt k20r anti-H3K27me3 uncropped.tif]

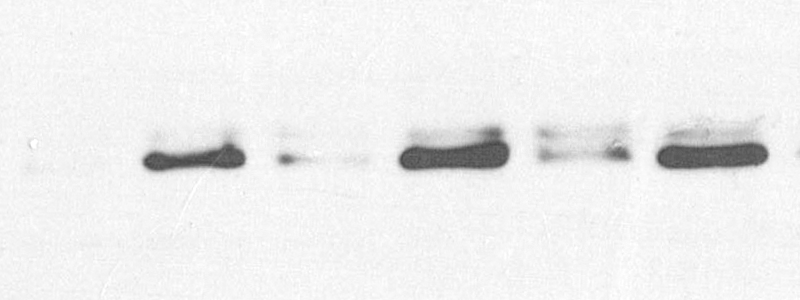

Supplement: Figure 8—source data 1. [file elife-86168-fig8-data1.zip › Figure 8 source data 1/Fig.8E k20r spleen 12weeks anti-GFI1B uncropped.tif]

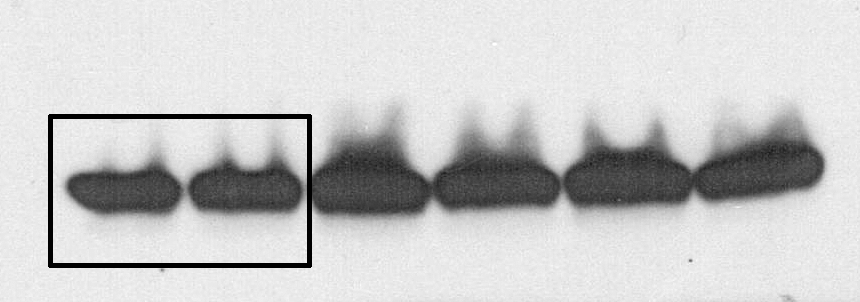

Supplement: Figure 8—source data 1. [file elife-86168-fig8-data1.zip › Figure 8 source data 1/Annotated/Fig.8C 20220201 4months spleen ezh2 wt k20r anti-panAKT uncropped.tif]

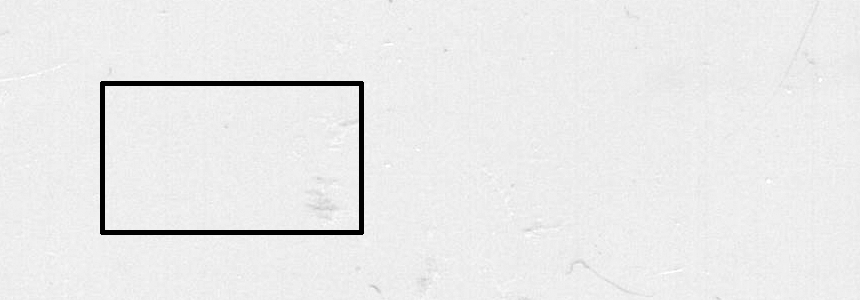

Supplement: Figure 8—source data 1. [file elife-86168-fig8-data1.zip › Figure 8 source data 1/Annotated/Fig.8C 20220201 4months spleen ezh2 wt k20r anti-pS473 AKT uncropped.tif]

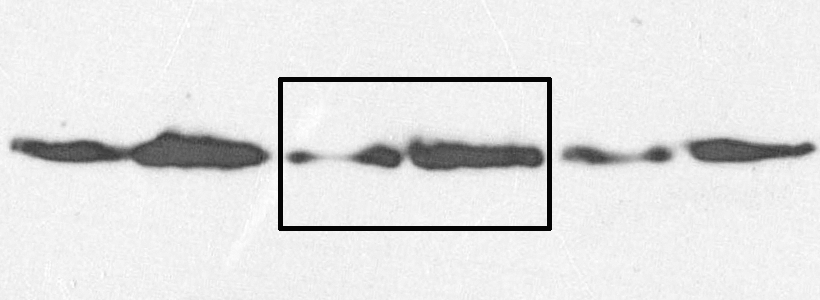

Supplement: Figure 8—source data 1. [file elife-86168-fig8-data1.zip › Figure 8 source data 1/Annotated/Fig.8E k20r spleen 12weeks anti-EED 1 uncropped.tif]

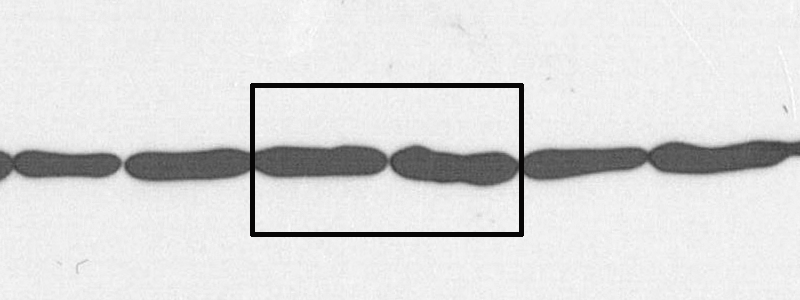

Supplement: Figure 8—source data 1. [file elife-86168-fig8-data1.zip › Figure 8 source data 1/Annotated/Fig.8E k20r spleen 12weeks anti-actin uncropped.tif]

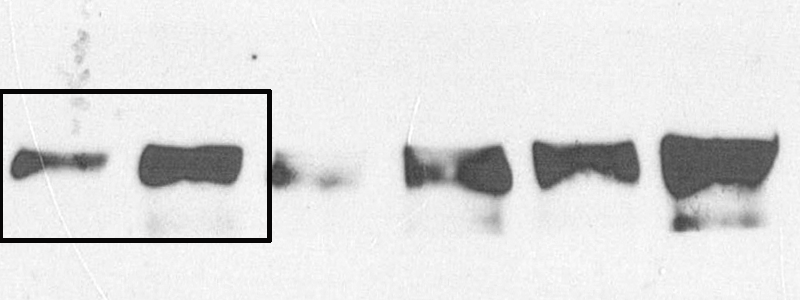

Supplement: Figure 8—source data 1. [file elife-86168-fig8-data1.zip › Figure 8 source data 1/Annotated/Fig.8E k20r spleen 12weeks anti-EZH2 1 uncropped.tif]

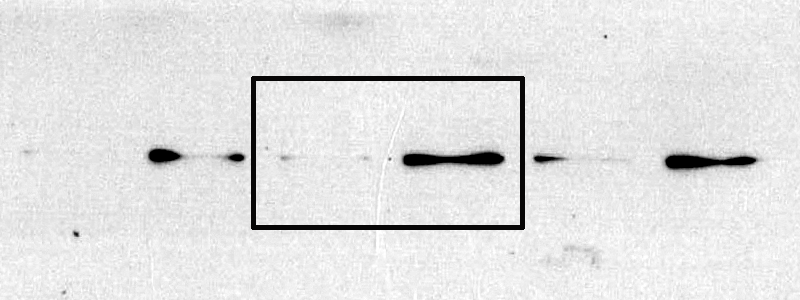

Supplement: Figure 8—source data 1. [file elife-86168-fig8-data1.zip › Figure 8 source data 1/Annotated/Fig.8E k20r spleen 12weeks anti-SUZ12 uncropped.tif]
