# Supplementary material for: A methylation-phosphorylation switch controls EZH2 stability and hematopoiesis
Source: eLife. 2024 Feb 12;13:e86168. doi: 10.7554/eLife.86168 (PMC10901513; doi:10.7554/eLife.86168)

Figure 6-figure supplement 2A-EZH2

High exposure

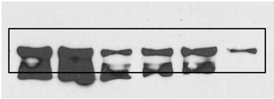

Low exposure

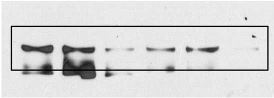

Figure 6-figure supplement 2A-L3MBTL3

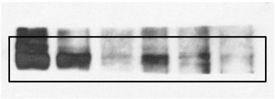

Figure 6-figure supplement 2A-LSD1

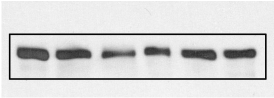

Figure 6-figure supplement 2A-Actin

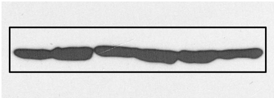

Figure6-Figure supplement 2B -EZH2

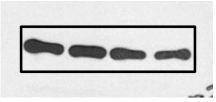

Figure6-Figure supplement 2B -EZH2-K20me

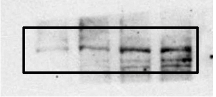

Figure6-Figure supplement 2B-EZH2-S21p

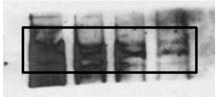

Figure6-Figure supplement 2B-pS473-AKT

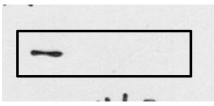

Figure6-Figure supplement 2B-AKT

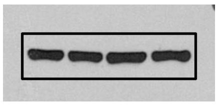

Figure6-Figure supplement 2B-H3K27me3

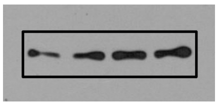

Figure6-Figure supplement 2B-H3

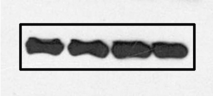

Figure6-Figure supplement 2C-EZH2

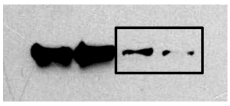

Figure6-Figure supplement 2C-EZH2-K20me

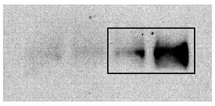

Figure6-Figure supplement 2C-EZH2-S21p

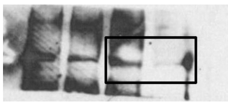

Figure6-Figure supplement 2C-pS473-AKT

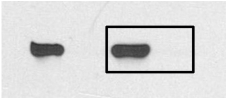

Figure6-Figure supplement 2C-AKT

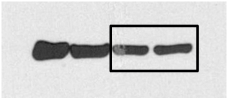

Figure6-Figure supplement 2C-H3K27me3

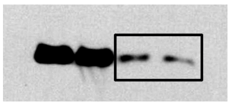

Figure6-Figure supplement 2C-H3

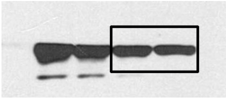

Supplement: Figure 6—figure supplement 2—source data 1. [file elife-86168-fig6-figsupp2-data1.zip › Figure 6-figure supplement2 source data 1/Figure 6-figure supplement 2.pdf]
