# Supplementary material for: A methylation-phosphorylation switch controls EZH2 stability and hematopoiesis
Source: eLife. 2024 Feb 12;13:e86168. doi: 10.7554/eLife.86168 (PMC10901513; doi:10.7554/eLife.86168)

Figure 6-figure supplement 4B-EZH2

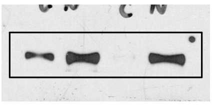

Figure 6-figure supplement 4B-H3

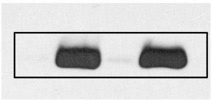

Figure 6-figure supplement 4B-Tubulin

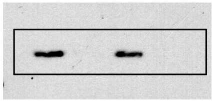

Figure 6-figure supplement 4C-EZH2

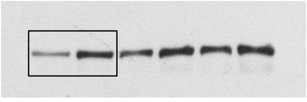

Figure 6-figure supplement 4C-SUZ12

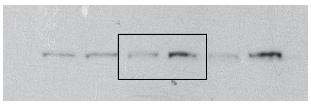

Figure 6-figure supplement 4C-GAPDH

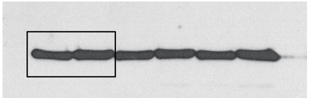

Figure 6-figure supplement 4C-H3K27me3

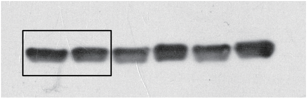

Figure 6-figure supplement 4C-H3

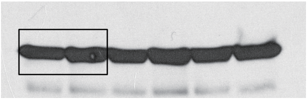

Supplement: Figure 6—figure supplement 4—source data 1. [file elife-86168-fig6-figsupp4-data1.zip › Figure 6-figure supplement4 source data/Figure 6-figure supplement 4.pdf]
