# Supplementary material for: A methylation-phosphorylation switch controls EZH2 stability and hematopoiesis
Source: eLife. 2024 Feb 12;13:e86168. doi: 10.7554/eLife.86168 (PMC10901513; doi:10.7554/eLife.86168)

Figure 8C-EZH2

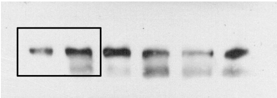

Figure 8C-H3K27me3

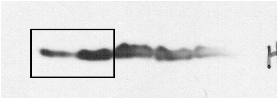

Figure 8C-H3

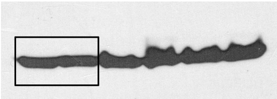

Figure 8C-pS473-AKT

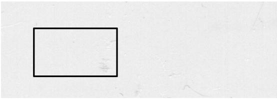

Figure 8C-pan-AKT

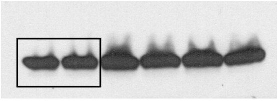

Figure 8E-EZH2

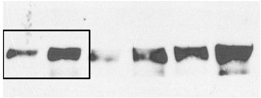

Figure 8E-EED

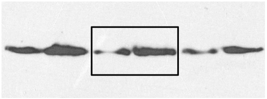

Figure 8E-SUZ12

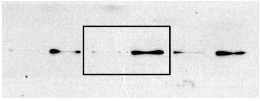

Figure 8E-GFI1B

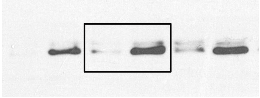

Figure 8E-pan-Actin

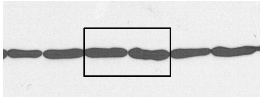

Supplement: Figure 8—source data 1. [file elife-86168-fig8-data1.zip › Figure 8 source data 1/Figure 8-annotated source data .pdf]
