# Supplementary figures and images for: A methylation-phosphorylation switch controls EZH2 stability and hematopoiesis (part 7 of 7)
Source: eLife. 2024 Feb 12;13:e86168. doi: 10.7554/eLife.86168 (PMC10901513; doi:10.7554/eLife.86168)

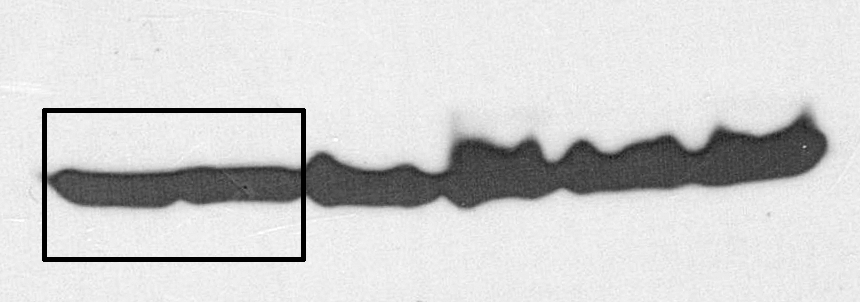

Supplement: Figure 8—source data 1. [file elife-86168-fig8-data1.zip › Figure 8 source data 1/Annotated/Fig.8C 20220201 4months spleen ezh2 wt k20r anti-H3 uncropped.tif]

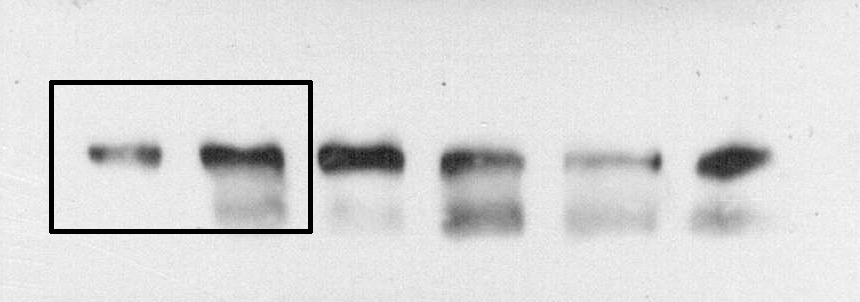

Supplement: Figure 8—source data 1. [file elife-86168-fig8-data1.zip › Figure 8 source data 1/Annotated/Fig.8C 20220201 4months spleen ezh2 wt k20r anti-ezh2 uncropped.tif]

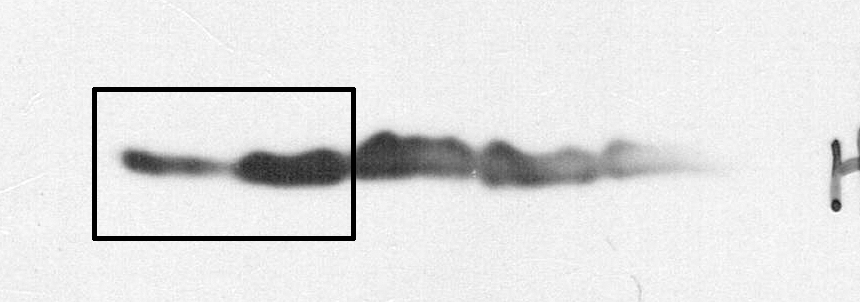

Supplement: Figure 8—source data 1. [file elife-86168-fig8-data1.zip › Figure 8 source data 1/Annotated/Fig.8C 20220201 4months spleen ezh2 wt k20r anti-H3K27me3 uncropped.tif]

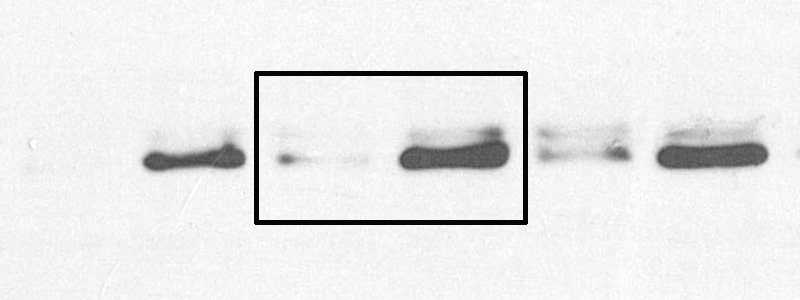

Supplement: Figure 8—source data 1. [file elife-86168-fig8-data1.zip › Figure 8 source data 1/Annotated/Fig.8E k20r spleen 12weeks anti-GFI1B uncropped.tif]

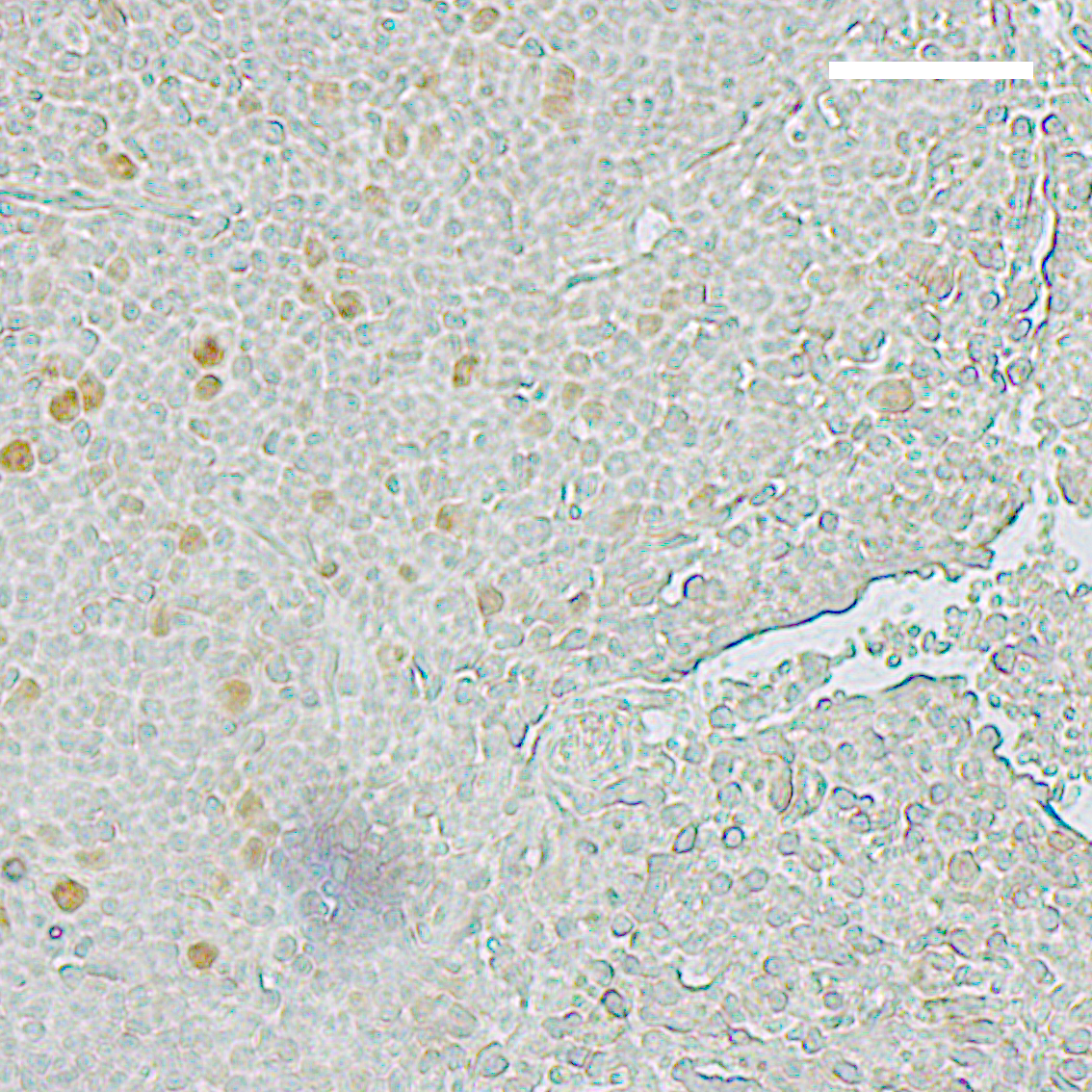

Supplement: Figure 8—source data 1. [file elife-86168-fig8-data1.zip › Figure 8 source data 1/Figure 8B/Middle panel/20221103 DAB staining for EZH2 WT 20X `04 cut with 200xpixel bar.tif]

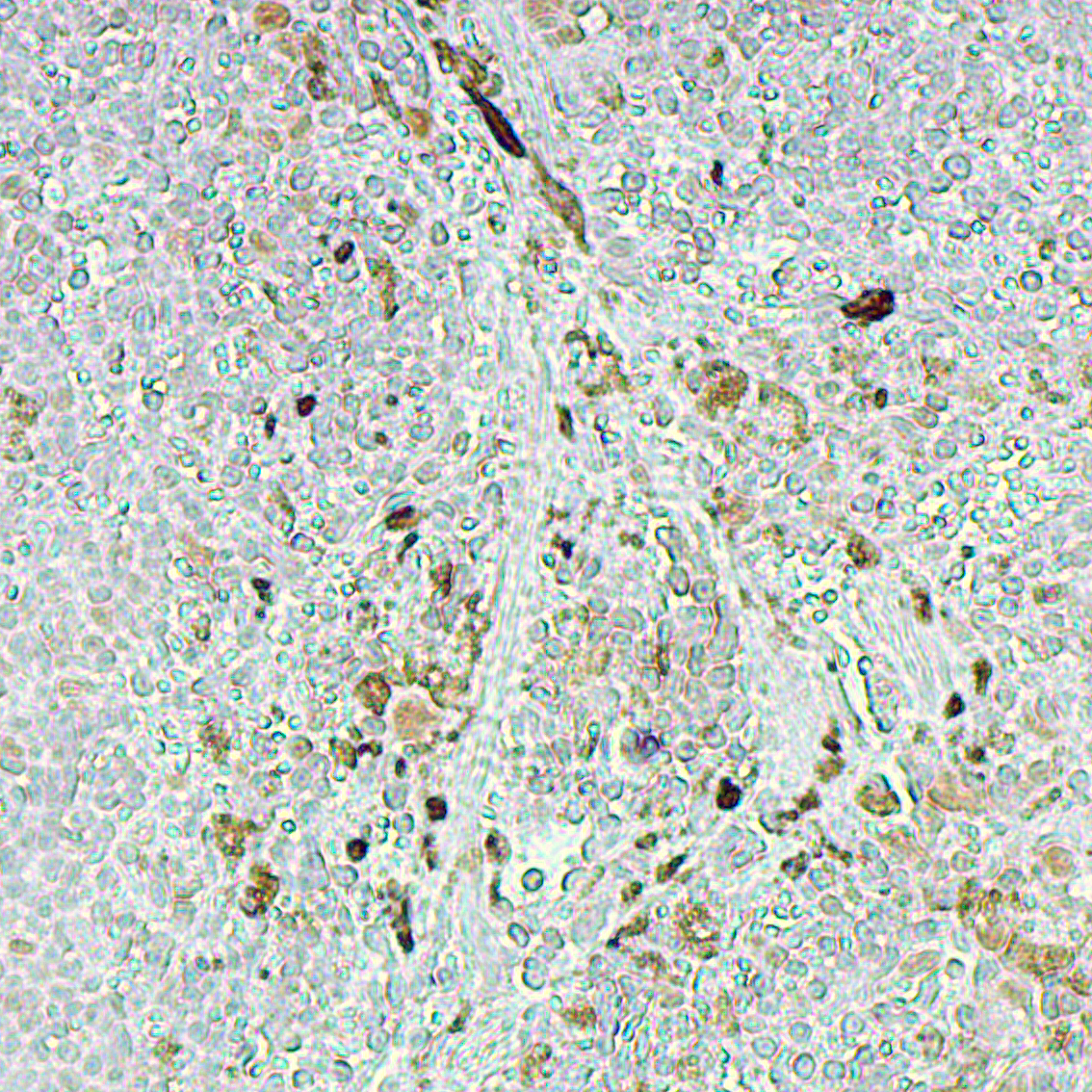

Supplement: Figure 8—source data 1. [file elife-86168-fig8-data1.zip › Figure 8 source data 1/Figure 8B/Middle panel/20221103 DAB staining for EZH2 k20r 20X 03 cut.tif]

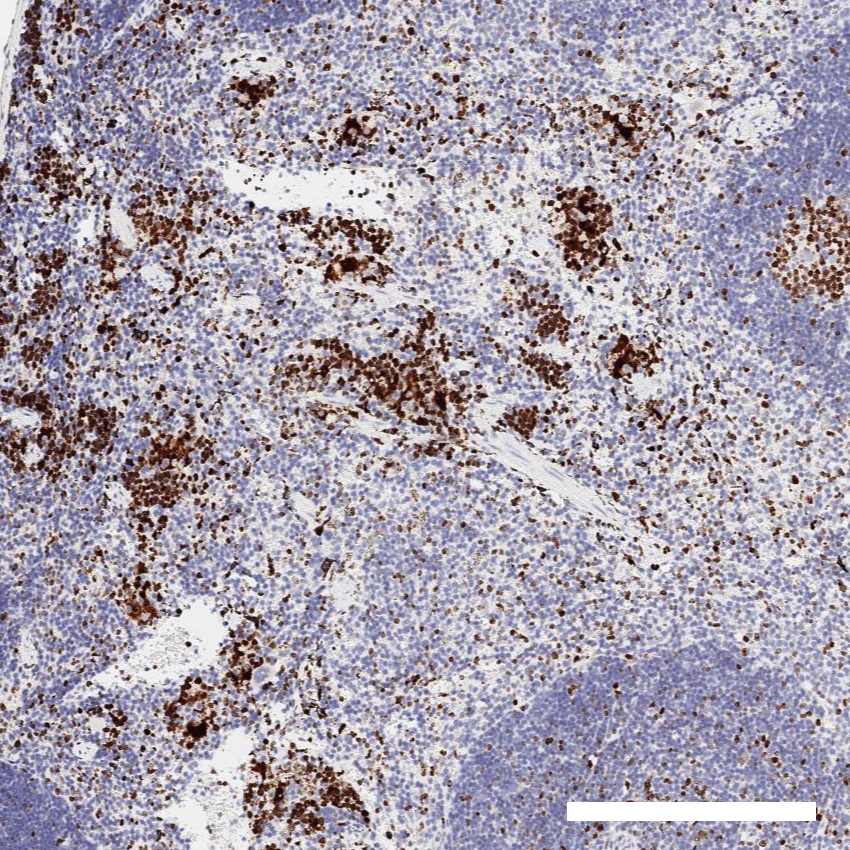

Supplement: Figure 8—source data 1. [file elife-86168-fig8-data1.zip › Figure 8 source data 1/Figure 8B/Left panel/2022-07-15 spleen K20R KI67 staining scale bar 500um.tif]

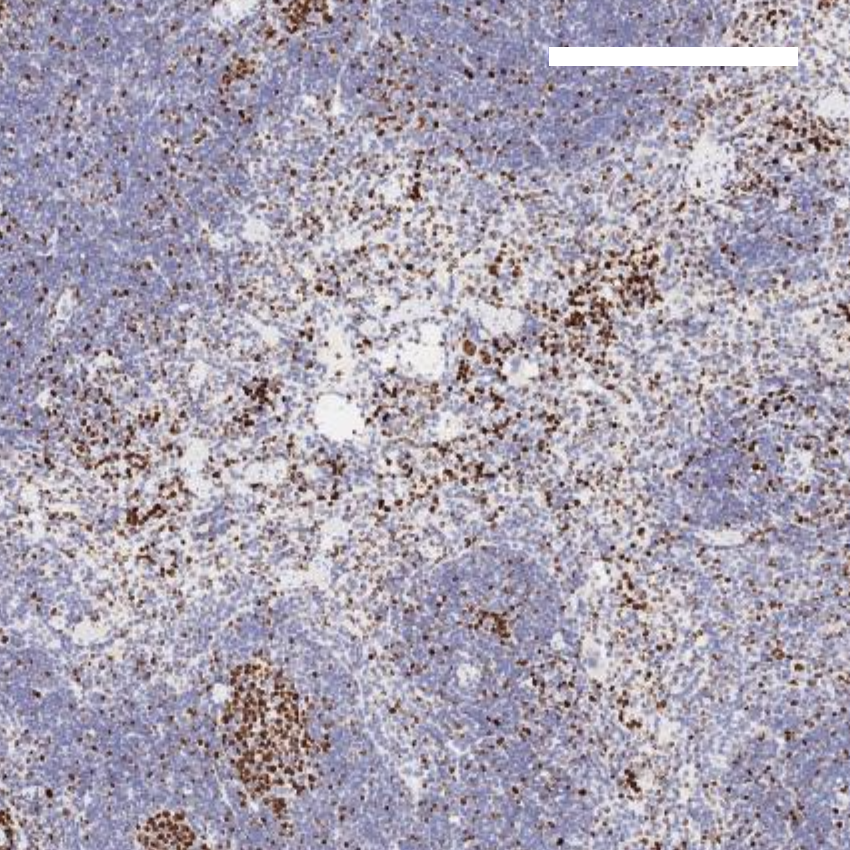

Supplement: Figure 8—source data 1. [file elife-86168-fig8-data1.zip › Figure 8 source data 1/Figure 8B/Left panel/2022-07-15 spleen WT KI67 staining scale bar 500um.tif]

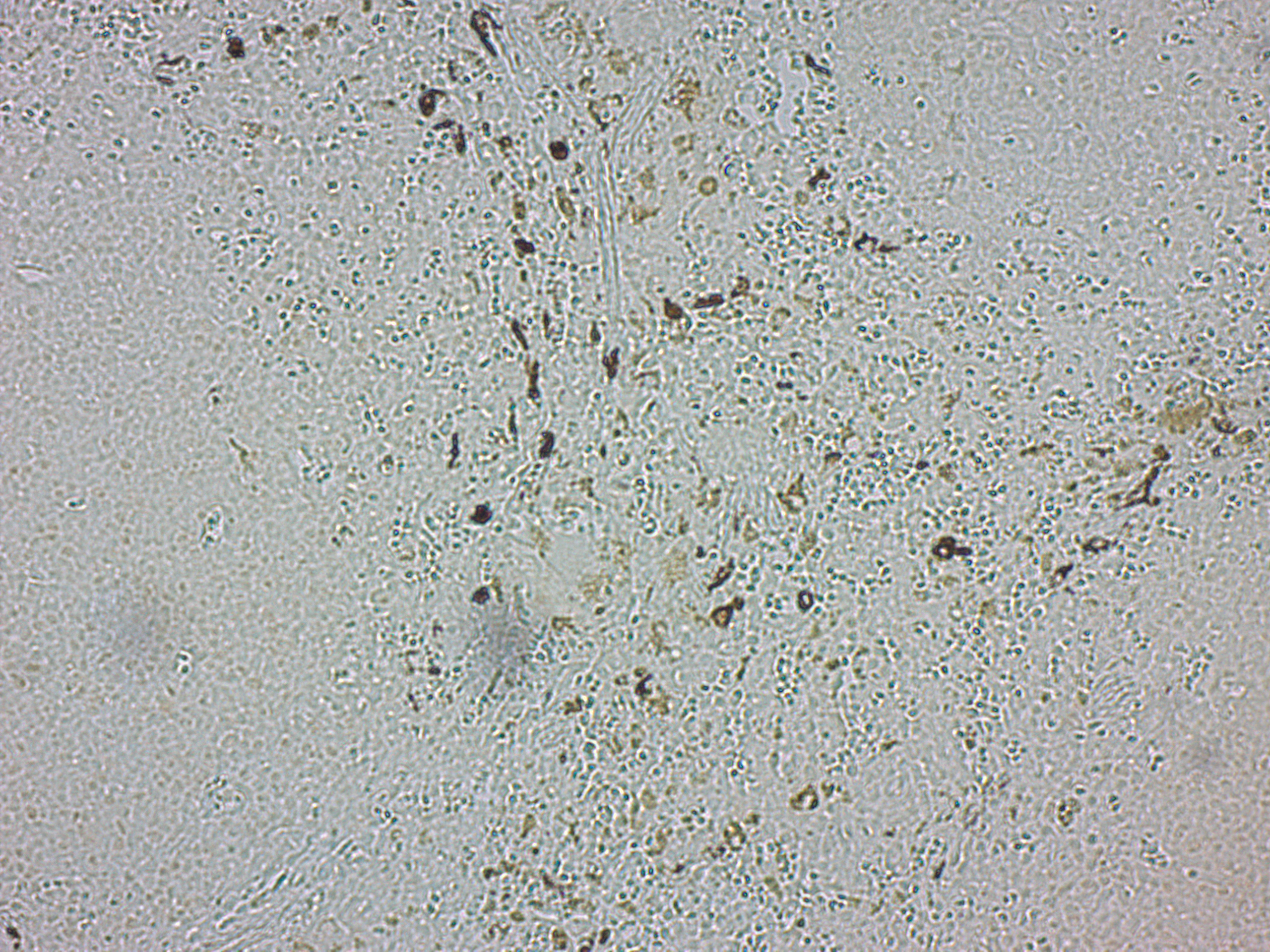

Supplement: Figure 8—source data 1. [file elife-86168-fig8-data1.zip › Figure 8 source data 1/Figure 8B/Right panel/K20R 20x 5.tif]

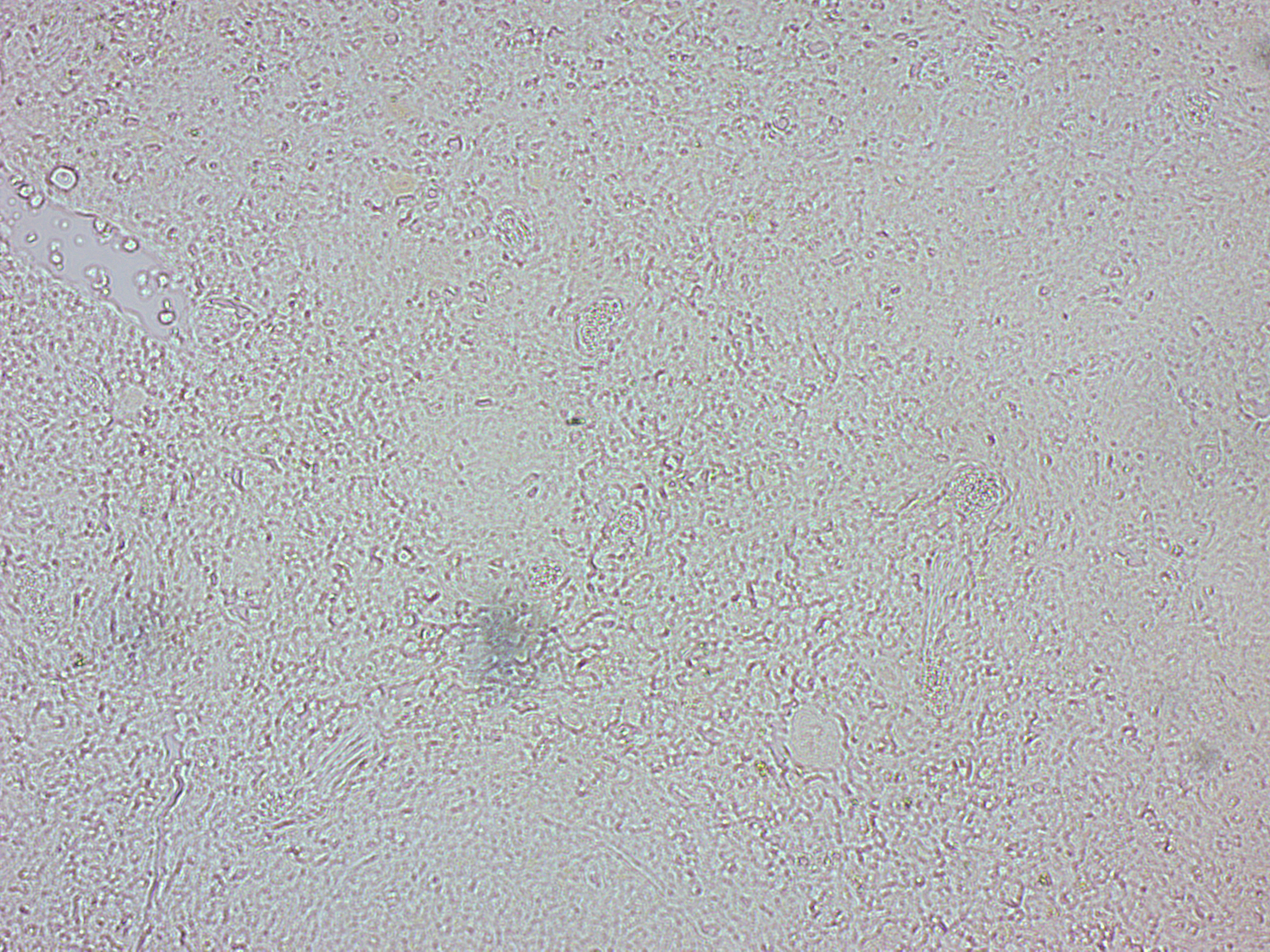

Supplement: Figure 8—source data 1. [file elife-86168-fig8-data1.zip › Figure 8 source data 1/Figure 8B/Right panel/wt 20x 56.tif]
